# Gallium in liquid state shows nuclease-mimicking activity

**DOI:** 10.1038/s41467-026-71346-7

**Published:** 2026-04-10

**Authors:** Li Liu, Jiewei Zheng, Xi Lu, Chowdhury Sarowar, Yuqin Wang, Martin A. Smith, Xin Wang, Fei Deng, Biswaranjan Mohanty, Nur-Adania Nor-Azman, Fusheng Zhang, Shih-Hao Chiu, Mario Torrado, Yi Li, Shi-Yang Tang, Jianbo Tang, Michelle J. S. Spencer, Priyank V. Kumar, Kourosh Kalantar-Zadeh, Chengchen Zhang

**Affiliations:** 1 School of Chemical and Biomolecular Engineering, University of Sydney, Sydney, New South Wales Australia; 2 School of Chemical Engineering, UNSW Sydney, Sydney, New South Wales Australia; 3 School of Science, RMIT University, Melbourne, Victoria Australia; 4 Digital Health and Biomedical Engineering, School of Electronics and Computer Science, University of Southampton, Southampton, UK; 5 Institute for Life Sciences, University of Southampton, Southampton, UK; 6 Bioanalytical Mass Spectrometry Facility, Mark Wainwright Analytical Centre, UNSW Sydney, Sydney, New South Wales Australia; 7 Ramaciotti Centre for Genomics, School of Biotechnology and Biomolecular Sciences, Faculty of Science, UNSW Sydney, Sydney, New South Wales Australia; 8 Australian Centre for Nano Medicine, UNSW Sydney, Sydney, New South Wales Australia; 9 School of Biomedical Engineering, Faculty of Engineering, University of New South Wales, Sydney, New South Wales Australia; 10 Sydney Analytical, Core Research Facilities, The University of Sydney, Sydney, Australia; 11 Center for AIE Research, Guangdong Provincial Key Laboratory of New Energy Materials Service Safety, College of Materials Science and Engineering, Shenzhen University, Shenzhen, China; 12 Key Laboratory of Clinical Laboratory Diagnostics (Ministry of Education), College of Laboratory Medicine, Chongqing Medical University, Chongqing, China; 13 School of Mechanical and Manufacturing Engineering, UNSW Sydney, Sydney, New South Wales Australia; 14 Department of Materials Science and Engineering, School of Engineering, Westlake University, Hangzhou, China

**Keywords:** Nanobiotechnology, Enzymes, Metals, Nucleases, Biocatalysis

## Abstract

Replicating biological systems using non-living materials, from the foundational molecular level to complex tissue structures, is central to abiotic mimicry. Enzymes play a vital role in these systems; however, replicating their enzymatic power with minimal components remains a key challenge. Here we show that gallium in the liquid state exhibits nuclease-like activity with preferred cleaving sites. The mechanism involves nucleotide-biased adsorption and hydroxyl radical-assisted phosphodiester hydrolysis. Compared with previously reported artificial metallonucleases, the liquid gallium uniquely integrates its oxide layer for substrate adsorption and its metallic core with electrons as a cleavage active center, forming a ligand- and cofactor-free artificial nuclease platform. Moreover, their activity is tunable through synthesis parameters and external stimuli, enabling programmable control with spatial or temporal precision. This work presents a minimalistic yet functional approach to enzyme mimicry, expanding the design space for abiotic enzymatic systems and offering potential opportunities in therapeutic applications, synthetic biology, and biomaterials.

## Abstract (web summary)

Replicating enzyme function with minimal components is a major challenge in abiotic mimicry. Here, the authors show that liquid gallium droplets act as tunable artificial nucleases, cleaving DNA with nucleotide bias via oxide-mediated adsorption and hydroxyl radical-assisted hydrolysis.

## Introduction

The creation of artificial biological systems relies on replicating biological components, and achieving this from the foundational molecular level to complex structures using non-living materials remains the central challenge in abiotic mimicry^1^. Among biological components, enzymes are indispensable for enabling specific and efficient chemical reactions^2^. Yet, reproducing their enzymatic power using minimal components, particularly without complex molecular scaffolds, presents a significant challenge^3^. To date, much of artificial enzyme research has focused on architectures such as metalloenzymes^4,5^ and ligand-supported metal complexes^6,7^. In the case of artificial metallonuclease, a type of artificial metalloenzyme, achieving nucleotide selectivity typically requires multi-metal centers or designed ligand environments^8^. Here, we introduce a fundamentally different strategy. We show the discovery that liquid gallium (Ga), in its micro/nanoscale droplet form, can mimic nuclease activity without requiring ligands or cofactors.

Liquid Ga is an important family member of liquid metals (LMs), which have recently emerged as a promising class of materials in biomedical applications, including cancer therapy^9^, wearable electronics^10^, and bioimaging^11^, due to their thermal and electrical conductivity, advantageous mechanical compatibility, and high biosafety^12,13^. In addition, LMs have gained attention as catalytically active soft materials^14^ characterized by their electron-rich and dynamic interfacial environments^15,16^. Their ability to be modified with or dissolve various metals has particularly broadened applications in catalysis^16–18^.

**Fig. 1:**
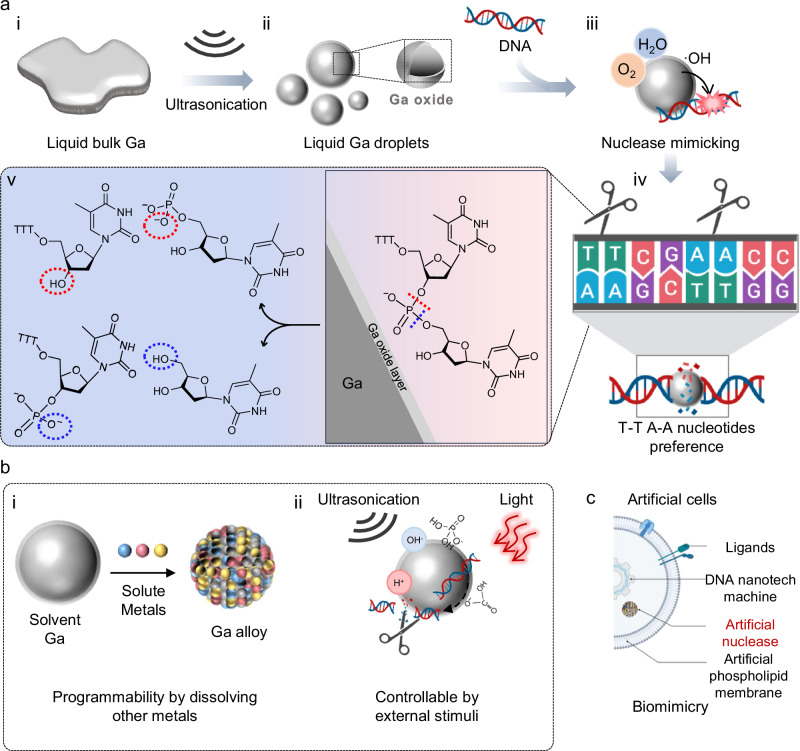
Schematic of Ga nuclease-mimicking activity. **a** Schematic of Gallium (Ga) droplet preparation (i, ii), nuclease-mimicking properties in the presence of water (H_2_O) and oxygen (O_2_) (iii) with the DNA nucleotide preference for nucleotide bases thymine (T) and adenine (A) (iv); and the underlying hydroxyl radical ( ∙ OH) -mediated hydrolytic cleavage mechanism (v). **b** Programmability through the dissolution of other metals (i) and controllability via external stimuli in biological environments (ii). **c** Schematic illustration of the proposed potential application of Ga droplets as artificial nucleases within a synthetic artificial cell environment. Some graphical elements in Fig. 1a–c were created using BioRender *Liu, L. (2026)* https://BioRender.com/8s6hpu0. Source data are provided as a Source Data file.

In this work (Fig. 1), we discover that Ga droplets, synthesized by sonication (Fig. 1ai–ii), can cleave deoxyribonucleic acid (DNA) with a preference for thymine-thymine (T-T) and adenine-adenine (A-A) sites (Fig. 1aiii–iv), which is an unprecedented opportunity for creating ligand- and cofactor-free based artificial nuclease mimics. Through a combination of experimental assays and computational simulations, we reveal that the cleavage proceeds via a nucleophilic substitution mechanism: Ga preferentially adsorbs onto the DNA backbone via electrostatic and van der Waals interactions, while the in-situ generation of hydroxyl radicals ( ∙ OH) during Ga surface oxidation facilitates phosphodiester bond cleavage (Fig. 1a,v). We then demonstrate that the nuclease-mimicking efficiency of Ga droplets can be modulated through size control, compositional tuning via alloying with other catalytically active metals, and exposure to external stimuli such as pH shifts, sonication, and laser irradiation (Fig. 1b). We also validate this nuclease-mimicking activity in various biological fluids, including serum, urine, and simulated gastric environments, highlighting the robustness and adaptability of this system. Although full sequence specificity is not achieved in this work, this degree of nucleotide-level bias represents a major departure from known artificial metallonucleases^5,8^ and demonstrates that even simple elemental materials can exhibit selective bifunctionality. Furthermore, the ease with which liquid Ga alloys with other metals^15^ to modify surface reactivity opens avenues for designing a broader range of artificial enzymes with tailored functions^19^. This insight establishes a direction for designing minimalist enzyme mimics and supports the broader goal of constructing functional abiotic systems with emergent biological capabilities (Fig. 1c).

## Results

### Discovery of Liquid Ga Droplets-Mediated Nuclease-Mimicking Activity

**Fig. 2:**
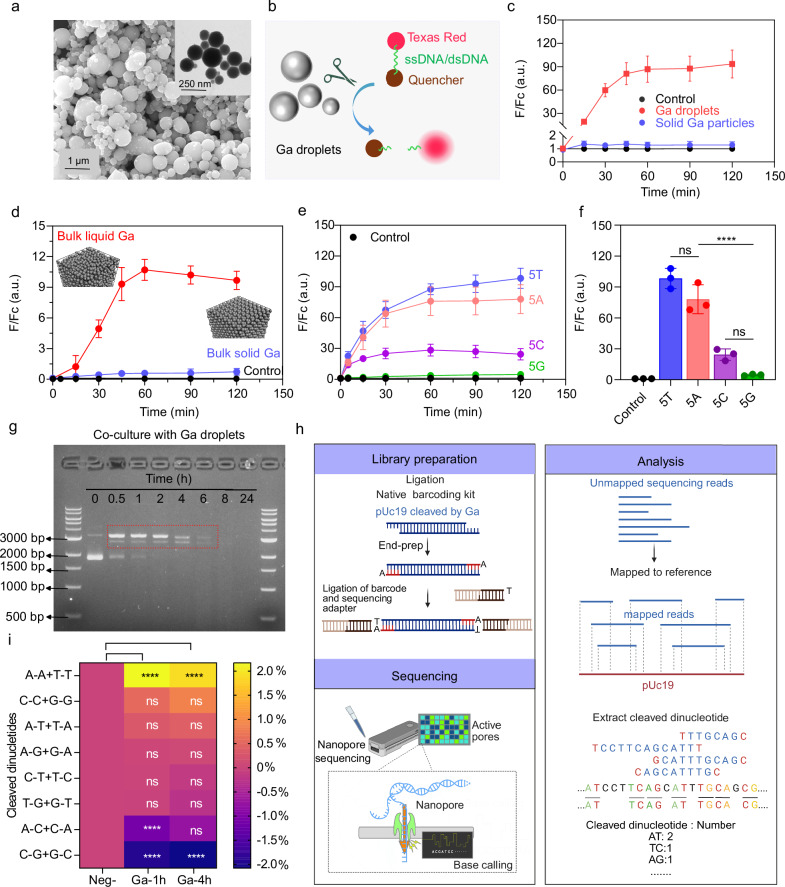
DNA cleavage activity and nucleotide preference of Ga droplets. **a** Representative SEM and TEM images of Ga droplets after 30 min of sonication. Each experiment was repeated three times independently with similar results. **b** Schematic of gallium (Ga) droplets cleaving DNA-based fluorescence reporters (Texas Red ‘TR’ linked to a quencher via single-stranded deoxyribonucleic acid (ssDNA) or double-stranded deoxyribonucleic acid (dsDNA). The scissor element is created in BioRender. Liu, L. (2026) https://BioRender.com/8s6hpu0. **c**,** d** Time-dependent fluorescence of TR-ssDNA reporters incubated with (**c**) Ga particles (*n* = 3 independent reactions) and (**d**) bulk Ga (*n* = 4 independent reactions) in either liquid or solid form. **e**,** f** (**e**) Time-course and (**f**) endpoint (120 min) fluorescence of TR-ssDNA reporters with different sequences treated with Ga droplets (P_(5T-5A)_ > 0.05, P_(5A-5C)_ < 0_._0001, P_(5C-5G)_ > 0.05, n = 3 independent reactions). **g** Agarose gel showing progressive cleavage of the pUC19 plasmid (2686 bp) by Ga droplets. The experiment was repeated three times independently with similar results. **h** Schematic of Oxford Nanopore Technologies (ONT) sequencing used for single-molecule cleavage site identification, adapted from “Principle of Nanopore DNA Sequencing” by DataBase Center for Life Science (DBCLS), *CC-BY 4.0* and some elements are created in *BioRender. Liu, L. (2026)* https://BioRender.com/38ph5fk*. (*Method 5*)*. **i** Heatmap of ONT results showing relative changes in cleaved dinucleotide frequencies in pUC 19 treated with Ga for 1 h and 4 h vs untreated control. Adenine (A), thymine (T), cytosine (C), and guanine (G). (*p < 0.05, **p < 0.01, ***p < 0.001, and ****p < 0.0001. a.u. = arbitrary units). Data are presented as means ± SDs. Source data are provided as a Source Data file.

Liquid Ga micro- and nano-droplets (median 320 nm ± 197 nm) were prepared in ethanol (EtOH) using our previously reported process (Fig. 1a; Method 1)^20^. Both scanning electron microscopy (SEM) and transmission electron microscopy (TEM) confirm the successful formation of spherical Ga droplets (Fig. 2a; Method 2). We identified the nuclease-mimicking activity of these Ga droplets by incubating them with a fluorescence-based nuclease reporter consisting of a fluorescent dye and a quencher linked by a short strand of single- or double-stranded DNA (Fig. 2b and Supplementary Table 1). In this commercial reporter, fluorescence is initially quenched when the dye is in close proximity to the quencher (within 5 nucleotides, ~3 nm). Upon DNA cleavage by a nuclease or nuclease mimic, the dye is separated from the quencher, resulting in a measurable fluorescent signal. Our results show that Ga droplets cleave both single-stranded DNA (ssDNA; Fig. 2c) and double-stranded DNA (dsDNA; Supplementary Fig. 1; Method 3), without affecting dye fluorescence (Supplementary Fig. 2). Temperature-dependent kinetic analyses reveal a markedly lowered apparent activation energy, indicating that Ga droplets function as a nuclease-mimicking platform^21^ (Supplementary Discussion 1, Supplementary Table 2, Supplementary Fig. 3 and Supplementary Method 1). This nuclease-mimicking activity is strongly dependent on the liquid state of Ga, as solid Ga, either in nanoparticle form (Fig. 2c) or as bulk material (Fig. 2d), exhibits negligible cleavage efficiency (Supplementary Method 2 and Supplementary Fig. 4). We attribute the observed activity to the high density of free surface electrons and dynamic surface interactions unique to liquid Ga, as previously reported^15–17^.

In addition, we discovered that Ga droplets exhibit nucleotide sequence-specific cleavage. As shown in Fig. 2e, f and Supplementary Fig. 5, reporters with different sequences demonstrate a preference for thymine-rich and adenine-rich motifs (e.g., T-T, A-A) over guanine- and cytosine-rich sequences (e.g., C-C, G-G). This observation was further validated using agarose gel electrophoresis. The results showed progressive cleavage of the pUC19 plasmid by Ga droplets, from the supercoiled form to nicked, linear, and ultimately a smear of fragmented state after 8 hours of incubation (Fig. 2g; Method 4; Supplementary Fig. 6). Notably, repeated banding patterns were observed between 0.5 and 4 h, prompting us to investigate whether sequence-selective cleavage was occurring. Ligation-based Oxford nanopore sequencing of native DNA was performed on pUC19 plasmids treated with Ga for 1 h and 4 h, with untreated pUC19 as a negative control. Following base calling, reads were aligned to the pUC19 reference sequence, and cleavage sites were determined by the terminal position of the aligned reads on the reference^22^ (Fig. 2h, Supplementary Fig. 7,8, Method 5 and Supplementary Discussion 2).

Chi-squared analysis of dinucleotide frequencies at inferred cleavage sites (Supplementary Table 3) confirmed that the Ga-treated group exhibited significantly higher cleavage at T-T and A-A sites, and lower cleavage at A-C + C-A and C-G + G-C sites compared to the control group (Fig. 2i). These results confirm that the T-T and A-A site preference observed in 5-mer oligonucleotides is retained in longer dsDNA substrates, supporting a consistent nucleotide preference in the nuclease-mimicking activity of Ga droplets.

### Mechanism of the nuclease-mimicking activity of Ga droplets

**Fig. 3:**
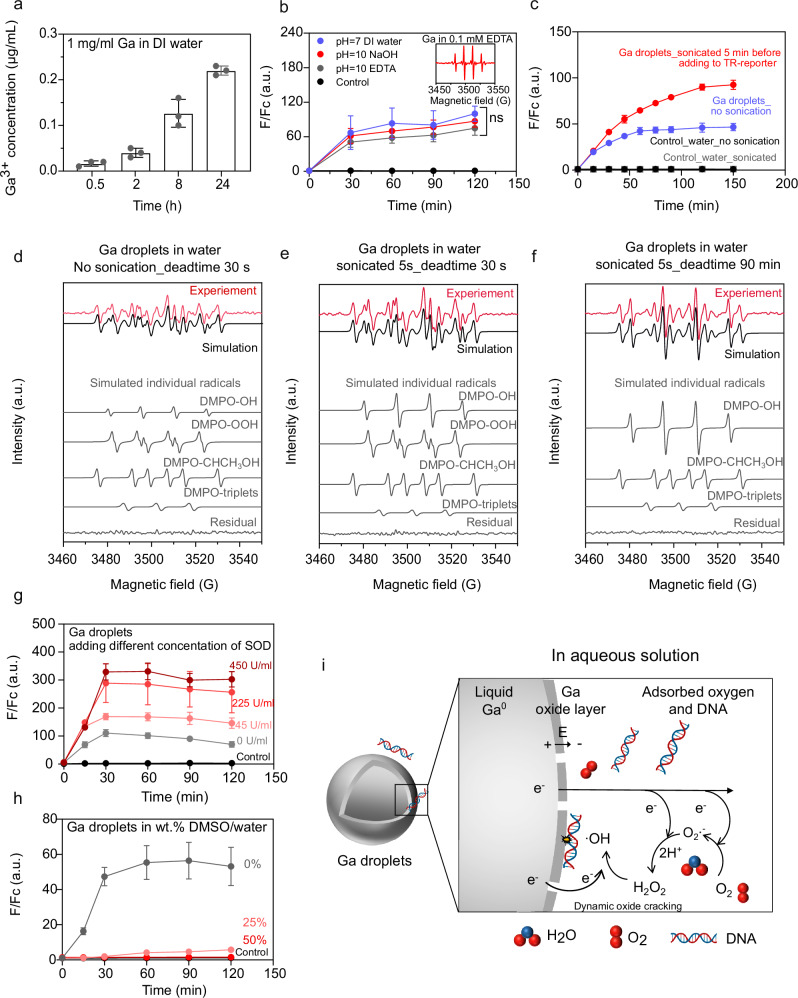
ROS generation of Ga droplets in aqueous solution. **a** Inductively coupled plasma-mass spectrometry (ICP-MS) analysis showing time-dependent release of gallium ion (Ga^3+^) from gallium (Ga) droplets in DI water (n = 3 independent reactions). **b** Fluorescence intensity of TR-ssDNA reporters after treatment with Ga droplets in the presence of ethylenediaminetetraacetic acid (EDTA) under chelating-active conditions, pH = 10 adjusted by sodium hydroxide (NaOH), (*n* = 4 independent reactions). Inset figure: EPR spectra of Ga droplets in 0.1 mM EDTA. (a.u. = arbitrary units). **c** Comparison of the nuclease-mimicking activity of Ga droplets with and without sonication before incubation with the ssDNA TR-reporter. **d**–**f** EPR spectra of Ga droplets in water without sonication with spin-trapping agent 5,5-Dimethyl-1-pyrroline N-oxide (DMPO, n = 3 independent reactions) (**d**), and after brief sonication (5 s), measured following different delay times (30 s (**e**) and 90 min (**f**); dead time defined as the interval between sample preparation, sealing, equipment setting, and EPR data acquisition). Corresponding simulated spectra are shown for comparison (see Supplementary Method 4). **g** Effect of superoxide dismutase (SOD), a superoxide scavenger, on the nuclease-mimicking activity of Ga droplets, evaluated using the TR-reporter. (*n* = 4 independent reactions). **h** Effect of hydroxyl radical ( ∙ OH) quenchers, dimethyl sulfoxide (DMSO) on the nuclease-mimicking activity of Ga droplets, using the TR-reporter (*n* = 4 independent reactions). **i** Schematic illustration of the mechanism for ∙OH generation from Ga droplets in aqueous solution with oxygen (O_2_). The middle product includes hydrogen peroxide (H_2_O_2_), superoxide (O_2_ ∙ ^-^). Abbreviation in the figure: Metallic Ga (Ga^0^), electrical field (E), electron (e^-^). The scissor element is created in BioRender. Liu, L. (2026) https://BioRender.com/8s6hpu0. Data are presented as means ± SDs. Source data are provided as a Source Data file.

The system was primarily composed of metallic Ga (Ga^0^) and DNA. Although Ga^3+^ ions (Fig. 3a; Method 6) and Ga oxides can coexist under these conditions^23^, rigorous control experiments showed that neither species contributed significantly to the observed activity. Specifically, chelating Ga^3+^ with ethylenediaminetetraacetic acid (EDTA) did not significantly alter DNA cleavage efficiency (Fig. 3b), and Ga^3+^ alone showed no cleavage activity (Supplementary Fig. 9a). Similarly, Ga oxides were also inactive when tested independently for DNA cleavage (Supplementary Fig. 9b, c, 10 and Supplementary Method 3). These results confirm that Ga^0^ is the central species driving DNA cleavage, prompting further investigation into the interfacial reactions between Ga^0^ and the aqueous environment. To elucidate how Ga^0^ mediates DNA cleavage, we investigated whether Ga^0^ -associated reactive oxygen species (ROS) generated in aqueous solution^9,24^ contribute to this process. Electron paramagnetic resonance (EPR) spectroscopy and signal simulations (Method 7 and Supplementary Method 4) were performed to detect these intermediates. To clarify the role of sonication, we compared the cleavage intensity and EPR signals of Ga droplets transferred to deionized (DI) water with and without sonication (Fig. 3c–e). The results show that Ga droplets can cleave DNA and generate ROS without sonication. However, brief sonication enhanced this activity by improving droplet dispersion, disrupting the surface oxide layer, and increasing interfacial exposure, as evidenced by the intensified EPR signals and increased cleavage efficiency (Supplementary Fig. 11 and Fig. 3c).

The EPR spectra (Fig. 3d, e) revealed a mixture of 5,5-Dimethyl-1-pyrroline N-oxide (DMPO) adducts: DMPO-OH, DMPO-OOH, and DMPO-CH(CH₃)OH adducts, indicating the generation of hydroxyl radical ( ∙ OH)^25^, superoxide (O_2_ ∙ ^−^)^26,27^, and 1-hydroxyethyl radicals (CH(CH_3_)OH ∙ )^28^, respectively. The CHCH_3_OH∙ signal likely stems from trace EtOH^29^ in the Ga stock solution. Due to the short half-life of DMPO-OOH (*t*_1/2_ = 60 s)^30^, this signal disappeared after a 90-min dead time, leaving a clear and prominent ∙OH signal (Fig. 3f). No ∙OH signal was detected in the Ga-free controls (Supplementary Fig. 12a, b), and the 2,2,6,6-tetramethylpiperidine (TEMP) probing ruled out singlet oxygen (Supplementary Fig. 13). These results collectively demonstrate that Ga^0^-water system intrinsically produces ∙OH and O₂∙⁻.

To identify which of these radicals were involved in this cleavage process, we performed a series of scavenger experiments. The addition of EtOH to probe the involvement of CHCH_3_OH∙ had no effect on cleavage (Supplementary Fig. 14), and the O_2_ ∙ ^−^ scavenger superoxide dismutase (SOD) actually increased the signal (Fig. 3g), likely by accelerating the conversion of O_2_ ∙ ^−^ to hydrogen peroxide (H_2_O_2_)^31^ and subsequently into ·OH. Conversely, the ·OH scavenger dimethyl sulfoxide (DMSO), which reacted with ·OH substantially faster than with O_2_ ∙ ^−^^32,33^ markedly suppressed the DNA cleavage (Fig. 3h). Moreover, the direct involvement of ∙OH was further confirmed by the observed decrease of ∙OH signal as the concentration of DNA increased (Supplementary Fig. 15a). Collectively, these results confirm that ·OH radicals are the primary species responsible for the Ga droplet-mediated DNA cleavage, while CHCH_3_OH∙ is negligible and O_2_ ∙ ^−^ is indirect. Finally, the suppression of cleavage under hypoxia (nitrogen-purged) or water-depleted (glycerol environment) conditions confirms that the presence of both oxygen and water is essential for the interfacial generation of the ∙OH radicals, which is required for this nuclease-mimicking activity (Supplementary Fig. 15b–d).

Based on our experimental results and established redox kinetics, we propose that the ∙OH formation (Fig. 3i) is driven by the potent electron-donating capacity of liquid Ga^12,13^. Upon contact with the aqueous interface, electrons from the metallic Ga^0^ core facilitate the stepwise reduction of dissolved oxygen, initiating a radical cascade forming O_2_ ∙ ^−^, H_2_O_2_ and ultimately ∙OH as shown in the EPR result (Fig. 3d–f and Supplementary Table 4). Concurrently, the Ga droplets undergo gradual oxidation to GaOOH (Supplementary Discussion 3).

We identify two primary pathways that facilitate the flux of electrons from the liquid Ga^0^ core to the aqueous interface. The first one can be described as quantum tunneling across the native oxide. In this case, initially, the oxide layer formed on Ga droplets in EtOH is sufficiently thin to allow quantum tunneling^34,35^. Within this oxide layer, the near-surface electron states below the Fermi level establish an internal electric field that facilitates electron transport^36,37^. Upon transfer to water, the Ga^0^ core donates electrons through this thin shell to dissolved oxygen in the water, initiating ROS generation. However, as the oxide layer thickens beyond a few nanometers, the electric field weakens, rendering the process self-limiting and eventually blocking further electron transport^37,38^. To validate this tunneling-dependent activity, we compared Ga droplets with varying degrees of oxide thicknesses. We first examined samples stored in EtOH for one day *vs* one month. X-ray photoelectron spectroscopy (XPS) analysis revealed that while Ga droplets aged for one month retain a dominant metallic Ga^0^ core, they exhibited a significantly higher Ga^3+^ contribution compared to the one-day sample (Supplementary Fig. 16). This chemical shift was also concurrent with SEM observations of less rounded morphologies, indicating thickened oxide layers. Functionally, the DNA cleavage activity of the one-month-old droplets remained detectable but was reduced compared to freshly prepared droplets (Supplementary Fig. 17). This demonstrates that while a thickened oxide layer substantially suppresses electron flux, it does not achieve total passivation. To further isolate the effect of oxide thickness, we prepared Ga droplets under nitrogen to minimize the oxidation (denoted as Ga droplets with thinner oxide layers), or under air bubbling to promote it (denoted as Ga droplets with thicker oxide layers). Upon transferring to aqueous DNA solutions, the droplets with thinner oxide layers exhibited significantly higher radical generation and cleavage efficiency than those with thicker ones (Supplementary Fig. 18). Collectively, these results provide evidence that the initial nuclease activity is likely mediated by electron tunneling across the oxide interface, a process that is progressively hindered as the dielectric barrier thickens.

The second alternative or concurrent pathway for the flux of electrons can be due to the mechanical surface renewal via oxide cracking. This involves the mechanical breakage of the oxide shell, which enables direct contact between the metallic Ga^0^ core and the aqueous environment. In water, the oxidation of Ga induces significant volumetric changes^39,40^ (Supplementary Discussion 3 and Supplementary Figs. 19–21) that generate interfacial strain^41^, leading to the cracking of the surface oxide layer^42^. This mechanical rupture continuously exposes fresh, unpassivated Ga^0^ surfaces to the surrounding solution. By maintaining a direct Ga^0^-water interface, this process facilitates unhindered electron transfer and ∙OH production. While tunneling is critical during the initial contact phase, this surface renewal mechanism ensures that reactive metallic sites remain accessible even as oxidation proceeds.

**Fig. 4:**
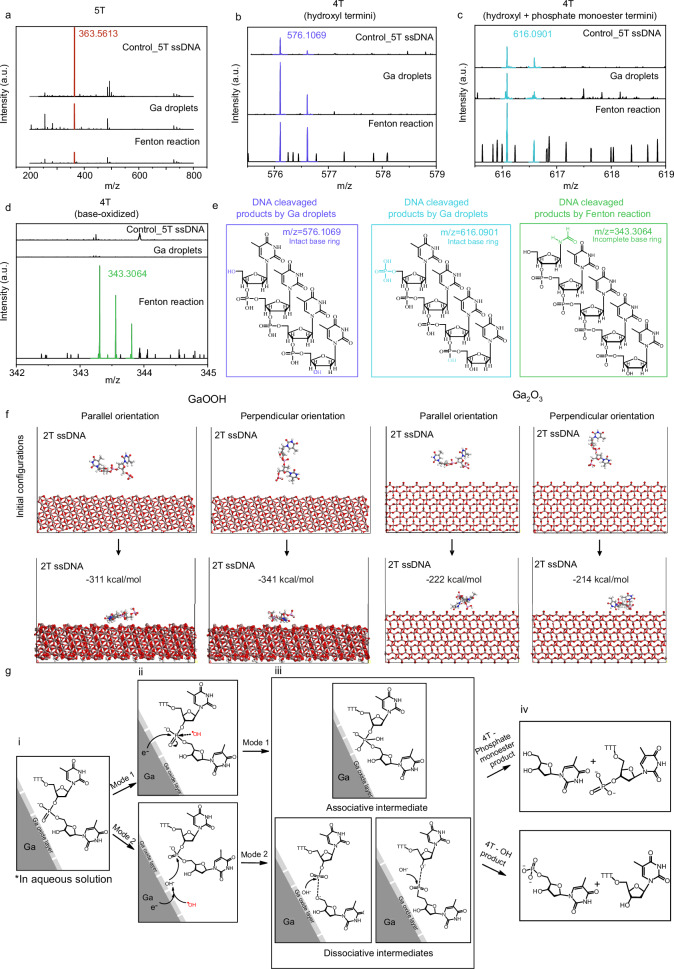
Mechanism of Ga droplets’ nuclease-mimicking effect: ROS-assisted hydrolytic cleavage activity. **a**–**d** MS analysis of five thymine (T) single-stranded deoxyribonucleic acid (ssDNA) fragments after Ga droplets or Fenton reaction treatment: (**a**) full spectrum showing fragmentation pattern with 5 T ssDNA peaks; (**b**) zoom-in of 4 T fragments with two hydroxyl termini; (**c**) zoom-in of 4 T fragment with hydroxyl and phosphate monoester termini; (**d**) zoom-in of 4 T fragment with oxidized base. In control samples, some hydrolytic-like peaks were observed due to in-source decay and high-voltage ionization^52,53^. The analysis of the 3T-to-1T fragments is provided in Supplementary Discussion 4. **e** 4 T fragment structures corresponding to m/z values in (**b**–**d**). **f** MD simulations of 2T-ssDNA interacting with the Ga oxide layer: Ga oxide (Ga_2_O_3_) and Ga oxyhydroxide (GaOOH), using two initial configurations: ssDNA perpendicular or parallel to the surface; The water molecules were made invisible for clarity of visualization. **g** Proposed mechanism of Ga-induced DNA cleavage. Source data are provided as a Source Data file.

To elucidate the DNA cleavage mechanism of Ga droplets, we compared their fragmentation patterns with those generated by a classical ∙OH-based Fenton reaction using high-resolution mass spectrometry (MS)^43^ (Fig. 4a–e, Method 8 and Supplementary Method 5). Relative to the control, both Ga-treated and Fenton-treated samples showed a marked decrease in the intact 5 T ssDNA ion (*m/z* 363.56, z = −4), accompanied by the appearance of 4 T fragments at *m/z* 576.10 and 616.09 (z = −2) (Supplementary Figs. 22, 23 and Supplementary Tables 5, 6). These ions correspond to two specific cleavage products: a 4 T fragment bearing two hydroxyl termini and a second fragment containing one hydroxyl and one phosphate monoester terminus (Supplementary Fig. 24, Supplementary Tables 7–10 and Supplementary Discussion 4).

To assess the involvement of oxidative pathways, expected fragments arising from typical metallonuclease mechanisms, including hydrogen abstraction and nucleobase oxidation, were predicted and used for targeted *m/z* screening (Supplementary Fig. 25 and Supplementary Tables 11, 12). Oxidative base-modified fragments were detected in the Fenton reaction, whereas no such species were observed in Ga-treated samples (Supplementary Fig. 26). This agrees with established Fenton chemistry, in which ∙OH radicals preferentially attack nucleobases, causing secondary cleavage of the phosphodiester backbone^44,45^. In contrast, Ga droplets exclusively generated phosphodiester bond-derived cleavage products, with no detectable nucleobase or sugar-ring oxidation, showing similar hydrolytic nuclease activity. These results further provide evidence that Ga droplets cleave DNA via an ∙OH-assisted hydrolytic mechanism targeting the phosphodiester bond, rather than through oxidative degradation characteristics of classical Fenton systems.

To further elucidate the role of the Ga interface, we employed multiscale computational approaches to examine the individual contributions of the species present in the system: metallic Ga^0^, Ga oxide layer, and free Ga^3+^. Classical molecular dynamics (MD) simulations were employed to investigate the adsorption behavior of ssDNA on Ga oxide surfaces (Supplementary Table 13, Supplementary Discussion 5 and Supplementary Method 6). We evaluated two representative orientations: ssDNA aligned either parallel or perpendicular to the Ga oxide surface (Fig. 4f). The simulations indicate that, given the significant size disparity between the Ga droplets (average diameter ≈ 330 nm) and the ssDNA ( ~ 3 nm in length), the DNA molecules preferentially adopt a flat adsorption configuration. This lying-down orientation on the relatively planar droplet surface facilitates simultaneous interfacial interactions across multiple molecular moieties of the DNA strand.

Density functional theory (DFT) calculations were employed to characterize the adsorption affinity of ssDNA toward both Ga^0^ atoms and Ga^3+^ ions (Supplementary Discussion 6). The simulations show that Ga^0^ can adsorb nucleobases, the sugar ring, and the phosphodiester bond (Supplementary Fig. 27a), mirroring the multi-site, whole-molecule adsorption observed in our MD simulations. Notably, the phosphate groups exhibit a higher adsorption affinity for Ga^0^ than the nucleobase nitrogen sites, likely due to enhanced electrostatic and van der Waals interactions. In the presence of ∙OH, the cleavage of this phosphate diester bond is energetically favorable (Supplementary Fig. 27b, c, Supplementary Discussion 6 and Supplementary Method 7). In contrast, free Ga^3+^ -phosphate interactions lead to the formation of thermodynamically stable Ga-O bonds. These stable bonds effectively ‘lock’ the structure and do not promote cleavage, even in the presence of ∙OH, thereby explaining why Ga^3+^ does not contribute to the system’s nuclease-like activity (Supplementary Fig. 28). This finding is fundamentally consistent with our experimental observation in (Fig. 3b and Supplementary Fig. 9a).

Together, these computational and experimental results indicate a dual-pathway mechanism for DNA cleavage: (1) Oxide-mediated adsorption and tunneling: Ga oxides (Ga_2_O_3_) and Ga oxyhydroxide (GaOOH) act as a scaffold that adsorbs ssDNA, effectively accumulating them at the interfaces. Simultaneously, quantum tunneling across this native oxide layer can facilitate the necessary electron transfer for ∙OH generation, leading to localized DNA cleavage. (2) Direct metallic contact: At sites of oxide rupture, Ga^0^ is directly accessible to oxygen, water and ssDNA, enabling localized electron transfer and efficient ∙OH formation to mediate the subsequent cleavage of the phosphodiester bond.

Integrating the results, we propose that Ga-induced hydrolysis of DNA occurs via a synergistic multi-step mechanism (Fig. 4g): (1) Interfacial Sequestration (Fig. 4g, i) in which ssDNA is adsorbed onto the Ga droplet surface while Ga droplets generate ∙OH radicals through the previously discussed electron-transfer pathways. (2) Radical-Mediated Activation (Fig. 4g, ii–iii) in which the ∙OH participates in the reaction via two proposed initiation modes as follows: (a) Phosphodiester groups adsorbed on the Ga droplets surface, carrying an increased negative charge that enhances their attraction to ∙OH and facilitates direct radical attacks on the phosphorus center (Fig. 4g, ii). (b) Alternatively, ∙OH near the Ga droplets surface receives electrons from the metallic core to OH⁻, which activates the *P* = O bond and induces nucleophilic attack by hydroxide ions (Fig. 4g, ii). DFT results suggest that Ga preferentially associates with two oxygen atoms that are not directly connected to the sugar rings, indicating the hydrolysis reacts via forming associative nucleophilic intermediates (Fig. 4g, iii), possibly through an associative nucleophilic substitution followed by dissociative elimination, or a concerted associative–dissociative pathway^46^. The synergistic effect of Lewis’s acid activation by Ga and nucleophile attack by Ga and nucleophile activation by ∙OH or OH⁻, underpins the ∙OH assisted hydrolytic cleavage mechanism^46,47^. The details of a possible mechanism for Ga droplets’ preference to T-T and A-A are provided in Supplementary Discussion 7, Supplementary Fig. 29 and Supplementary Method 8. (3) Hydrolytic Product Formation (Fig. 4g, iv): The reaction concludes with the release of distinct 4 T fragments. Mode 1 primarily yields a 4T-phosphate monoester product, preserving the phosphate group on one terminus and mode 2 results in a 4T-OH product, where both cleavage fragments possess hydroxyl termini.

Together, Ga droplets implement DNA cleavage through a combination of adsorption preference and ∙OH-assisted phosphodiester hydrolysis. This mechanism differs fundamentally from conventional ROS-induced cleavage, where ∙OH typically abstracts hydrogen from the sugar moiety or oxidizes nucleobases^44,45^, resulting in random fragmentation^5^. Instead, Ga droplets’ surface-localized activity results in a T-T and A-A cleavage preference while generating fragments with intact phosphate or ribose ring termini^5^.

### Programmable and controllable nuclease-mimicking properties of Ga

**Fig. 5:**
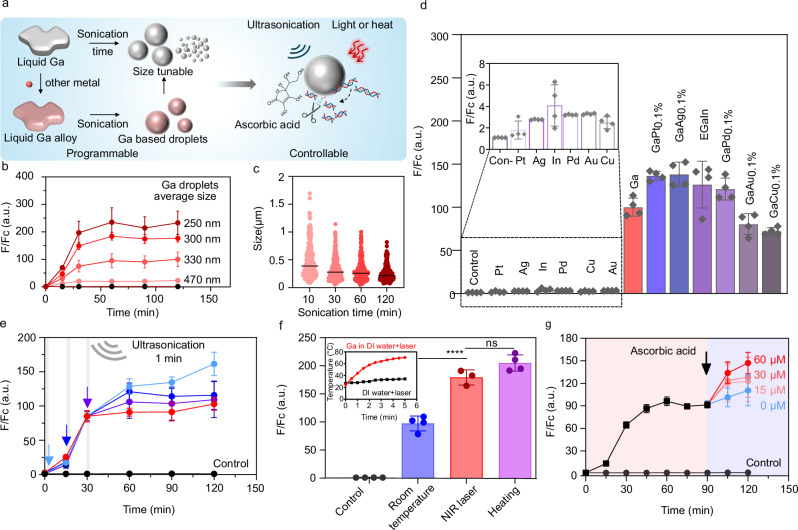
Programmable and externally controllable nuclease-mimicking activity of Ga droplets. **a** Schematic illustration of the programmable and controllable properties of gallium (Ga) droplets. Some graphic elements are created in BioRender. Liu, L. (2026) https://BioRender.com/8s6hpu0. **b** DNA cleavage efficiency of Ga droplets with varying sizes (*n* = 4 independent reactions). **c** Average size of Ga droplets tuned by sonication time (particle numbers = 317). **d** DNA cleavage activity of other pure metal nanoparticles and Ga-based alloy droplets (*n* = 4 independent reactions). **e** Enhancement of Ga-mediated DNA cleavage by 1 min bath sonication applied at different time points during incubation (*n* = 4 independent reactions). **f** Enhancement of Ga nuclease-mimicking activity by 808 nm near-infrared (NIR) laser irradiation and heat treatment for 5 min at 15 min point (P_(NIR-Heating)_ > 0.05, P_(NIR-Heating)_ < 0.0001, *n* = 4 independent reactions). The inset shows the temperature profile of laser exposure, with heat treatment mimicking the same thermal change. **g** DNA cleavage activity of Ga droplets following the addition of ascorbic acid at the 90 min time point (*n* = 4 independent reactions). (*p* < 0.05 is considered as statistically significant. *p < 0.05, **p < 0.01, ***p < 0.001, and ****p < 0.0001. a.u. = arbitrary units). Data are presented as means ± SDs. Source data are provided as a Source Data file.

Building on the intrinsic DNA-cleaving activity of Ga droplets, we further demonstrated their potential as programmable and externally controllable artificial nucleases. Their cleavage efficiency can be systematically tuned by adjusting synthesis conditions and applying external physical stimuli (Fig. 5a).

One effective strategy to modulate this activity is controlling droplet size via sonication. Extending sonication time from 10 to 120 min reduced droplet diameter from ~ 470 nm to ~ 250 nm (Fig. 5b, c and Supplementary Fig. 30), leading to enhanced TR-ssDNA reporter cleavage. This size-dependent effect is attributed to the increased surface area-to-volume ratio. In addition, given liquid Ga’s ability to readily dissolve a broad range of metals^15–18^, we investigated whether dissolving other metals into Ga could modulate its nuclease-mimicking activity. We examined several commercial metallic micro/nano particles such as gold (Au), copper (Cu), platinum (Pt), indium (In) within the size range of Ga droplets, none of which exhibited intrinsic DNA cleaving capability (Fig. 5d). In the Ga-based alloy droplets (Supplementary Method 9 and Supplementary Fig. 31), the addition of these metals led to either enhanced activity (GaAg, GaPt, EGaIn, and GaPd) or reduced activity (GaAu and GaCu) compared to pure Ga (Fig. 5d). Since Ag, Pt, In, and Pd are known as redox catalysts.^14^, this enhancement may result from the augmented electron-transfer interactions at the alloy interface that promote radical generation or DNA cleavage. Although the precise mechanism requires further investigation, these findings position Ga droplets as a versatile platform for designing tunable artificial nucleases with adjustable cleavage efficiency and potential sequence selectivity.

Beyond programmable synthesis, Ga droplets’ activity can be modulated by external stimuli. Brief bath sonication during DNA incubation significantly enhanced cleavage efficiency without damaging the DNA substrate (Fig. 5e, Supplementary Fig. 32 and Supplementary Method 10). This likely results from surface renewal, as sonication disrupts the passivating oxide layer^48^, exposing Ga^0^ that promotes ∙OH radical formation. The enhanced activity underscores Ga droplets’ potential as a mechanically responsive artificial nuclease. Similarly, exposing Ga droplets to 808 nm near-infrared (NIR) laser irradiation nearly doubled DNA cleavage efficiency (Fig. 5f). Since the laser also elevated the solution temperature^9^, a matched heat treatment was conducted to assess whether the NIR enhancement was thermally driven. The comparable results (Fig. 5f) suggested that temperature is the main factor, likely by accelerating the oxide shell lack of integrity, reaction kinetics and also ∙OH generation. Furthermore, when the reaction plateaued likely due to the accumulation of a stable oxide layer that passivates the Ga droplets surface, the addition of ascorbic acid, a known reducing agent, reactivated the cleavage process. This was evidenced by a renewed fluorescence intensity (Fig. 5g), suggesting that reduction of the oxidized shell back to Ga^0^ results in better interfacial access, enhanced ∙OH generation and DNA cleavage. These findings highlight the reversible and stimuli-responsive nature of Ga droplets’ activity, offering a valuable strategy for on-demand reactivation by further disrupting the integrity of the natural oxides.

Together, these results demonstrate that Ga droplets function as a tunable and reactivatable artificial nuclease, whose activity can be regulated through both synthesis design and by external mechanical, photothermal, and chemical inputs. Their ability to maintain their function under elevated temperatures further highlights their robustness in conditions where natural enzymes fail. This combination of programmability, responsiveness, and thermal stability positions Ga droplets as a promising platform for controlled and on-demand DNA cleavage in diverse biochemical environments.

### Nuclease-mimicking activity of Ga in physiological fluids

**Fig. 6:**
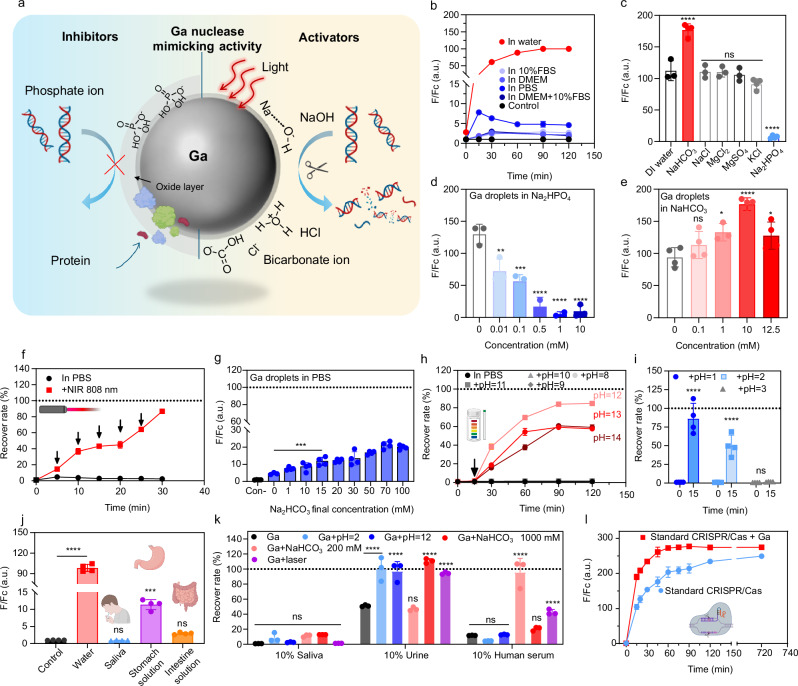
Modulation of Ga droplets’ nuclease-mimicking activity in typical biological environments. **a** Schematic illustrating the inhibition and recovery of Ga droplets-mediated deoxyribonucleic acid (DNA) cleavage under biologically relevant conditions. **b** Cleavage of TR-ssDNA reporters by Ga droplets in various biological media, including DI water, DMEM, DMEM with 10% FBS, pure FBS, and PBS. **c** Effects of intracellularly ions on Ga nuclease-mimicking activity at physiological concentrations (P_(DIwater-NaHCO3)_ < 0.0001_,_ P_(DIwater-Na2HPO4)_ < 0.0001, P_(DIwater-NaCl,MgCl2,MgSO4,KCl)_ > 0.05, *n* = 3 independent reactions). **d** Inhibition of Ga-mediated DNA cleavage by Na₂HPO₄ solution (P_(0-0.01)_ = 0.0028_,_ P_(0-0.1)_ = 0.0002, P_(0-0.5)_ < 0.0001, P_(0-1)_ < 0.0001, P_(0-10)_ < 0.0001,* n* = 3 independent reactions). **e** Concentration-dependent enhancement of Ga nuclease-mimicking activity by NaHCO₃ (P_(0-0.1)_ ≥ 0.05_,_ P_(0-1)_ = 0.0110, P_(0-10)_ < 0.0001, P_(0-12.5)_ = 0.0201, *n* = 4 independent reactions). **f**–**i** Recovery of Ga nuclease-mimicking activity in PBS by (**f**) 808 nm NIR laser irradiation (*n* = 3 independent reactions), (**g**) adding NaHCO_3_ (P_(0-15)_ = 0.0004, *n* = 4 independent reactions), (**h**) adding NaOH solution (final pH = 11, *n* = 4 independent reactions), (**i**) adding HCl solution (final pH = 3, P_(pH=1, 0-15)_ < 0.0001, P_(pH=2, 0-15)_ < 0.0001, P_(pH=3, 0-15)_ > 0.05, *n* = 4 independent reactions) (**g**). **j**–**I** TR-ssDNA reporters cleaved by Ga droplets in different biologically relevant environments: (**j**) in 100% saliva, simulated gastric and intestinal fluids (P_(Control-Water)_ < 0.0001_,_ P_(Control-Saliva)_ > 0.05, P_(Control-Stomach solution)_ < 0.0002, P_(Control-Intestine solution)_ > 0.05, n = 4 independent reactions), (**k**) in 10% saliva, urine, human serum, with different additives (10% saliva: P_(Ga-other groups)_ > 0.05; 10% urine: P_(Ga-Ga+pH=2)_ < 0.0001, P_(Ga-Ga+pH=12)_ < 0.0001, P_(Ga-Ga+Na2HCO3 20 mM)_ < 0.0001, P_(Ga-Ga+Na2HCO3 100 mM)_ = 0.0413, P_(Ga-Ga+laser)_ < 0.0001, 10% human serum: P_(Ga-Ga+pH=2)_ > 0.05, P_(Ga-Ga+pH=12)_ > 0.05, P_(Ga-Ga+Na2HCO3 20 mM)_ < 0.0001, P_(Ga-Ga+Na2HCO3 100 mM)_ > 0.05, P_(Ga-Ga+laser)_ < 0.0001, n = 3 independent reactions), (**l**) in the CRISPR (clustered regularly interspaced short palindromic repeats)/Cas9 (CRISPR-associated protein 9) (n = 3 independent reactions). (*p* < 0.05 is considered as statistically significant. *p < 0.05, **p < 0.01, ***p < 0.001, and ****p < 0.0001. a.u = arbitrary units). Some graphical elements in Fig. 6a and f-I were created *in BioRender. Liu, L. (2026)* https://BioRender.com/407xx9q. Data are presented as means ± SDs. Source data are provided as a Source Data file.

To evaluate the potential of Ga droplets for biomedical applications, we assessed their DNA-cleaving activities across various physiological media. Like natural nucleases, whose activity can be regulated by cofactors, inhibitors, and activators, Ga droplets’ cleavage behavior can also be adjusted by biochemical components as inhibitors and by external stimuli as activators (Fig. 6a).

We first observed that Ga-induced DNA cleavage was significantly inhibited in phosphate-buffered saline (PBS), Dulbecco’s modified eagle medium (DMEM), 10% fetal bovine serum (FBS), and DMEM with 10% FBS (Fig. 6b). The inhibition in FBS is likely due to protein corona formation^49^, where adsorbed proteins passivated the Ga surface and hindered its enzymatic function^50^. To identify the inhibitory components in PBS and DMEM, we examined individual ions (Supplementary Table 14 and Supplementary Fig. 33) and found that phosphate (Na₂HPO₄, ≥ 1 mM) significantly inhibited DNA cleavage, while bicarbonate ions (NaHCO₃, ~ 10 mM) enhanced it (Fig. 6c). These effects were concentration-dependent (Fig. 6d, e), resembling the modulatory roles of inhibitors and activators in natural enzyme systems.

To restore cleavage activity under inhibitory conditions, we used PBS as a model and evaluated several reactivation strategies based on prior results (Fig. 5e–g). Neither ascorbic acid nor sonication recovered the activity (Supplementary Fig. 34), but 808 nm laser irradiation successfully restored DNA cleavage (Fig. 6f), possibly by cracking the oxides on the surface of droplets and allowing better access to Ga^0^. This effect was not replicated by heat treatment, suggesting that high-energy intensity from the laser is required (Supplementary Fig. 35). NaHCO₃ treatment also reinstated activity (Fig. 6g), likely through the assistance for better removal of the oxide shells, mild alkalinity ( ~ 8.4 at 100 mM), combined with competitive adsorption with phosphate. To isolate the role of pH, we tested acidic and basic conditions and found that both extremes (pH ≤ 2 or ≥ 12) could restore up to 80% of cleavage activity (Fig. 6h, i and Supplementary Fig. 36), confirming pH as a key factor modulator and likely through the removal of the oxides and enhanced access to the active Ga^0^ interface.

We next assessed the Ga droplets’ activity in clinically relevant biofluids. DNA cleavage was completely suppressed in saliva and intestinal fluid simulants but remained effective in gastric fluid simulants (pH = 2, that promotes the removal of the oxides and access to Ga^0^ active surface), indicating possible application in orally delivered systems that activate in the stomach (Fig. 6j). In urine and human serum, Ga-induced DNA cleavage was inhibited, due to assistance to the formation of the oxide shell, but could be partially recovered with specific external stimuli (Fig. 6k and Supplementary Figs. 37, 38) that helped in cracking the shells and access to Ga^0^. These findings support Ga droplets’ potential for extracellular DNA degradation and biosensing applications in complex biological matrices. Furthermore, CRISPR/Cas (Fig. 6I and Supplementary Method 11) and commercial nuclease buffers (Supplementary Table 15) are compatible with Ga droplets’ activity, showing strong potential to integrate Ga droplets with nucleases through rationally designed cooperative systems to enhance overall cleavage efficiency.

Furthermore, systematic comparisons in both DI water and PBS against commercial nucleases (S1 nuclease, Benzonase, and DNase I, Supplementary Fig. 39, Supplementary Table 16 and Supplementary Method 12) established Ga droplets as a distinct and efficient nuclease-mimic platform. In DI water, Ga droplets (0.1 mg mL^−1^, ~ 330 nm) achieved approximately one-third of the cleavage efficiency of S1 nuclease, while size reduction to ~ 250 nm enabled cleavage efficiencies comparable to S1 and exceeding those of Benzonase and DNase I. In PBS, phosphate ions suppressed the activity of both S1 nuclease and Ga droplets, whereas Benzonase retained partial activity; critically, unlike protein enzymes, Ga droplets can be reactivated and even outperform commercial nucleases upon external stimulation, exemplified here by 808 nm laser irradiation. In dsDNA reporter assays, S1 nuclease showed no activity and DNase I failed to cleave short dsDNA fragments ( < 5-6 bp), consistent with its intrinsic substrate and length constraints^51^, whereas Ga droplets maintained endonuclease-like activity across ssDNA and dsDNA substrates. Beyond their tunable activity (Fig. 5a), wide temperature ranges (Fig. 5f and Supplementary Fig. 3), high ionic strength (Supplementary Fig. 33), and EDTA-containing environments (Fig. 3b), Ga droplets achieve comparable cleavage efficiencies at ~ 1% of the cost. Importantly, in contrast to many radical-generating artificial nucleases that cause extensive base oxidation and sugar-ring degradation^4,44^, Ga droplets predominantly yield hydrolysis-like cleavage products, preserving nucleobase and sugar-ring integrity while selectively cleaving the phosphodiester backbone.

Together, the sensitivity of Ga droplets to inhibitors such as phosphate, together with their reactivation by physical and chemical triggers, including pH adjustment, bicarbonate addition, and light irradiation via modulation of surface oxide integrity, highlights a unique, switchable, and programmable nuclease-mimic behavior.

## Discussion

In this study, we uncovered the intrinsic nuclease-mimicking properties of liquid-state Ga droplets, which display selective DNA cleavage activity with a strong preference for T- and A-rich sequences over C and G. Through fluorescence reporter assays, ONT sequencing, DFT and MD simulations, we showed that Ga-mediated DNA cleavage was governed by a dual mechanism of phosphate diester bond preferred adsorption and ∙OH-assisted phosphodiester hydrolysis via nucleophilic substitution. This synergy was shown to enable site preference while preserving intact termini, thereby minimizing the non-specific oxidative damage that frequently limits traditional ROS-based natural and artificial nucleases.

Furthermore, compared with other ligands- and cofactors- dependent artificial metallonuclease, liquid Ga droplets uniquely utilize their native oxide layer for substrate adsorption and the electron-rich Ga^0^ core as the active center, forming a scaffold-free, metal-only nuclease-mimic platform. Their activity is tunable via synthetic strategies (e.g., droplet size, alloy composition, etc.) and dynamically controlled by external stimuli such as pH, light, sonication, and chemical additives. Despite suppression in protein- and phosphate-rich physiological fluids, we demonstrate that Ga activity can be reversibly reactivated, offering potential for stimulus-triggered DNA cleavage in complex biological environments for controlled activity release.

To our knowledge, this is the first artificial nuclease system that simultaneously exhibits nucleotide adsorption with preference and controlled ROS-assisted cleavage, which is a property highly desirable for precision applications. These findings not only deepen our understanding of how LMs interact with DNA but also position Ga droplets as a promising and programmable alternative to enzyme-based nucleases.

Beyond mechanistic insights, our work opens directions in therapeutic design by which the ability to target T-rich regions introduces a modality for sequence-biased nucleic acid cleavage, with potential applications in DNA degradation, gene editing, and antiviral therapies such as targeting T-rich genomic elements or T-rich viral and bacterial genomes (e.g., Mycoplasma, parvoviruses); and biomimicry where this scaffold- and peptide-free, metal-only, liquid-phase Ga system presents a versatile and tunable platform for developing next-generation artificial enzymes and advancing minimalist, bioinspired enzyme-mimics in abiotic systems, thereby broadening the design space for artificial enzymes beyond traditional coordination complexes.

## Methods

### Chemicals and materials

Gallium (Ga) and indium (beads, 99.99%) were purchased from RotoMetals Inc. (USA). Silver (5–8 μm powder, ≥ 99.9%), platinum (shot, ≤ 3 mm, ≥ 99.9%), palladium (shot, ≤ 3 mm, ≥ 99.9%), gold (5–8 μm powder, ≥ 99.9%), copper (wire, ≥ 99.9%), indium particles ( < 150 nm, > 99.99%), silver particles ( < 150 nm, > 99%), platinum particles ( < 50 nm, > 99%), palladium particles ( < 25 nm, > 99.5%), gold particles ( < 100 nm, > 99.9%), copper particles (40–60 nm, > 99.5%), PBS (pH 7.4), DMSO, glycerol, ascorbic acid, Ga nitrate, Ga oxyhydroxide, disodium ethylenediaminetetraacetate, sodium hydroxide, hydrochloric acid, sodium chloride, potassium chloride, sodium bicarbonate, magnesium chloride, magnesium sulphate, disodium hydrogen phosphate, potassium phosphate monobasic, Sodium dodecyl sulfate (SDS), DMEM, and DMPO, TEMP, SOD, TAE buffer, S1 nuclease, Benzonase, DNase I, H_2_O_2_, methylene blue, ammonium iron(III) sulfate dodecahydrate (NH_4_Fe(SO_4_)_2_ · 12H_2_O) were purchased from Sigma-Aldrich (USA). FBS, pUC19 plasmid, human serum, agarose, DNase/RNase-free water, and 5 T oligonucleotide were obtained from Thermo Fisher Scientific (USA). Saliva and urine samples were procured from healthy adult volunteers following informed consent and institutional guidelines. Hydrochloric acid (33 wt% in water) was obtained from Chem-Supply Pty Ltd. (Australia). EnGen® Lba Cas12a (Cpf1) protein, ENB2.1 buffer, and 1 kb DNA ladder were purchased from New England Biolabs (USA). HydraGreen™ Safe DNA Dye (20,000 × in water) and 10× Orange Loading Dye were obtained from HydraGene (China) and LicorBio (China), respectively. DNA-based reporters were obtained from Integrated DNA Technologies (IDT, USA) and Sangon Biotech Ltd. (China); please refer to Supplementary Table 1 for more details.

### Ga droplets preparation (Method 1)

Half micro-sized Ga droplets were synthesized using a typical sonication method as described in our previous work^20^. Ga (1 g) was added to 10 mL of EtOH (100%, undenatured, ChemSupply, Australia) in a round-bottom vial. The tube was then placed in an oven at 50 °C for 5 min to melt Ga into its liquid state. The liquid Ga was subsequently sonicated using a probe sonicator (SONICS VCX 750, Amp: 40%) in burst mode (on/ off: 6/4 s). Sonication was performed for various durations, corresponding to total “on-time” periods of 0.5, 1, 1.5, and 2 h. The resulting Ga droplets were stored in EtOH for further use. Besides, liquid bulk Ga for the experiment in Fig. 2d was prepared as described in Supplementary Method 2. Ga alloy droplets for the experiment in Fig. 5d were prepared following the procedure in Supplementary Method 9. In addition, Ga exhibits a pronounced supercooling effect, allowing it to remain in the liquid state for extended periods below its melting point. Additional details are provided in Supplementary Method 2.

### Characterization of Ga droplets (Method 2)

SEM and Energy-Dispersive X-ray Spectroscopy (EDS) were performed using an analytical SEM system (JEOL JSM-IT 500 HR with EDS, Bruker Silicon). The SEM samples were obtained by drop-casting 10 μL of Ga droplet suspensions (1 mg/ml) onto silicon wafers. TEM analyses were conducted using a JEOL JEM-F200, operating at 200 kV with a cold field-emission gun. The instrument was equipped with an annular dark-field detector and a JEOL windowless 100 mm^2^ silicon drift x-ray detector. XPS measurements were carried out using a SPECS PHOIBOS 100 instrument with a monochromatic Al Kα source to characterize surface chemical composition.

### Reporter experiment (Method 3)

The Ga droplets in EtOH underwent solvent exchange with DI water via centrifugation, then were vortexed or bath-sonicated for 5 min to ensure dispersion. A mixture of 90 µL of 166 nM reporter and 10 µL of Ga droplets was added to each well of a 96-well plate, with at least three replicates per group. The reaction occurred at room temperature, and fluorescence intensity was measured using a plate reader (CLARIOstar® Plus, BMG LABTECH, USA) at Ex/Em wavelengths: 570/615 nm (Texas Red) and 550/570 nm (Cy3). Details of reporter experiments corresponding to Figs. 5 and 6, including specific experimental settings, are provided in Supplementary Methods 10.

### Agarose gel electrophoresis (Method 4)

Agarose gel electrophoresis was performed as follows, unless otherwise specified. The pUC19 plasmid (5 ng/μL) was incubated with Ga droplets (0.5 mg/mL) in DI water. After different periods of time, Na_2_HPO_4_ was added to a final concentration of 20 mM to stop the reaction and detach the cleaved plasmid from the droplets’ surface, which improved visualization of bands in the gel. Without it, the gel showed some smearing of pUC19 after 0.5 hours of incubation, due to DNA adsorption on the surface of Ga droplets (Supplementary Fig. 4). This behavior is attributed to the strong adsorption affinity of phosphate ions toward Ga oxide/hydroxide surfaces, which may competitively displace DNA adsorption. The details are provided in Supplementary Discussion 7.

After adding Na_2_HPO_4_ for 12 h, the plasmid and Ga droplets were separated by centrifugation. The plasmid samples (15 μL) were mixed with 1.5 μL 10X Orange loading buffer and ran on a 1.5% agarose gel stained with HydraGreen (1:25,000 dilution) in 1X TAE buffer. Electrophoresis was conducted at 100 V for 35 min, and images were captured using a Bio-Rad GelDoc XR System with Imaging Lab software.

### ONT DNA sequencing (Method 5)

ONT DNA sequencing requires a high concentration of pUC19, so both the plasmid and Ga droplets concentrations were proportionally increased. The pUC19 plasmid (100 ng/µL) was co-incubated with Ga droplets (22 mg/mL) in DI water for varying durations. To detach the cleaved plasmid from the droplets’ surface, Na_2_HPO_4_ was added to a final concentration of 50 mM. After 12 hours, the plasmid and droplets were separated by centrifugation. The control sample contained only pUC19, while the positive control was digested with the restriction enzyme HindIII.

Prior to ONT library prep, the samples were bead cleaned to remove excess Na_2_HPO_4._ The samples were cleaned using a 1.2 ×  ratio of AMPureXP beads, washed twice with 80% EtOH, and eluted in 50 μL of nuclease-free water. The samples (1000 ng per sample) were prepared for sequencing using Oxford Nanopore Native Barcoding Kit 96 V14 (SQK-NBD114-96) before being loaded into a partial R10.4.1 PromethION flowcell (FLO-PRO114M) and sequenced on a PromethION 2 Solo device, a nanopore sequencer capable of generating long-read data in a single run, using Minknow version 24.06.15 with default settings.

Sequencing reads were demultiplexed using Dorado (v1.0.1) with the SQK-NBD114-96 kit and output as FASTQ files. Reads were aligned to the pUC19 reference sequence using minimap2 (v2.17), and the resulting alignments were sorted and indexed with samtools. Coverage statistics were generated using SAMtools depth. BAM files were converted to PAF format using htsbox. Read length distributions were calculated from the FASTQ files and visualized in R with ggplot2. All analyses were performed on the UNSW Katana computational cluster.

Detailed analysis is presented in Supplementary Discussion 2, and key scripts are provided in Supplementary Scripts.

### ICP-MS (Method 6)

1 mL solution of water containing 10 mg of Ga droplets was placed in a dialysis bag (MWCO 3500 Da) and dialyzed against 10 mL of water at room temperature with continuous magnetic stirring. The solution was collected at different time points (0.5, 2, 4, and 8 h) and analyzed for Ga^3+^ concentration using inductively coupled plasma plasma mass spectrometry (ICP-MS, PerkinElmer, USA).

### EPR spectroscopy (Method 7)

For the detection of ∙OH and O_2_ ∙ ^−^, Ga droplets (40 μL, 1 mg mL⁻¹ in EtOH) were subjected to solvent exchange into DI water. Briefly, EtOH was removed by centrifugation, followed by the addition of DI water and gentle pipetting to redisperse the Ga droplets prior to the experiment. DMPO was purified by treatment with activated charcoal, filtered, and stored as frozen aliquots prior to use. Then DMPO (4 μL, 100 mM) was added. Samples were measured without sonication or after sonication for 5 s or 30 s. Negative control samples contained only DI water and DMPO, with and without identical sonication. The positive control was the Fenton reaction; experimental details are described in the section on Fenton reaction–mediated DNA cleavage. EPR spectra were recorded after defined dead times (0-90 min) following sample preparation, as specified in each figure, to capture the temporal evolution of O_2_ ∙ ^−^ to ∙OH.

For the detection of singlet oxygen, Ga droplets (40 μL, 1 mg mL⁻¹ in EtOH) were solvent-exchanged into DI water. TEMP, freshly distilled at 152 °C was then added. Samples were measured directly without sonication. Negative control samples containing only DI water with TEMP were prepared. Positive control samples consisted of TEMP mixed with 50 μM methylene blue and irradiated with red light ( > 630 nm) to induce photosensitized singlet oxygen (^1^O_2_) generation.

All samples were immediately transferred into glass capillaries after different treatments and sealed with wax. Spectra were acquired at room temperature using a Bruker EMX X-band spectrometer (microwave power 20 mW; scan width 200 G).

### MS measurement (Method 8)

Five groups of samples were analyzed by mass spectrometry: Ga + 5 T ssDNA (1.5 h, biological replicates *n* = 3), Ga + 5 T ssDNA (4 h, *n* = 3), negative control (5 T ssDNA in DI water, 4 h, *n* = 3), DI water blank processed through ZipTip desalting (*n* = 1), and Fenton reaction positive controls (30 min, 2 h, and overnight; *n* = 1 each). All samples were prepared independently unless otherwise stated. Although the Fenton reactions were not performed in triplicate for each time point, all three reaction durations yielded identical oxidative cleavage peaks.

Desalted 5 T ssDNA (Thermo Fisher Scientific) was used as received and dissolved in DI water for the reaction. Reactions were performed in a total volume of 80 µL containing 40 µL of Ga droplets (2 mg mL⁻¹ in DI water) and 40 µL of 5 T ssDNA (0.1 µg µL⁻¹ in DI water). Samples were incubated at 22 °C for 1.5 h or 4 h without agitation. To release DNA adsorbed on Ga droplets, 2 µL of 10 mM Na₂HPO₄ was added and incubated for an additional 30 min at 22 °C. Ga droplets were removed by centrifugation at 4500 rpm for 45 s at 22 °C. The supernatant was collected and subjected to desalting.

Samples were desalted using C18 reverse-phase pipette tips following the standard oligonucleotide preparation protocol (Sample Preparation of Oligonucleotides Prior to MALDI-TOF MS Using ZipTip C18 and ZipTip μ-C18 Pipette Tips). Briefly, tips were conditioned with 50% acetonitrile (ACN)/Milli-Q water, equilibrated with 0.1 M triethylammonium acetate (TEAA, pH 7.0), washed sequentially with 0.1 M TEAA and Milli-Q water, and eluted with 50% ACN/Milli-Q water. The final elution volume was 8 µL. All the groups were processed using the identical desalting workflow. Fenton reactions were performed as positive controls for oxidative DNA cleavage as described in Supplementary Method 5. Fenton-treated samples were desalted and analyzed under identical mass spectrometry conditions.

Mass spectra were acquired using an LTQ Orbitrap XL mass spectrometer (Thermo Scientific, USA) equipped with a static nanospray ionization source. Analyses were performed in negative electrospray ionization (ESI) mode with a spray voltage of 1.1–1.5 kV, a capillary temperature of 200 °C, a capillary voltage of –35 V, and a tube lens voltage of –100 V. Full MS scans were recorded over an m/z range of 150–2000 at a resolution of 60,000 (at m/z 400) with one microscan. The maximum ion injection time was set to 10 ms with an automatic gain control target of 5 × 10^5^. Data acquisition and processing were performed using Xcalibur software (version 2.1, Thermo Fisher Scientific, Inc). All raw mass spectra are deposited in 10.5281/zenodo.19041335^43^.

### Statistics & reproducibility

No statistical method was used to predetermine sample size. All fluorescence measurements were performed on independent replicates. Quantitative data are presented as means ± SDs (n = 3 or 4). Data analysis was performed using Origin 2025b (10.25), Image J (version 1.8.0) and GraphPad Prism Software 10.5.0 (GraphPad Prism, San Diego, California, USA). Statistical comparisons were performed using one-way ANOVA followed by post-hoc Tukey’s multiple comparison test for absolute values for multiple groups calculated in GraphPad Prism. For DNA sequencing data, the Chi-square test was used. A p-value of < 0.05 was considered statistically significant. *p < 0.05, **p < 0.01, ***p < 0.001, and ****p < 0.0001. No statistical method was used to predetermine sample size. No data were excluded from the analyses, except in cases where individual points were clear anomalies inconsistent with the immediate trend. In these instances, only the aberrant point was removed, ensuring that a minimum of three biological replicates were retained for all conditions. The experiments were not randomized. The investigators were not blinded to allocation during experiments and outcome assessment.

### Software

All data were collected using commercial software integrated with the respective experimental instruments: CLARIOstar® Plus plate reader, JEOL JSM-IT 500 HR SEM, JEOL JEM-F200 TEM, Bruker EMX X-Band EPR, PerkinElmer ICP-MS, and Thermo LTQ Orbitrap XL. The data are analyzed by commercial software: ImageJ v1.8.0, Origin 2025b, GraphPad Prism v10.5.0 for general analysis; Minimap2 v2.17, SAMtools, Dorado v1.0.1, R with ggplot2 for DNA sequencing; Materials Studio 2022, DMol^3^ for DFT; Xenon for EPR analysis and Xcalibur^TM^ for MS analysis.

### Ethical statement

Human plasma experiments were approved by the UNSW Ethics Committee (UNSW HC210160), in addition to ACTRN12616000908437. All human saliva experiments were approved by the UNSW Ethics Committee (UNSW HC200568). All urine experiments were approved by the Ethics Committee of the First Affiliated Hospital of Chongqing Medical University, China (K2024-024-02). Written informed consent was obtained from all human sample donors.

### Reporting summary

Further information on research design is available in the Nature Portfolio Reporting Summary linked to this article.

## Supplementary information

Supplementary Information

Reporting Summary

Transparent Peer Review file

## Source data

Source Data

## Data Availability

All mass spectrometry data supporting the findings of this study have been deposited in Zenodo (available at: 10.5281/zenodo.19041335)^43^. All ONT sequencing data have been deposited in the National Center for Biotechnology Information GenBank under BioProject PRJNA1307762. The reference plasmid sequence (pUC19) is available in GenBank under accession number M77789.2 [https://www.ncbi.nlm.nih.gov/nuccore/M77789.2]. Source data are provided in this paper.

## Code availability

The scripts used for processing ONT DNA sequencing data are available in Code Ocean (10.24433/CO.3229876.v3)^22^.
