# Supplementary material for: Gallium in liquid state shows nuclease-mimicking activity
Source: Nat Commun. 2026 Apr 10;17:5126. doi: 10.1038/s41467-026-71346-7 (PMC13247054; doi:10.1038/s41467-026-71346-7)
Supplement: Supplementary file 1 — Supplementary Information [file 41467_2026_71346_MOESM1_ESM.pdf]

Supplementary Information

Gallium in Liquid State Shows Nuclease-Mimicking Activity

*Li Liu[#1,2], Jiewei Zheng[#3], Xi Lu[#4,5], Chowdhury Sarowar[6], Yuqin Wang[1], Martin A. Smith[7,8], Xin Wang[4,5], Fei Deng[9],Biswaranjan Mohanty[10,] Nur-Adania Nor-Azman[1], Fusheng Zhang[11], Shih-Hao Chiu[1,2], Mario Torrado[10], Yi Li[9,12], Shi-Yang Tang[4,5,13], Jianbo Tang[2,14], Michelle J.S. Spencer[3], Priyank V. Kumar[2], Kourosh Kalantar-Zadeh[*1], Chengchen Zhang[*4,5]*

[1]*School of Chemical and Biomolecular Engineering, University of Sydney, Sydney, New South Wales, Australia*

[2]*School of Chemical Engineering, UNSW Sydney, Sydney, New South Wales, Australia*

[3]*School of Science, RMIT University, Melbourne, Victoria, Australia*

[4]*Digital Health and Biomedical Engineering, School of Electronics and Computer Science, University of Southampton, Southampton, UK*

[5]*Institute for Life Sciences, University of Southampton, Southampton, UK*

[6]*Bioanalytical Mass Spectrometry Facility, Mark Wainwright Analytical Centre, UNSW Sydney, Sydney, New South Wales, Australia*

[7]*Ramaciotti Centre for Genomics, School of Biotechnology and Biomolecular Sciences, Faculty of Science, UNSW Sydney, Sydney, New South Wales, Australia*

[8]*Australian Centre for Nano Medicine, UNSW Sydney, Sydney, New South Wales, Australia*

[9]*School of Biomedical Engineering, Faculty of Engineering, UNSW Sydney, Sydney, New South Wales, Australia*

[10]*Sydney Analytical, Core Research Facilities, The University of Sydney, Sydney, New South Wales, Australia*

[11]*Center for AIE Research, Guangdong Provincial Key Laboratory of New Energy Materials Service Safety, College of Materials Science and Engineering, Shenzhen University, Shenzhen, 518055, China.*

[12]*Key Laboratory of Clinical Laboratory Diagnostics (Ministry of Education), College of Laboratory Medicine, Chongqing Medical University, Chongqing, China*

[13]*School of Mechanical and Manufacturing Engineering, UNSW Sydney, Sydney, New South Wales, Australia*

[14]*Department of Materials Science and Engineering, School of Engineering, Westlake University, Hangzhou, China*

[#]These authors contributed equally: Li Liu, Jiewei Zheng, Xi Lu

[*]Corresponding authors: Kourosh Kalantar-Zadeh (email: kourosh.kalantarzadeh@sydney.edu.au), Chengchen Zhang (email: Chengchen.Zhang@soton.ac.uk)

Table of Contents

Supplementary Methods 1
Supplementary Method 1. Kinetic analysis of single-stranded deoxyribonucleic acid (ssDNA) cleavage by Ga droplets 1
Supplementary Method 2. Preparation of bulk solid and liquid gallium (Ga) for Fig. 2c, d 3
Supplementary Method 3. Ga oxide preparation in Fig. 3 3
Supplementary Method 4. Electron paramagnetic resonance (EPR) spectral analysis and simulation 3
Supplementary Method 5. Fenton reaction–mediated DNA cleavage 4
Supplementary Method 6. Molecular dynamics (MD) simulations details 4
Supplementary Method 7. Density functional theory (DFT) computational methods 6
Supplementary Method 8. Comparison of ssDNA adsorption on GaOOH and Ga_2O_3 surfaces 7
Supplementary Method 9. Alloy preparation referred in Fig. 5 7
Supplementary Method 10. Reporter experiments in Fig. 5,6 7
Supplementary Method 11. Standard procedure for the evaluation of trans-ssDNA cleavage activity of Cas12a with Ga droplets 9
Supplementary Method 12. Commercial nuclease activity 9
Supplementary Discussion 10
Supplementary Discussion 1. Kinetic analysis of Ga droplets as nuclease mimics 10
Supplementary Discussion 2. DNA sequencing 11
2.1 Library preparation 12
2.2 Analysis workflow 13
2.2.1 Coverage analysis 13
2.2.2 Sequence preference analysis 13
2.3 Nucleotide preference at cleavage sites 14
Supplementary Discussion 3. Oxidation State Evolution of Ga Droplets 15
Supplementary Discussion 4. Mass spectrometry (MS) 16
4.1 Desalting 16
4.2 Hydrolytic or oxidative cleavage fragments analysis 17

4.3 Reasons for focusing on 5T and 4T ssDNA and low charge states in hydrolytic cleavage fragments....17
Supplementary Discussion 5. MD simulation for ssDNA and Ga oxide interaction....18
Supplementary Discussion 6. DFT calculation for ssDNA and Ga^0 and Ga^{3+} interaction....19
6.1 Energetic basis for enhanced 4T fragment yield in MS....19
6.2 Comparison of short and long ssDNA cleavage by Ga....20
Supplementary Discussion 7. Possible mechanism of Ga droplets' preference to T-T and A-A....20
Supplementary Figures....22
Supplementary Fig. 1....22
Supplementary Fig. 2....23
Supplementary Fig. 3....24
Supplementary Fig. 4....25
Supplementary Fig. 5....26
Supplementary Fig. 6....27
Supplementary Fig. 7....28
Supplementary Fig. 8....29
Supplementary Fig. 9....30
Supplementary Fig. 10....31
Supplementary Fig. 11....32
Supplementary Fig. 12....33
Supplementary Fig. 13....34
Supplementary Fig. 14....35
Supplementary Fig. 15....36
Supplementary Fig. 16....37
Supplementary Fig. 17....38
Supplementary Fig. 18....39
Supplementary Fig. 19....40
Supplementary Fig. 20....41
Supplementary Fig. 21....42

Supplementary Fig. 22. 43
Supplementary Fig. 23 44
Supplementary Fig. 24. 45
Supplementary Fig. 25. 46
Supplementary Fig. 26. 49
Supplementary Fig. 27. 50
Supplementary Fig. 28. 51
Supplementary Fig. 29. 52
Supplementary Fig. 30. 53
Supplementary Fig. 31 54
Supplementary Fig. 32 55
Supplementary Fig. 33. 56
Supplementary Fig. 34. 57
Supplementary Fig. 35. 58
Supplementary Fig. 36. 59
Supplementary Fig. 37. 60
Supplementary Fig. 38. 61
Supplementary Fig. 39. 62
Supplementary Table 63
Supplementary Table 1. DNA reporter and oligonucleotide used in the experiment 63
Supplementary Table 2. Kinetic parameters for Ga droplets acting as artificial nuclease mimics. 64
Supplementary Table 3. The frequency of dinucleotides at cleavage points by Ga droplets 65
Supplementary Table 4. Potential reactions and ROS chain reactions triggered by the introduction of Ga droplets into DI water. 66
Supplementary Table 5. Qualitative analysis of ssDNA related peaks in the MS spectra of samples following ZipTip desalting. 67
Supplementary Table 6. Qualitative analysis of major non-DNA-related peaks in the MS spectra of samples after ZipTip desalting. 68
Supplementary Table 7. Calculated m/z values of theoretical hydrolytic cleavage fragments (1T–5T) under different ionization states. 70

Supplementary Table 8. Observed m/z values of hydrolytic cleavage fragments (1T–5T) under different ionization states in control sample. 71

Supplementary Table 9. Observed m/z values of hydrolytic cleavage fragments (1T–5T) under different ionization states in Ga_1.5 h sample. 72

Supplementary Table 10. Observed m/z values of hydrolytic cleavage fragments (1T–5T) under different ionization states in Ga_4 h sample. 73

Supplementary Table 11. Calculated m/z values of theoretical oxidative cleavage fragments under different ionization states. 74

Supplementary Table 12. Calculated m/z values of theoretical fragments: product 1 to 5 in Supplementary. Fig. 26. 75

Supplementary Table 13. MD calculation results 79

Supplementary Table 14. Intracellular and extracellular ion types and concentrations 80

Supplementary Table 15. Commercial nuclease products with no phosphate in their formulations 81

Supplementary Table 16. Comparison between Ga droplets as nuclease mimics and commercial nucleases. 82

Supplementary References 83

Supplementary Methods

Supplementary Method 1. Kinetic analysis of single-stranded deoxyribonucleic acid (ssDNA) cleavage by Ga droplets

Fluorescence intensity $F(t)$(a.u.) from TR-ssDNA reporters was used to quantify the extent of ssDNA cleavage. To convert fluorescence signals into product formation, excess S1 nuclease was added at each temperature to achieve complete reporter cleavage, and the corresponding fluorescence intensity was taken as the maximum signal ($F_{\max}$) for that temperature. For each substrate (TR-ssDNA reporter) concentration, the maximum fluorescence intensity was obtained and used as a reference for data normalization (Supplementary Fig. 3).

Determination of initial reaction rates (v_0)

v_0 were determined from the early linear region of the fluorescence–time progress curves. A time window was selected in which the fluorescence increase was linear, and substrate depletion was negligible. The initial rate $v_0$was approximated as:

$$\boldsymbol{v_0} \approx \frac{\boldsymbol{F}_{\text{corr}}(\boldsymbol{t_2}) - \boldsymbol{F}_{\text{corr}}(\boldsymbol{t_1})}{\boldsymbol{t_2} - \boldsymbol{t_1}} \quad (1)$$

where $F_{\text{corr}}(t)$is the background-corrected fluorescence intensity at time t.

Determination of apparent $V_{\max}$and K_m

Steady-state kinetic parameters were determined independently at each temperature by fitting the initial rate data to the Michaelis–Menten model:

$$\boldsymbol{v_0}([\boldsymbol{S}], \boldsymbol{T}) = \frac{\boldsymbol{V}_{\max}(\boldsymbol{T})\,[\boldsymbol{S}]}{\boldsymbol{K_m}(\boldsymbol{T}) + [\boldsymbol{S}]} \quad (2)$$

where [S] is the TR-ssDNA reporter concentration (nM), $v_0$is the initial rate (a.u.·min^{-1} or nM·min^{-1}), $V_{\max}(T)$ is the apparent maximum reaction rate at the temperature T, and $K_m(T)$ is the apparent Michaelis constant. Nonlinear regression of $v_0$versus $[S]$was used to extract $V_{\max}$and K_m. By definition, $K_m$corresponds to the substrate concentration at which $v_0 = V_{\max}/2$.

Apparent turnover rate constant (k_{cat})

An apparent turnover rate constant was defined as:

$$\boldsymbol{k}_{\text{cat}} = \frac{\boldsymbol{V}_{\mathbf{max}}}{[\boldsymbol{E}]_{\text{eff}}} \tag{3}$$

where $[E]_{\text{eff}}$ represents the effective concentration of catalytically active Ga droplets. Because the effective catalyst concentration was identical for all temperatures, $[E]_{\text{eff}}$ was treated as a constant. Taking the natural logarithm:

$$\mathbf{ln}\ \boldsymbol{k}_{\text{cat}} = \mathbf{ln}\ \boldsymbol{V}_{\mathbf{max}} - \mathbf{ln}\ [\boldsymbol{E}]_{\text{eff}} \tag{4}$$

The constant term ln $[E]_{\text{eff}}$ affects only the intercept and does not influence the temperature-dependent slope used to extract the activation energy. Therefore, for Arrhenius analysis, the effective catalyst concentration was set to unity, and $k(T)$ was taken as $V_{\text{max}}(T)$.

Activation energy determination by Arrhenius analysis

The apparent activation energy was determined using the Arrhenius equation:

$$\boldsymbol{k}(\boldsymbol{T}) = \boldsymbol{A}\ \boldsymbol{e}^{-\boldsymbol{E_a}/(\boldsymbol{RT})} \tag{5}$$

Taking the natural logarithm gives:

$$\mathbf{ln}\ \boldsymbol{k} = \mathbf{ln}\ \boldsymbol{A} - \frac{\boldsymbol{E_a}}{\boldsymbol{R}}\frac{\mathbf{1}}{\boldsymbol{T}} \tag{6}$$

where T is the absolute temperature (K), calculated as $T(\text{K}) = T(^\circ\text{C}) + 273.15$, and R=8.314 J mol^{-1} K^{-1}. Arrhenius plots were constructed by plotting $\ln k$ (with $k = V_{\text{max}}$) against $1/T$. Linear regression ($y = b + mx$) was performed, and the activation energy was obtained from the slope:

$$\boldsymbol{E_a} = -\boldsymbol{mR} \tag{7}$$

Apparent turnover number (TON)

In chemical catalysis, the turnover number (TON) is defined as the total number of substrate molecules converted per catalyst unit before deactivation:

$$\text{TON} = \frac{\boldsymbol{n}_{\text{product}}}{\boldsymbol{n}_{\text{catalyst}}} \tag{8}$$

In this study, the amount of cleaved ssDNA reporter was 41.7 ± 4.2nM, and the Ga concentration was 0.1 mg·mL^{-1}, corresponding to 1,434,246.9 nM. Because only the surface of Ga droplets is considered catalytically active, the TON reported here is an apparent turnover number and is provided for reference only. The apparent TON was calculated to be $2.9 \pm 0.29 \times 10^{-5}$.

Supplementary Method 2. Preparation of bulk solid and liquid gallium (Ga) for Fig. 2c, d

Due to the supercooling effect[1], melted bulk Ga can remain in a liquid state below its melting point 29.8°C. We also confirmed it by doing differential scanning calorimetry (DSC) analysis (Supplementary Fig. 4) using a simultaneous thermal analyzer (SDT 650, TA Instruments, USA), which is consistent with the data shown in previous studies[2]. The maximum sample weight in the TA instrument was approximately 200 mg, corresponding to a bulk Ga volume of around 0.034 cm^3.

To investigate the effect of phase state on deoxyribonucleic acid (DNA) cleavage activity (Fig. 2c, d), gallium (Ga) samples in both droplet and bulk forms were prepared in liquid and solid states using controlled thermal histories. For Ga droplets, suspensions stored in ethanol (EtOH,1 mg mL^{-1}) were placed at -30 °C for one week to eliminate supercooling and ensure complete solidification. The solidified Ga droplets were collected by centrifugation at 4 °C and then introduced into the TR-ssDNA reporter solution. All other experimental conditions were identical to those used for liquid-state Ga droplets. For bulk Ga, equal masses (0.1 g) of liquid and solid Ga were prepared prior to use (Fig. 2d). Liquid Ga was obtained by heating the sample to 50 °C and maintaining it at 35 °C to ensure a fully molten state. Solid Ga was either used directly in its native solid form or prepared by melting, followed by cooling to 4 °C to induce solidification. Both solid and liquid bulk Ga samples were co-incubated with the TR-ssDNA reporter (Supplementary Table 1), and fluorescence intensity was continuously monitored for 2 h using a plate reader (CLARIOstar® Plus, BMG LABTECH, USA) at excitation/emission wavelengths of 570/615 nm.

Supplementary Method 3. Ga oxide preparation in Fig. 3

Gallium oxyhydroxide (GaOOH) was prepared by incubating Ga droplets in deionized (DI) water at 60 °C for 6 hours[3]. Ga oxide (Ga_2O_3) was prepared by annealing Ga droplets in ambient air at 500 °C for 120 min[4].

Supplementary Method 4. Electron paramagnetic resonance (EPR) spectral analysis and simulation

Continuous-wave X-band EPR spectra were analyzed using the SpinFit module in Xenon (Bruker BioSpin), which employed perturbation theory, with the electron Zeeman interaction treated as the dominant term and isotropic hyperfine coupling included as a first-order perturbation. Experimental spectra were fitted using multicomponent simulations to account for overlapping signals arising from different 5,5-dimethyl-1-pyrroline N-oxide (DMPO) spin-trapped radical adducts. Hyperfine coupling constants, including the g value of each radical adduct, were initially loaded from the SpinFit library. A

single isotropic g value was assumed for each radical species. Least-squares fitting was used to optimize hyperfine coupling constants, line positions, line widths, and relative component intensities simultaneously, enabling estimation of the relative contributions of individual radical species. The quality of the fits was assessed by inspection of the residual spectra, defined as the difference between the experimental and simulated spectra. Following spectral simulation, the hyperfine coupling constants of each DMPO radical adduct were compared with values reported in the NIEHS spin trap database: https://www.niehs.nih.gov/ research/resources/databases/spintrap.

The hyperfine coupling constants used in the simulations were as follows. 1. Ga in water without sonication: DMPO-hydroxyl radical (DMPO-OH) (aN = 15.05 G, aH = 14.45 G), DMPO-hydroperoxyl radical (DMPO-OOH) (a_N = 14.15 G, a_H^β = 11.27 G, a_H^γ = 1.21 G), DMPO-1-hydroxyethyl (DMPO-CH(OH)CH_3)· (a_N = 15.87 G, a_H = 22.97 G), and a triplet attributed to partially degraded DMPO: a_N = 14.91 G; 2. Ga in water sonicated 5s_deadtime 30s: DMPO-OH (a_N = 14.99 G, a_H = 14.55 G), DMPO-OOH (a_N = 14.11 G, a_H^β = 11.31 G, a_H^γ = 1.22 G), DMPO-CH(OH)CH_3 (a_N = 15.87 G, a_H = 22.96 G), and a triplet attributed to partially degraded DMPO: a_N = 15.02 G; 3. Ga in water sonicated 5s_deadtime 90 min: DMPO-OH (a_N = 14.94 G, a_H = 14.57 G), DMPO-CH(OH)CH_3 (a_N = 15.85 G, a_H = 22.88 G), and a triplet attributed to partially degraded DMPO: a_N = 14.66 G.

For the DMPO-OOH adduct, because DMPO spin trapping cannot distinguish between superoxide radical ($O_2{\cdot}^-$) and its protonated form, the hydroperoxyl radical (·OOH), radical species assignment is guided by the solution pH[5]. Under neutral conditions (DI water), $O_2{\cdot}^-$ predominates ($O_2{\cdot}^- + H^+ \rightleftharpoons {\cdot}OOH$, pK_a ≈4.9), and the DMPO-OOH signal is therefore attributed to $O_2{\cdot}^-$ [5, 6].

Supplementary Method 5. Fenton reaction–mediated DNA cleavage

ssDNA (2 mg mL^{-1}) was incubated with Fe^{2+} (from $NH_4Fe(SO_4)_2 \cdot 12H_2O$, 20 µM), ascorbic acid (0.1 mM), and hydrogen peroxide (H_2O_2,10 mM) to induce oxidative cleavage. Reactions (total volume, 20 µL) were carried out for 2 h or 16 h and quenched by adding 2 µL of 1 M ethylenediaminetetraacetic acid (EDTA), followed by brief mixing. Samples were allowed to stand for 10–20 min to ensure complete metal chelation, placed on ice to suppress residual reactions, desalted using ZipTip, and analyzed using an LTQ Orbitrap XL mass spectrometer equipped with static nanospray (Thermo Scientific, USA).

Supplementary Method 6. Molecular dynamics (MD) simulations details

Classical MD simulations were performed using the Large-scale Atomic/Molecular Massively Parallel Simulator (LAMMPS) to investigate the interactions between the single-stranded DNA (ssDNA) and

the oxide layer surfaces.[7] Force field parameters for the Ga atoms were initially adapted from the Universal Force Field (UFF) parameters for aluminium (Al), given their similar chemical properties as group 13 elements.[8] These parameters were subsequently refined through iterative optimization until the simulated system density was within 10% of the experimental values.

To model the Ga_2O_3 substrate, the (100) surface was cleaved from the most stable β-Ga_2O_3 bulk structure.[9] The optimised surface dimensions were 54.75 × 58.06 × 24.77 $Å^{3}$, and the model contained 2880 Ga and 4320 O atoms. The GaOOH substrate was constructed using the bulk crystal structure obtained from the Materials Project database and cleaving the (110) surface with dimensions of 45.71 × 62.22 × 14.91 $Å^3$ and containing 1200 Ga, 1200 H, and 2400 O atoms. This surface was chosen as it corresponds to the dominant diffraction peak, as consistently reported in prior studies[10, 11]. The optimized force field parameters for both oxides are presented in Supplementary Table 13. After iterative optimization, the systems reached equilibrium densities of 6.05 g/cm^3 for Ga_2O_3 and 4.68 g/cm^3 for GaOOH after 5 ns *NPT* simulations (1 fs timestep), in good agreement with experimental values of 5.89 g/cm^3 and 5.20 g/cm^3, respectively.

The ssDNA-water layer was constructed by randomly distributing water molecules to achieve a density of approximately 1.0 g cm^{-3} to account for solvent effects. This ssDNA-water layer was then added to the simulation boxes of the Ga_2O_3 and GaOOH substrates. Force field parameters for the ssDNA and the water molecules were taken from the AMBER force field.[12] Molecular dynamics simulations were performed for 5 ns using the *NVT* ensemble at 305 K for 8 systems: Ga_2O_3-ssDNA-two thymine (2T), Ga_2O_3-ssDNA-two guanine (2G), GaOOH-ssDNA-2T, and GaOOH-ssDNA-2G, with each system simulated with the ssDNA initially oriented parallel and perpendicular to the surfaces. Non-bonded intramolecular interactions were handled using AMBER scaling: 1-2 and 1-3 interactions were excluded, while 1-4 electrostatic and van der Waals interactions were scaled by factors of 1/1.2 and 1/2, respectively.[13] The temperature was maintained using the Nosé-Hoover thermostat[14, 15]. Non-bonded interactions were treated with cutoff distances of 8.5 Å for van der Waals forces and 15 Å for electrostatic interactions. Geometric mixing rules were used for the pairwise van der Waals interactions. All simulations employed a 1 fs timestep, with atomic coordinates recorded every 10 ps for the trajectory analysis. Binding energies (E_{bin}) were calculated using the following equation:

$$E_{bin} = E_{total} - E_{substrate} - E_{solvated\_DNA} \quad (9)$$

where E_{total} is the total energy of the combined system after 5 ns *NVT* equilibration, $E_{\mathrm{substrate}}$ is the equilibrated energy of the isolated substrate (Ga_2O_3 or GaOOH), and $E_{\mathrm{solvated\_DNA}}$ is the energy of the solvated ssDNA layer. All binding energies were calculated in the presence of explicit water molecules to account for solvation effects.

Supplementary Method 7. Density functional theory (DFT) computational methods

All studied molecules were constructed using the Materials Studio 2022 software package.[16, 17, 18] DFT calculations were performed using the DMol^3 code.[19, 20, 21] The exchange-correlation functional was described using the generalized gradient approximation (GGA) with the Perdew-Burke-Ernzerhof (PBE) functional.[22, 23, 24] Effective core potentials were employed to account for relativistic effects.[25] A double numerical atomic orbital basis set augmented with polarization p-functions (DNP) was used, which is roughly equivalent in size to the 6-31G** Gaussian basis set.[26] Spin-unrestricted calculations were performed throughout. Grimme's dispersion correction (DFT-D) was applied to account for weak van der Waals interactions[27]. The energy and force convergence criteria for all geometry optimizations were set to 1.0×10^{-5} Hartree and 4.0×10^{-3} Hartree·Å^{-1}, respectively. A smearing value of 0.005 Hartree was used in all calculations.

ssDNA models were constructed as 5-mer nucleotides and 2-mer nucleotides. The 5-mer nucleotide sequence was designed as 5'-TTTTT-3' (5T ssDNA), while two types of 2-mer nucleotides were constructed, with sequences 5'-TT-3' (2T ssDNA) and 5'-GG-3' (2G ssDNA). Both termini carried hydroxyl groups to maintain consistency with the 5T ssDNA used in MS analysis. The phosphate diester bonds from the 5' to 3' end in 5T ssDNA were labeled as DNA5T-1, DNA5T-2, DNA5T-3, and DNA5T- 4.

DNA cleavage was shown to proceed *via* three sequential steps. In the first step, Ga adsorbs to the phosphate group of the DNA backbone:

$$\text{DNA+ Ga} \rightarrow \text{DNA-Ga} \qquad \text{Step 1}$$

In the second step, ·OH cleaves the DNA-Ga complex, generating two distinct fragments. Fragment 1 contains a 3'-hydroxyl group (3'-OH), while Fragment 2 retains the Ga-bound phosphate group at the 5'-end:

$$\text{DNA-Ga + ·OH} \rightarrow \text{DNA-1 + DNA-2-Ga} \qquad \text{Step 2}$$

In the final step, Ga is desorbed from Fragment 2:

$$\text{DNA-2-Ga} \rightarrow \text{DNA-2 + Ga} \qquad \text{Step 3}$$

Relative energy diagrams were constructed by setting the combined total energy of the ssDNA, Ga, and ·OH radical systems as the reference state (zero point). The relative energy of each reaction step was calculated by subtracting this reference energy from the total energy of the corresponding products.

Calculations for the Ga ion (Ga^{3+}) -ssDNA system followed the same reaction sequence and energy-referencing scheme, providing qualitative insight into the relative energetics of Ga^0- and Ga^{3+}-involved DNA cleavage pathways.

Supplementary Method 8. Comparison of ssDNA adsorption on GaOOH and Ga_2O_3 surfaces

To evaluate the adsorption efficiencies of Ga_2O_3 and GaOOH toward 5T-ssDNA and 5G-ssDNA TR- reporters were employed. Ga_2O_3 or GaOOH particles (prepared as described in Supplementary Method 3) were incubated with the ssDNA reporters for 5 min to allow adsorption. DI water without Ga_2O_3 or GaOOH particles served as the control for adsorption 0%. The suspensions were then centrifuged to pellet the particles, and the supernatants containing unadsorbed ssDNA were collected. The supernatants were subsequently treated with S1 nuclease to digest the unadsorbed ssDNA reporters. The resulting fluorescence intensity therefore reflects the fraction of ssDNA remaining unadsorbed after incubation with Ga_2O_3 or GaOOH.

Supplementary Method 9. Alloy preparation referred in Fig. 5

The preparation of Ga-based alloys followed previously established protocols[28, 29, 30]. Owing to the unique ability of liquid Ga to dissolve a wide range of metals at relatively mild temperatures (< 400 °C), this approach provides a straightforward route for alloy formation[30]. Using Ga-silver (Ag) as an example, Ag (0.1 g) was added to bulk liquid Ga (10 g). The mixture was manually ground at approximately 200 °C with a mortar and pestle inside a nitrogen-filled glove box to prevent oxidation of the liquid metal. The grinding process was carried out for approximately 40 min, after which the mixture was left undisturbed for an additional two hours to ensure complete alloying. The resulting alloy exhibited a smooth and reflective appearance. The preparation of other Ga alloys followed the same procedure. The Ga alloys obtained were subsequently sonicated into nanoparticles, using the same method as for Ga droplets.

Supplementary Method 10. Reporter experiments in Fig. 5,6

The Ga droplets in EtOH underwent solvent exchange with DI water *via* centrifugation, then were vortexed or bath-sonicated for 5 min to ensure dispersion. A mixture of 90 μL of 166 nM reporter and 10 μL of Ga droplets was added to each well of a 96-well plate, with at least three replicates per group. At different times as indicated in the main figures, various external stimuli or solutions were applied to assess their effects on Ga droplets' nuclease-mimicking activity.

For external stimuli, the solution in each group was transferred to a reaction tube (Eppendorf Tubes 1.5 mL, Eppendorf, Germany). Each arrow shown in the figures indicates a single treatment. The following are the experimental procedures for single treatment by different stimuli.

Ultrasonication was performed by placing the reaction tube in a bath sonicator (Ultrasonic Cleaner, FXP10 Series; Australian Scientific, Australia) for 1 min. Laser irradiation was conducted using an

808 nm infrared (LSR-PS-II, LASEVER, China) laser with 2 W output. The reaction tube was irradiated for 5 min, and the temperature was monitored in real time using a thermal camera (HIKMICRO Mini2 V2). Immediately after irradiation, 100 μL of the solution was transferred to a 96-well microplate, and fluorescence intensity was measured using a microplate reader under consistent settings. Thermal treatment was performed using a temperature controller (Eppendorf ThermoMixer C, Eppendorf, Germany) to precisely control the temperature of the reaction tubes.

For solution modulation, 10 μL of test solution (e.g., ascorbic acid, varying pH buffers, or sodium bicarbonate ($NaHCO_3$) at different concentrations) was added to 90 μL of the TR-ssDNA reporter solution containing Ga droplets in each well of a 96-well plate. The concentrations indicated in the figures represent the final concentrations after addition to the reaction solution.

Simulated gastric fluid was prepared by dissolving 0.2% (w/v) sodium chloride (NaCl) in 0.7% hydrochloric acid (HCl), following the formulation provided by Avantor®. Simulated intestinal fluid was prepared by dissolving potassium phosphate monobasic (1.02 g) and sodium dodecyl sulphate (SDS) (0.375 g) in 150 mL of DI water, followed by pH adjustment to 6.8 ± 0.1 using sodium hydroxide[31].

Human plasma was obtained commercially, while saliva and urine samples were procured from healthy adult volunteers following informed consent and institutional guidelines. For the assay, 10 μL of each biofluid was added to 90 μL of TR-ssDNA reporter solution, resulting in a 1:10 dilution.

For each experiment, different control groups were included to rule out the influence of other variables. Because fluorescence ratios (F/Fc) normalize to the control and Fc/Fc ≈ 1, only one representative control group is shown for clarity and simplicity in figure presentation. Taking the experiment of Ga droplets in phosphate-buffered saline (PBS) recovered by laser as an example, the experimental groups are as follows:

1. TR-ssDNA reporter in DI water
2. TR-ssDNA reporter in PBS
3. TR-ssDNA reporter + Ga droplets in DI water
4. TR-ssDNA reporter + Ga droplets in PBS
5. TR-ssDNA reporter in PBS + laser
6. TR-ssDNA reporter + Ga droplets in PBS + laser

The following fluorescence intensity ratios are defined:

- A (Ga droplets in PBS) = (Fluorescence intensity of TR-ssDNA reporter + Ga droplets in PBS) / (Fluorescence intensity of TR-ssDNA reporter in PBS)
- B (Ga droplets in PBS + laser) = (Fluorescence intensity of TR-ssDNA reporter + Ga droplets in PBS + laser) / (Fluorescence intensity of TR-ssDNA reporter in PBS + laser)

- C (Ga droplets in water) = (Fluorescence intensity of TR-ssDNA reporter + Ga droplets in DI water) / (Fluorescence intensity of TR-ssDNA reporter in DI water)
- D (Control) = (Fluorescence intensity of TR-ssDNA reporter in PBS + laser) / (Fluorescence intensity of TR-ssDNA reporter in PBS)

The recovery rate is calculated as: Recovery rate (%) = (B / C) × 100%

Supplementary Method 11. Standard procedure for the evaluation of trans-ssDNA cleavage activity of Cas12a with Ga droplets

The standard CRISPR/Cas12a assay was performed according to established protocols. Briefly, the reaction mixture was prepared by gently mixing 1 μL of Cas12a protein (100 μM, 100 pmol) with 5 μL of specifically designed guide ribonucleic acid (gRNA) (20 μM, 100 pmol) in 3.6 mL of 1× NEB 2.1 buffer. Next, 6 μL of TR-ssDNA reporter (100 μM, 0.6 nmol) was added and thoroughly mixed to form the standard reaction mixture. Ga droplets (0.1 mg/mL) were then added, and the reaction was carried out and recorded at room temperature.

Supplementary Method 12. Commercial nuclease activity

S1 nuclease, Benzonase, and DNase I digestions were performed according to the manufacturers' instructions. S1 nuclease reactions were conducted in acetate-based buffer containing zinc ions (Zn^{2+}), while Benzonase and DNase I reactions were in Tris-based buffers supplemented with magnesium ions (Mg^{2+}) and calcium ions (Ca^{2+}) for DNase I. All reactions were incubated at 37 °C for the indicated time. Reporter DNA concentrations were identical to those used in the Ga-droplet experiments (100 μL, 166 nM per well; 0.024 μg ssDNA per well). Nucleases were added at 0.033 U per well (1 U defined as activity per 1 μg DNA).

Supplementary Discussion

Supplementary Discussion 1. Kinetic analysis of Ga droplets as nuclease mimics

To quantitatively evaluate the nuclease-mimic behaviour of Ga droplets, we performed fluorescence-based steady-state kinetic analysis of ssDNA cleavage (Supplementary Fig. 3). The reaction rates exhibited clear substrate-saturation behaviour and could be described using an apparent Michaelis–Menten framework, yielding maximum reaction velocity (V_{max}) and Michaelis constant (K_m) values (Supplementary Table 2; Supplementary Method 2).

Although Ga droplets do not possess discrete, well-defined active sites analogous to protein enzymes, the extracted parameters provide a rigorous quantitative description of the maximal cleavage velocity and the effective substrate affinity of the reactive interface. The observed saturation behaviour suggests that the rate-limiting step involves the interfacial association of DNA with the Ga-oxide surface prior to radical-mediated scission. By applying this framework, we demonstrate that the Ga-droplet platform effectively recapitulates the kinetic signature of natural nucleases, providing a high-efficiency pathway for DNA degradation.

Arrhenius analysis of the temperature-dependent kinetic parameters yielded an apparent activation energy of 16.87 $kJ \cdot mol^{-1}$, which is substantially lower than the activation energy typically reported for uncatalyzed phosphodiester hydrolysis of DNA (~90–120 $kJ \cdot mol^{-1}$)[32]. This value is comparable to activation energies reported for radical-mediated Fenton-type reactions (typically ~20–40 $kJ \cdot mol^{-1}$)[33] and falls within the range of apparent activation energies observed for enzymatic nucleases and artificial nuclease systems (~10–60 $kJ \cdot mol^{-1}$)[34, 35]. This significant reduction in energetic barrier supports the conclusion that Ga droplets function as effective nuclease mimics, facilitating DNA backbone cleavage through an accelerated pathway. The lowered apparent energy activation is consistent with a mechanism in which surface-mediated processes, such as substrate adsorption, electronic interactions with the Ga surface, and ·OH generation, collectively reduce the energetic cost of phosphodiester bond scission.

It is important to note that the activation energy derived here represents an apparent activation energy, as the experimentally determined kinetic parameters are influenced by Ga droplet concentration, particle size distribution, and surface oxidation state. Accordingly, the reported value should be interpreted as a system-specific reference rather than an intrinsic elementary reaction barrier.

The cleavage efficiency of Ga droplets was further evaluated by estimating an apparent turnover number (TON) following conventions commonly used in organometallic and heterogeneous reagent-mediated scission. Because only the surface of Ga droplets is considered reactive and the number of accessible active sites cannot be rigorously quantified, the resulting TON is necessarily an apparent turnover number and is provided for reference. The relatively low apparent TON observed under the present conditions is attributed to progressive surface oxidation of Ga droplets in aqueous environments

(Supplementary Discussion 3), which limits sustained activity over multiple cycles (Supplementary Fig.21). Previous studies have demonstrated that Ga oxide can be removed or reduced through chemical or electrochemical treatments followed by re-sonication, enabling regeneration of reactive Ga droplets[36, 37]. While such regeneration strategies could, in principle, extend the functional lifetime of the platform, they were not explored in this work to maintain focus on elucidating the intrinsic DNA cleavage behaviour.

Taken together, Ga droplets function as nuclease mimics by lowering the apparent energetic barrier for DNA cleavage. Nevertheless, their mode of action is fundamentally different from that of canonical enzymes, as it is dictated by surface chemistry and materials properties rather than by the precise spatial arrangement of a discrete active site. By bridging the gap between heterogeneous inorganic chemistry and biochemical function, this platform provides a unique, ligand-free alternative for achieving accelerated DNA scission.

Supplementary Discussion 2. DNA sequencing

Oxford Nanopore Technology (ONT) sequencing is a third-generation DNA sequencing technology that guides single DNA molecules through a nanoscale protein pore, measuring changes in ionic current and translating them into nucleotide sequences[38].

There are two main reasons why we chose ONT for DNA sequencing. First, ONT is well known for its capacity to generate ultra-long reads, with individual reads exceeding 2.3 Mbp having been reported. This is essential for preserving the integrity of DNA fragments generated by Ga droplets. In contrast, traditional sequencing methods such as Sanger sequencing are limited to read lengths of around 1 kb, thus require digestion of the sample prior to sequencing, which can introduce additional cleavage sites and thereby mask those originally induced by Ga droplets. Second, the ONT native barcoding protocol does not involve polymerase chain reaction (PCR) amplification or enzymatic fragmentation of DNA, both of which are common in other platforms. This avoids polymerase errors or sequence-dependent amplification introduced significant bias, particularly in AT- or GC-rich regions. Therefore, the ONT workflow can truly preserve the original cleavage pattern, allowing accurate profiling of the original cleavage pattern of Ga droplets.

ONT sequencing was performed on pUC19 plasmids treated with Ga droplets for 1 h, 4 h, and 8 h, untreated pUC19 as a negative control, and HindIII-digested pUC19 as a positive control. The Ga_8h group yielded a very low number of sequencing reads, likely due to the strong adsorption of DNA fragments onto Ga droplets, making their desorption difficult. As a result, this sample was excluded from further analysis.

The key scripts used in the ONT sequencing analysis pipeline are provided in https://codeocean.com/capsule/3229876/tree.

2.1 Library preparation

We used the Oxford Nanopore Native Barcoding Kit 96 V14 (SQK-NBD114.96) (Oxford Nanopore Technologies, Ligation sequencing amplicons – native barcoding kit 96 v14, SQK-NBD114.96; available at https://nanoporetech.com/document/ligation-sequencing-amplicons-native-barcoding-v14-sqk-nbd114-96; accessed 18 July 2022) to perform a ligation-based library preparation. In brief, plasmid DNA (1000 ng per sample) was first repaired and end prepared. This step fills in any single-stranded overhangs or nicks, repairs base damage, and adds a 3′-adenine overhang to form A-tailed DNA. The main purpose is to produce blunt-ended DNA fragments that are compatible with the later adapter ligation. Next, native barcodes (unique DNA tags) were ligated to the prepared DNA ends. Each barcode adapter carries a 5′-T overhang that anneals to the A-tailed DNA, and a cohesive end for later adapter attachment. Barcoded samples were then pooled together for sequencing. Finally, the sequencing adapters, which contain the motor protein for nanopore capture, were ligated onto the barcode-tagged DNA. The resulting library fragments, therefore, have the structure:

[Sequencing Adapter]– [Barcode Adapter]– [DNA]– [± Barcode Adapter (RevComp)]– [± Sequencing Adapter (RevComp)].

End repair is crucial and necessary because the sequencing and barcode adapters carry complementary overhangs (e.g., a 3′-T), which can only ligate efficiently to these polished ends. Without end repairing, damaged or degraded ends would often fail to attach adapters, compromising the library. Therefore, we followed the manufacturer’s protocol for combining FFPE-repair and dA-tailing (NEBNext Ultra II End Repair/dA-Tailing). However, there is an inherent limitation of end repair for this study: Because polymerases “fill in” overhangs, the original cutting nucleotide becomes ambiguous. This means that a break between an A-T base pair versus a C-G base pair will yield the same blunt-ended fragment. Nanopore sequencing thus cannot distinguish these two scenarios at single-base resolution. For example, Szczelkun *et al.* have reported, polymerase/exonuclease end-repair causes additional processing, in which overhangs are “filled back in” and become “invisible” to downstream analysis[39]. In summary, end-repair necessarily degrades the base-pair-level resolution at breakpoints: after processing, an A and T, C and G break leaves an identical outcome and cannot be told apart in the read. Therefore, complementary dinucleotides are analyzed together, for example, AA and TT, or CG and GC.

Sequencing was performed on a PromethION 2 Solo system using an R10.4.1 flow cell and MinKNOW (v24.06.15), which provides substantially higher throughput and improved data quality. The PromethION sequencing data were basecalled and demultiplexed using Dorado with default settings.

Raw reads were aligned to the pUC19 reference sequence and stored as Binary Alignment/Map (BAM) files, which were subsequently converted to Pairwise mApping Format (PAF) files for downstream analysis.

2.2 Analysis workflow

The initial results show that there is a strong, sharp peak in the HindIII-treated sample, consistent with the known restriction site. However, similar peaks were also observed in untreated control samples. This pattern indicated barcode cross-contamination, where reads from the HindIII-digested sample were misassigned to other barcode groups. To resolve this problem, we used stringent demultiplexing - barcode-both-ends -. This option ensures that double-ended barcodes are detected on both ends before classification. After applying this step, the barcode misassignment was significantly reduced.

Demultiplexing was performed with adapter trimming enabled by default, so no additional trimming was required. Each demultiplexed read was then aligned to the pUC19 reference plasmid sequence using Minimap2. The output was saved in standard BAM format and subsequently converted to PAF files for further analysis.

2.2.1 Coverage analysis

To assess coverage, we computed the per-base depth across the pUC19 reference from the aligned reads using samtools depth. This yielded the number of reads covering each nucleotide. We plotted this coverage in R to check for uniform sequencing and any biases.

The results show the coverage was relatively even in all samples, indicating the uniform sequencing and that no significant biases were produced. There is a clear peak of HindIII-treated pUC19, as the end repair caused a double quantity of DNA in the sticky end area. This also proves the right demultiplexing and alignment we performed.

2.2.2 Sequence preference analysis

To assess whether Ga droplets exhibit sequence preference during DNA cleavage, we analysed the start and end positions of aligned sequencing reads. The idea was to determine whether specific positions along the DNA sequence showed significant enrichment, which would suggest cleavage specificity.

We used the PAF output from Minimap2 for this analysis. PAF provides detailed alignment information for each sequencing read. In particular, it includes query_length (total number of bases in the read),

query_start, and query_end, the positions at which each read aligns to the reference, using a zero-based coordinate system.

Columns 8 and 9 of the PAF file record the alignment start and end positions on the reference sequence. To ensure compatibility with R and standard genomic coordinates, we converted the start positions to a one-based system: start_positions <- paf [[8]] + 1; stop_positions <- paf [[9]]. The start is adjusted by +1 since R uses 1-based indexing. The end position in PAF is exclusive, but for breakpoint counting, it can be used directly.

As a validation, reads from the HindIII-digested control showed a sharp, consistent breakpoint at position 234–237 (Supplementary Fig. 7). This corresponds to the known HindIII restriction site and confirms the validity of our pipeline. In contrast, Ga-treated samples showed no obvious enrichment at any specific cleavage site (Supplementary Fig. 8). No position stood out with significantly higher read start or end counts. This suggests that Ga droplets do not cleave DNA in a position-specific manner, or they do not show sequence preference.

2.3 Nucleotide preference at cleavage sites

To explore whether Ga droplets prefer to cleave certain nucleotides, we analysed the dinucleotide context around each inferred cleavage site.

For each start and end position on both strands, we extracted the surrounding dinucleotide: For the start, the base before and at the start; for the end, the base at and after the end.

Strand information was handled carefully. Reads aligned to the negative strand were analysed using the same method as the positive strand to simplify the code, but a reverse complement of the negative strand was performed later during comparison. Then we counted the frequency of all possible dinucleotides (e.g., AA, AT, CG, etc.) and calculated their relative proportions. The differences were then evaluated using the Chi-squared test to identify statistically significant deviations.

To clearly show the results and avoid bias introduced by library preparation or sequencing, we subtracted the dinucleotide proportions observed in the untreated control (pUC19-only) samples, and the result is shown in Fig. 1i.

Supplementary Discussion 3. Oxidation State Evolution of Ga Droplets

Upon the transfer from EtOH to DI water, the formation of ·OH is accompanied by the oxidation of metallic (Ga^0) into Ga. After 24 h in DI water (Supplementary Fig. 19), SEM images reveal a gradual morphological transformation of Ga droplets into rod-like oxide structures. Correspondingly, the Ga 3d X-ray photoelectron spectroscopy (XPS) spectrum exhibits a dominant peak at ~20 eV, characteristic of Ga^{3+} species**.** Together, these results indicate the formation of GaOOH [11, 36, 37]. Consistently, after 24 h, radical signals were no longer detectable by EPR, and DNA cleavage activity was fully suppressed (Supplementary Fig. 20).

Based on these observations, together with the ·OH signals detected in Fig. 3 and prior literature, the reaction pathway of Ga droplets in water is summarized below.

Upon contact with water, two reactions occur: First, Ga droplets were prepared in EtOH, with Ga_2O_3 present on their surface. Upon exposure to aqueous solution, Ga_2O_3 gradually transforms into Ga $(OH)_3$, then GaOOH *via* Reaction 1[36, 37, 40].

Reaction 1:

$$\mathbf{Ga_2O_3 + 3H_2O \rightarrow 2Ga(OH)_3} \tag{10}$$

$$\mathbf{Ga(OH)_3 \rightarrow GaOOH + H_2O} \tag{11}$$

Based on ·OH detection in the EPR and previous research on GaOOH, we first propose the following reaction pathway (reaction 2). The corresponding reduction potentials and potential ROS chain reactions are provided in Supplementary Table 4.

Reaction 2:

$$\mathbf{2Ga + 2H_2O + O_2 \rightarrow 2GaOOH + H_2} \tag{12}$$

$$\mathbf{2Ga \rightarrow 2Ga^{3+} + 6e^-} \tag{13}$$

$$\mathbf{O_2 + 2e^- + 2H_2O \rightarrow 4 \cdot OH} \tag{14}$$

$$\mathbf{2Ga^{3+} + 4 \cdot OH\ + 4e^- \rightarrow 2GaOOH + H_2} \tag{15}$$

The above equation indicates that electron transfer from Ga droplets to dissolved oxygen in the presence of water is essential for ·OH generation.

Because the oxidation of Ga droplets in aqueous environments is unavoidable, Ga droplets' ssDNA reporter cleavage activity could only be maintained for up to three cycles (each cycle ~ 90 min) (Supplementary Fig. 21). Previous studies have shown that surface Ga oxides can be removed or reduced through acidic or basic treatments, electrochemical reduction, followed by re-sonication to regenerate Ga droplets[36, 37]. Although such regeneration strategies could, in principle, restore reactive Ga droplets for additional cleavage cycles, they were not investigated in the present work in order to maintain a clear focus on the intrinsic DNA cleavage mechanism. Moreover, Ga droplets are considerably less expensive than commercial nucleases, as presented in Supplementary Table 16, such that high recyclability is not a critical requirement for their practical use.

Supplementary Discussion 4. Mass spectrometry (MS)

4.1 Desalting

Salts in the sample can significantly affect DNA's mass spectrometric signal intensity, known as ion suppression[41]. Therefore, an effective desalting step is essential for accurate mass spectrometry (MS) analysis.

Among the available desalting methods: ZipTip desalting[42], EtOH precipitation[43], and HPLC[44]; ZipTip was identified as the most suitable method for our study. There are several reasons:

ZipTip is more effective for small DNA fragments. In EtOH precipitation, large oligonucleotides possess sufficient molecular weight and length to aggregate effectively during EtOH precipitation. In contrast, small oligos tend to remain soluble and are often lost in this process. For instance, Jeff C has reported that smaller RNA fragments lost more during EtOH precipitation, whereas most of them were recovered using ZipTip[43].

ZipTip minimizes sample loss. The small volume (8~10 μL) of the ZipTip allows efficient desalting with minimal sample requirements. In contrast, high-performance liquid chromatography–mass spectrometry (HPLC-MS) typically requires a larger sample volume (50~200 μL) and often results in greater sample loss, especially for low-abundance species, making peak detection and analysis difficult[45].

The ZipTip desalting process produces many unrelated mass peaks of ssDNA fragments. The observed main peaks in the MS spectrum, their possible molecular structure, and the source are summarized in the Supplementary Tables 5,6.

4.2 Hydrolytic or oxidative cleavage fragments analysis

Fluorescence-labelled reporter assays indicate that the DNA cleavage involves scission of the phosphate diester backbone. However, it is important to note that both hydrolytic and oxidative cleavage mechanisms ultimately lead to phosphate diester bond breakage. In oxidative cleavage, the initial step usually involves hydrogen abstraction from the sugar moiety. This triggers ring-opening of the deoxyribose, leading to structural destabilization and eventual cleavage of the adjacent phosphate diester bonds[46]. Therefore, analysis of the cleavage products and their dynamic abundance trends in 5T-ssDNA treated with Ga droplets is crucial to discerning whether the reaction proceeds *via* a hydrolytic or oxidative pathway. Samples treated with Ga droplets for 1.5 h and 4 h were examined to characterize the resulting fragments.

The structures of the expected hydrolytic cleavage products are illustrated in the Supplementary Fig. 24, and their theoretical and observed mass-to-charge (m/z) values across different charge states are summarized in Supplementary Tables 7-10.

Potential oxidative cleavage fragments were also considered based on prior systematic studies[47]. Due to their chemical characteristics and potential size, some of these fragments fall outside the detection limits of MS. Accordingly, four representative types of oxidative fragments were defined and searched for (Supplementary Tables 11,12; Supplementary Fig. 25); however, none were detected in the mass spectra of the ssDNA-Ga group.

4.3 Reasons for focusing on 5T and 4T ssDNA and low charge states in hydrolytic cleavage fragments

In the hydrolytic cleavage fragments, we mainly focus on the analysis of 5T and 4T ssDNA and their low charge states. The reasons are listed below.

Quantitative analysis of ion intensities from the mass spectrum reveals changes in the 5T, 4T, 3T, and 2T ssDNA fragments. Specifically, the ion intensities of 5T, 3T, and 2T ssDNA decreased after Ga droplet treatment, whereas that of 4T ssDNA increased. The density functional theory (DFT) calculation further indicated that in 5T ssDNA, Ga preferentially cleaves at the 1T and 4T positions. Detailed discussion is provided in Supplementary Discussion 4.1. Thus, the opposing trends in 5T and 4T ssDNA intensities are attributed to Ga-induced DNA cleavage. In contrast, the decreased signals of 2T and 3T ssDNA are likely attributable to a lower cleavage frequency of short ssDNA (Supplementary Discussion 4.2), combined with greater sample loss during ZipTip desalting with C18 resin. Owing to their lower hydrophobicity compared to 4T ssDNA, these fragments are more prone to elution and subsequent loss. As a result, their MS signal is lower than that of the control group (no ZipTip desalting). Therefore, our analysis focuses mainly on the 5T and 4T ssDNA fragments.

For 5T and 4T ssDNA, we specifically examine the low charge species due to charge state suppression caused by Ga^{3+}. Previous reports[48]show that Ga^{3+} preferentially adsorbs onto the phosphate backbone of DNA, forming stable complexes. It competes with magnesium ions for binding sites, exhibiting an affinity approximately 100 times higher[49]. This strong interaction with DNA appears difficult to remove by ZipTip desalting. The loss of high-charge species caused by ions or salts has also been reported[50], where the spectra of linear DNA strands shift from higher charge states (e.g., -4 to -10) to lower ones (e.g., -4 to -6) as salt concentration increases. This shift is attributed to cation-induced charge suppression, which also leads to a reduction in signal intensity. Therefore, our analysis focuses on 5T and 4T species in their low-charge states.

Lastly, the results of MS analysis do not imply that Ga droplets are incapable of cleaving 4T ssDNA into shorter fragments. Due to current technical limitations, shorter fragments such as 2T and 3T ssDNA are difficult to be detected in MS because of losses during the desalting process, while only 5T ssDNA is commercially available for experimental use.

Supplementary Discussion 5. MD simulation for ssDNA and Ga oxide interaction

To investigate the binding interactions between the ssDNA and Ga_2O_3/GaOOH substrates in different initial orientations, we performed MD simulations with explicit solvation (Fig. 4f and Supplementary Fig.29 d). After equilibration (5 ns *NVT* at 305 K), we analysed the interfacial interactions from the final frame of each trajectory and calculated the corresponding binding energies (Fig. 4f and Supplementary Fig.29d). The water molecules were made invisible for clarity of visualization.

For ssDNA-2T on Ga_2O_3 (Fig.4f), both initial orientations (parallel and perpendicular) converged to parallel configurations, with the phosphate backbone preferentially aligned towards surface O atoms. The binding energies were -222 kcal mol^{-1} and -214 kcal mol^{-1} for the two structures, respectively. In contrast, ssDNA-2G exhibited a stronger affinity to Ga_2O_3, with the initially parallel orientation (Supplementary Fig. 29d) maintaining a V-shaped conformation by changing its torsion angles (binding energy: -290 kcal mol^{-1}), while the initially perpendicular orientation adopted a tilted configuration on the surface (binding energy: -139 kcal mol^{-1}). The binding energy of this structure was weaker due to fewer functional groups interacting with the surface. The enhanced binding of ssDNA-2G compared to ssDNA-2T can be attributed to the greater number of aromatic rings in the guanine bases, which provide stronger van der Waals interactions with the Ga_2O_3 substrate.

For ssDNA interacting with the GaOOH substrate, the binding was notably stronger than with Ga_2O_3. For ssDNA-2T (Fig.4f), both initial orientations converged to parallel configurations with binding energies of −311 kcal mol^{-1} and -341 kcal mol^{-1}. In comparison, ssDNA-2G displayed a stronger affinity for the GaOOH surface than ssDNA-2T, adopting a parallel orientation regardless of its initial

configuration (Supplementary Fig.29d). Both structures had binding energies of -462 kcal mol^{-1} (initially parallel) and -369 kcal mol^{-1} (initially perpendicular). The consistently stronger binding energies for a parallel to the surface indicate greater thermodynamic stability as a result of increased contact area and enhanced interactions due to the type of functional group.

Overall, the MD simulations demonstrate that: (i) ssDNA binds more strongly to GaOOH than Ga_2O_3, and (ii) ssDNA-2G exhibits enhanced substrate affinity compared to ssDNA-2T due to the additional aromatic rings that enhance van der Waals interactions.

Supplementary Discussion 6. DFT calculation for ssDNA and Ga^0 and Ga^{3+} interaction

6.1 Energetic basis for enhanced 4T fragment yield in MS

The relative energies for cleavage of 5T ssDNA by a Ga atom with ·OH were calculated following the reaction steps (Method 9). For Step 1 (adsorption of Ga), the results indicate that the DNA5T-1 and DNA5T-4 sites have stronger adsorption energies (-1.64 eV and -2.38 eV, respectively) compared to the DNA5T-2 and DNA5T-3 sites (-1.52 eV and -1.09 eV, respectively), indicating that Ga prefers to adsorb to the terminal sites of ssDNA. For Step 2 ·OH cleavage, the calculations indicate that ·OH cleavage is more energetically favourable when Ga adsorbs to the DNA5T-2 and DNA5T-3 sites, with reaction energies of -6.87 eV and -8.51 eV, respectively, compared to -3.63 eV and -6.58 eV for Ga adsorbed to the DNA5T-1 and DNA5T-4 sites, respectively. These results suggest that ·OH preferentially attacks ssDNA at the DNA5T-2 and DNA5T-3 sites when Ga is bonded to these respective sites. For Step 3 (removal of Ga), the calculations indicate that the removal of Ga from all ssDNA fragments requires overcoming significant energy barriers. Ga desorption from the DNA5T-4 fragment requires the highest energy (8.77 eV), while the energy barriers for the other fragments are comparable (DNA5T-1: 4.56 eV, DNA5T-2: 4.30 eV, DNA5T-3: 4.16 eV).

From an energetic perspective, ssDNA cleavage most likely occurs at the DNA5T-1 site. Since Step 2 (·OH cleavage) is the least energy-demanding, the rate-determining steps are Step 1 (adsorption of Ga) and Step 3 (desorption of Ga). The DNA5T-1 site provides an optimal balance between a moderate adsorption energy (-1.64 eV) and a manageable desorption barrier (4.56 eV), ensuring stable complex formation and efficient catalyst regeneration. Other sites exhibit either too weak adsorption (DNA5T-3: -1.09 eV) or excessive adsorption with high desorption barriers (DNA5T-4: -2.38 eV, 8.77 eV), preventing effective catalytic turnover. This energetic profile facilitates the complete catalytic cycle, yielding DNA-4T and DNA-1T fragments.

6.2 Comparison of short and long ssDNA cleavage by Ga

The reaction energies between short (2T) and long (5T) ssDNA sequences were compared. The results indicate that DNA2T exhibits a similar Ga adsorption energy (-1.34 eV) to DNA5T-1 (-1.64 eV), as both involve coordination to the terminal phosphate site of ssDNA. Similarly, DNA2T shows comparable cleavage energy (-3.30 eV) to DNA5T-1 (-3.63 eV), since both involve cleavage of a single nucleotide unit. However, these cleavage energies are significantly less exothermic than those of DNA5T-2 (-6.87 eV), DNA5T-3 (-8.51 eV), and DNA5T-4 (-6.58 eV), indicating that ·OH-mediated cleavage is thermodynamically less favourable for shorter ssDNA chains. For Step 3, desorption of Ga from DNA2T requires moderate energy (4.39 eV), which is smaller than that of DNA5T-1 (4.56 eV) but higher than that of DNA5T-2 (4.30 eV) and DNA5T-3 (4.16 eV). Overall, these results indicate that Ga-induced cleavage of longer ssDNA sequences (5T) is thermodynamically more favourable than that of shorter sequences (2T).

Supplementary Discussion 7. Possible mechanism of Ga droplets' preference to T-T and A-A

The adsorption behavior of ssDNA on Ga droplets was further examined using competitive inhibition by thymidine and phosphate ions. Previous studies have established that inhibition by free nucleosides (e.g., thymidine) indicates adsorption through sugar or nucleobase moieties[51], whereas inhibition by phosphate ions implies interaction with the phosphodiester backbone[52]. The results show that Ga droplets' DNA cleavage activity was selectively suppressed by phosphate ions but not by thymidine (Supplementary Fig.29 a, b). Combined with MD simulation results showing that ssDNA adopts a largely flat adsorption configuration on Ga oxide layer and DFT results indicating preferential binding of the phosphodiester group to Ga^0, these findings indicate that the ssDNA molecule adsorbs as a whole onto the Ga surface, while interactions involving the phosphodiester backbone play the dominant role in the subsequent cleavage.

To clarify the relationship between adsorption and the observed cleavage preference toward 5T vs 5G sequences, quantitative adsorption experiments were performed (Supplementary Method 8,9). The results show that GaOOH exhibits stronger ssDNA adsorption than Ga_2O_3, and that both oxide surfaces display higher affinity for 5G ssDNA compared to 5T ssDNA (Supplementary Fig.29c). Consistently, classical molecular dynamics (MD) simulations reveal that (i) ssDNA binds more strongly to GaOOH than to Ga_2O_3, and (ii) 2G ssDNA exhibits higher binding affinity than 2T ssDNA, attributable to additional aromatic rings that enhance van der Waals interactions (Fig.4f; Supplementary Fig.29d, e). In the DFT calculations, 5T and 5G exhibit comparable adsorption strengths to the Ga^0 atom, with a slight preference for 5T (Supplementary Fig. 27 b,c).

To exclude the possibility that reduced cleavage of 5G arises from overly strong adsorption leading to slow desorption and blockage of reactive sites on Ga droplets, we conducted cleavage experiments using excess Ga droplets to provide abundant reactive interfaces, followed by PBS addition to promote ssDNA desorption. Under these conditions, Ga droplets still exhibited a clear cleavage preference for T-rich sequences over G-rich sequences (Supplementary Fig.29f). Moreover, when normalized to the activity of the commercial nuclease S1, Ga droplets retained a stronger relative cleavage efficiency toward 5T ssDNA over 5G ssDNA (Supplementary Fig.29g). These results indicate that the observed cleavage preference originates from difference in adsorption and binding configuration, rather than from desorption-limited kinetics.

Based on these results, we propose the following mechanism: Both experimental results and MD simulations show that, on Ga oxide surfaces (Ga_2O_3 and GaOOH), 2G ssDNA exhibits stronger adsorption than 2T ssDNA, whereas Ga droplets display lower cleavage efficiency toward 2G.

This suggests that DNA adsorption strength does not correlate linearly with cleavage efficiency. One possible reason is that strong adsorption of 2G induces pronounced conformational distortions, as evidenced by torsional angle changes and distorted structures (Supplementary Fig. 29d). Such structural deformations are likely to impede cleavage by altering the geometry of the phosphodiester bond, introducing steric barriers to ·OH access, and restricting the conformational flexibility required for efficient electron transfer. In contrast, 2T ssDNA maintains a more native and less distorted conformation, preserving the structural accessibility of the phosphodiester linkage (Fig. 4f) and thereby enabling more efficient cleavage. Another possible reason is the differences in interfacial electron redistribution. Following initial adsorption, Ga^0-2T and Ga^0-2G complexes exhibit different electronic redistribution patterns (Supplementary Fig.27d). In both cases, electrons are transferred from Ga to non-bridging phosphate oxygens; however, Ga^0-2T displays a more moderate and delocalized redistribution, resulting in a less stabilized phosphate environment and increased susceptibility to cleavage.

Supplementary Figures

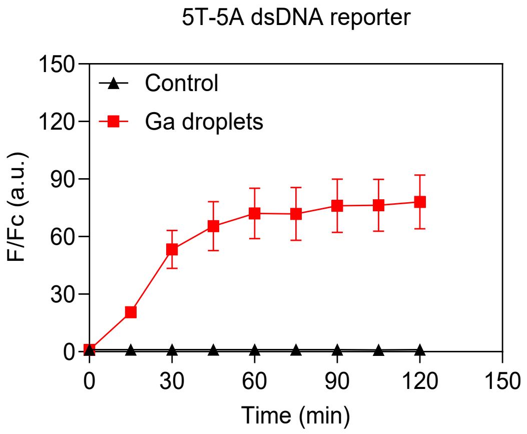

Supplementary Fig. 1. Cleavage of 5T-5A double-stranded deoxyribonucleic acid (dsDNA) reporter by Ga droplets (n = 3 independent reactions). Data are presented as means ± SDs. Source data are provided as a Source Data file.

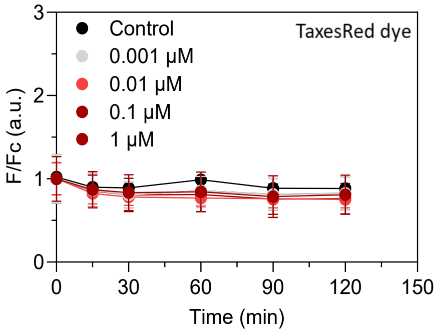

Supplementary Fig. 2. Time-dependent fluorescence of TexasRed dye incubated with Ga droplets to exclude the influence of Ga droplets on the TexasRed dye (n = 4 independent reactions). Data are presented as means ± SDs. Source data are provided as a Source Data file.

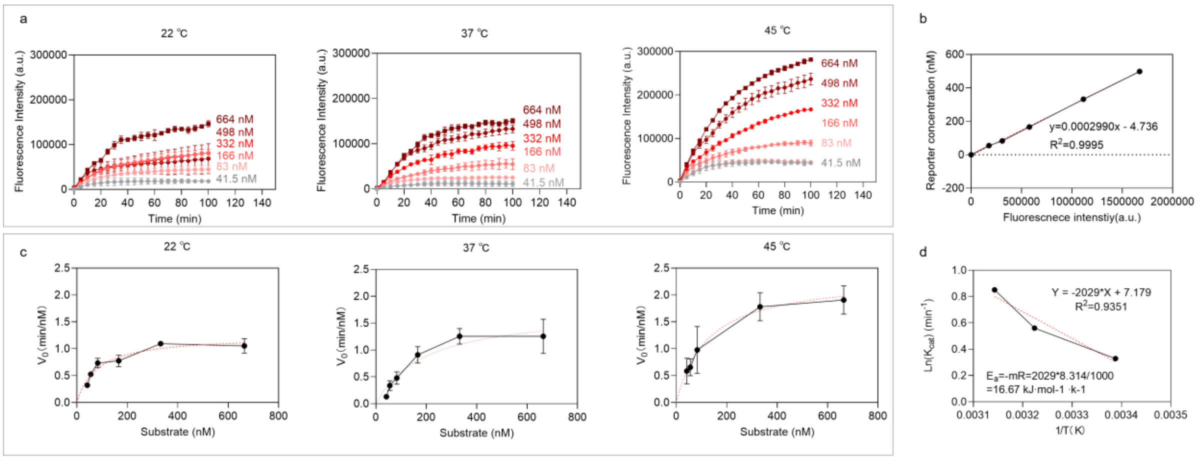

Supplementary Fig. 3. (a) Kinetic performance of Ga droplets at 22 °C (n = 4 independent reactions), 37 °C (n = 4 independent reactions), and 45 °C (n = 3 independent reactions), shown as time-dependent fluorescence increases of TR-ssDNA reporters at increasing substrate concentrations.(b) Relationship between fluorescence intensity and TR-ssDNA reporter concentration, with excess S1 nuclease used to define the maximal fluorescence corresponding to complete substrate cleavage. (c) v_0 extracted from the early linear regions of the progress curves and fitted using the Michaelis–Menten model to obtain apparent kinetic parameters $V_{\max}$ and K_m (n = 4 independent reactions). (d) Arrhenius plots derived from temperature-dependent kinetic parameters, used to calculate the apparent activation energy of Ga droplet–mediated ssDNA cleavage. Data are presented as means ± SDs. Source data are provided as a Source Data file.

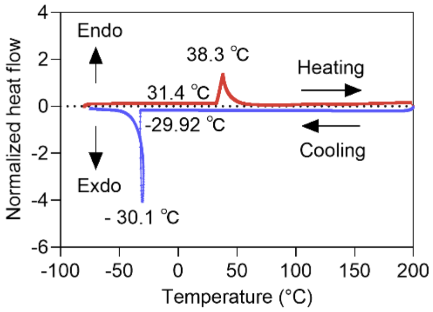

Supplementary Fig. 4. DSC analysis of bulk Ga shows that they exhibit significant supercooling, with a freezing point around -30.1 °C, allowing them to remain in the liquid state at room temperature. Source data are provided as a Source Data file.

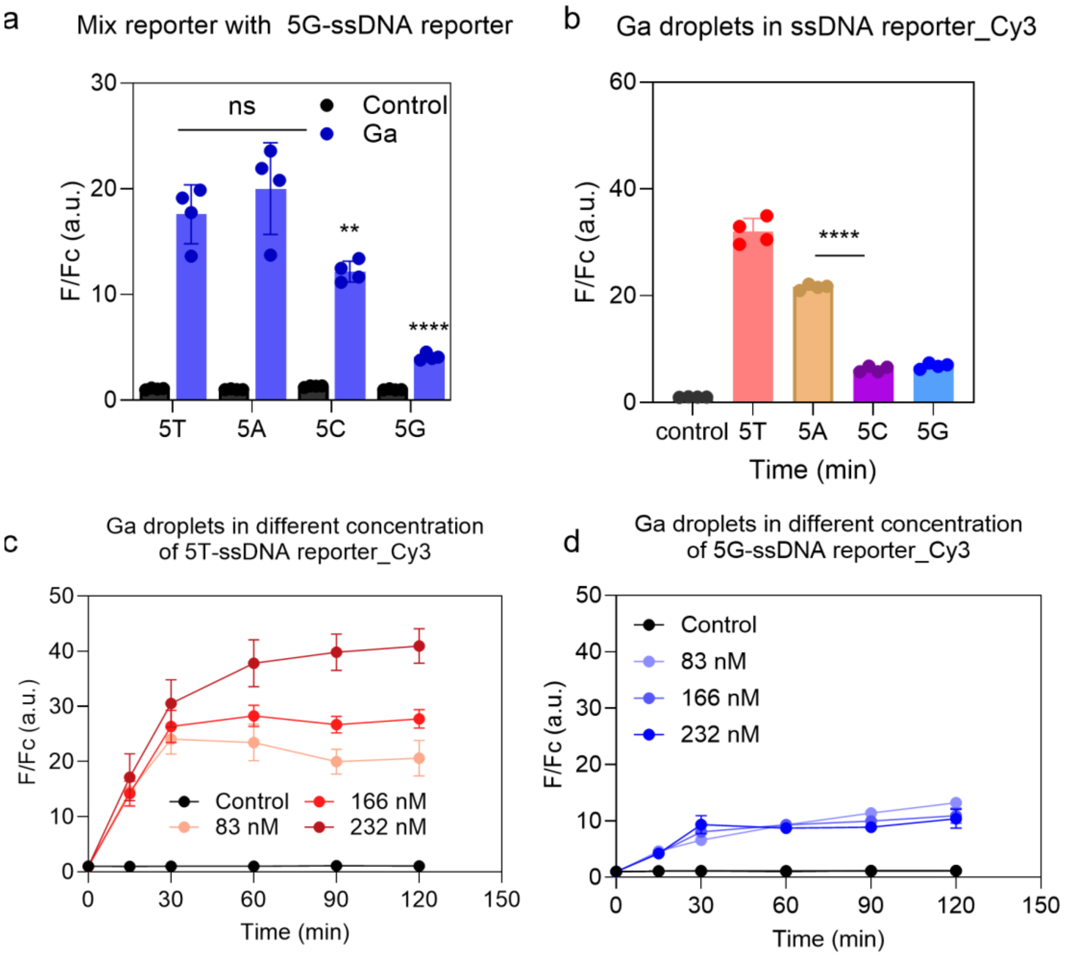

Supplementary Fig. 5. Experiment demonstrating that Ga droplets' nucleotide preference is not caused by quenching artifacts from the 5G-ssDNA reporter solution, guanine-induced fluorophore quenching, or fluorophore dependency. (a) Ga droplets cleavage of TR-ssDNA reporter mixed with 5G ssDNA reporter ($P_{(5A\text{-}5T)} > 0.05$, $P_{(5A\text{-}5C)} = 0.0057$, $P_{(5A\text{-}5G)} < 0.0001$), excluding the possibility that the observed cleavage preference is due to quenching factors in 5G-reporter. Validation of Ga nucleotide preference independent of fluorophore. G nucleobases with the lowest oxidation potential of G (1.40V) < A (1.75 V) < C (2.18 V) ≈ T (2.0 V) can potentially quench adjacent fluorophores. However, Cy3 has been reported to be minimally affected by neighbouring G-induced quenching, typically showing only 10% fluorescence reduction; thus, the Cy3-labeled reporter was chosen for the following experiment: (b) Ga droplets cleavage of Cy3-labeled reporter ($P_{(5A\text{-}5C)} < 0.0001$). Ga droplets nuclease activity comparison between (c) 5T- and (d) 5G-ssDNA reporter. The signal reduction of the 5G-ssDNA Cy3 reporter compared to the 5T-ssDNA Cy3 reporter was approximately 75%, indicating that the observed preferred cleavage arises from true nucleotide preference rather than G-induced quenching or fluorophore type. n = 4 independent reactions; $p < 0.05$ is considered as statistically significant. *$p < 0.05$, **$p < 0.01$, ***$p < 0.001$, and ****$p < 0.0001$. Data are presented as means ± SDs. Source data are provided as a Source Data file.

Co-culture with Ga droplets for different time

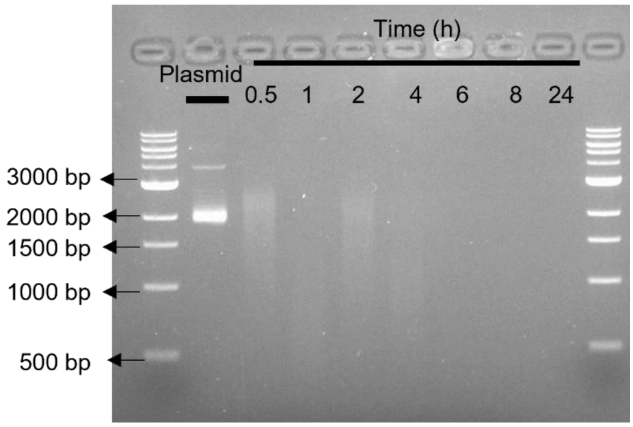

Supplementary Fig. 6. Agarose gel electrophoresis of pUC19 plasmid (2696 bp) incubated with Ga droplets over time without adding Na_2HPO_4 to desorb the fragmented DNA from the Ga droplets' surface. Original uncropped gel is provided as a Source Data file.

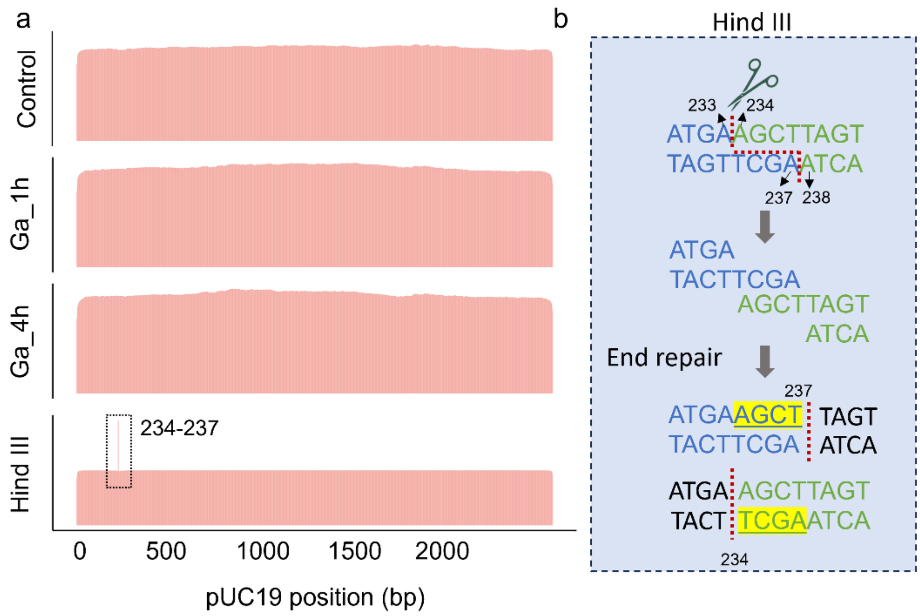

Supplementary Fig. 7. (a) Coverage depth profiles of DNA samples analysed *via* ONT sequencing, including untreated control pUC19 (2696 bp), pUC19 incubated with Ga droplets for 1 hour, pUC19 incubated with Ga for 4 hours, and pUC19 digested with HindIII. (b) Schematic illustration of the influence of end-repair during library preparation on cleavage site resolution in ONT sequencing. Therefore, in the HindIII-digested pUC19 sample, the read coverage exhibits an artificially elevated signal, approximately twofold higher, across the end-repaired region (positions 234–237). This signal inflation results from the presence of duplicated terminal sequences (e.g., AGCT and TCGA) introduced during blunt-end repair of 5′ overhangs. The results of the positive control confirm that the coverage analysis methodology is correct and accurate. For detailed discussion, please refer to Supplementary Discussion 2. The scissors used in the figure are created in BioRender. Liu, L. (2026) https://BioRender.com/8s6hpu0. Figures are plotted by code directly, which is provided in the https://codeocean.com/capsule/3229876/tree.

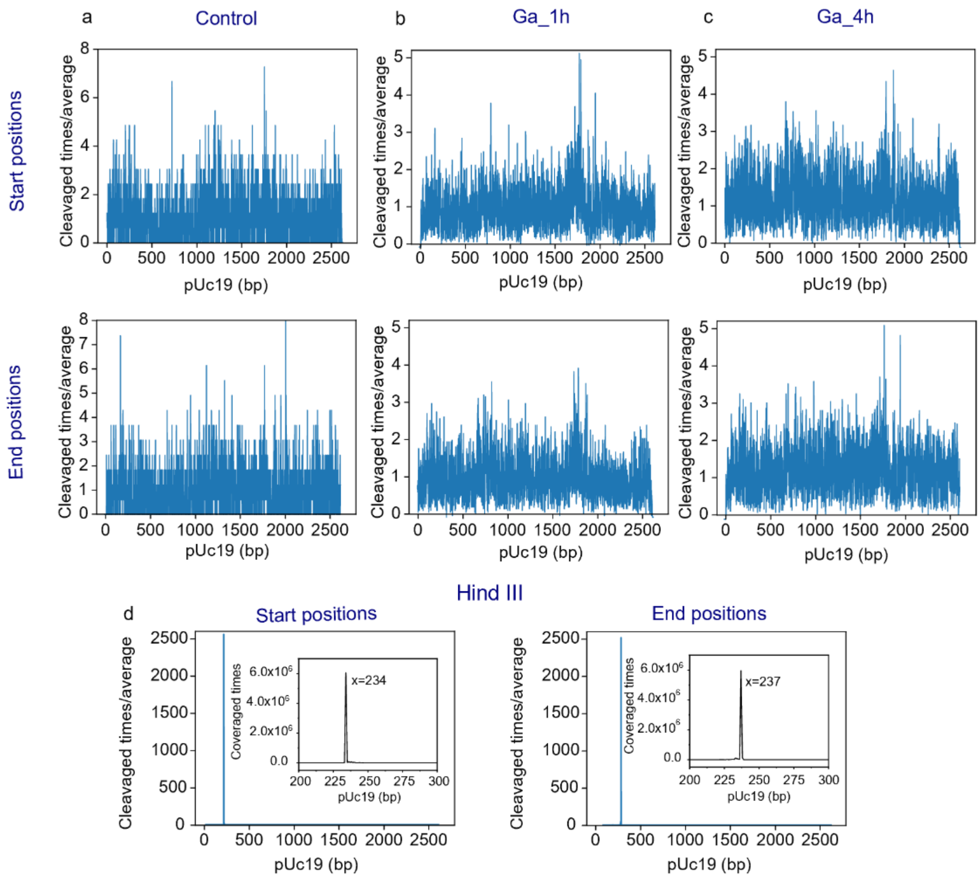

Supplementary Fig. 8. Start and end positions of sequenced DNA fragments from (a) untreated control (pUC19), (b) pUC19 treated with Ga for 1 hour, (c) pUC19 treated with Ga droplets for 4 hours, and (d) pUC19 digested with HindIII. The results show that Ga droplets do not exhibit sequence specificity, while the positive control (HindIII digestion) shows clear specificity at positions 234 and 237. Theoretically, HindIII recognizes and cleaves a specific site in each DNA strand (as shown in Supplementary 7b), resulting in two start sites (positions 234 and 238) and two end sites (positions 233 and 237). However, due to end-repair of the overhangs during library preparation, the sequencing analysis detects only one start site (position 234) and one end site (position 237) for the HindIII-digested DNA fragments. The results of the positive control are consistent with theoretical expectations, confirming that the sequence specificity analysis methodology is accurate and reliable. For detailed discussion, please refer to Supplementary Discussion 2. Figures are plotted by code directly, which is provided in the https://codeocean.com/capsule/3229876/tree.

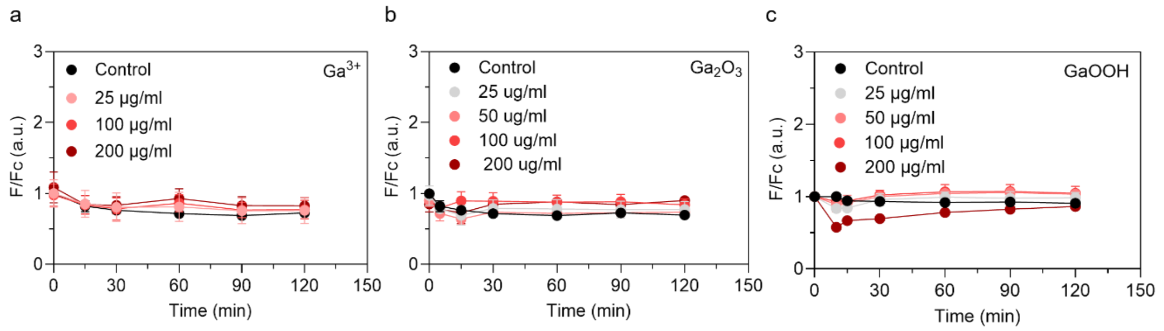

Supplementary Fig. 9. Incubation of TR-ssDNA reporter with (a) Ga^{3+} (Ga $(NO_3)_3$ solution), (b) Ga_2O_3, and (c) GaOOH, all showing negligible or no nuclease activity. n = 4 independent reactions. Data are presented as means ± SDs. Source data are provided as a Source Data file.

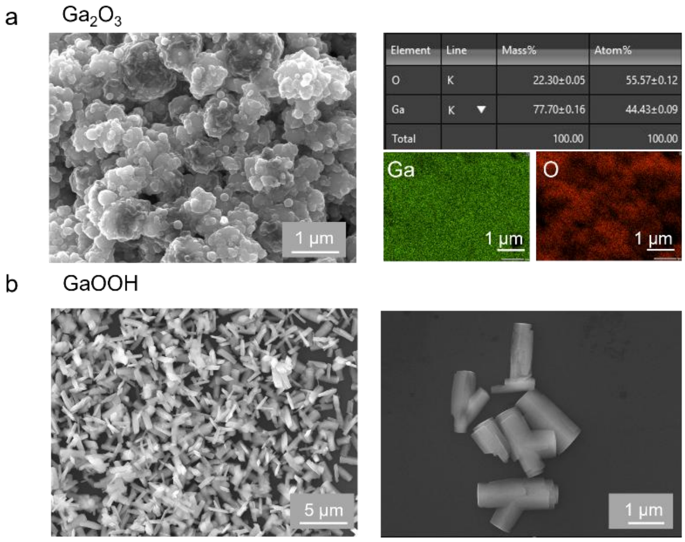

Supplementary Fig. 10. (a) Representative scanning electron microscopy (SEM) image of thermally oxidized Ga_2O_3 particles, quantification of Ga and O elements, and energy-dispersive X-ray spectroscopy (EDS) confirming the Ga_2O_3 composition. (b) Representative SEM image of GaOOH. Each experiment was repeated three times independently with similar results.

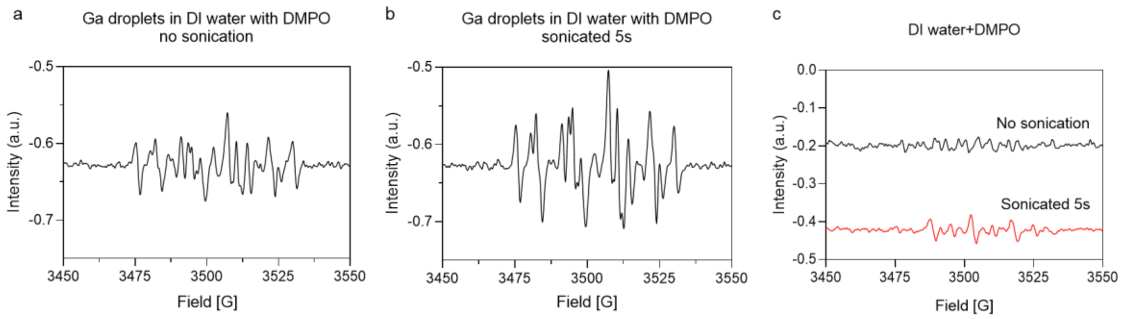

Supplementary Fig. 11. EPR spectra of Ga droplets in DI water in the presence of DMPO: (a) no sonication, (b) sonicated for 5 s. (c) Control spectra of DI water + DMPO without sonication and after 5 s sonication. Dead time = 30s. The triplet signal observed in DI water after 5 s sonication indicates that it originates from sonication-induced partial degradation of DMPO. Each experiment was repeated three times independently with similar results. Source data are provided as a Source Data file.

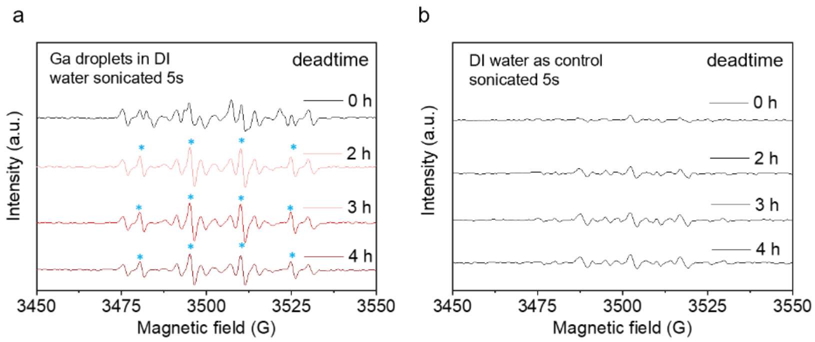

Supplementary Fig. 12. EPR spectra of (a) Ga droplets in DI water with spin trap DMPO, (b) DI water with spin trap DMPO as control; dead time defined as the interval between sample preparation, sealing, equipment setting, and EPR data acquisition: spectra were recorded at different time points following sample preparation. Each experiment was repeated three times independently with similar results. Source data are provided as a Source Data file.

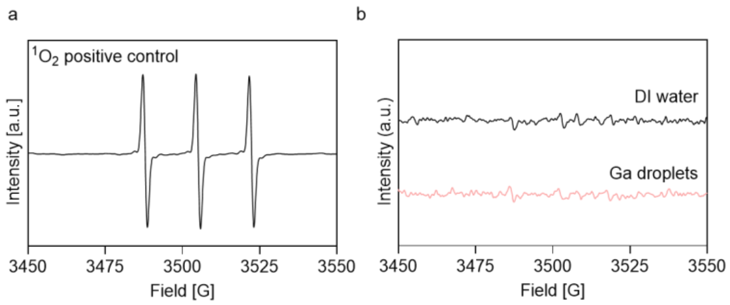

Supplementary Fig. 13 EPR spectra of (**a**) methylene blue (MB) with 2,2,6,6-tetramethylpiperidine (TEMP) under red-light irradiation as a positive control for singlet oxygen generation, and (**b**) DI water and Ga droplets in DI water with TEMP. No detectable signal was observed in the Ga droplet samples, indicating that Ga droplets do not generate singlet oxygen. Each experiment was repeated three times independently with similar results. Source data are provided as a Source Data file.

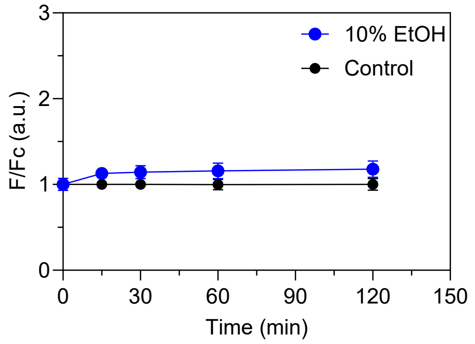

Supplementary Fig. 14. Incubation of TR-ssDNA reporter with 10% EtOH after 5s sonication showing no cleavage effect, indicating the contribution of $CH_3CH_2O\cdot$ is negligible (n = 4 independent reactions). Data are presented as means ± SDs. Source data are provided as a Source Data file.

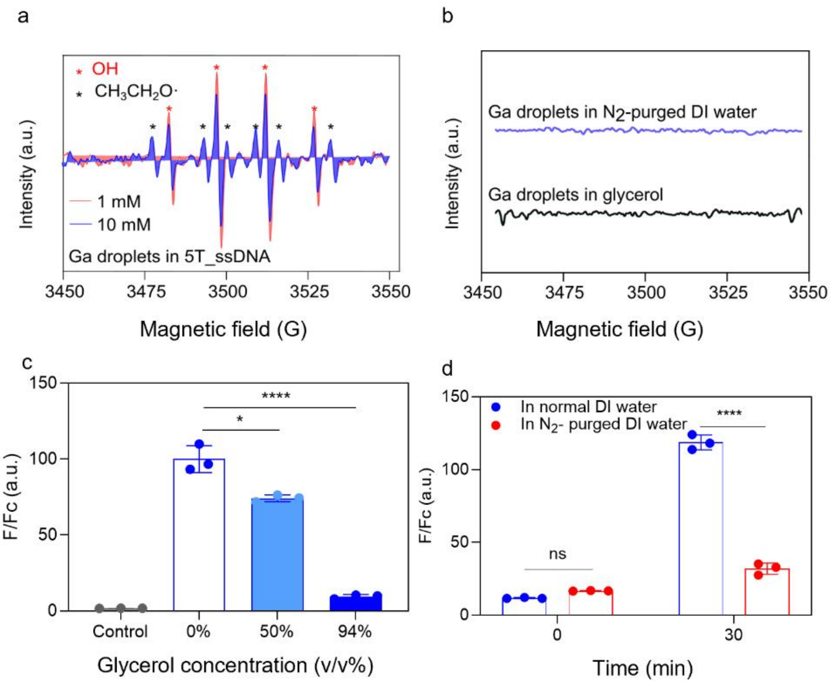

Supplementary Fig. 15. (a) EPR spectra of Ga droplets in 1 mM or 10 mM 5T_ssDNA; deadtime 90 min. (b) EPR spectra of Ga droplets in oxygen-depleted water (nitrogen-purge) and water-depleted (glycerol) condition, showing suppressed radical formation. Each experiment was repeated three times independently with similar results. TR-ssDNA reporters cleavage activity of Ga droplets in (c) water-depleted (glycerol) condition (n = 3 independent reactions) and (d) oxygen-depleted water (nitrogen-purge) water (n = 3 independent reactions). $*p < 0.05$, $**p < 0.01$, $***p < 0.001$, and $****p < 0.0001$. Data are presented as means ± SDs. Source data are provided as a Source Data file.

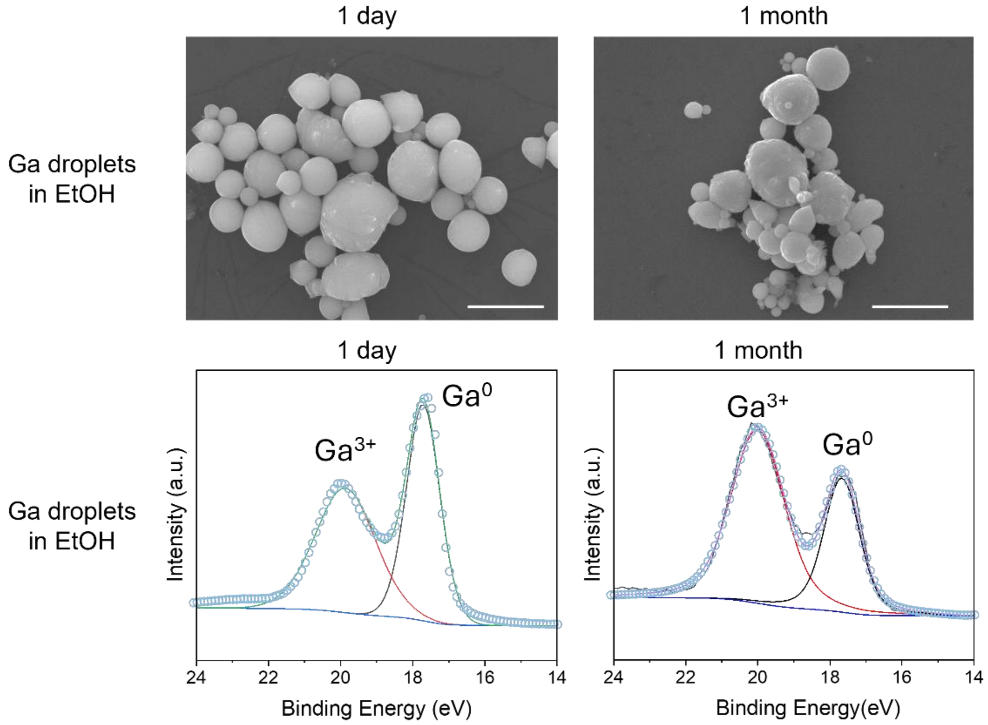

Supplementary Fig. 16. SEM images and Ga 3d XPS spectra of Ga droplets stored in EtOH for one day and one month. SEM reveals negligible morphological evolution during long-term storage. In the Ga 3d region, a dominant doublet centered at 17.8 ± 0.1 eV is assigned to metallic Ga^0, while a higher binding energy peaks at 20.0 ± 0.1 eV corresponds to oxidized Ga^{3+} species, consistent with the presence of a thin surface oxide layer. The relative intensities indicate that metallic Ga^0 still remains the one of the major chemical states after prolonged storage in EtOH, with limited surface oxidation. Each experiment was repeated three times independently with similar results. Source data are provided as a Source Data file.

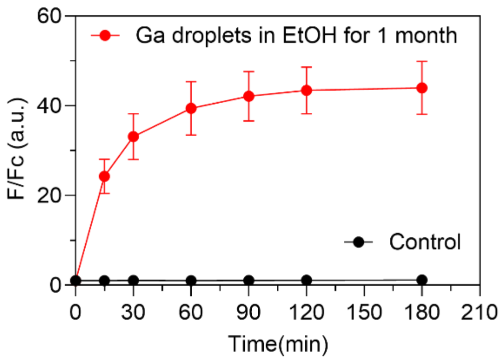

Supplementary Fig. 17. Time-dependent fluorescence of TR-ssDNA reporters incubated with Ga droplets stored in EtOH for one month, showing that long-term stored Ga droplets retain nuclease-mimic activity (n = 4 independent reactions). Data are presented as means ± SDs. Source data are provided as a Source Data file.

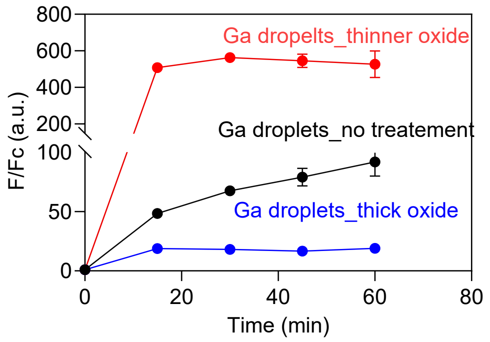

Supplementary Fig. 18. Time-dependent fluorescence of TR–ssDNA reporters incubated with Ga droplets with thin (N_2-purged), thick (air-exposed), or untreated oxide layers (n = 3 independent reactions). Data are presented as means ± SDs. Source data are provided as a Source Data file.

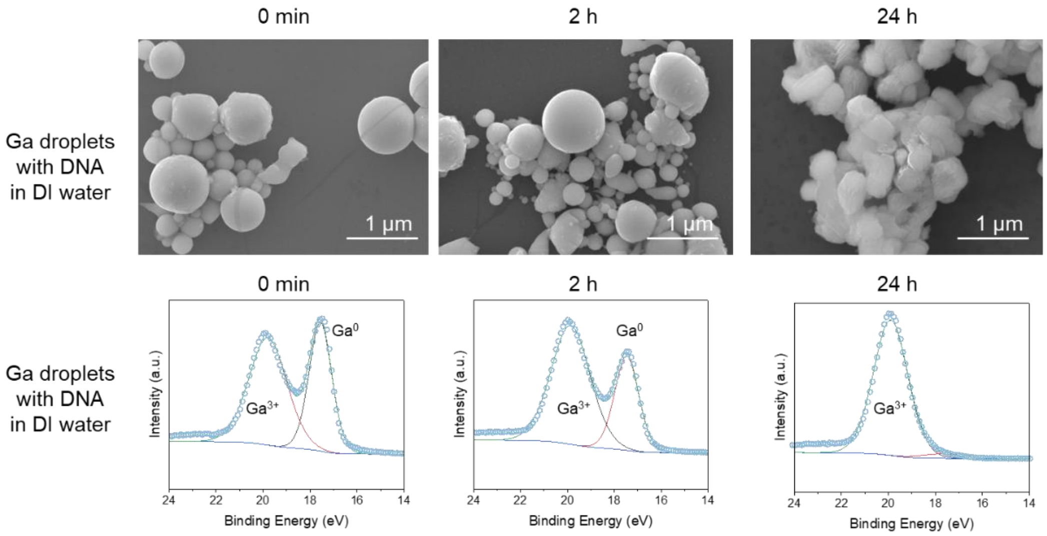

Supplementary Fig. 19. SEM images and Ga 3d XPS spectra of Ga droplets incubated with the TR-ssDNA reporter for 0 min, 2 h, and 24 h; SEM images show a gradual transformation of spherical droplets into rod-like structures; XPS results show a progressive oxidation of metallic Ga^0, with Ga^0 no longer detectable after 24 h. Based on this characteristic morphology and prior reports, the oxidized phase is assigned to GaOOH[11, 36, 37]. Each experiment was repeated three times independently with similar results. Source data are provided as a Source Data file.

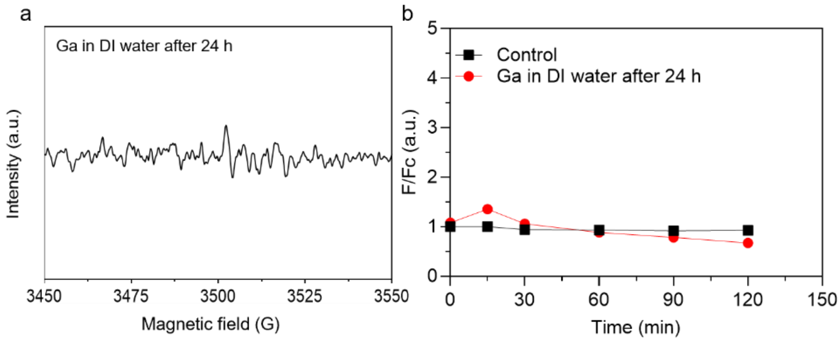

Supplementary Fig. 20. (a) EPR spectra of Ga droplets in water after 24 h. (b) ssDNA reporters' cleavage activity of Ga droplets in DI water after 24h then incubated together with TR-ssDNA reporter (n = 4 independent reactions). Data are presented as means ± SDs. Source data are provided as a Source Data file.

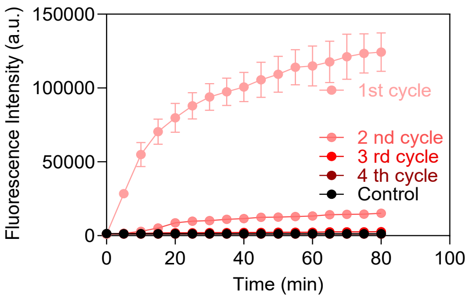

Supplementary Fig. 21. The cleavage effect of Ga droplets on TR-ssDNA reporters after reusing (n = 3 independent reactions). Data are presented as means ± SDs. Source data are provided as a Source Data file.

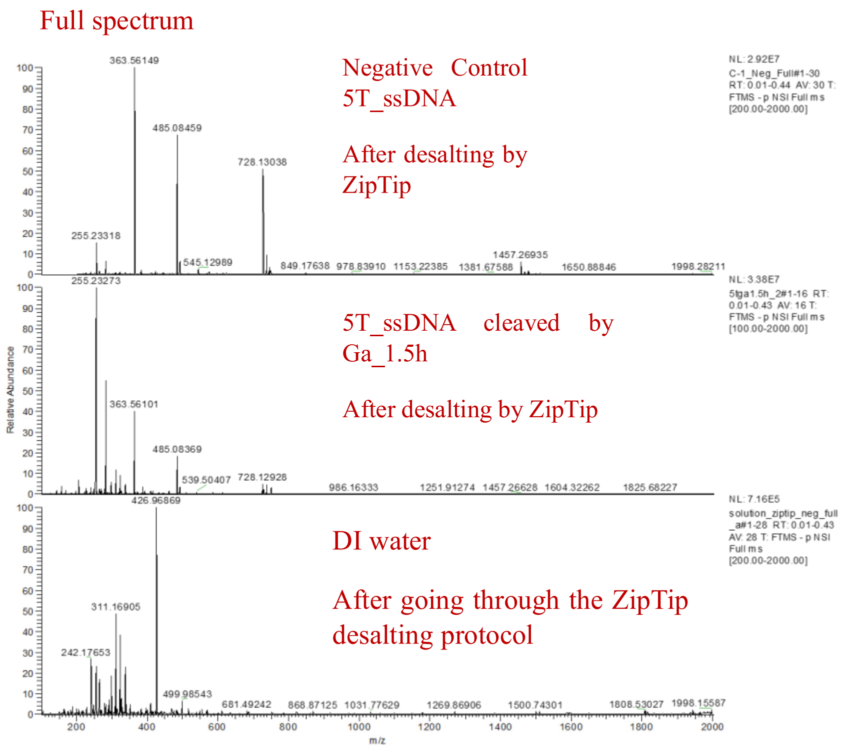

Supplementary Fig. 22. Full MS spectrum of negative control 5T ssDNA, 5T ssDNA cleaved by Ga_1.5h and DI water. All mass spectrometry data supporting the findings of this study have been deposited in Zenodo and are available at the following URL: https://doi.org/10.5281/zenodo.19041335.

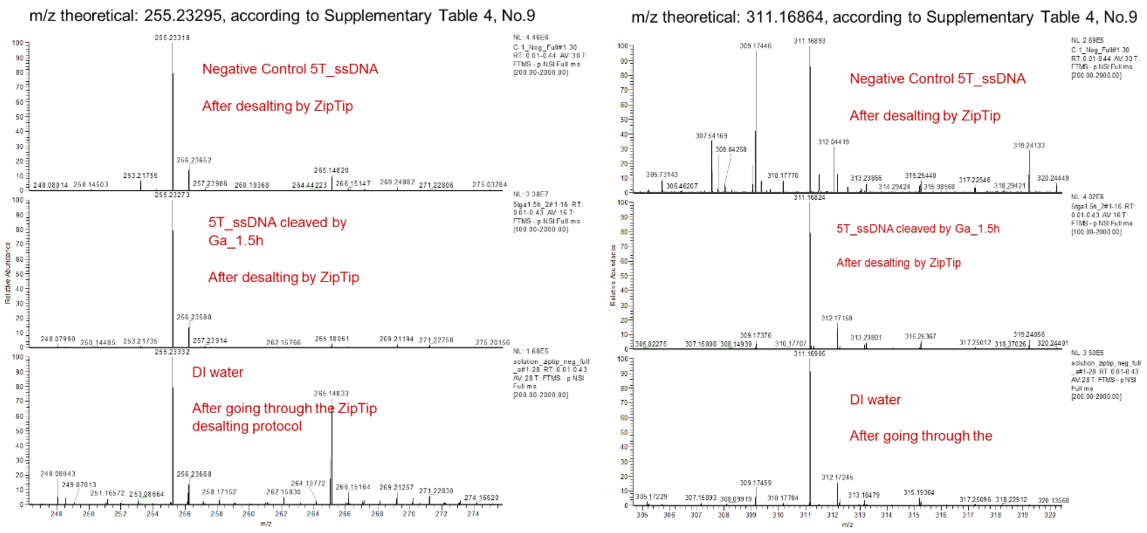

Supplementary Fig. 23. Enlarged MS spectrum of negative control 5T ssDNA, 5T ssDNA cleaved by Ga_1.5h and DI water, all went through the identical ZipTip protocol. The same fatty acid-like peaks were also observed in negative controls (Negative control 5T_ssDNA and only DI water processed through the identical and standard ZipTip protocol), indicating that these signals originate from the desalting workflow. All mass spectrometry data supporting the findings of this study have been deposited in Zenodo and are available at the following URL: https://doi.org/10.5281/zenodo.19041335.

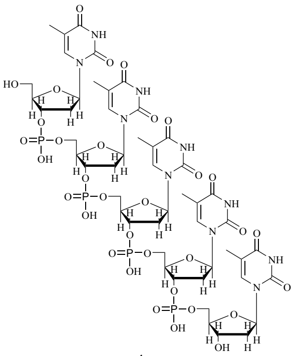

1

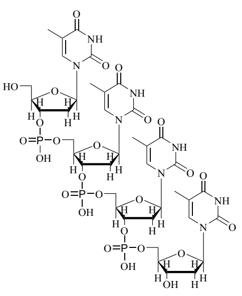

2

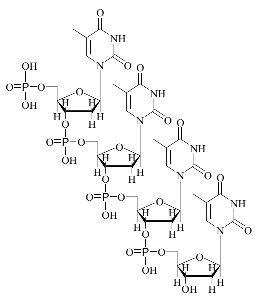

3

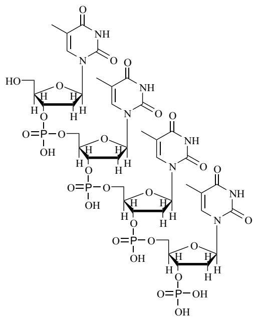

4

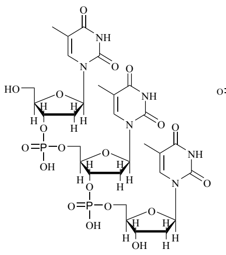

5

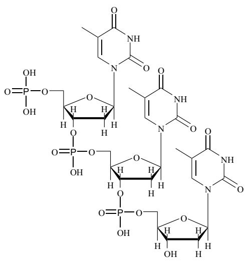

6

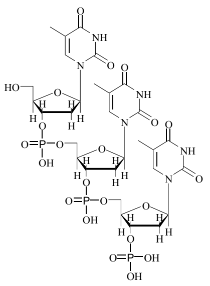

7

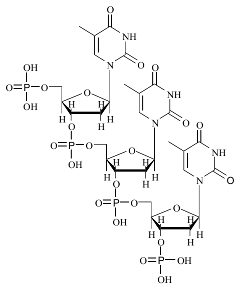

8

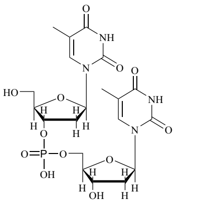

9

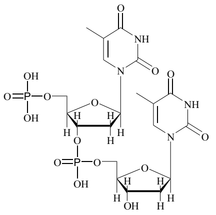

10

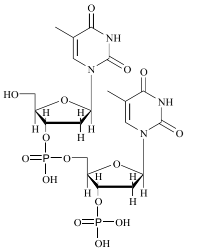

11

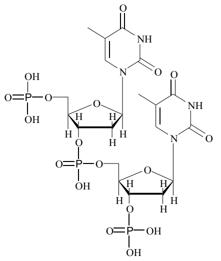

12

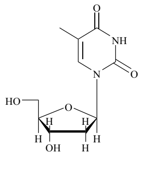

13

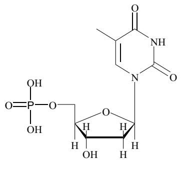

14

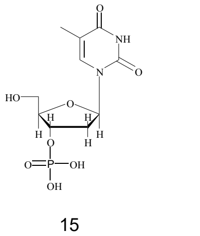

15

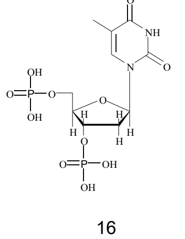

16

Supplementary Fig. 24. Structure of hydrolytic cleavage fragments.

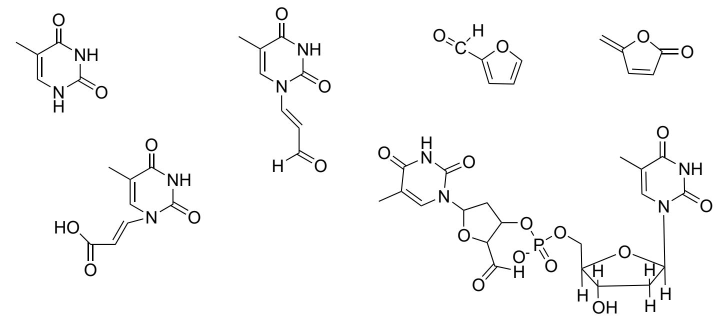

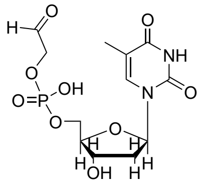

1

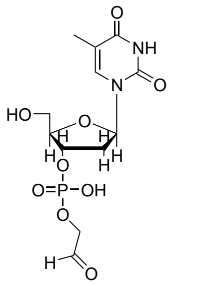

2

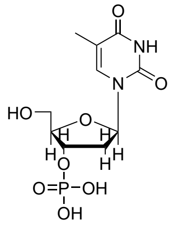

3

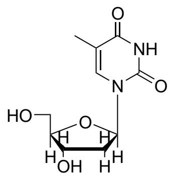

4

Supplementary Fig. 25. Structure of oxidative cleavage fragments.

Product 1 ↓:

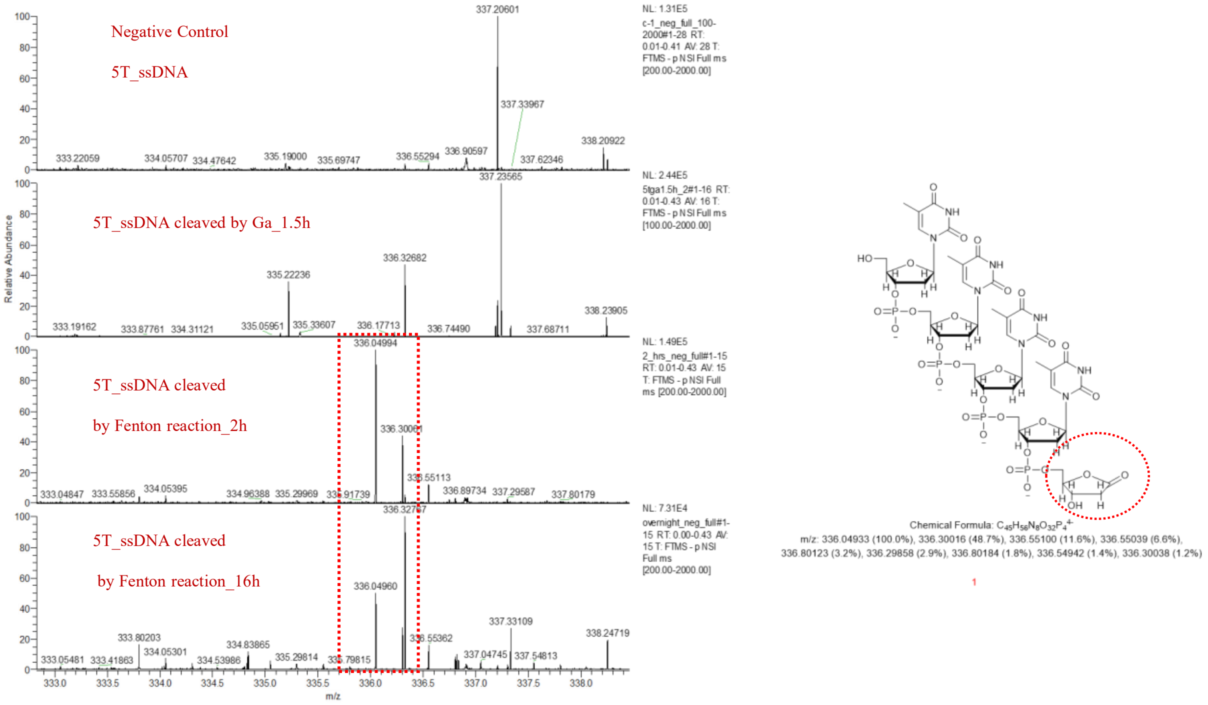

1

Product 2 ↓:

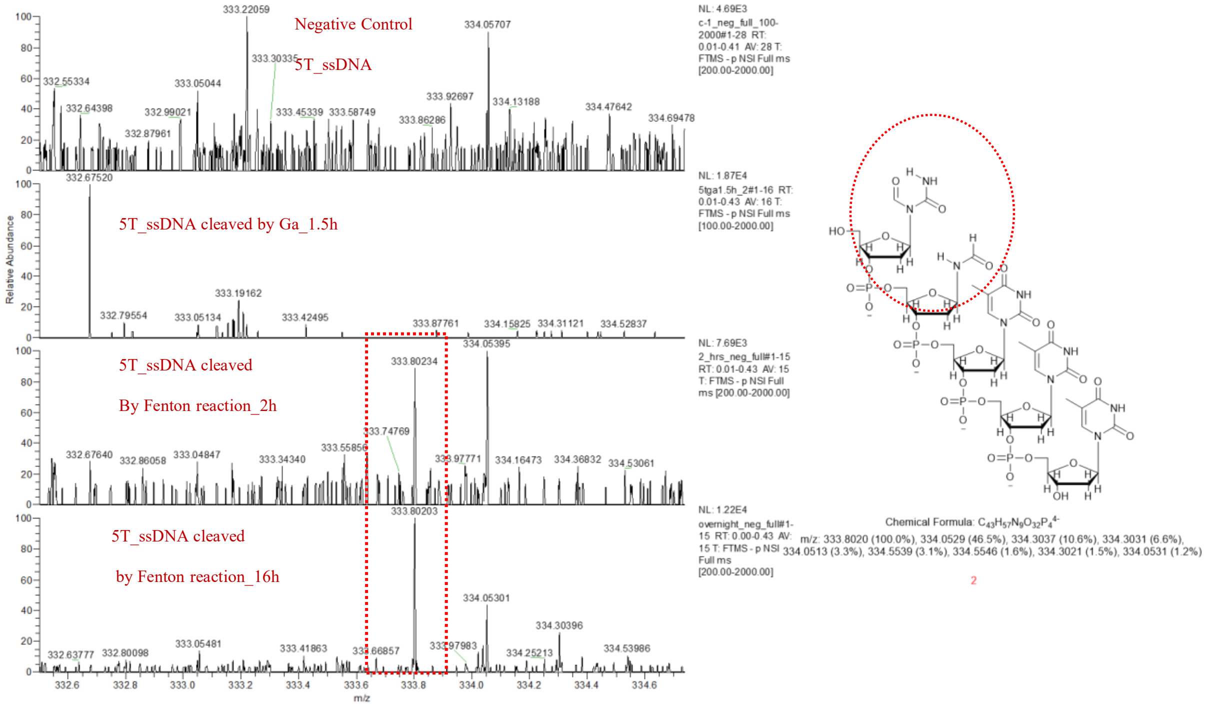

2

Product 3 ↓ :

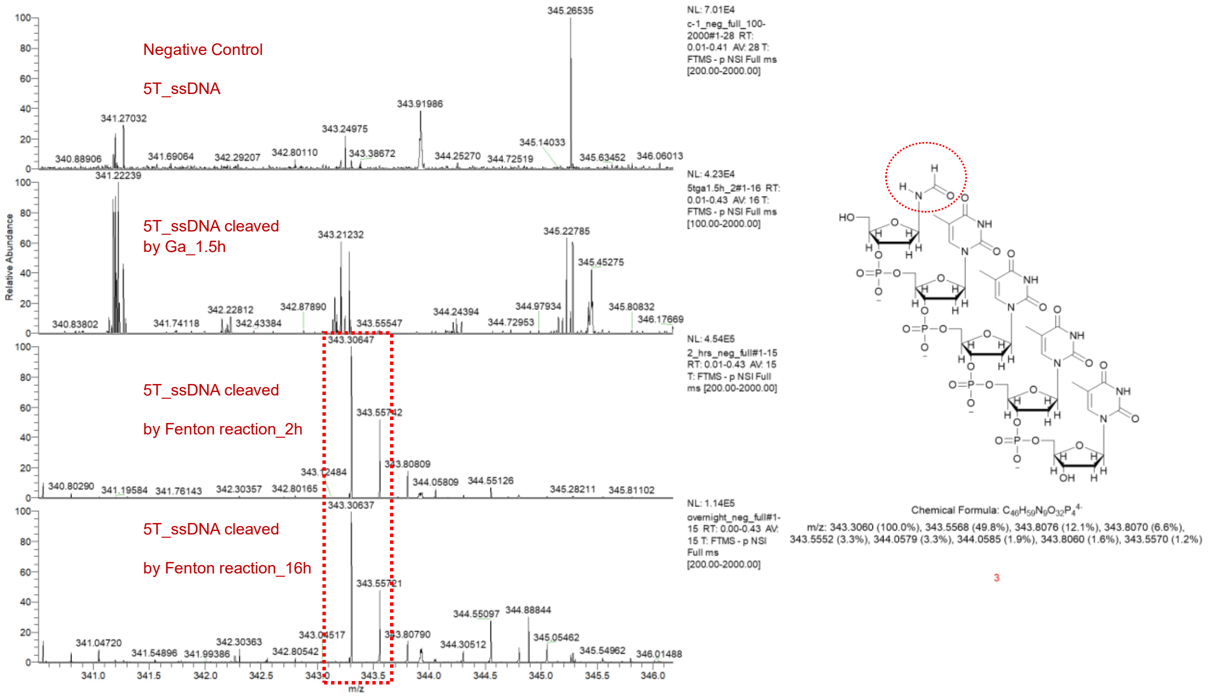

Product 4 ↓ :

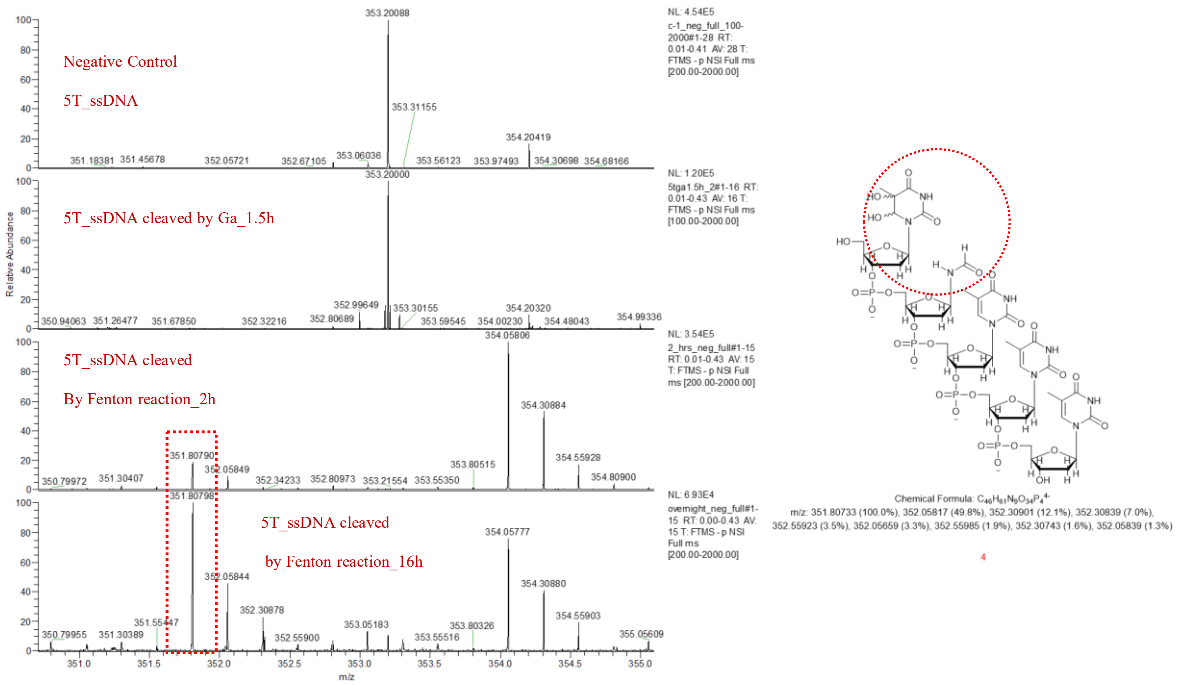

Product 5 ↓:

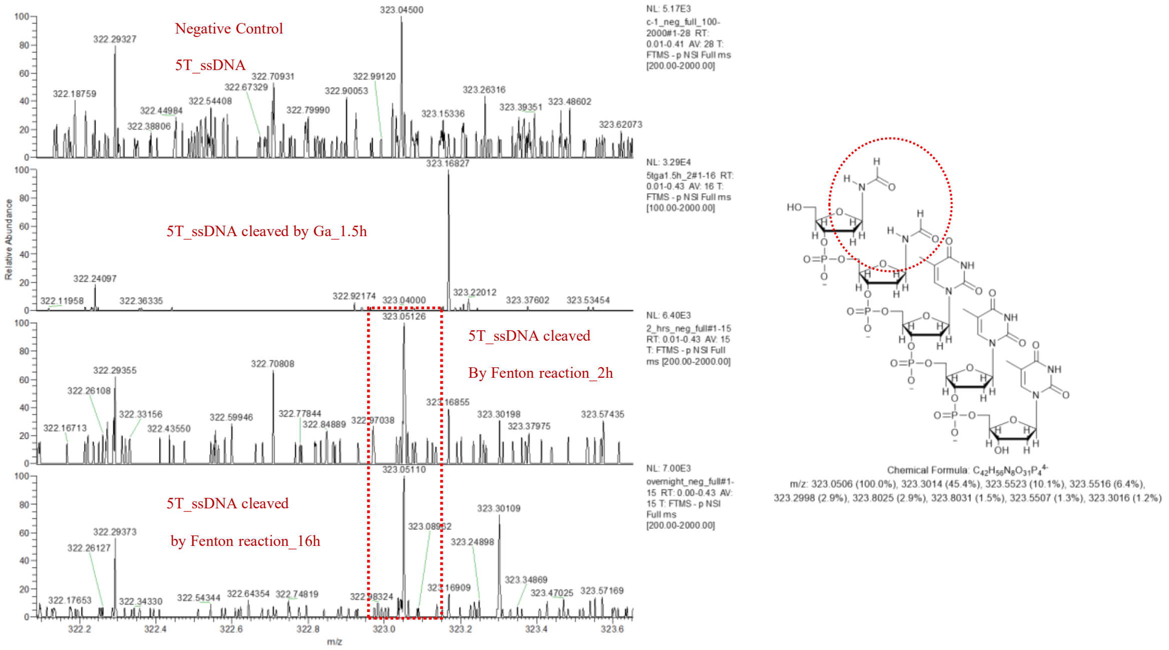

Supplementary Fig. 26. The mass spectra of oxidative fragments derived from 5T ssDNA, observed only under Fenton-mediated cleavage conditions, together with their proposed structures, are shown; the positions of fragmented nucleobases may vary within the 5T sequences. All mass spectrometry data supporting the findings of this study have been deposited in Zenodo and are available at the following URL: https://doi.org/10.5281/zenodo.19041335.

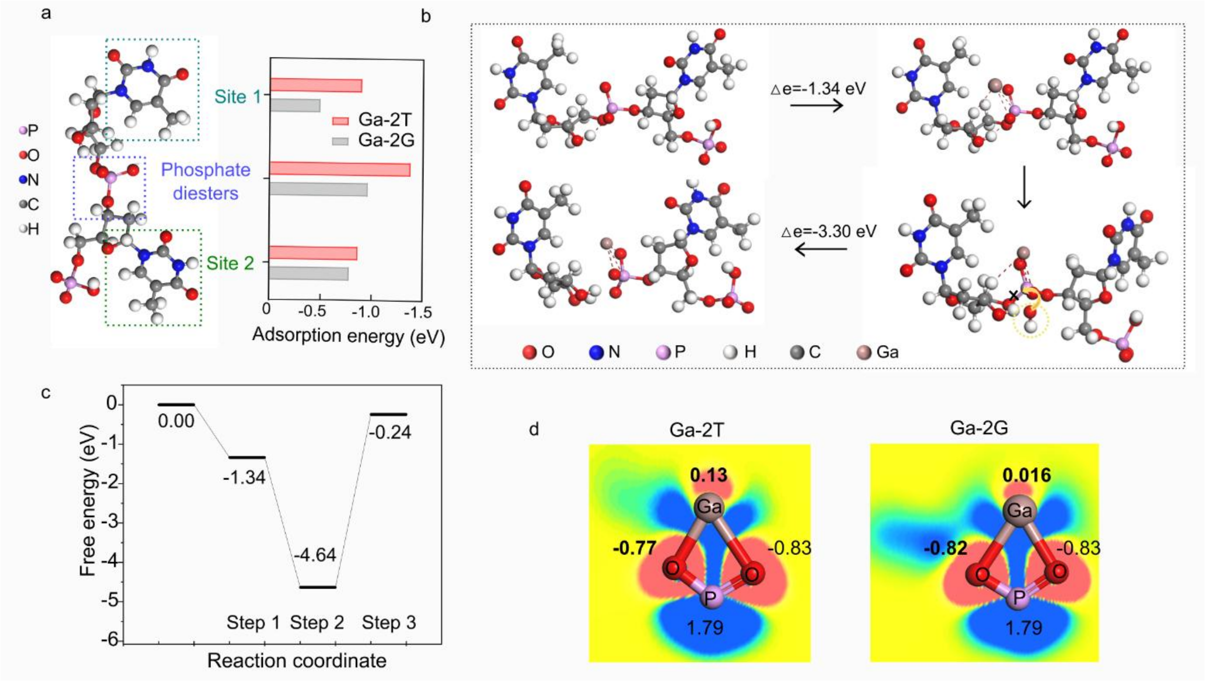

Supplementary Fig. 27. (a) Comparison of adsorption energies between Ga and 2T or 2G ssDNA at three representative adsorption sites: phosphate diester bond, base site 1 (near 3'-OH), base site 2 (near 5'-phosphate). (b) DFT-calculated reaction pathway, including Ga adsorption, ·OH cleavage, and Ga desorption from 2T ssDNA. (Supplementary Method 7) (c) Calculated reaction energy diagram of 2T ssDNA showing the energy profile for Ga^0-induced cleavage of the phosphate diester bond. (d) Charge density difference plot showing the Ga coordinated with the oxygen atoms around the phosphate diester bond of 2T *vs* 2G.

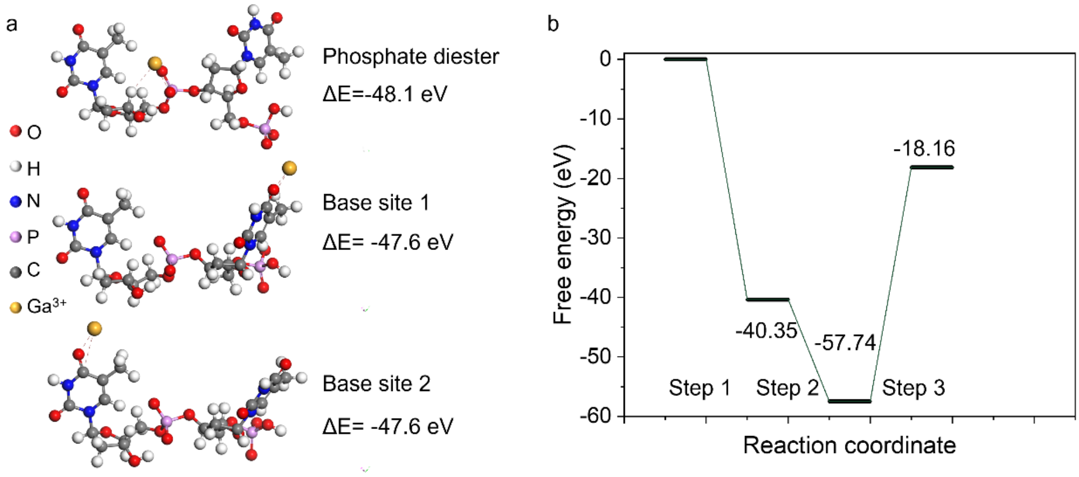

Supplementary Fig. 28. (a) Calculated adsorption energies of Ga^{3+} on 2T and 2G ssDNA at three representative binding sites: the phosphate diester group, base site 1 (proximal to the 3′-OH), and base site 2 (proximal to the 5′-phosphate). Ga^{3+} exhibits strong electrostatic coordination to the phosphate moiety relative to nucleobase sites for both sequences. (b) Computed reaction energy profile for the Ga^{3+}–2T ssDNA complex in the presence of ·OH (Supplementary Method 7). The Ga^{3+}-phosphate interaction excessively stabilizes the post-reaction intermediate, resulting in a prohibitively high desorption barrier for product release ($\Delta E \approx 39.58$ eV for step 3). This large energetic penalty renders Ga^{3+}-assisted phosphodiester cleavage kinetically inaccessible, indicating that Ga^{3+} does not function as an effective center for DNA backbone scission.

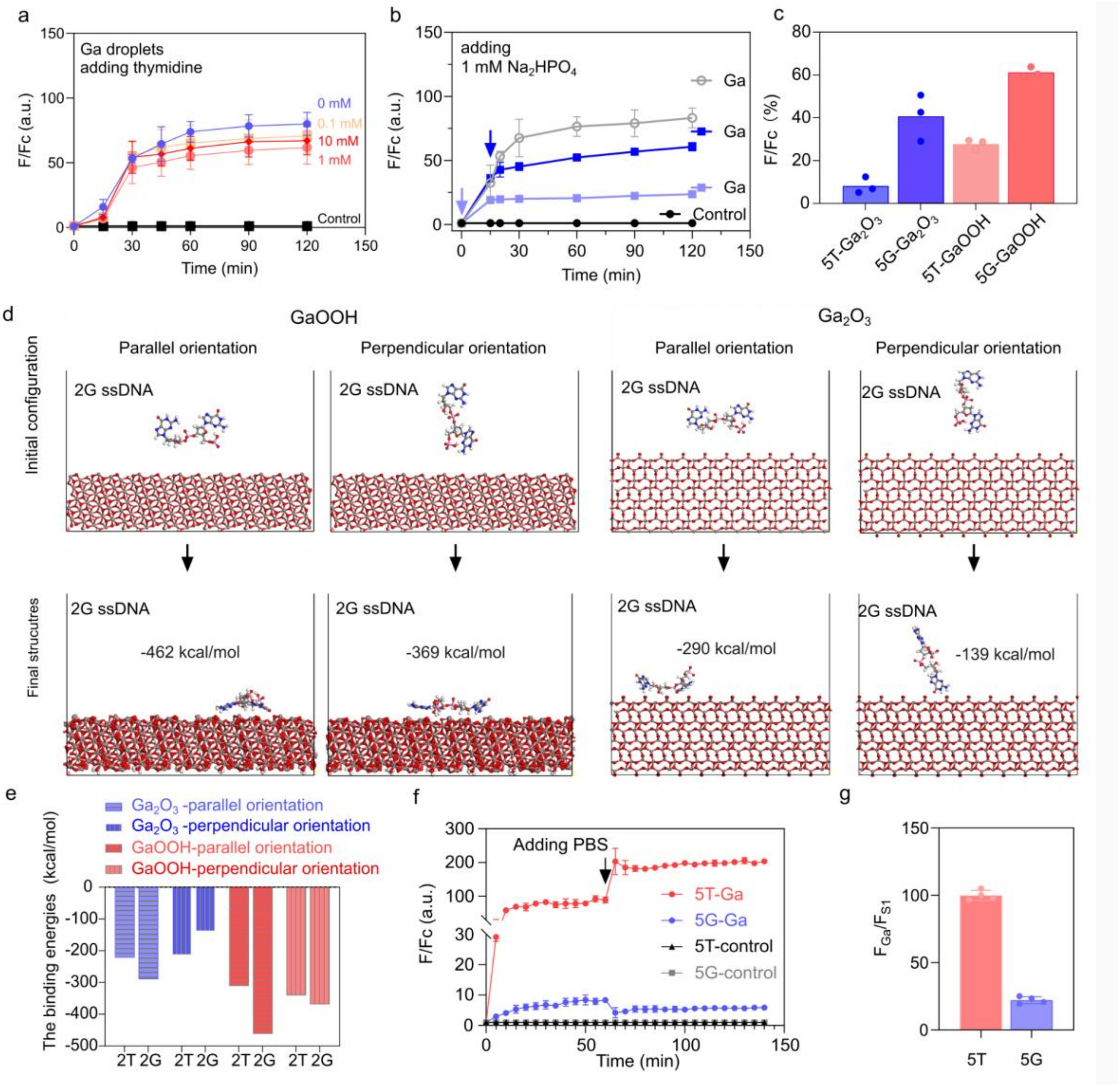

Supplementary Fig. 29. The ssDNA adsorption on Ga droplets and base-sequence preference. a-b, DNA cleavage activity of Ga droplets in the presence of added **(a)** thymidine and **(b)** Na_2HPO_4. (n = 4 independent reactions). **(c)**, Adsorption percentage of 5T ssDNA TR-reporter on Ga oxide surfaces (Ga_2O_3 and GaOOH) (n = 3 independent reactions, Supplementary Method 3,8). **(d)** Classical molecular dynamics (MD) simulations of 2G ssDNA interacting with Ga oxide layers (Ga_2O_3 and GaOOH) using two initial configurations: ssDNA oriented perpendicular or parallel to the surface (Supplementary Method 6). **(e)** Comparison of binding energies in MD simulations of 2T ssDNA and 2G ssDNA interacting with Ga_2O_3 and GaOOH surfaces. **(f)** DNA cleavage activity of excess Ga droplets (0.5 mg mL^{-1}, fivefold higher than that used in other experiments) toward 5T and 5G ssDNA TR-reporters. Adding PBS pH=7 to promote the detachment of adsorbed ssDNA reporter from the surface of Ga droplets. (n = 4 independent reactions) **(g)** End-point fluorescence intensity (at 120 min) of 5T and 5G ssDNA TR-reporters cleaved by Ga droplets, normalized to the activity of S1 nuclease (n = 4 independent reactions). Data are presented as means ± SDs. Source data are provided as a Source Data file.

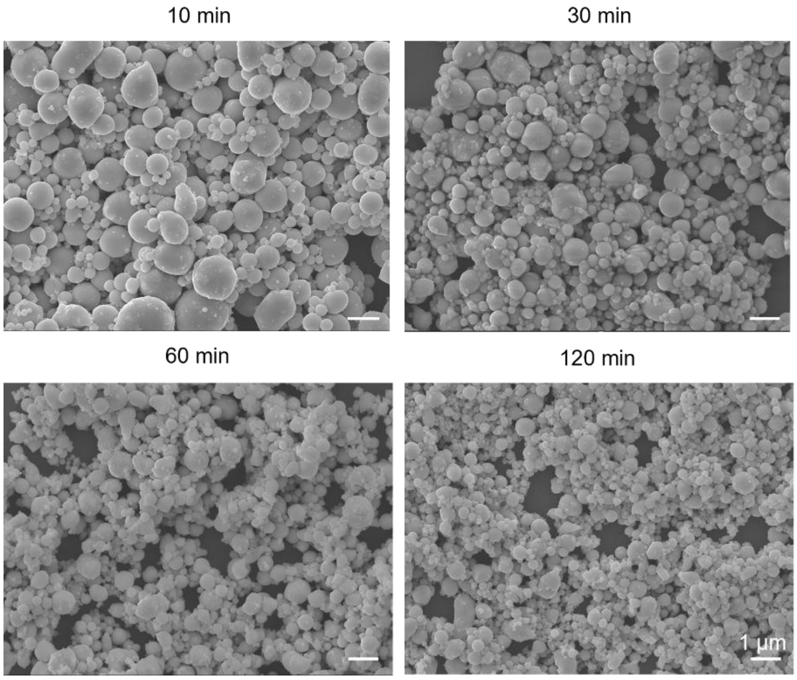

Supplementary Fig. 30. Representative SEM images of Ga droplets formed by sonication for 10, 30, 60, and 120 min. Each experiment was repeated three times independently with similar results.

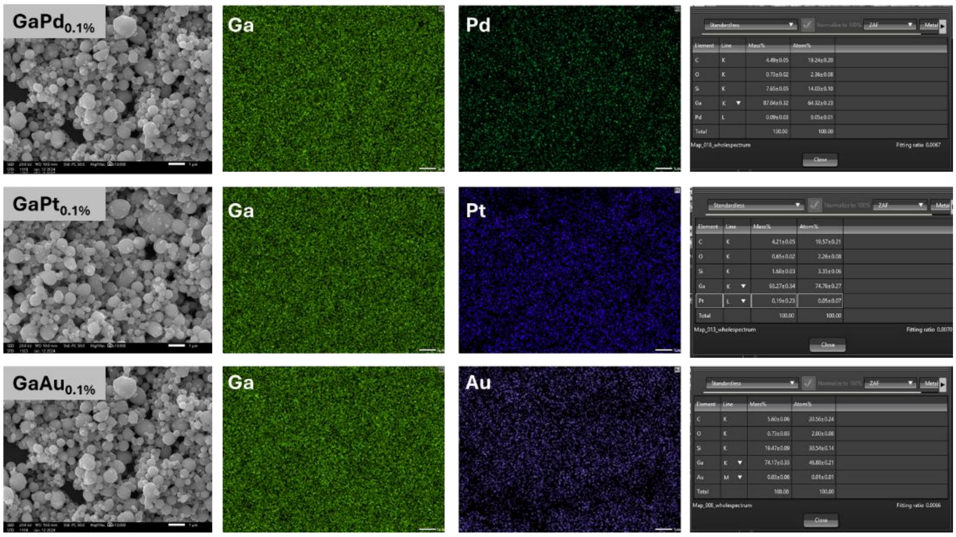

Supplementary Fig. 31. SEM images, EDS elemental maps, and elemental composition analysis of Ga-based alloys. Each experiment was repeated three times independently with similar results.

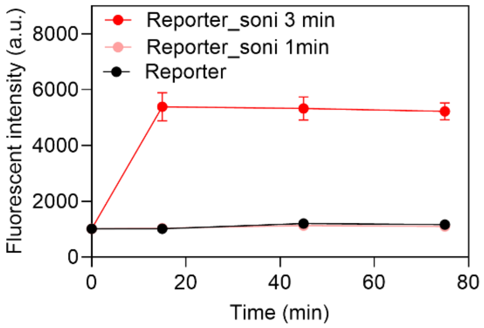

Supplementary Fig. 32. Effect of short bath sonication (1 min vs. 3 min) on TR-ssDNA reporter integrity, showing 1 min sonication has minimal negative impact (n = 4 independent reactions). Data are presented as means ± SDs. Source data are provided as a Source Data file.

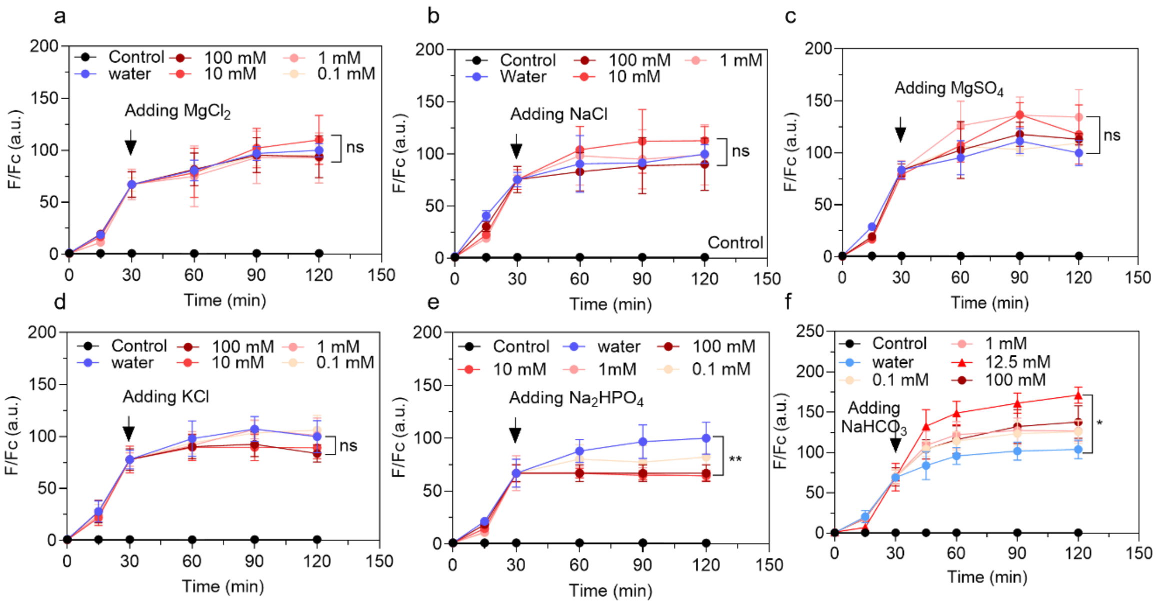

Supplementary Fig. 33. Effect of various salt additives (a) $MgCl_2$, (b) NaCl, (c) $MgSO_4$, (d) KCl, (e) Na_2HPO_4, (f) $NaHCO_3$ on Ga-mediated TR-reporter cleavage, adding in 30 min. $p < 0.05$ is considered as statistically significant. n = 4 independent reactions. *$p < 0.05$, **$p < 0.01$, ***$p < 0.001$, and ****$p < 0.0001$. Data are presented as means ± SDs. Source data are provided as a Source Data file.

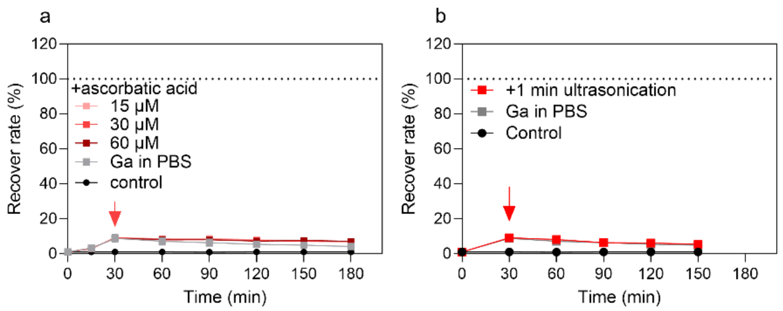

Supplementary Fig. 34. Fluorescence intensity of TR-ssDNA reporter co-incubated with Ga droplets in PBS upon (a) addition of ascorbic acid (final concentration) and (b) application of 1-min bath sonication. n = 4 independent reactions. Data are presented as means ± SDs. Source data are provided as a Source Data file.

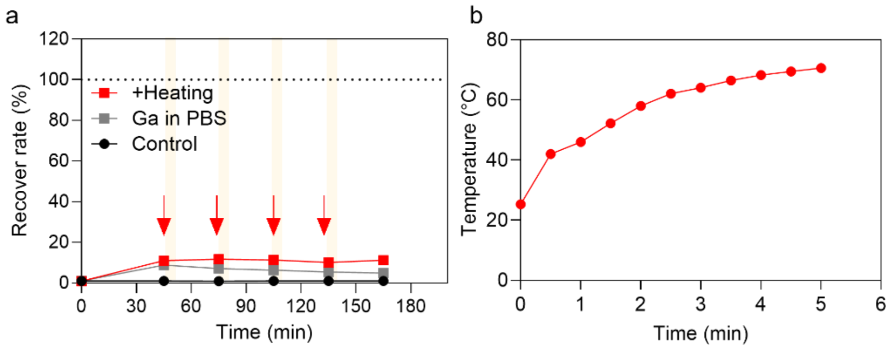

Supplementary Fig. 35. Fluorescence intensity of TR-ssDNA reporter co-incubated with Ga droplets in PBS under heating treatment. (b) The corresponding temperature curves. n = 4 independent reactions. Data are presented as means ± SDs. Source data are provided as a Source Data file.

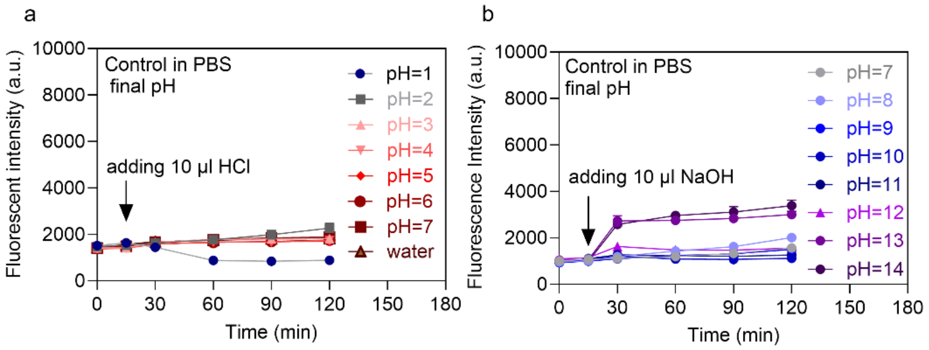

Supplementary Fig. 36. Control experiments showing fluorescence changes of the TR-reporter in PBS upon HCl or NaOH addition. In the control sample containing only TR-ssDNA reporter with HCl or NaOH, the fluorescence increased modestly from ~1500 a.u. to < 3000 a.u. (< 2-fold). In contrast, samples with Ga droplets exhibited a pronounced fluorescence increase exceeding 120,000 a.u. (>80-fold in Fig.6). These results indicate that Ga droplets act as efficient artificial nuclease mimics, enabling DNA cleavage even under extreme pH conditions. n = 4 independent reactions. Data are presented as means ± SDs. Source data are provided as a Source Data file.

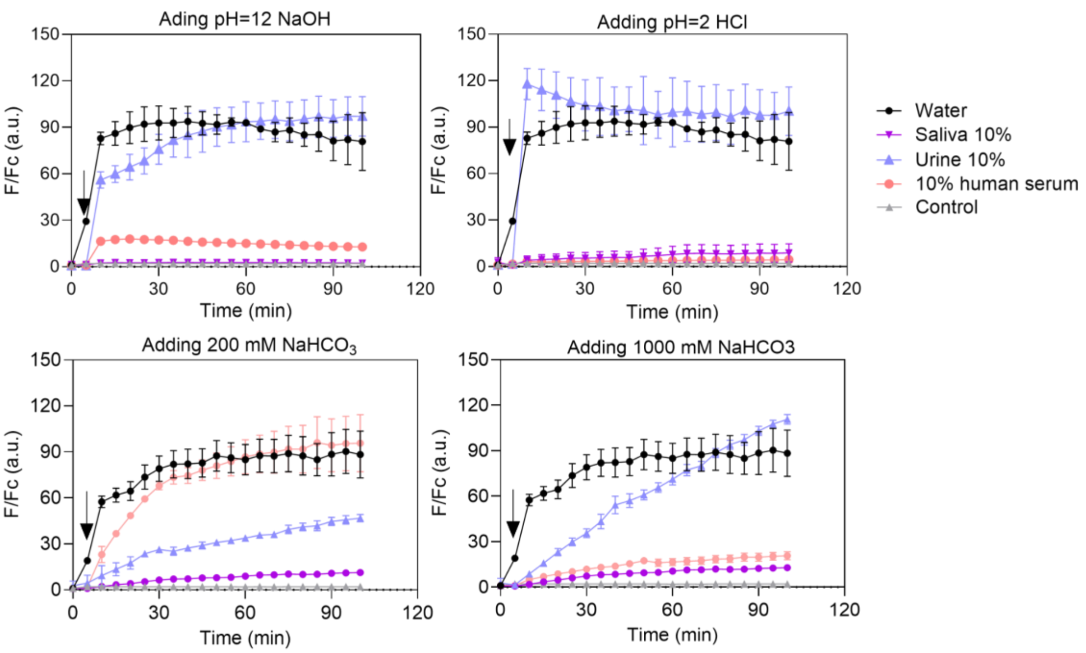

Supplementary Fig. 37. Fluorescence intensity of TR-ssDNA reporter co-incubated with Ga droplets in 10% saliva, urine, and human serum, after treatment with (a) NaOH (final pH = 11), (b) HCl (final pH = 3), and $NaHCO_3$ at final concentrations of (c) 20 mM and (d) 100 mM. n = 3 independent reactions. Data are presented as means ± SDs. Source data are provided as a Source Data file.

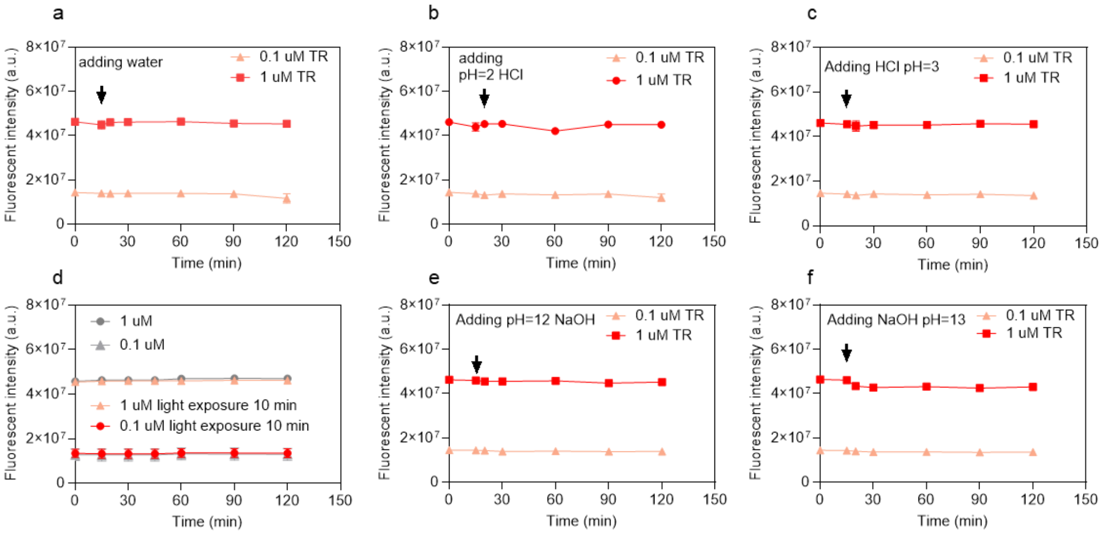

Supplementary Fig. 38. Influence of external stimuli on the Texas Red dye: (a) water, (b) pH 2 HCl, (c) pH 3 HCl, (d) light exposure for 10 min, (e) pH 12 NaOH, and (f) pH 13 NaOH; 10 µL was added to the 100 µL solution at the time indicated by the arrow. n = 4 independent reactions. Data are presented as means ± SDs. Source data are provided as a Source Data file.

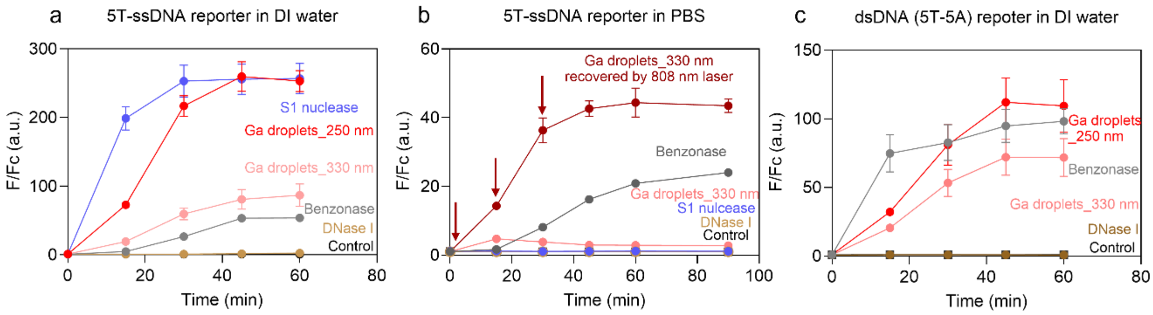

Supplementary Fig. 39. Comparison of the DNA cleavage activity of Ga droplets and commercial nucleases (S1 nuclease, Benzonase, DNase I). Ga droplets with mean diameters of ~330 nm and ~250 nm were synthesized by probe sonication for 30 min and 120 min, respectively. n = 3 independent reactions. Data are presented as means ± SDs. Source data are provided as a Source Data file.

Supplementary Table

Supplementary Table 1. DNA reporter and oligonucleotide used in the experiment

Reporter	Oligo type	Sequence 5'-3'	Fluorophores	Quenchers
TR-ssDNA reporter	ssDNA	TTATT	5'-Texas Red	3'-BHQ2
5A-ssDNA reporter	ssDNA	AAAAA	5'-Texas Red	3'-BHQ2
5T-ssDNA reporter	ssDNA	TTTTT	5'-Texas Red	3'-BHQ2
5C-ssDNA reporter	ssDNA	CCCCC	5'-Texas Red	3'-BHQ2
5G-ssDNA reporter	ssDNA	GGGGG	5'-Texas Red	3'-BHQ2
5A-ssDNA Cy3 reporter	ssDNA	AAAAA	5'- Cy3	3'-BHQ2
5T-ssDNA Cy3 reporter	ssDNA	TTTTT	5'- Cy3	3'-BHQ2
5C-ssDNA Cy3 reporter	ssDNA	CCCCC	5'- Cy3	3'-BHQ2
5G-ssDNA Cy3 reporter	ssDNA	GGGGG	5'- Cy3	3'-BHQ2
5T ssDNA	ssDNA	TTTTT	N/A	N/A
TR-dsDNA reporter	dsDNA	5′- T T T A A -3′<br>3′- A A A T T -5′	5'-Texas Red	3'-BHQ2

Supplementary Table 2. Kinetic parameters for Ga droplets acting as artificial nuclease mimics.

Supplementary Table 2-1. Michaelis–Menten parameters

Michaelis-Menten	22 °C	37 °C	45 °C
Best-fit values			
V_{max}	1.325	1.752	2.343
K_m	159.1	195.9	123.4
95% CI (profile likelihood)			
V_{max}	1.159 to 1.543	1.419 to 2.225	1.935 to 2.884
K_m	108.4 to 238.3	117.7 to 335.2	72.25 to 214.1
Goodness of Fit			
Degrees of Freedom	26	20	16
R squared	0.8751	0.8341	0.8206
Sum of Squares	0.3635	0.7449	1.157
Sy.x	0.1182	0.193	0.2689

Supplementary Table 2-2. Parameters used for apparent activation energy determination.

T (°C)	V_{max} (nM/min)	K_m (nM)	T(K)	K_{cat} (min^{-1})	1/T	Ln（K_{cat}）
22	1.325	158.9	295.15	1.325	0.003388	0.281412459
37	1.752	145.7	310.15	1.752	0.003224	0.560757993
45	2.343	616.8	318.15	2.343	0.003143	0.85143216

Supplementary Table 3. The frequency of dinucleotides at cleavage points by Ga droplets

Dinucleotide	Control	Ga-1h	Ga-4h	HindIII
AC+CA	12.5259	11.4198	11.7948	1.1490
AG+GA	12.0649	12.1169	12.1403	0.7423
CG+GC	16.4669	14.5305	14.3878	1.6868
CC+GG	12.7794	13.3166	13.6340	0.4553
CT+TC	12.0419	12.0546	11.8954	0.8064
AA+TT	13.1136	15.2815	15.0001	94.3205
TA+AT	8.7577	9.05845	9.1581	0.2546
TG+GT	12.2493	12.2213	11.9893	0.5851

Supplementary Table 4. Potential reactions and ROS chain reactions triggered by the introduction of Ga droplets into DI water.

Half reaction	Redox potential* (E_0) [V]
$Ga - 3e^- \rightarrow Ga^{3+}$	0.55
$O_2 + e^- \rightarrow O_2^- \cdot$	-0.16
$O_2^- \cdot + 2H^- + e^- \rightarrow H_2O_2$	0.89
$H_2O_2 + H^+ + e^- \rightarrow H_2O + \cdot OH$	0.38

*pH=7.0 [53]

Supplementary Table 5. Qualitative analysis of ssDNA related peaks in the MS spectra of samples following ZipTip desalting.

No	Supposed formula	Exact Mass	Proposed structure	m/z theoretical	ion	m/z measured
1.	$C_{50}H_{66}N_{10}O_{33}P_4$	1458.27442	5T ssDNA	492.41151	$[M-4H^{+}+Na^{+}]^{3-}$	492.41095
2.	$C_{50}H_{66}N_{10}O_{33}P_4$	1458.27442	5T ssDNA	363.56133	$[M-4H^{+}]^{4-}$	363.56128
3.	$C_{50}H_{66}N_{10}O_{33}P_4$	1458.27442	5T ssDNA	485.08420	$[M-3H^{+}]^{3-}$	485.08390
4.	$C_{50}H_{66}N_{10}O_{33}P_4$	1458.27442	5T ssDNA	728.12993	$[M-2H^{+}]^{2-}$	728.12934
5.	$C_{50}H_{66}N_{10}O_{33}P_4$	1458.27442	5T ssDNA	739.12091	$[M-3H^{+}+Na^{+}]^{2-}$	739.12066
6.	$C_{50}H_{66}N_{10}O_{33}P_4$	1458.27442	5T ssDNA	1457.26715	$[M-H^{+}]^{-}$	1457.26727
7.	$C_{40}H_{54}N_8O_{29}P_4$	1234.19472	4T (hydroxyl + phosphate monoester termini)	616.09008	$[M-2H^{+}]^{2-}$	616.08925
8.	$C_{40}H_{53}N_8O_{26}P_3$	1154.22838	4T (hydroxyl termini)	576.10691	$[M-2H^{+}]^{2-}$	576.10648

Supplementary Table 6. Qualitative analysis of major non-DNA-related peaks in the MS spectra of samples after ZipTip desalting.

No.	Supposed formula	Exact Mass	Proposed structure*	m/*z* theoretical	ion	m/*z* measured
1.	$C_8H_{16}O_2$	144.11503	Octanoic acid	143.10775	$[M-H^+]^-$	143.10774
2.	$C_9H_{18}O_2$	158.13068	Nonanoic acid	157.12340	$[M-H^+]^-$	157.12338
3.	$C_{10}H_{20}O_2$	172.14633	Decanoic acid	171.13905	$[M-H^+]^-$	171.13905
4.	$C_{11}H_{22}O_2$	186.16198	Undecanoic acid	185.15470	$[M-H^+]^-$	185.15464
5.	$C_{12}H_{24}O_2$	200.17763	Dodecanoic acid	199.17035	$[M-H^+]^-$	199.17026
6.	$C_{14}H_{22}O$	206.16707	4-octylphenol	205.15979	$[M-H^+]^-$	205.15968
7.	$C_{14}H_{22}O$	206.16707	4-octylphenol	265.18092	$[M+CH_3COO^-]^-$	265.18073
8.	$C_{14}H_{28}O_2$	228.20893	Tetradecanoic acid	227.20165	$[M-H^+]^-$	227.20153
9.	$C_{16}H_{32}O_2$	256.24023	Palmitic acid	255.23295	$[M-H^+]^-$	255.23278
10.	$C_{18}H_{36}O_2$	284.27153	Stearic acid	283.26425	$[M-H^+]^-$	283.26400
11.	$C_{17}H_{28}O_3S$	312.17592	4-undecylbenzene-sulfonic acid	311.16864	$[M-H^+]^-$	311.16837
12.	$C_{18}H_{30}O_3S$	326.19157	4-dodecylbenzene-sulfonic acid	325.18429	$[M-H^+]^-$	325.18401
13.	$C_{19}H_{32}O_3S$	340.20722	4-tridecylbenzene-sulfonic acid	339.19994	$[M-H^+]^-$	339.19969
14.	$C_{21}H_{42}O_6$	390.29814	Pentadecyl D-glucoside	389.29086	$[M-H^+]^-$	389.29056
15.	$C_{23}H_{46}O_6$	418.32944	Heptadecyl D-glucoside	417.32216	$[M-H^+]^-$	417.32190

*The theoretical results are from the simulation of ChemDraw Professional (Version 23.1.1.3). Some peaks observed in the MS spectra are not attributable to DNA fragments and cannot be assigned using standard MS databases, which are most likely derived from fatty acid–like residues introduced during the ZipTip

desalting process. Most fatty acids may come from the ZipTip desalting column; they are commonly used in silica modification. Other background chemicals may come from instrument cleaning (e.g., surfactants) or other experimental sources.

Supplementary Table 7. Calculated m/z values of theoretical hydrolytic cleavage fragments (1T–5T) under different ionization states.

Number*		Status	Residue	MS	$[M\text{-}H^+]$	$[M\text{+}Cl^-]^-$	$[M\text{-}2H^+]^{2-}/2$	$[M\text{-}2H^+\text{+}Na^+]^-$	$[M\text{-}3H^+\text{+}Na^+]^{2-}/2$	$[M\text{-}3H^+]^{3-}/3$	$[M\text{-}4H^+\text{+}Na^+]^{3-}/3$	$[M\text{-}4H^+]^{4-}/4$	$[M\text{-}4H^+\text{+}Ga^{3+}]^-$
1	5T	whole	3'-OH, 5'-OH	1458.2744	1457.2671	1493.2438	728.1299	1479.2492	739.1210	485.0842	492.4116	363.5613	1523.9692
2		whole	3'-OH, 5'-OH	1154.2284	1153.2211	1189.1978	576.1069	1175.2032	587.0980	383.7355	391.0629	287.5498	1219.9232
3	4T	hydrolysed 5'	3'-OH, 5'-extra p acid	1234.1947	1233.1874	1269.1641	616.0901	1255.1695	627.0812	410.3909	417.7184	307.5414	1299.8895
4		hydrolysed 3'	3'-extra p acid, 5'-OH	1234.1947	1233.1874	1269.1641	616.0901	1255.1695	627.0812	410.3909	417.7184	307.5414	1299.8895
5		whole	3'-OH, 5'-OH	850.1823	849.1750	885.1517	424.0839	871.1572	435.0750	282.3868			
6		hydrolysed 5'	3'-OH, 5'-extra p acid	930.1487	929.1414	965.1181	464.0670	951.1235	475.0582	309.0423			
7	3T	hydrolysed 3'	3'-extra p acid, 5'-OH	930.1487	929.1414	965.1181	464.0670	951.1235	475.0582	309.0423			
8		hydrolysed 5' and 3'	3'-extra p acid, 5'-extra p acid	1010.1150	1009.1077	1045.0844	504.0502	1031.0898	515.0413	335.6977			
9		whole	3'-OH, 5'-OH	546.1363	545.1290	581.1057	272.0609	567.1111					
10	2T	hydrolysed 5'	3'-OH, 5'-extra p acid	626.1026	625.0953	661.0720	312.0440	647.0775					
11		hydrolysed 3'	3'-extra p acid, 5'-OH	626.1026	625.0953	661.0720	312.0440	647.0775					
12		hydrolysed 5' and 3'	3'-extra p acid, 5'-extra p acid	706.0690	705.0617	741.0384	352.0272	727.0438					
13		whole (without p acid)	3'-OH, 5'-OH	242.0903	241.0830	277.0596							
14		hydrolysed 5'	3'-OH, 5'-extra p acid	322.0566	321.0493	357.0260							
15	T	hydrolysed 3'	3'-extra p acid, 5'-OH	322.0566	321.0493	357.0260							
16		hydrolysed 5' and 3'	3'-extra p acid, 5'-extra p acid	402.0229	401.0156	436.9923							

*The chemical structures corresponding to each number are provided in Supplementary Figure 10.

Supplementary Table 8. Observed m/z values of hydrolytic cleavage fragments (1T–5T) under different ionization states in control sample.

Product number*		Status	Residue	Expected MS	[M-H$^+$]	[M+Cl$^-$]$^-$	[M-2H$^+$]$^{2-}$/2	[M-2H$^+$+Na$^+$]$^-$	[M-3H$^+$+Na$^+$]$^{2-}$/2	[M-3H$^+$]$^{3-}$/3	[M-4H$^+$+Na$^+$]$^{3-}$/3	[M-4H$^+$]$^{4-}$/4	[M-4H$^+$+Ga^{3+}]$^-$
1	5T	whole	3'-OH, 5'-OH	1458.2744	N/A	N/A	4.49E+03	N/A	1.21E+04	6.22E+06	1.19E+07	5.48E+07	N/A
2	4T	whole	3'-OH, 5'-OH	1154.2284	N/A	N/A	3.00E+04	N/A	N/A	1.87E+06	6.91E+04	N/A	N/A
3		hydrolysed 5'	3'-OH, 5'-extra p acid	1234.1947	N/A	N/A	1.66E+04	N/A	N/A	3.78E+05	8.27E+04	N/A	N/A
4		hydrolysed 3'	3'-extra p acid, 5'-OH	1234.1947	N/A	N/A	1.66E+04	N/A	N/A	3.78E+05	8.27E+04	N/A	N/A
5	3T	whole	3'-OH, 5'-OH	850.1823	1.89E+04	N/A	8.08E+05	N/A	1.70E+04	N/A			
6		hydrolysed 5'	3'-OH, 5'-extra p acid	930.1487	1.29E+04	N/A	1.14E+05	N/A	3.55E+04	2.78E+05			
7		hydrolysed 3'	3'-extra p acid, 5'-OH	930.1487	1.29E+04	N/A	1.14E+05	N/A	3.55E+04	2.78E+05			
8		hydrolysed 5' and 3'	3'-extra p acid, 5'-extra p acid	1010.1150	N/A	N/A	N/A	N/A	N/A	N/A			
9	2T	whole	3'-OH, 5'-OH	546.1363	1.10E+05	N/A	N/A	N/A	N/A	N/A			
10		hydrolysed 5'	3'-OH, 5'-extra p	626.1026	N/A	N/A	3.14E+05	N/A	N/A	N/A			
11		hydrolysed 3'	3'-extra p acid, 5'-OH	626.1026	N/A	N/A	3.14E+05	N/A	N/A	N/A			
12		hydrolysed 5' and 3'	3'-extra p acid, 5'-extra p acid	706.0690	N/A	N/A							
13	1T	whole (without p acid)	3'-OH, 5'-OH	242.0903	1.59E+05	N/A							
14		hydrolysed 5'	3'-OH, 5'-extra p acid	322.0566	4.88E+05	N/A							
15		hydrolysed 3'	3'-extra p acid, 5'-OH	322.0566	4.88E+05	N/A							
16		hydrolysed 5' and 3'	3'-extra p acid, 5'-extra p acid	402.0229									

*The chemical structures corresponding to each number are provided in Supplementary Figure 24.

Supplementary Table 9. Observed m/z values of hydrolytic cleavage fragments (1T–5T) under different ionization states in Ga_1.5 h sample.

Product number*		Status	Residue	Expected MS	$[M-H^+]$	$[M+Cl^-]^-$	$[M-2H^+]^{2-}/2$	$[M-2H^++Na^+]^-$	$[M-3H^++Na^+]^{2-}/2$	$[M-3H^+]^{3-}/3$	$[M-4H^++Na^+]^{3-}/3$	$[M-4H^+]^{4-}/4$	$[M-4H^++Ga^{3+}]^-$
1	5T	whole	3'-OH, 5'-OH	1458.2744	3.73E+03	N/A	2.99E+06	3.44E+04	1.46E+06	8.62E+06	5.69E+05	1.70E+07	N/A
2	4T	whole	3'-OH, 5'-OH	1154.2284	N/A	N/A	5.65E+04	N/A	N/A	2.60E+05	N/A	N/A	N/A
3		hydrolysed 5'	3'-OH, 5'-extra p acid	1234.1947	N/A	N/A	5.65E+03	N/A	N/A	4.19E+04	N/A	N/A	N/A
4		hydrolysed 3'	3'-extra p acid, 5'-OH	1234.1947	N/A	N/A	5.65E+03	N/A	N/A	4.19E+04	N/A	N/A	N/A
5	3T	whole	3'-OH, 5'-OH	850.1823	N/A	N/A	3.97E+04	N/A	N/A	N/A	N/A		
6		hydrolysed 5'	3'-OH, 5'-extra p acid	930.1487	N/A	N/A	N/A	N/A	N/A	N/A			
7		hydrolysed 3'	3'-extra p acid, 5'-OH	930.1487	N/A	N/A	N/A	N/A	N/A	N/A			
8		hydrolysed 5' and 3'	3'-extra p acid, 5'-extra p acid	1010.1150	N/A	N/A	N/A	N/A	N/A	N/A			
9	2T	whole	3'-OH, 5'-OH	546.1363	9.89E+03	N/A	N/A	N/A					
10		hydrolysed 5'	3'-OH, 5'-extra p acid	626.1026	N/A	N/A	N/A	N/A					
11		hydrolysed 3'	3'-extra p acid, 5'-OH	626.1026	N/A	N/A							
12		hydrolysed 5' and 3'	3'-extra p acid, 5'-extra p acid	706.0690	N/A	N/A							
13	T	whole (without p acid)	3'-OH, 5'-OH	242.0903	N/A	N/A							
14		hydrolysed 5'	3'-OH, 5'-extra p acid	322.0566	N/A	N/A							
15		hydrolysed 3'	3'-extra p acid, 5'-OH	322.0566	N/A	N/A							
16		hydrolysed 5' and 3'	3'-extra p acid, 5'-extra p acid	402.0229	N/A	N/A							

*The chemical structures corresponding to each number are provided in Supplementary Figure 24.

Supplementary Table 10. Observed m/z values of hydrolytic cleavage fragments (1T–5T) under different ionization states in Ga_4 h sample.

Product number*		Status	Residue	Expected MS	[M-H$^+$]	[M+Cl$^-$]$^-$	[M-2H$^+$]$^{2-}$/2	[M-2H$^+$+Na$^+$]$^-$	[M-3H$^+$+Na$^+$]$^{2-}$/2	[M-3H$^+$]$^{3-}$/3	[M-4H$^+$+Na$^+$]$^{3-}$/3	[M-4H$^+$]$^{4-}$/4	[M-4H$^+$+Ga^{3+}]$^-$
1	5T	whole	3'-OH, 5'-OH	1458.2744	6.64E+04	N/A	3.48E+06	5.54E+04	1.93E+06	9.06E+06	1.11E+06	2.07E+07	N/A
2	4T	whole	3'-OH, 5'-OH	1154.2284	N/A	N/A	9.35E+04	N/A	N/A	4.55E+05	N/A	N/A	N/A
3		hydrolysed 5'	3'-OH, 5'-extra p acid	1234.1947	N/A	N/A	2.05E+04	N/A	N/A	1.71E+05	N/A	N/A	N/A
4		hydrolysed 3'	3'-extra p acid, 5'-OH	1234.1947	N/A	N/A	2.05E+04	N/A	N/A	1.71E+05	N/A	N/A	N/A
5	3T	whole	3'-OH, 5'-OH	850.1823	N/A	N/A	8.45E+04	N/A	N/A	N/A			
6		hydrolysed 5'	3'-OH, 5'-extra p acid	930.1487	N/A	N/A	2.46E+04	N/A	N/A	N/A			
7		hydrolysed 3'	3'-extra p acid, 5'-OH	930.1487	N/A	N/A	N/A	N/A	N/A	N/A			
8		hydrolysed 5' and 3'	3'-extra p acid, 5'-extra p acid	1010.1150	N/A	N/A	N/A	N/A	N/A	N/A			
9	2T	whole	3'-OH, 5'-OH	546.1363	3.66E+04	N/A	N/A	N/A					
10		hydrolysed 5'	3'-OH, 5'-extra p acid	626.1026	N/A	N/A	N/A	N/A					
11		hydrolysed 3'	3'-extra p acid, 5'-OH	626.1026	N/A	N/A	N/A	N/A					
12		hydrolysed 5' and 3'	3'-extra p acid, 5'-extra p acid	706.0690	N/A	N/A	N/A	N/A					
\13	T	whole (without p acid)	3'-OH, 5'-OH	242.0903	N/A	N/A							
14		hydrolysed 5'	3'-OH, 5'-extra p acid	322.0566	3.12E+04	N/A							
15		hydrolysed 3'	3'-extra p acid, 5'-OH	322.0566	3.12E+04	N/A							
16		hydrolysed 5' and 3'	3'-extra p acid, 5'-extra p acid	402.0229	N/A	N/A							

*The chemical structures corresponding to each number are provided in Supplementary Figure 24.

Supplementary Table 11. Calculated m/z values of theoretical oxidative cleavage fragments under different ionization states.

Product number*		Expected MS	[M-H$^+$]$^-$	[M-2H$^+$+Na$^+$]$^-$	[M-2H$^+$]$^{2-}$/2	[M-3H$^+$+Na$^+$]$^{2-}$ /2	[M-3H$^+$]$^{3-}$/3	[M-4H$^+$+Na$^+$]$^{3-}$/3	[M-4H$^+$]$^{4-}$/4
1,2	mono 1	364.0671	363.0598	385.0419					
	mono 1 +2T	668.1132	667.1059	689.0880	333.0493	344.0404			
	mono 1+ 2T	972.1592	971.1519	993.1340	485.0723	496.0634	323.0457		
	mono 1+ 3T	1276.2052	1275.1979	1297.1801	637.0953	648.0864	424.3944	431.7219	318.0440
3	mono 3	320.0409	319.0336	341.0157					
	mono 3 + T	624.0869	623.0796	645.0618	311.0361	322.0273			
	mono 3 + 2T	928.1330	927.1257	949.1078	463.0592	474.0503	308.3703		
4	mono 4	240.0746							
	mono 4 + T	544.1206	543.1133	565.0954					
	mono 4 + 2T	848.1667	847.1594	869.1415	423.0760				
	mono 4 + 3T	1152.2127	1151.2054	1173.1875	575.0990	586.0901	383.0636		

*The chemical structures corresponding to each number are provided in Supplementary Figure 25.

Supplementary Table 12. Calculated m/z values of theoretical fragments: product 1 to 5 in Supplementary. Fig. 26.

These tables summarize the screened data from this procedure. Representative main peaks and structures are shown in Supplementary. Fig. 26.

Supplementary Table 12-1. (only hydroxyl-group residues at the DNA fragment termini)

Product 1	Expected MS	$[M-H^+]^-$	$[M-2H^++Na^+]^-$	$[M-2H^+]^{2-}/2$	$[M-3H^++Na^+]^{2-}/2$	$[M-3H^+]^{3-}/3$	$[M-4H^++Na^+]^{3-}/3$	$[M-4H^+]^{4-}/4$
5T	1348.2264	1347.2191	1369.2016	673.1059	684.09715	448.4015	455.729	336.0493
4T	1044.18036	1043.17306	1065.15556	521.08288	532.07413	347.05282		
3T	740.13432	739.12702	761.10952	369.05986				
2T	436.08828	435.08098						
T	NA							
Product 2	Expected MS	$[M-H^+]^-$	$[M-2H^++Na^+]^-$	$[M-2H^+]^{2-}/2$	$[M-3H^++Na^+]^{2-}/2$	$[M-3H^+]^{3-}/3$	$[M-4H^++Na^+]^{3-}/3$	$[M-4H^+]^{4-}/4$
5T	1339.2373	1338.23	1360.2125	668.61135	679.6026	445.40513	452.73263	333.80202
4T	1035.19126	1034.18396	1056.16646	516.58833	527.57958	344.05645		
3T	731.14522	730.13792	752.12042	364.56531				
2T	427.09918	426.09188						
T	NA							
Product 3	Expected MS	$[M-H^+]^-$	$[M-2H^++Na^+]^-$	$[M-2H^+]^{2-}/2$	$[M-3H^++Na^+]^{2-}/2$	$[M-3H^+]^{3-}/3$	$[M-4H^++Na^+]^{3-}/3$	$[M-4H^+]^{4-}/4$
5T	1377.253	1376.2457	1398.2282	687.6192	698.61045	458.07703	465.40453	343.30595
4T	1073.20696	1072.19966	1094.18216	535.59618	546.58743	356.72835		
3T	769.16092	768.15362	790.13612	383.57316				
2T	465.11488	464.10758						
T								
Product 4	Expected MS	$[M-H^+]^-$	$[M-2H^++Na^+]^-$	$[M-2H^+]^{2-}/2$	$[M-3H^++Na^+]^{2-}/2$	$[M-3H^+]^{3-}/3$	$[M-4H^++Na^+]^{3-}/3$	$[M-4H^+]^{4-}/4$
5T	1411.2584	1410.2511	1432.2336	704.6219	715.61315	469.41217	476.73967	351.8073
4T	1107.21236	1106.20506	1128.18756	552.59888	563.59013	368.06349		
3T	803.16632	802.15902	824.14152	400.57586				
2T	499.12028	498.11298						

T								
Product 5	Expected MS	[M-H$^+$]$^-$	[M-2H$^+$+Na$^+$]$^-$	[M-2H$^+$]$^{2-}$/2	[M-3H$^+$+Na$^+$]$^{2-}$ /2	[M-3H$^+$]$^{3-}$/3	[M-4H$^+$+Na$^+$]$^{3-}$ /3	[M-4H$^+$]$^{4-}$ /4
5T	1296.2315	1295.2242	1317.2067	647.10845	658.0997	431.06987	438.39737	323.050575
4T	992.18546	991.17816	1013.16066	495.08543	506.07668	329.72119		
3T	688.13942	687.13212	709.11462	343.06241				
2T	384.09338	383.08608						
T	NA							

Supplementary Table 12-2. (only one hydroxyl-group residue at the DNA fragment termini)

Product 1	Expected MS	[M-H$^+$]$^-$	[M-2H$^+$+Na$^+$]$^-$	[M-2H$^+$]$^{2-}$/2	[M-3H$^+$+Na$^+$]$^{2-}$ /2	[M-3H$^+$]$^{3-}$/3
5T						
4T	1124.14666	1123.13936	1145.12186	561.06603	572.05728	373.7082533
3T	820.10062	819.09332	841.07582	409.04301		
2T	516.05458	515.04728				
T	212.00854	211.00124				
Product 2	Expected MS	[M-H$^+$]$^-$	[M-2H$^+$+Na$^+$]$^-$	[M-2H$^+$]$^{2-}$/2	[M-3H$^+$+Na$^+$]$^{2-}$ /2	[M-3H$^+$]$^{3-}$/3
5T						
4T	1115.15756	1114.15026	1136.13276	556.57148	567.56273	370.7118867
3T	811.11152	810.10422	832.08672	404.54846		
2T	507.06548	506.05818				
T	NA	NA				
Product 3	Expected MS	[M-H$^+$]$^-$	[M-2H$^+$+Na$^+$]$^-$	[M-2H$^+$]$^{2-}$/2	[M-3H$^+$+Na$^+$]$^{2-}$ /2	[M-3H$^+$]$^{3-}$/3
5T						
4T	1153.17326	1152.16596	1174.14846	575.57933	586.57058	383.3837867
3T	849.12722	848.11992	870.10242	423.55631		
2T	545.08118	544.07388				
T	241.03514	240.02784				
Product 4	Expected MS	[M-H$^+$]$^-$	[M-2H$^+$+Na$^+$]$^-$	[M-2H$^+$]$^{2-}$/2	[M-3H$^+$+Na$^+$]$^{2-}$ /2	[M-3H$^+$]$^{3-}$/3

5T						
4T	1187.17866	1186.17136	1208.15386	592.58203	603.57328	394.71892
3T	883.13262	882.12532	904.10782	440.55901		
2T	579.08658	578.07928				
T	NA	NA				
Product 5	Expected MS	$[M-H^+]^-$	$[M-2H^++Na^+]^-$	$[M-2H^+]^{2-}/2$	$[M-3H^++Na^+]^{2-}/2$	$[M-3H^+]^{3-}/3$
5T						
4T	1072.15176	1071.14446	1093.12696	535.06858	546.05983	356.37662
3T	768.10572	767.09842	789.08092	383.04556		
2T	464.05968	463.05238				
T	NA	NA				

Supplementary Table 12-3. (two hydroxyl-group residues at the DNA fragment termini)

Product 1	Expected MS	$[M-H^+]^-$	$[M-2H^++Na^+]^-$	$[M-2H^+]^{2-}/2$
5T				
4T				
3T	900.06692	899.05962	921.04212	449.02616
2T	596.02088	595.01358		
T	291.97484	290.96704		
Product 2	Expected MS	-H'	-2H+Na'	-2H/2'
5T				
4T				
3T	891.07782	890.07052	912.05302	444.53161
2T	587.03178	586.02448		
T	NA	NA		
Product 3	Expected MS	-H'	-2H+Na'	-2H/2'
5T				
4T				
3T	929.09352	928.08622	950.06872	463.53946

2T	625.04748	624.04018		
T	321.00144	319.99364		
Product 4	Expected MS	$[M-H^+]^-$	$[M-2H^++Na^+]^-$	$[M-2H^+]^{2-}/2$
5T				
4T				
3T	963.09892	962.09162	984.07412	480.54216
2T	659.05288	658.04558		
T	NA	NA		
Product 5	Expected MS	$[M-H^+]^-$	$[M-2H^++Na^+]^-$	$[M-2H^+]^{2-}/2$
5T				
4T				
3T	848.07202	847.06472	869.04722	423.02871
2T	544.02598	543.01868		
T	NA	NA		

Supplementary Table 13. MD calculation results

Supplementary Table 13-1. Lenard-Jones parameters for Ga_2O_3

Element	Epsilon (kca mol^{-1})	Sigma (Å)	Charges (e)
Ga	0.505000	4.008153	1.20
O	0.170000	3.000012	-0.80

Supplementary Table 13-2. Bond parameters for Ga_2O_3

Bond	K (kcal mol^{-1} Å^{-2})	r_0 (Å)
Ga-O	800.0000	1.9485

Supplementary Table 13-3. Angle parameters for Ga_2O_3

Angle	K (kcal mol^{-1} rad^{-2})	q_0 (degrees)
O-Ga-O	119.50287	106.960
Ga-O-Ga	119.50287	121.946

Supplementary Table 13-4. Lenard-Jones parameters for GaOOH

Element	Epsilon (kca mol^{-1})	Sigma (Å)	Charges (e)
Ga	0.505000	4.008153	0.450000
H	0.000000	0.000000	0.090000
OH	0.210400	3.066000	-0.240000
Oz	0.170000	3.000012	-0.300000

Supplementary Table 13-5. Bond parameters for GaOOH

Bond	K (kcal mol^{-1} Å^{-2})	r_0 (Å)
Ga-OH	597.500	2.05140
Ga-Oz	597.500	1.94647
H-OH	742.800	0.97000

Supplementary Table 13-6. Angle parameters for GaOOH

Angle	K (kcal mol^{-1} rad^{-2})	q_0 (degrees)
OH-Ga-OH	119.50287	76.926
Ga-OH-H	119.50287	111.988
Ga-OH-Ga	119.50287	103.074

Supplementary Table 14. Intracellular and extracellular ion types and concentrations

Ion molecule	Intracellular concentration	Extracellular concentration	Concentration in experiments	Reference
Sodium ion (Na^+)	5-15 mM	135-145 mM	NaCl: 100,10,1,0.1 mM	[13]
Potassium ion (K^+)	140-150 mM	3.5-5 mM	KCl: 100,10,1,0.1 mM	[14]
Chloride ion (Cl^-)	5-15 mM	98-106 mM	NaCl: 100,10,1,0.1 mM	[15]
Bicarbonate ion (HCO_3^-)	10-15 mM	23-30 mM	$NaHCO_3$: 100,10,1,0.1 mM	[16]
Calcium ion (Ca^{2+})	0.0001 mM	1-2 mM	$CaCl_2$: 10,1,0.1,0.01 mM	[17]
Magnesium ion (Mg^{2+})	0.5-1 mM	0.7-0.9 mM	$MgCl_2$: 10,1,0.1,0.01 mM	[18]
Phosphate ion (HPO_4^{2-})	0.5-5 mM	0.81-1.45 mM	Na_2HPO_4: 10,1,0.1,0.01 mM	[16]
Sulphate ion (SO_4^{2-})	0.5-1 mM	0.3~0.5 mM	$NaSO_4^{2-}$: 10,1,0.1,0.01 mM	[19]

Supplementary Table 15. Commercial nuclease products with no phosphate in their formulations

Products	Supplier	Buffer composition	Phosphate level
Alt-R™ CRISPR-Cas9	IDT	25 mM Tris-HCl, 300 mM NaCl, 0.1 mM EDTA, 1 mM DTT, 50% glycerol	Phosphate-free
EnGen Cas9 Nuclease	NEB	10 mM Tris-HCl, 300 mM NaCl, 0.1 mM EDTA, 1 mM DTT, 50% glycerol	Phosphate-free
EnGen Cas12a (Cpf1)	NEB	20 mM Sodium Acetate, 500 mM NaCl, 0.1 mM EDTA, 0.1 mM TCEP, 50% glycerol	Phosphate-free
GenCRISPR Cas12a	GenScript	20 mM Sodium Acetate, 500 mM NaCl, 1 mM TCEP, 0.1 mM EDTA, 50% glycerol	Phosphate-free
DNase I, RNase-free	Thermo Scientific	50 mM Tris-HCl, 10 mM $CaCl_2$, 50% glycerol	Phosphate-free
RNase A	Sigma-Aldrich	10 mM Tris-HCl, 50% glycerol	Phosphate-free
Benzonase Nuclease	Merck Millipore	20 mM Tris-HCl, 20 mM NaCl, 2 mM $MgCl_2$, 50% glycerol	Phosphate-free
Pierce Universal Nuclease	Thermo Scientific	20 mM Tris-HCl, 20 mM NaCl, 2 mM $MgCl_2$, 50% glycerol	Phosphate-free
Micrococcal Nuclease (MNase)	Thermo Scientific	10 mM Tris-HCl, 50 mM NaCl, 1 mM EDTA, 50% glycerol	Phosphate-free

Supplementary Table 16. Comparison between Ga droplets as nuclease mimics and commercial nucleases.

*The information was compiled from the official product manuals of Sigma-Aldrich, New England Biolabs, and Thermo Fisher Scientific.

	S1 Nuclease	Benzonase	DNase I	Ga droplets (~330 nm)
Description & Source	endonuclease from *Aspergillus* (fungal)	Broad-spectrum endonuclease from *Serratia marcescens* (bacterial)	Non-specific deoxyribonuclease from bovine pancreas	Metallic -Oxide micro/ nanodroplets
Substrate Specificity	ssDNA > >dsDNA; Endonuclease	dsDNA, ssDNA; Endonuclease	dsDNA (100-500 x over ssDNA); length > 6bp; Endonuclease	dsDNA, ssDNA, Endonuclease-like/nuclease-mimic cleavage
Temperature	Optimal at ~37 °C.	Optimal temp 37 °C (active 0-42 °C).	Optimal at ~37 °C.	Broad temperature tolerance (0-70 °C); cleavage activity
Ion tolerance	Salt Inhibition>100 mM monovalent salts (Na^+, K^+) Phosphate Inhibition>1 mM	Salt Inhibition>300 mM monovalent salts (Na^+, K^+) Phosphate Inhibition>20 mM	Salt Inhibition>20 mM. Phosphate Inhibition>1 mM	Salt Inhibition>300 mM monovalent salts (Na^+, K^+)； Phosphate Inhibition>1 mM；can be recovered
EDTA tolerance	No	No	No	Yes
Price	Sigma_N5661-50KU 401 AUD	Sigma_E8263-5KU 358 AUD	Biolabs_M0303S_1000U 152 AUD	~2.4 AUD / g
Cost required to achieve the effect in Supplementary Figure 4.5	0.033U/166 nM 0.145 AUD/μM ssDNA	0.033U/166 nM 0.0133 AUD/μM ssDNA	0.033U/166 nM 0.0277 AUD/μM ssDNA	0.033U/166 nM 0.00145 AUD/μM ssDNA

Supplementary References

1. Malakooti MH, Kazem N, Yan J, Pan C, Markvicka EJ, Matyjaszewski K*, et al*. Liquid metal supercooling for low-temperature thermoelectric wearables. *Adv. Funct. Mater.* **29**, 1906098 (2019).

2. Zhang C, Li L, Yang X, Shi J, Gui L, Liu J. Study on the nucleating agents for gallium to reduce its supercooling. *Int. J. Heat Mass Transfer* **148**, 119055 (2020).

3. Lin Y, Liu Y, Genzer J, Dickey MD. Shape-transformable liquid metal nanoparticles in aqueous solution. *Chem. Sci.* **8**, 3832-3837 (2017).

4. Nor-Azman N-A, Ghasemian MB, Fuchs R, Liu L, Widjajana MS, Yu R*, et al*. Mechanism behind the controlled generation of liquid metal nanoparticles by mechanical agitation. *ACS Nano* **18**, 11139-11152 (2024).

5. Huang X, Song D, Zhao Q, Young RP, Chen Y, Walter ED*, et al*. Photolysis of dissolved organic matter over hematite nanoplatelets. *Environ Sci Technol* **58**, 2798-2807 (2024).

6. Clément J-L, Ferré N, Siri D, Karoui H, Rockenbauer A, Tordo P. Assignment of the epr spectrum of 5,5-dimethyl-1-pyrroline n-oxide (dmpo) superoxide spin adduct. *J. Org. Chem.* **70**, 1198-1203 (2005).

7. Plimpton SJ, Kohlmeyer A, Thompson AP, Moore SG, Berger R. *Lammps: Large-scale atomic/molecular massively parallel simulator*. Zenodo, 2023.

8. Rappe A, Colwell K, Casewit C. Application of a universal force field to metal complexes. *Inorg. Chem.* **32**, 3438-3450 (1993).

9. Tang J, Lambie S, Meftahi N, Christofferson AJ, Yang J, Ghasemian MB*, et al*. Unique surface patterns emerging during solidification of liquid metal alloys. *Nat. Nanotechnol.* **16**, 431-439 (2021).

10. Hsieh Y-S, Li C-Y, Lin C-M, Wang N-F, Li JV, Houng M-P. Investigation of metal-insulator-semiconductor diode with alpha-ga2o3 insulating layer by liquid phase deposition. *Thin Solid Films* **685**, 414-419 (2019).

11. Lin Y, Liu Y, Genzer J, Dickey MD. Shape-transformable liquid metal nanoparticles in aqueous solution. *Chem. Sci.* **8**, 3832-3837 (2017).

12. Wang J, Wolf RM, Caldwell JW, Kollman PA, Case DA. Development and testing of a general amber force field. *J. Comput. Chem.* **25**, 1157-1174 (2004).

13. Yang L, Tan C-h, Hsieh M-J, Wang J, Duan Y, Cieplak P*, et al*. New-generation amber united-atom force field. *J. Phys. Chem. B* **110**, 13166-13176 (2006).

14. Evans DJ, Holian BL. The nose–hoover thermostat. *J. Chem. Phys.* **83**, 4069-4074 (1985).

15. Martyna GJ, Klein ML, Tuckerman M. Nosé–hoover chains: The canonical ensemble via continuous dynamics. *J. Chem. Phys.* **97**, 2635-2643 (1992).

16. Fu Y, He G, Liu Z, Wang J, Li M, Zhang Z*, et al*. DNA base pairing-inspired supramolecular nanodrug camouflaged by cancer-cell membrane for osteosarcoma treatment. *Small* **18**, e2202337 (2022).

17. Azhati A, Zhu H, Ouyang T, He T, Zeng Y, Wu P*, et al*. DNA-assisted creation of a library of ultrasmall multimetal/metal oxide nanoparticles confined in silica. *Small* **18**, e2107123 (2022).

18. Xiao G, Fang X, Ma YJ, Yan D. Multi-mode and dynamic persistent luminescence from metal cytosine halides through balancing excited-state proton transfer. *Adv. Sci.* **9**, e2200992 (2022).

19. Abozeed AA, Younis O, Al-Hossainy AF, El-Mawla NA, Sayed M, A MKE-D*, et al*. Combined experimental and td-dft/dmol(3) investigations, optical properties, and photoluminescence behavior of a thiazolopyrimidine derivative. *Sci. Rep.* **12**, 15674 (2022).

20. Ganjali Koli M, Eshaghi Malekshah R, Hajiabadi H. Insights from molecular dynamics and dft calculations into the interaction of 1,4-benzodiazepines with 2-hydroxypropyl-betacd in a theoretical study. *Sci. Rep.* **13**, 9866 (2023).

21. Delley B. An all‐electron numerical method for solving the local density functional for polyatomic molecules. *J. Chem. Phys.* **92**, 508-517 (1990).

22. Perdew JP, Burke K, Ernzerhof M. Generalized gradient approximation made simple. *Phys. Rev. Lett.* **77**, 3865-3868 (1996).

23. Kresse G, Furthmüller J. Efficiency of ab-initio total energy calculations for metals and semiconductors using a plane-wave basis set. *Comput. Mater. Sci.* **6**, 15-50 (1996).

24. Kresse G, Furthmüller J. Efficient iterative schemes for ab initio total-energy calculations using a plane-wave basis set. *Phys. Rev. B* **54**, 11169 (1996).

25. Lee YS, Ermler WC, Pitzer KS. Ab initio effective core potentials including relativistic effects. I. Formalism and applications to the xe and au atoms. *J. Chem. Phys.* **67**, 5861-5876 (1977).

26. Luo Y, Yin S, Lai W, Wang Y. Effects of global orbital cutoff value and numerical basis set size on accuracies of theoretical atomization energies. *Theor. Chem. Acc.* **133**, 1580 (2014).

27. Grimme S, Antony J, Ehrlich S, Krieg H. A consistent and accurate ab initio parametrization of density functional dispersion correction (dft-d) for the 94 elements h-pu. *J. Chem. Phys.* **132**, 154104 (2010).

28. Rahim MA, Tang J, Christofferson AJ, Kumar PV, Meftahi N, Centurion F, *et al.* Low-temperature liquid platinum catalyst. *Nat. Chem.* **14**, 935-941 (2022).

29. Tang J, Tang J, Mayyas M, Ghasemian MB, Sun J, Rahim MA, *et al.* Liquid-metal-enabled mechanical-energy-induced co_2 conversion. *Adv. Mater.* **34**, 2105789 (2022).

30. Idrus-Saidi SA, Tang J, Lambie S, Han J, Mayyas M, Ghasemian MB, *et al.* Liquid metal synthesis solvents for metallic crystals. *Science* **378**, 1118-1124 (2022).

31. Pan XM, Li J, Gan R, Hu XN. Preparation and in vitro evaluation of enteric-coated tablets of rosiglitazone sodium. *Saudi Pharm. J.* **23**, 581-586 (2015).

32. Luong TKN, Shestakova P, Parac-Vogt TN. Kinetic studies of phosphoester hydrolysis promoted by a dimeric tetrazirconium(iv) wells–dawson polyoxometalate. *Dalton Trans.* **45**, 12174-12180 (2016).

33. Kumar A, Pottiboyina V, Sevilla MD. Hydroxyl radical (oh•) reaction with guanine in an aqueous environment: A dft study. *J. Phys. Chem. B* **115**, 15129-15137 (2011).

34. Klvaňa M, Bren U, Florián J. Uniform free-energy profiles of the p-o bond formation and cleavage reactions catalyzed by DNA polymerases β and λ. *J Phys Chem B* **120**, 13017-13030 (2016).

35. Van R, Pan X, Rostami S, Liu J, Agarwal PK, Brooks B, *et al.* Exploring crispr-cas9 hnh-domain-catalyzed DNA cleavage using accelerated quantum mechanical molecular mechanical free energy simulation. *Biochem.* **64**, 289-299 (2025).

36. Creighton MA, Yuen MC, Susner MA, Farrell Z, Maruyama B, Tabor CE. Oxidation of gallium-based liquid metal alloys by water. *Langmuir* **36**, 12933-12941 (2020).

37. Gan T, Handschuh-Wang S, Shang W, Zhou X. Gaooh crystallite growth on liquid metal microdroplets in water: Influence of the local environment. *Langmuir* **38**, 14475-14484 (2022).

38. Wang Y, Zhao Y, Bollas A, Wang Y, Au KF. Nanopore sequencing technology, bioinformatics and applications. *Nat. Biotechnol.* **39**, 1348-1365 (2021).

39. Torres Montaguth OE, Cross SJ, Ingram KWA, Lee L, Diffin FM, Szczelkun MD. Endo-pore: High-throughput linked-end mapping of single DNA cleavage events using nanopore sequencing. *Nucleic Acids Res.* **49**, e118 (2021).

40. Rashid-Nadimi S, Okatenko V, Song M, Shetty Y, Bachmann AL, Mehrabian N, *et al.* The role of electrochemical oxidation on the interfacial tension of eutectic gallium indium. *ACS Electrochem.* **1**, 103-114 (2025).

41. King R, Bonfiglio R, Fernandez-Metzler C, Miller-Stein C, Olah T. Mechanistic investigation of ionization suppression in electrospray ionization. *J. Am. Soc. Mass Spectrom.* **11**, 942-950 (2000).

42. Ruparel H, Bi L, Li Z, Bai X, Kim DH, Turro NJ, *et al.* Design and synthesis of a 3′-o-allyl photocleavable fluorescent nucleotide as a reversible terminator for DNA sequencing by synthesis. *PNAS* **102**, 5932-5937 (2005).

43. Joyner JC, Keuper KD, Cowan JA. Analysis of rna cleavage by maldi-tof mass spectrometry. *Nucleic Acids Res.* **41**, e2 (2013).

44. Sinha ND, Jung KE. Analysis and purification of synthetic nucleic acids using hplc. *Curr. Protoc. Nucleic Acid Chem.* **61**, 10.15.11-10.15.39 (2015).

45. Li X-l, Yuan J, Dong Y-s, Fu C-h, Li M-T, Yu L-j. Optimization of an hplc method for determining the genomic methylation levels of taxus cells. *J. Chromatogr. Sci.* **54**, 200-205 (2015).

46. Pogozelski WK, Tullius TD. Oxidative strand scission of nucleic acids: routes initiated by hydrogen abstraction from the sugar moiety. *Chem. Rev.* **98**, 1089-1108 (1998).

47. Sugiyama H, Tsutsumi Y, Fujimoto K, Saito I. Photoinduced deoxyribose c2' oxidation in DNA. Alkali-dependent cleavage of erythrose-containing sites via a retroaldol reaction. *J. Am. Chem. Soc.* **115**, 4443-4448 (1993).

48. Hart MM, Smith CF, Yancey ST, Adamson RH. Toxicity and antitumor activity of gallium nitrate and periodically related metal salts2. *JNCI* **47**, 1121-1128 (1971).

49. Sun W, Qi M, Cheng S, Li C, Dong B, Wang L. Gallium and gallium compounds: New insights into the “trojan horse” strategy in medical applications. *Mater. Des.* **227**, 111704 (2023).

50. Guo X, Bruist MF, Davis DL, Bentzley CM. Secondary structural characterization of oligonucleotide strands using electrospray ionization mass spectrometry. *Nucleic Acids Res*. **33**, 3659-3666 (2005).

51. Lu C, Liu Y, Ying Y, Liu J. Comparison of mos2, ws2, and graphene oxide for DNA adsorption and sensing. *Langmuir* **33**, 630-637 (2017).

52. Liu G, Guo L, Wang C, Liu J, Hu Z, Dahlke HE, *et al*. Revealing the infiltration process and retention mechanisms of surface applied free DNA tracer through soil under flood irrigation. *Sci. Total Environ*. **905**, 167378 (2023).

53. Li J, Jiang M, Zhou H, Jin P, Cheung KMC, Chu PK, *et al*. Vanadium dioxide nanocoating induces tumor cell death through mitochondrial electron transport chain interruption. *Glob. Challenges* **3**, 1800058 (2019).

54. Madelin G, Kline R, Walvick R, Regatte RR. A method for estimating intracellular sodium concentration and extracellular volume fraction in brain in vivo using sodium magnetic resonance imaging. *Sci. Rep*. **4**, 4763 (2014).

55. Greenlee M, Wingo CS, McDonough AA, Youn JH, Kone BC. Narrative review: Evolving concepts in potassium homeostasis and hypokalemia. *Ann. Intern. Med*. **150**, 619-625 (2009).

56. Raut SK, Singh K, Sanghvi S, Loyo-Celis V, Varghese L, Singh ER, *et al*. Chloride ions in health and disease. *Biosci. Rep*. **44**, 1-17 (2024).

57. Shrimanker I, Bhattarai S. Electrolytes. *StatPearls [Internet]*. Treasure Island (FL): StatPearls Publishing; 2025.

58. Drake TM, Gupta V. Calcium. *StatPearls [Internet]*. Treasure Island (FL): StatPearls Publishing; 2025.

59. Drake TM, Gupta V. Magnesium. *StatPearls [Internet]*. Treasure Island (FL): StatPearls Publishing; 2025.

60. Tise CG, Anforth LE, Zhou AE, Perry JA, McArdle PF, Streeten EA, *et al*. Sex-specific effects of serum sulfate level and slc13a1 nonsense variants on dhea homeostasis. *Mol. Genet. Metab. Rep*. **10**, 84-91 (2017).
