# Supplementary material for: Gallium in liquid state shows nuclease-mimicking activity
Source: Nat Commun. 2026 Apr 10;17:5126. doi: 10.1038/s41467-026-71346-7 (PMC13247054; doi:10.1038/s41467-026-71346-7)
Supplement: Supplementary file 3 — Transparent Peer Review file [file 41467_2026_71346_MOESM3_ESM.pdf]

nature portfolio

Peer Review File

Gallium in Liquid State Shows Nuclease-Mimicking Activity

Corresponding Author: Professor Kourosh Kalantar-Zadeh

This file contains all reviewer reports in order by version, followed by all author rebuttals in order by version.

Version 0:

Reviewer comments:

Reviewer #1

(Remarks to the Author)
The authors report an interesting discovery that the nuclease-like activity of Gallium droplets with tunable and programmable capabilities, and a proposed mechanism involving nucleotide-biased adsorption and hydroxyl radical–assisted phosphodiester hydrolysis. This study is of significance for the development of artificial nuclease-mimicking systems. Thus, I would like to recommend the publications of this work. Following please find some minor issues.

1） It is intriguing that the nuclease-mimicking activity is highly dependent on the liquid state of Ga, as demonstrated in Figure 2c and d. What about the performance of solid-state Ga nanoparticles?

2） How stable are the Ga droplets during the process? Were there any precipitants observed, or did the droplet size change over time?

3） What is the long-term performance of the Ga droplets? Are they reusable?

4) How about the performance of nuclease activity of Ga droplet as compared with traditional method?

(Remarks on code availability)

Reviewer #2

(Remarks to the Author)
This manuscript submitted by Kalantar-Zadeh and Zhang reports on nucleolytic activity of liquid Ga. Whereas the approach is interesting, the work or at least its description within the manuscript is not conclusive even though a lot of different experiments were carried out. The conclusions “bioinspired catalysis in abiotic systems, thereby broadening the design space for artificial enzymes beyond traditional coordination complexes“ and “compared with other ligands and cofactors dependent on artificial metallonuclease, liquid Ga droplets uniquely serve as both the catalytic center and structural framework, forming a scaffold- free, metal-only platform. Their activity is tunable via synthetic strategies (e.g., droplet size, alloy composition, etc.) and dynamically controlled by external stimuli such as pH, light, sonication, and chemical additives.“ sound promising, however, these claims are not fulfilled based on the data shown in the manuscript. Other effects that might lead to the observed data have not been taken into account.

Major issues:
- Abstract: Phosphodiester hydrolysis is claimed to be carried out by hydroxyl radicals. The authors should refer to the literature where cleavage of phosphate esters is definitively not carried out by hydroxyl radicals. These highly reactive species rather attack the sugars/bases of DNA. The mechanism which is supported by DFT is not convincing (Figure 12 in the SI).
- Liquid Ga is claimed “as catalytic centers and as cofactors, forming a ligand- and cofactor-free artificial nuclease platform”. Clarify the term “cofactor”. In the context it is used here, “catalytic center” should be synonymous.
- Figure 1 c is completely hypothetical. Do the authors really know that the Ga droplets stay like this when/if they enter the cells? Also, how would they be taken up, some droplets are > 500 nm. Would phagocytosis be a potential mechanism?
- Experiments for DNA cleavage have apparently been carried out at room temperature. Since the melting point of Ga is

about 29 °C only, it is very important to specify what "room temperature" is. Depending on lab air conditioning and season r.t. might vary between 20 and 30 °C.
- For a mechanism where hydroxyl radicals are involved, it is difficult to infer DNA cleavage solely from a DNA beacon cleavage process based on fluorescence (de)quenching; radicals may also attack the dyes.
- The claim that Ga(0) is responsible for cleavage is difficult to conclude. In order to form reactive oxygen species, a change in oxidation state should be observed.
- Figure 4: Why would phosphate (from the diester bond) bind to elemental Ga? Phosphate typically binds Ga(III). DFT is not conclusive, see above.
- The mechanistic discussion about GaOOH is based on a Langmuir and a Chemical Science paper about eutectic gallium indium (EGaIn) liquid metal nanoparticles. It is not so clear that this can legitimately be compared with the present system.
- Also high pH values were tested. Shouldn't $Ga(OH)_3$ form at high pH values? Also, extreme pH conditions lead to DNA degradation anyways (dsDNA is typically stable only between pH 5-9).
- The formation of alloy particles is not described in detail.
- "After different periods of time, Na_2HPO_4 was added to a final concentration of 20 mM to stop the reaction and detach the cleaved plasmid from the droplets' surface, which improved visualization of bands in the gel." What is the rationale for this step? And could the increased pH (around 9) instead explain the observed effect?
- EPR: "Ga droplets (40 µL, 1 mg/mL in EtOH) underwent solvent exchange into water, followed by 5 s of sonication for dispersion. Then, 4 µL of 1 M DMPO (for ·OH) or TEMPO (for O_2^{-}) in aqueous solution was added and sonicated" Sonication itself may induce radical formation. Was this aspect considered and double-checked with the literature? Also, it is not clear what "solvent exchange" specifically means here.

Minor issues:
- For agarose gel electrophoresis, Ga droplets were "co-cultured" with DNA – "co-cultured" is inappropriate terminology. It is rather used when two cell lines are incubated at the same time at the same place. Better use "incubated together".
- Figure 2. It should be explained somewhere what "MinION" is.
- Also other ROS might play a role for DNA cleavage. Only hydroxyl radicals, superoxide radical anions and CO_2 radicals were evaluated.
- It is good to list contaminants from MS analysis, however, the presence of numerous fatty acids from ZIP-tip desalting seems unusual, and why are they present in such abundance?
- Clarify whether ICP-MS or -OES was used. The reader finds contradictory information about the ICP technique.
- Figure 3c: $Ga(OH)_3$ is mentioned in the description, but it is not shown in the figure.

(Remarks on code availability)

Reviewer #3

(Remarks to the Author)
The manuscript "Gallium in Liquid State Shows Nuclease-Mimicking Activity" presents a study on the use of liquid gallium for nuclease-like activity with preferred cleavage sites. It is an interesting and unusual work, combining aspects of heterogeneous catalysis and biochemistry. For me, the main serious weakness of this manuscript lies in the proposed role of gallium and its involvement in the cleavage process, which requires further research. Below are several points that could help improve the paper:
1. The study relies mainly on fluorescence data for cleavage, without providing kinetic constants, turnover numbers, or activation energies. The paper definitely requires more quantitative kinetic data, if possible, under varying pH, O_2 levels, and temperature.
2. It is unclear whether gallium acts catalytically or stoichiometrically (i.e., is consumed during oxidation). To clarify this, recycling experiments should be performed to confirm whether gallium deactivates or retains its activity.
3. The surface of liquid gallium particles is typically covered by a thin oxide film in aqueous environments. Although the authors state that Ga^0 is the active species, the reaction conditions strongly suggest that the reactions occur at the oxide–liquid interface. A clearer surface analysis (e.g., XPS, FTIR) would strengthen this claim. Atomic-resolution TEM of droplet surface evolution would also be very helpful.
4. The proposed OH-assisted cleavage is interesting, but the origin of OH radicals is not clear. Usually, such radicals are generated in photocatalysis; here, it appears they might arise from the oxidation of Ga by water, but the mechanism is not well explained. Radicals could also form sonochemically during redispersion of Ga, but they are unlikely to persist long enough to react. This pathway and the mechanism of OH radical generation should be studied in greater detail.
5. If OH radicals produced from water are involved in cleavage, hydroxyl groups should appear in the degraded DNA. I recommend performing experiments with labeled H_2O^{17} and detecting incorporation by NMR to confirm the proposed mechanism.
6. OH radicals alone (without Ga) might also cause DNA cleavage. Model tests using OH radicals generated by H_2O_2 or other methods could help clarify the specific role of gallium versus pure OH radicals.
7. The DFT simulations use isolated Ga atoms, ignoring solvation effects and dynamic oxide surfaces. The authors should discuss how these might affect the calculated results.
8. The paper refers to "nuclease-mimicking activity," but, from a non-specialist perspective, it would be valuable to compare the results with actual enzymes using catalytic numbers or other relevant metrics.

(Remarks on code availability)

Version 1:

Reviewer comments:

Reviewer #2

(Remarks to the Author)
The authors have put a lot of effort into the revision. All concerns raised by this reviewer were carefully addressed. Publication can thus now be recommended.

(Remarks on code availability)

Reviewer #3

(Remarks to the Author)
From my point of view, the authors have made significant efforts to address the reviewers' comments. The mechanism of OH generation, the role of Ga and the cleavage mechanism are now clearer. I believe the manuscript can now be accepted for publication.

(Remarks on code availability)

Open Access This Peer Review File is licensed under a Creative Commons Attribution 4.0 International License, which permits use, sharing, adaptation, distribution and reproduction in any medium or format, as long as you give appropriate credit to the original author(s) and the source, provide a link to the Creative Commons license, and indicate if changes were made.
In cases where reviewers are anonymous, credit should be given to 'Anonymous Referee' and the source.
The images or other third party material in this Peer Review File are included in the article's Creative Commons license, unless indicated otherwise in a credit line to the material. If material is not included in the article's Creative Commons license and your intended use is not permitted by statutory regulation or exceeds the permitted use, you will need to obtain permission directly from the copyright holder.
To view a copy of this license, visit https://creativecommons.org/licenses/by/4.0/

Reviewers' comments:

Reviewer #1 (Remarks to the Author):

The authors report an interesting discovery that the nuclease-like activity of Ga droplets with tunable and programmable capabilities, and a proposed mechanism involving nucleotide-biased adsorption and hydroxyl radical–assisted phosphodiester hydrolysis. This study is of significance for the development of artificial nuclease-mimicking systems. Thus, I would like to recommend the publications of this work. Following please find some minor issues.

1-1. It is intriguing that the nuclease-mimicking activity is highly dependent on the liquid state of Ga, as demonstrated in Figure 2c and d. What about the performance of solid-state Ga nanoparticles?

Response: We thank the reviewer for this insightful question regarding the physical state of Ga on its nuclease-mimicking activity. To address this, we conducted additional control experiments using solid-state Ga particles. As shown in the updated Fig. 2c, solid-state Ga particles exhibited negligible cleavage efficiency compared to its liquid-state counterpart. These results, alongside data from bulk liquid Ga, confirm that the nuclease-mimicking activity is strictly dependent on the liquid state of the metal.

We have updated the Main manuscript to include this discussion and provided a detailed protocol for the solid-state control experiments in Supplementary Method 2. The relevant revisions in manuscript and Supplementary Information are highlighted below:

Main manuscript (Page 2)

"This nuclease-mimicking activity is strongly dependent on the liquid state of Ga, as solid Ga, either in nanoparticle form (Fig. 2c) or as bulk material (Fig. 2d), exhibits negligible cleavage efficiency (Supplementary Method 2; Supplementary Fig. 4)."

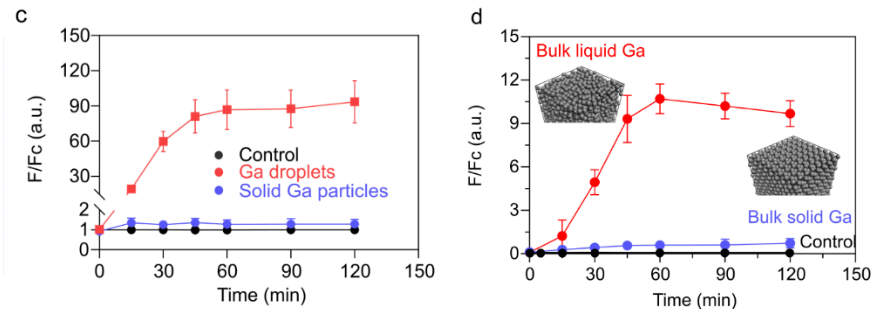

Fig.2 c, d. Time-dependent fluorescence of TR-ssDNA reporters incubated with **(c)** Ga particles and **(d)** bulk Ga in either liquid or solid form.

Supplementary Method 2:

"For Ga droplets, suspensions stored in ethanol (EtOH,1 mg mL^{-1}) were placed at -30 °C for one week to eliminate supercooling and ensure complete solidification. The solidified Ga droplets were collected by centrifugation at 4 °C and then introduced into the TR-ssDNA reporter solution. All other experimental conditions were identical to those used for liquid-state Ga droplets."

1-2. How stable are the Ga droplets during the process? Were there any precipitants observed, or did the droplet size change over time?

Response: We thank the reviewer's inquiry regarding the stability and morphological evolution of Ga droplets during the cleavage process. To address this, we performed detailed SEM imaging and XPS analysis on Ga droplets incubated with DNA in both EtOH and in deionized (DI) water.

Our findings indicate that:

In EtOH, Ga droplets remain structurally stable over long periods. While the oxide layer gradually thickens (as confirmed by XPS and slightly less rounded SEM morphologies), the metallic Ga^0 core is preserved, allowing the droplets to retain nuclease-mimicking activity for over a month (see Supplementary Fig. 16–17).

In aqueous environments, Ga droplets undergo a time-dependent transformation. While they initially sediment due to their higher density than water, the DNA cleavage activity remains high. Prolonged exposure (24h) leads to the transformation of spherical Ga droplets into rod-shaped GaOOH structures (see Supplementary Fig. 19).

The manuscript and Supplementary Information have been revised to include these mechanistic insights and copied here for your convenience:

Main manuscript (Page 7):

"To validate this tunneling-dependent activity, we compared Ga droplets with varying degrees of oxide thicknesses. We first examined samples stored in EtOH for one day *vs* one month. X-ray photoelectron spectroscopy (XPS) analysis revealed that while Ga droplets aged for one month retain a dominant metallic Ga^0 core, they exhibited a significantly higher Ga^{3+} contribution compared to the one-day sample (Supplementary Fig. 16). This chemical shift was also concurrent with SEM observations of less rounded morphologies, indicating thickened oxide layers. Functionally, the DNA cleavage activity of the one-month-old droplets remained detectable but was reduced compared to freshly prepared droplets (Supplementary Fig. 17). This demonstrates that while a thickened oxide layer substantially suppresses electron flux, it does not achieve total passivation."

Main manuscript (page 17):

Characterization of Ga droplets (Method 2)

"XPS measurements were carried out using a SPECS PHOIBOS 100 instrument with a monochromatic Al Kα source to characterize surface chemical composition**."**

Supplementary Discussion 3:

"Upon the transfer from EtOH to DI water, the formation of ·OH is accompanied by the oxidation of metallic (Ga^0) into Ga. After 24 h in DI water (Supplementary Fig. 19), SEM images reveal a gradual morphological transformation of Ga droplets into rod-like oxide structures. Correspondingly, the Ga 3d X-ray photoelectron spectroscopy (XPS) spectrum exhibits a dominant peak at ~20 eV, characteristic of Ga^{3+} species**.** Together, these results indicate the formation of GaOOH [1-3]…"

"The above equation indicates that electron transfer from Ga droplets to dissolved oxygen in the presence of water is essential for ·OH generation."

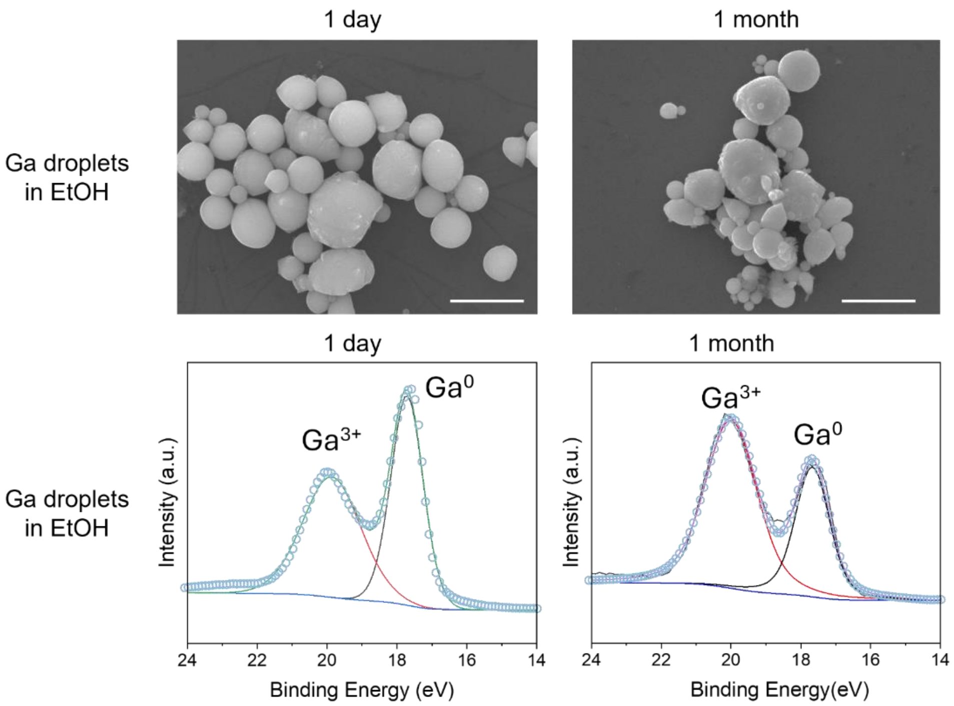

Supplementary Fig. 1. SEM images and Ga 3d XPS spectra of Ga droplets stored in EtOH for one day and one month. SEM reveals negligible morphological evolution during long-term storage. In the Ga 3d region, a dominant doublet centered at 17.8 ± 0.1 eV is assigned to metallic Ga^0, while a higher binding energy peaks at 20.0 ± 0.1 eV corresponds to oxidized Ga^{3+} species, consistent with the presence of a thin surface oxide layer. The relative intensities indicate that metallic Ga^0 still remains the one of the major chemical states after prolonged storage in EtOH, with limited surface oxidation.

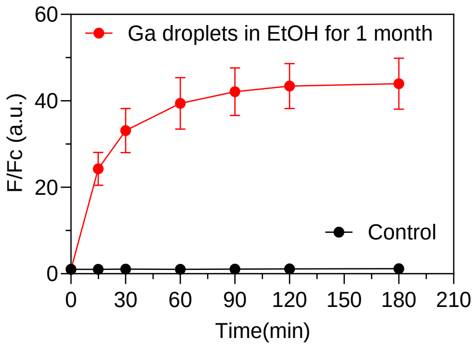

Supplementary Fig. 2. Time-dependent fluorescence of TR-ssDNA reporters incubated with Ga droplets stored in EtOH for one month, showing that long-term stored Ga droplets retain nuclease-mimic activity."

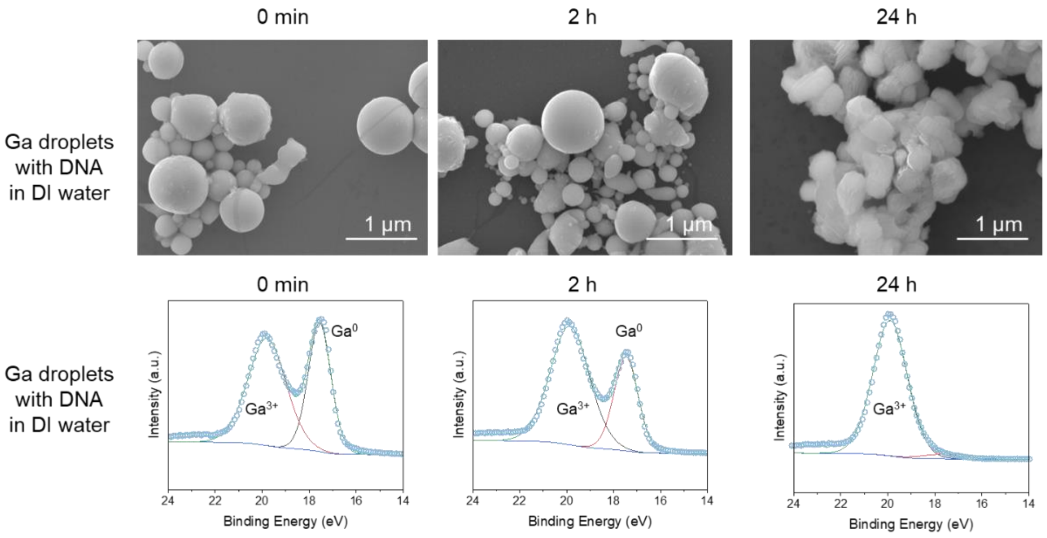

Supplementary Fig. 3. SEM images and Ga 3d XPS spectra of Ga droplets incubated with the TR-ssDNA reporter for 0 min, 2 h, and 24 h; SEM images show a gradual transformation of spherical droplets into rod-like structures; XPS results show a progressive oxidation of metallic Ga^0, with Ga^0 no longer detectable after 24 h. Based on this characteristic morphology and prior reports, the oxidized phase is assigned to GaOOH[1-3].

1-3. What is the long-term performance of the Ga droplets? Are they reusable?

Response: We thank the reviewer for this practical question regarding the longevity and reusability of the Ga droplets. We have systematically evaluated the long-term performance and reusability of Ga droplets and integrated the findings into the revised manuscript and Supplementary Discussion 3 to provide a clearer mechanistic context.

As detailed in our response to **Q1-2**, the droplets remain stable and active for over a month when stored in ethanol, despite a slight thickening of the oxide layer. Furthermore, our results demonstrate that DNA cleavage activity is maintained for approximately three cycles in water with ssDNA TR-reporter (90 min per cycle, Supplementary Fig. 21). Although we did not implement complex regeneration protocols (such as acid/base washes or electrochemical reduction) in this study to keep the focus on the intrinsic

mechanism, Ga droplets are significantly more cost-effective than commercial nucleases (Supplementary Table 16). This makes the system economically viable even with a finite number of cycles.

The relevant revisions and additions are provided below for your convenience:

Supplementary Discussion 3:

"Because the oxidation of Ga droplets in aqueous environments is unavoidable, Ga droplets' ssDNA reporter cleavage activity could only be maintained for up to three cycles (each cycle ~ 90 min) (Supplementary Fig. 21). Previous studies have shown that surface Ga oxides can be removed or reduced through acidic or basic treatments, electrochemical reduction, followed by re-sonication to regenerate Ga droplets[2,3]. Although such regeneration strategies could, in principle, restore reactive Ga droplets for additional cleavage cycles, they were not investigated in the present work in order to maintain a clear focus on the intrinsic DNA cleavage mechanism. Moreover, Ga droplets are considerably less expensive than commercial nucleases (as presented in Supplementary Table 16), such that high recyclability is not a critical requirement for their practical use."

Supplementary Figures:

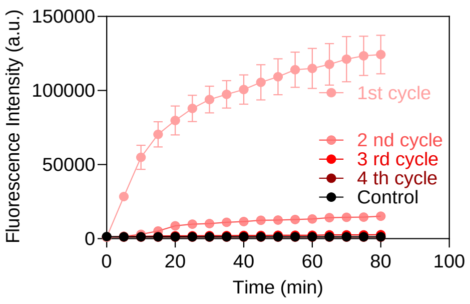

Supplementary Fig. 21. The cleavage effect of Ga droplets on TR-ssDNA reporters after reusing.

Supplementary Tables:

Supplementary Table 16:

Supplementary Table 16. Comparison between Ga droplets as nuclease mimics and commercial nucleases.

*The information was compiled from the official product manuals of Sigma-Aldrich, New England Biolabs, and Thermo Fisher Scientific.

	S1 Nuclease	Benzonase	DNase I	Ga droplets (~330 nm)
Description & Source	endonuclease from *Aspergillus* (fungal)	Broad-spectrum endonuclease from *Serratia marcescens* (bacterial)	Non-specific deoxyribonuclease from bovine pancreas	Metallic -Oxide micro/nanodroplets
Substrate Specificity	ssDNA > >dsDNA; Endonuclease	dsDNA, ssDNA; Endonuclease	dsDNA (100-500 x over ssDNA); length > 6bp; Endonuclease	dsDNA, ssDNA, Endonuclease-like/nuclease-mimic cleavage
Temperature	Optimal at ~37 °C.	Optimal temp 37 °C (active 0–42 °C).	Optimal at ~37 °C.	Broad temperature tolerance (0–

				70 °C); cleavage activity
Ion tolerance	Salt Inhibition>100 mM monovalent salts (Na+, K+) Phosphate Inhibition>1 mM	Salt Inhibition>300 mM monovalent salts (Na+, K+) Phosphate Inhibition>20 mM	Salt Inhibition>20 mM. Phosphate Inhibition>1 mM	Salt Inhibition>300 mM monovalent salts (Na+, K+)；Phosphate Inhibition>1 mM； can be recovered
EDTA tolerance	No	No	No	Yes
Price	Sigma_N5661-50KU 401 AUD	Sigma_E8263-5KU 358 AUD	Biolabs_M0303S_1000U 152 AUD	~2.4 AUD / g
Cost required to achieve the effect in Supplementary Fig.39	0.033U/166 nM 0.145 AUD/µM ssDNA	0.033U/166 nM 0.0133 AUD/µM ssDNA	0.033U/166 nM 0.0277 AUD/µM ssDNA	0.033U/166 nM 0.00145 AUD/µM ssDNA

1-4. How about the performance of nuclease activity of Ga droplet as compared with traditional method?

Response: Thank you for this important question. To evaluate the performance of Ga droplets, we compared them with three widely used commercial nucleases, including S1 nuclease, Benzonase, and DNase. Our comparative analysis (detailed in Supplementary Method 12, Supplementary Fig. 39 and Supplementary Table 16) demonstrates that Ga droplets are a robust and versatile nuclease-mimic platform.

Key findings include: 1) High performance: Ga droplets (~250 nm) match the efficiency of S1 nuclease and outperform Benzonase and DNase I under standard conditions. 2) Extreme robustness: Unlike traditional enzymes, they require no cofactors and remain active across high temperatures (up to 70 °C) and tolerate EDTA. 3) External control: Their activity can be uniquely restarted or boosted in inhibitory environments (like PBS) using 808 nm laser irradiation. 4) economic advantage: They provide comparable results at roughly 1% of the cost of commercial protein-based nucleases.

The following discussion and summary table have been revised or added to the Main manuscript and Supplementary information:

Main manuscript (page 14):

"Furthermore, systematic comparisons in both DI water and PBS against commercial nucleases (S1 nuclease, Benzonase, and DNase I; Supplementary Fig. 39; Supplementary Table 16); Supplementary Method 12) established Ga droplets as a distinct and efficient nuclease-mimic platform. In DI water, Ga droplets (0.1 mg mL $^{-1}$, ~330 nm) achieved approximately one-third of the cleavage efficiency of S1 nuclease, while size reduction to ~250 nm enabled cleavage efficiencies comparable to S1 and exceeding those of Benzonase and DNase I. In PBS, phosphate ions suppressed the activity of both S1 nuclease and Ga droplets, whereas Benzonase retained partial activity; critically, unlike protein enzymes, Ga droplets can be reactivated and even outperform commercial nucleases upon external stimulation, exemplified here by 808 nm laser irradiation. In dsDNA reporter assays, S1 nuclease showed no activity and DNase I failed to cleave short dsDNA fragments (<5-6 bp), consistent with its intrinsic substrate and length constraints[4], whereas Ga droplets maintained endonuclease-like activity across ssDNA and dsDNA substrates. Beyond their tunable activity (Fig. 5a), wide temperature ranges (Fig.5f; Supplementary Fig.3), high ionic strength (Supplementary Fig. 33), and EDTA-containing

environments (Fig. 3b), Ga droplets achieve comparable cleavage efficiencies at ~1% of the cost. Importantly, in contrast to many radical-generating artificial nucleases that cause extensive base oxidation and sugar-ring degradation[5,6], Ga droplets predominantly yield hydrolysis-like cleavage products, preserving nucleobase and sugar-ring integrity while selectively cleaving the phosphodiester backbone.

Together, the sensitivity of Ga droplets to inhibitors such as phosphate, together with their reactivation by physical and chemical triggers, including pH adjustment, bicarbonate addition, and light irradiation *via* modulation of surface oxide integrity, highlights a unique, switchable, and programmable nuclease-mimic behavior."

Supplementary Method:

Supplementary Method 12. Commercial nuclease activity

"S1 nuclease, Benzonase, and DNase I digestions were performed according to the manufacturers' instructions. S1 nuclease reactions were conducted in acetate-based buffer containing zinc ions (Zn^{2+}), while Benzonase and DNase I reactions were in Tris-based buffers supplemented with magnesium ions (Mg^{2+}) and calcium ion (Ca^{2+}) for DNase I). All reactions were incubated at 37 °C for the indicated time. Reporter DNA concentrations were identical to those used in the Ga-droplet experiments (100 μL, 166 nM per well; 0.024 μg ssDNA per well). Nucleases were added at 0.033 U per well (1 U defined as activity per 1 μg DNA)."

Supplementary Figures:

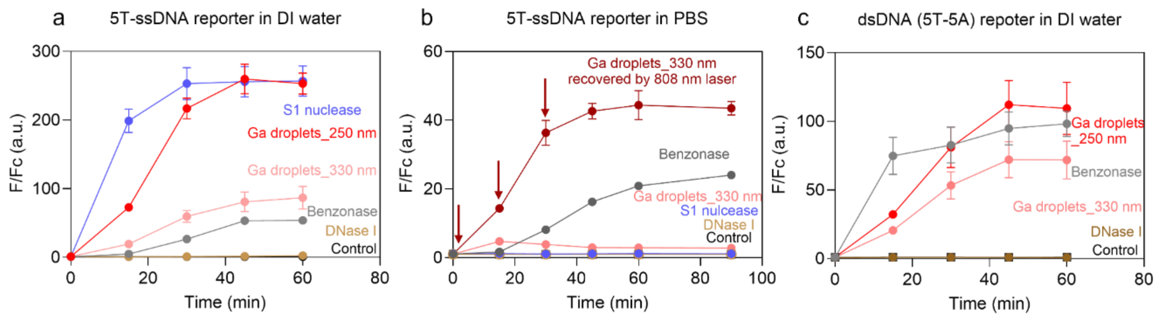

Supplementary Fig. 39. Comparison of the DNA cleavage activity of Ga droplets and commercial nucleases (S1 nuclease, Benzonase, DNase I). Ga droplets with mean diameters of ~330 nm and ~250 nm were synthesized by probe sonication for 30 min and 120 min, respectively.

Supplementary Tables:

Supplementary Table 16. Comparison between Ga droplets as nuclease mimics and commercial nucleases. *The information was compiled from the official product manuals of Sigma-Aldrich, New England Biolabs, and Thermo Fisher Scientific.

	S1 Nuclease	Benzonase	DNase I	Ga droplets (~330 nm)
Description & Source	endonuclease from *Aspergillus* (fungal)	Broad-spectrum endonuclease from *Serratia marcescens* (bacterial)	Non-specific deoxyribonuclease from bovine pancreas	Metallic -Oxide micro/nanodroplets
Substrate Specificity	ssDNA > >dsDNA; Endonuclease	dsDNA, ssDNA; Endonuclease	dsDNA (100-500 x over ssDNA); length > 6bp; Endonuclease	dsDNA, ssDNA, Endonuclease-like/nuclease-mimic cleavage
Temperature	Optimal at ~37 °C.	Optimal temp 37 °C (active 0–42 °C).	Optimal at ~37 °C.	Broad temperature tolerance (0–70 °C); cleavage activity
Ion tolerance	Salt Inhibition>100 mM monovalent salts (Na+, K+) Phosphate Inhibition>1 mM	Salt Inhibition>300 mM monovalent salts (Na+, K+) Phosphate Inhibition>20 mM	Salt Inhibition>20 mM. Phosphate Inhibition>1 mM	Salt Inhibition>300 mM monovalent salts (Na+, K+)； Phosphate Inhibition>1 mM； can be recovered
EDTA tolerance	No	No	No	Yes
Price	Sigma_N5661-50KU 401 AUD	Sigma_E8263-5KU 358 AUD	Biolabs_M0303S_1000U 152 AUD	~2.4 AUD / g
Cost required to achieve the effect in Supplementary Fig.39	0.033U/166 nM 0.145 AUD/µM ssDNA	0.033U/166 nM 0.0133 AUD/µM ssDNA	0.033U/166 nM 0.0277 AUD/µM ssDNA	0.033U/166 nM 0.00145 AUD/µM ssDNA

Reviewer #2 (Remarks to the Author):

This manuscript submitted by Kalantar-Zadeh and Zhang reports on nucleolytic activity of liquid Ga. Whereas the approach is interesting, the work or at least its description within the manuscript is not conclusive even though a lot of different experiments were carried out. The conclusions "bioinspired catalysis in abiotic systems, thereby broadening the design space for artificial enzymes beyond traditional coordination complexes "and "compared with other ligands and cofactors dependent on artificial metallonuclease, liquid Ga droplets uniquely serve as both the catalytic center and structural framework, forming a scaffold- free, metal-only platform. Their activity is tunable via synthetic strategies (e.g., droplet size, alloy composition, etc.) and dynamically controlled by external stimuli such as pH, light, sonication, and chemical additives. sound promising, however, these claims are not fulfilled based on the data shown in the manuscript. Other effects that might lead to the observed data have not been taken into account.

Major issues:

2-1. Abstract: Phosphodiester hydrolysis is claimed to be carried out by hydroxyl radicals. The authors should refer to the literature where cleavage of phosphate esters is definitively not carried out by hydroxyl radicals. These highly reactive species rather attack the sugars/bases of DNA. The mechanism which is supported by DFT is not convincing (Figure 12 in the SI).

Response: We thank the reviewer for this critical assessment of the proposed mechanism. We completely agree that in classical systems (such as radiation or Fenton chemistry), freely diffusing ·OH radicals are highly non-specific and primarily cause damage to nucleobases and sugar moieties rather than selective phosphodiester scission. However, our experimental evidence suggests that the Ga-droplet system operates differs significantly from Fenton chemistry.

We performed comparative high-resolution mass spectrometry (MS) analysis of DNA fragments generated by Ga droplets versus those produced by a standard Fenton reaction (Fe^{2+}/H_2O_2). As detailed in Figure 3, Supplementary Fig.26, the cleavage patterns are strikingly different.

Fenton reaction produced a distribution of fragments with significant base damage and multiple oxidative modifications, characteristic of non-specific radical attack (Figure 3d; Supplementary Fig.26; Supplementary Table 12), while Ga droplets yielded a much cleaner fragment distribution with terminal chemistries (e.g., 3'-phosphate and 5'-hydroxyl groups) consistent with scission of the phosphodiester backbone (Figure 3b,c; Supplementary Fig.24).

We hypothesize that this cleavage process is an interfacial event rather than a bulk solution reaction. The surface of the Ga droplet appears to act as a scaffold that orients the DNA molecules (Fig. 3i, 4f), facilitating a directed attack on the backbone (Fig. 4g). This confined radical chemistry, occurring at the Ga droplets interface, effectively channels the reactivity toward the phosphodiester bonds and prevents the typical oxidation of bases or sugar rings by free-diffusing radicals in a homogeneous medium.

We have revised the manuscript to emphasize this interfacial model and have included comparative MS data to provide experimental validation beyond the DFT simulations. For your convenience, the added discussions and figures are provided below:

Main manuscript (Page 9):

"To elucidate the DNA cleavage mechanism of Ga droplets, we compared their fragmentation patterns with those generated by a classical ·OH-based Fenton reaction using high-resolution mass spectrometry

(MS) (Fig. 4a-e; Method 8; Supplementary Method 5). Relative to the control, both Ga-treated and Fenton-treated samples showed a marked decrease in the intact 5T ssDNA ion (*m/z* 363.56, z = -4), accompanied by the appearance of 4T fragments at *m/z* 576.10 and 616.09 (z = -2) (Supplementary Figs. 22, 23; Supplementary Tables 5-6). These ions correspond to two specific cleavage products: a 4T fragment bearing two hydroxyl termini and a second fragment containing one hydroxyl and one phosphate monoester terminus (Supplementary Fig. 24; Supplementary Tables 7-10; Supplementary Discussion 4).

To assess the involvement of oxidative pathways, expected fragments arising from typical metallonuclease mechanisms, including hydrogen abstraction and nucleobase oxidation, were predicted and used for targeted *m/z* screening (Supplementary Fig. 25; Supplementary Tables 11, 12). Oxidative base-modified fragments were detected in the Fenton reaction, whereas no such species were observed in Ga-treated samples (Supplementary Fig. 26). This agrees with established Fenton chemistry, in which ·OH radicals preferentially attack nucleobases, causing secondary cleavage of the phosphodiester backbone[5,7]. In contrast, Ga droplets exclusively generated phosphodiester bond-derived cleavage products, with no detectable nucleobase or sugar-ring oxidation, showing similar hydrolytic nuclease activity. These results further provide evidence that Ga droplets cleave DNA *via* an OH-assisted hydrolytic mechanism targeting the phosphodiester bond, rather than through oxidative degradation characteristics of classical Fenton systems."

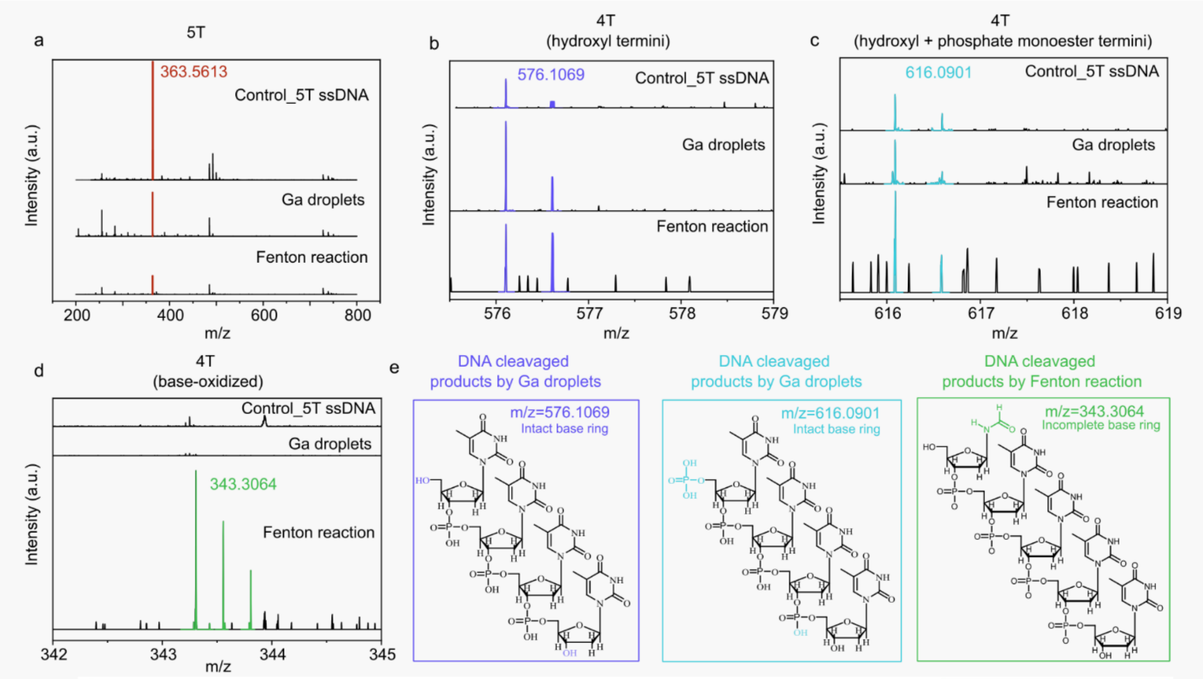

Fig. 4 | Mechanism of Ga droplets' nuclease-mimicking effect: ROS-assisted hydrolytic cleavage activity. a-d. MS analysis of 5T ssDNA fragments after Ga droplets or Fenton reaction treatment: full spectrum showing fragmentation pattern with 5T ssDNA peaks; **b**. zoom-in of 4T fragments with two hydroxyl termini; **c**. zoom-in of 4T fragment with hydroxyl and phosphate monoester termini; **d**. zoom-in of 4T fragment with oxidized base. In control samples, some hydrolytic-like peaks were observed due to in-source decay and high-voltage ionization[8,9]. The analysis of the 3T-to-1T fragments is provided in Supplementary Discussion 4. **e.** 4T fragment structures corresponding to m/z in b-d.

Supplementary Figures:

Product 1 ↓:

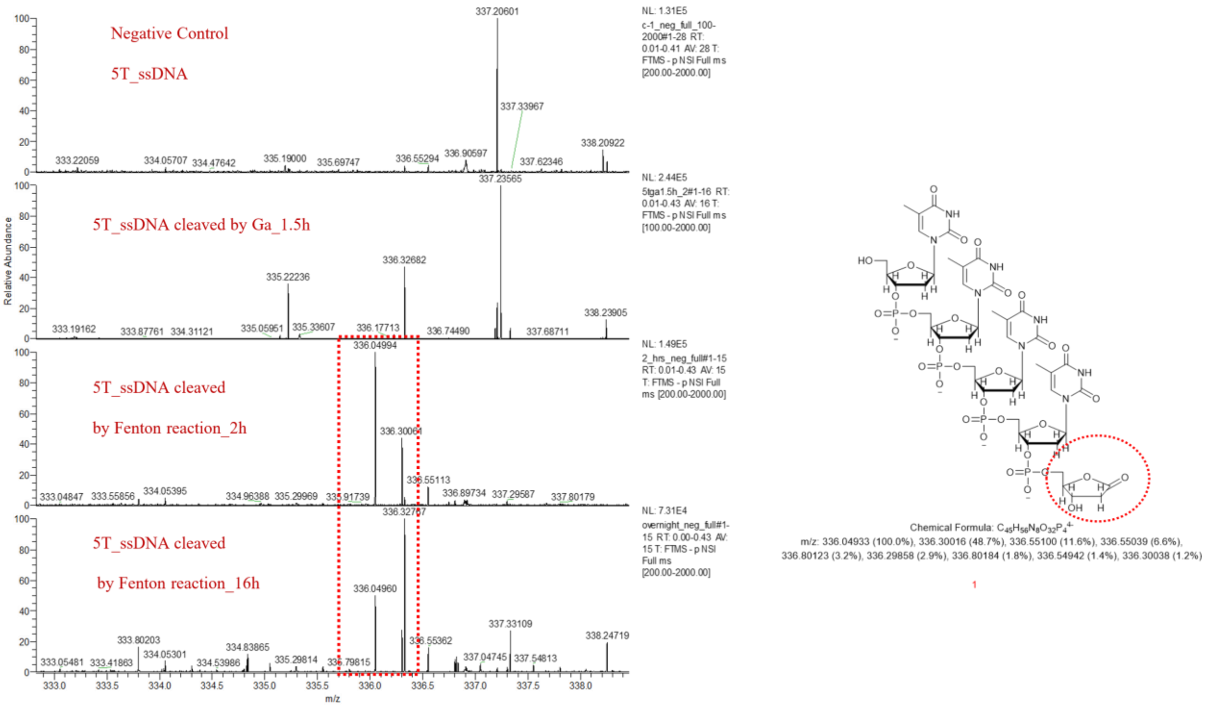
Negative Control
5T_ssDNA
5T_ssDNA cleaved by Ga_1.5h
5T_ssDNA cleaved
by Fenton reaction_2h
5T_ssDNA cleaved
by Fenton reaction_16h
Relative Abundance
m/z
NL: 1.31E5
NL: 2.44E5
NL: 1.49E5
NL: 7.31E4
Chemical Formula: C45H56N8O32P4 4-
m/z: 336.04933 (100.0%), 336.30016 (48.7%), 336.55100 (11.6%), 336.55039 (6.6%), 336.80123 (3.2%), 336.29858 (2.9%), 336.80184 (1.8%), 336.54942 (1.4%), 336.30038 (1.2%)
1

Product 2 ↓:

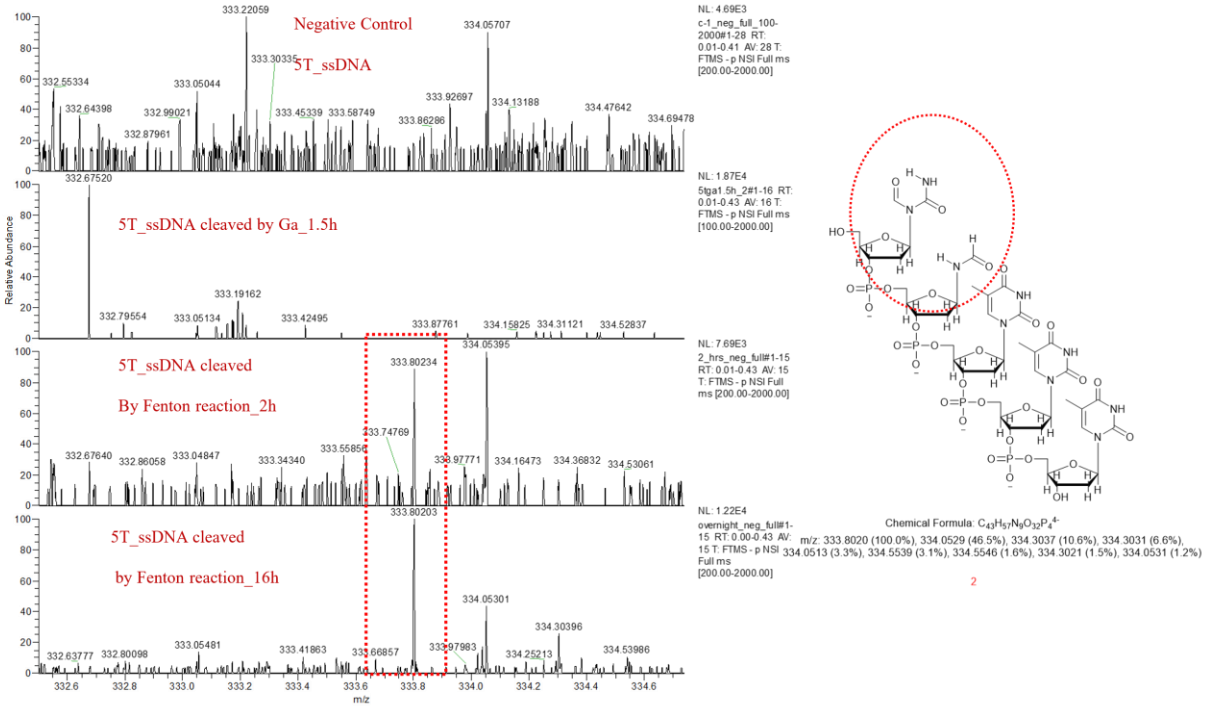
Negative Control
5T_ssDNA
5T_ssDNA cleaved by Ga_1.5h
5T_ssDNA cleaved
By Fenton reaction_2h
5T_ssDNA cleaved
by Fenton reaction_16h
Relative Abundance
m/z
NL: 4.69E3
NL: 1.87E4
NL: 7.69E3
NL: 1.22E4
Chemical Formula: C43H57N9O32P4 4-
m/z: 333.8020 (100.0%), 334.0529 (46.5%), 334.3037 (10.6%), 334.3031 (6.6%), 334.0513 (3.3%), 334.5539 (3.1%), 334.5546 (1.6%), 334.3021 (1.5%), 334.0531 (1.2%)
2

Product 3↓:

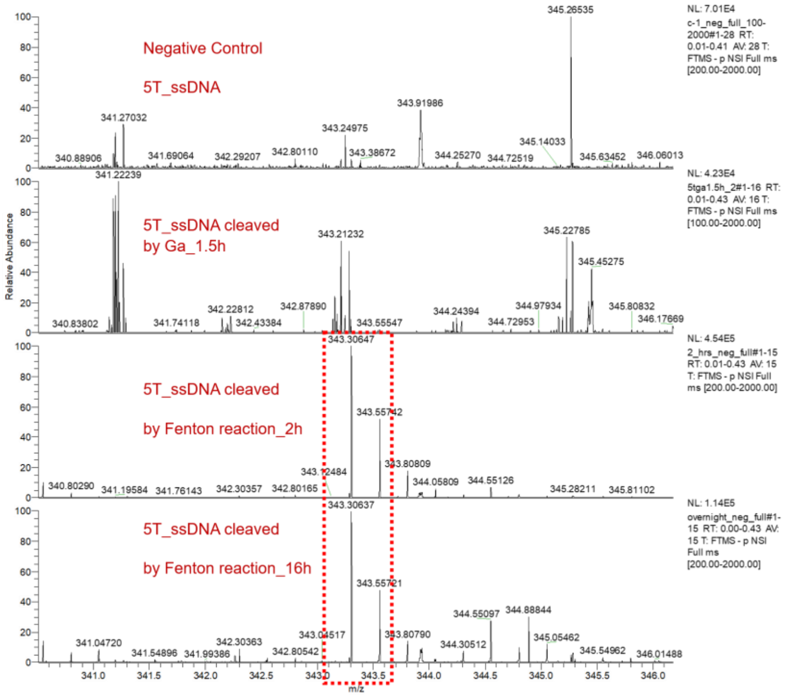

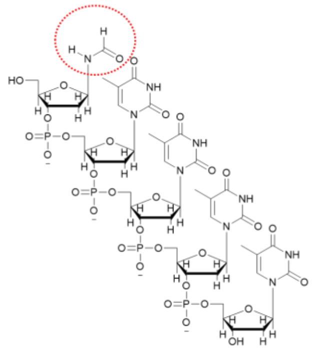

Chemical Formula: $C_{46}H_{59}N_9O_{32}P_4^{4-}$

m/z: 343.3060 (100.0%), 343.5568 (49.8%), 343.8076 (12.1%), 343.8070 (6.6%), 343.5552 (3.3%), 344.0579 (3.3%), 344.0585 (1.9%), 343.8060 (1.6%), 343.5570 (1.2%)

3

Product 4 ↓:

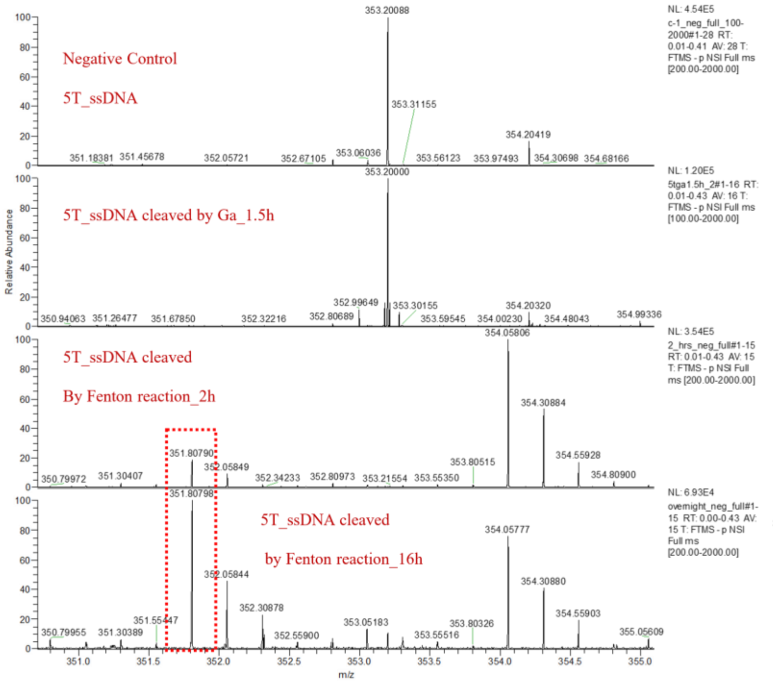

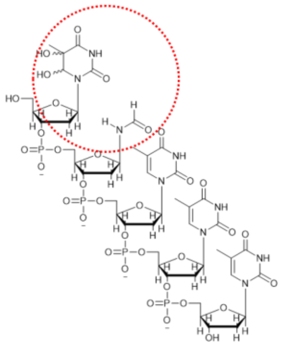

Chemical Formula: $C_{46}H_{61}N_9O_{34}P_4^{4-}$

m/z: 351.80733 (100.0%), 352.05817 (49.8%), 352.30901 (12.1%), 352.30839 (7.0%), 352.55923 (3.5%), 352.05659 (3.3%), 352.55985 (1.9%), 352.30743 (1.6%), 352.05839 (1.3%)

4

Product 5 ↓:

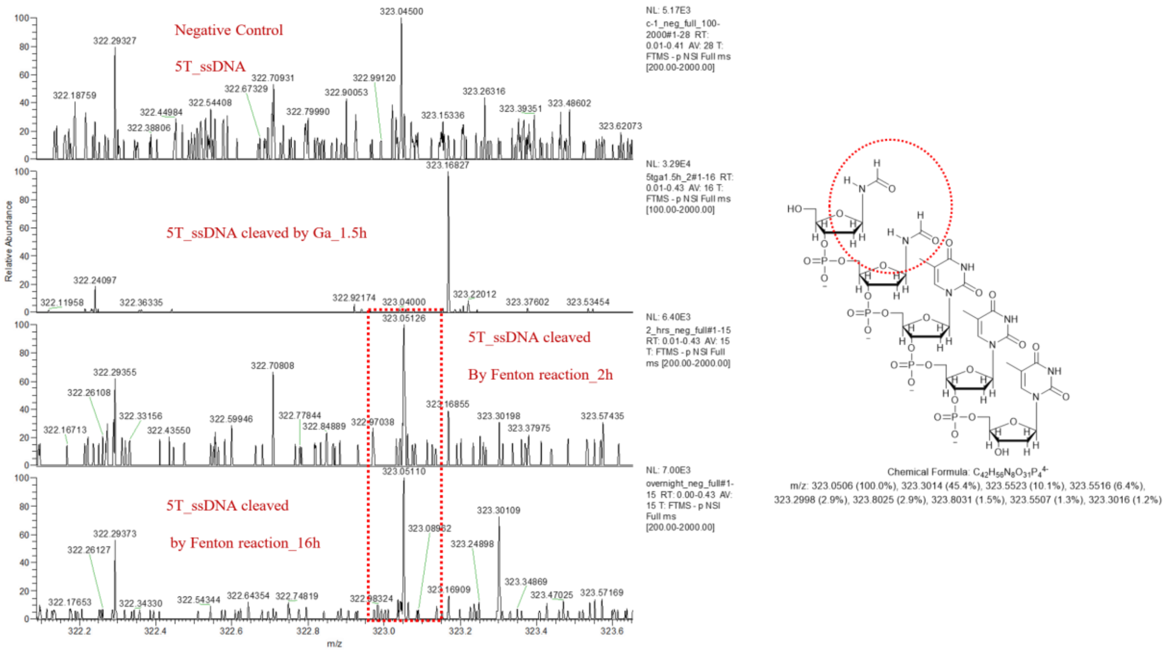

Supplementary Fig. 26. The mass spectra of oxidative fragments derived from 5T ssDNA, observed only under Fenton-mediated cleavage conditions, together with their proposed structures, are shown; the positions of fragmented nucleobases may vary within the 5T sequences.

Supplementary Tables:

Supplementary Table 12. Calculated m/z values of theoretical fragments: product 1 to 5 in Supplementary Fig. 26.

These tables summarize the screened data from this procedure. Additional data are available upon request; representative main peaks and structures are shown in **Supplementary Fig. 26**.

Supplementary Table 12-1. (only hydroxyl-group residues at the DNA fragment termini)

Product 1	Expected MS	-H'	-2H+Na'	-2H/2'	-3H+Na'	-3H/3	-4H+Na'	-4H'
5T	1348.2264	1347.2191	1369.2016	673.1059	684.09715	448.4015	455.729	336.0493
4T	1044.18036	1043.17306	1065.15556	521.08288	532.07413	347.05282		
3T	740.13432	739.12702	761.10952	369.05986				
2T	436.08828	435.08098						
T	NA							
Product 2	Expected MS	-H'	-2H+Na'	-2H/2'	-3H+Na'	-3H/3	-4H+Na'	-4H'
5T	1339.2373	1338.23	1360.2125	668.61135	679.6026	445.40513	452.73263	333.80202
4T	1035.19126	1034.18396	1056.16646	516.58833	527.57958	344.05645		
3T	731.14522	730.13792	752.12042	364.56531				

2T	427.09918	426.09188						
T	NA							
Product 3	Expected MS	-H'	-2H+Na'	-2H/2'	-3H+Na'	-3H/3	-4H+Na'	-4H'
5T	1377.253	1376.2457	1398.2282	687.6192	698.61045	458.07703	465.40453	343.30595
4T	1073.20696	1072.19966	1094.18216	535.59618	546.58743	356.72835		
3T	769.16092	768.15362	790.13612	383.57316				
2T	465.11488	464.10758						
T								
Product 4	Expected MS	-H'	-2H+Na'	-2H/2'	-3H+Na'	-3H/3	-4H+Na'	-4H'
5T	1411.2584	1410.2511	1432.2336	704.6219	715.61315	469.41217	476.73967	351.8073
4T	1107.21236	1106.20506	1128.18756	552.59888	563.59013	368.06349		
3T	803.16632	802.15902	824.14152	400.57586				
2T	499.12028	498.11298						
T	NA							
Product 5	Expected MS	-H'	-2H+Na'	-2H/2'	-3H+Na'	-3H/3	-4H+Na'	-4H'
5T	1296.2315	1295.2242	1317.2067	647.10845	658.0997	431.06987	438.39737	323.050575
4T	992.18546	991.17816	1013.16066	495.08543	506.07668	329.72119		
3T	688.13942	687.13212	709.11462	343.06241				
2T	384.09338	383.08608						
T	NA							

Supplementary Table 12-2. (only one hydroxyl-group residue at the DNA fragment termini)

Product 1	Expected MS	-H'	-2H+Na'	-2H/2'	-3H+Na'	-3H/3
5T						
4T	1124.14666	1123.13936	1145.12186	561.06603	572.05728	373.7082533
3T	820.10062	819.09332	841.07582	409.04301		
2T	516.05458	515.04728				
T	212.00854	211.00124				
Product 2	Expected MS	-H'	-2H+Na'	-2H/2'	-3H+Na'	-3H/3
5T						
4T	1115.15756	1114.15026	1136.13276	556.57148	567.56273	370.7118867
3T	811.11152	810.10422	832.08672	404.54846		
2T	507.06548	506.05818				
T	NA	NA				
Product 3	Expected MS	-H'	-2H+Na'	-2H/2'	-3H+Na'	-3H/3
5T						
4T	1153.17326	1152.16596	1174.14846	575.57933	586.57058	383.3837867
3T	849.12722	848.11992	870.10242	423.55631		
2T	545.08118	544.07388				
T	241.03514	240.02784				

Product 4	Expected MS	-H'	-2H+Na'	-2H/2'	-3H+Na'	-3H/3
5T						
4T	1187.17866	1186.17136	1208.15386	592.58203	603.57328	394.71892
3T	883.13262	882.12532	904.10782	440.55901		
2T	579.08658	578.07928				
T	NA	NA				
Product 5	Expected MS	-H'	-2H+Na'	-2H/2'	-3H+Na'	-3H/3
5T						
4T	1072.15176	1071.14446	1093.12696	535.06858	546.05983	356.37662
3T	768.10572	767.09842	789.08092	383.04556		
2T	464.05968	463.05238				
T	NA	NA				

Supplementary Table 12-3. (two hydroxyl-group residues at the DNA fragment termini)

Product 1	Expected MS	-H'	-2H+Na'	-2H/2'
5T				
4T				
3T	900.06692	899.05962	921.04212	449.02616
2T	596.02088	595.01358		
T	291.97484	290.96704		
Product 2	Expected MS	-H'	-2H+Na'	-2H/2'
5T				
4T				
3T	891.07782	890.07052	912.05302	444.53161
2T	587.03178	586.02448		
T	NA	NA		
Product 3	Expected MS	-H'	-2H+Na'	-2H/2'
5T				
4T				
3T	929.09352	928.08622	950.06872	463.53946
2T	625.04748	624.04018		
T	321.00144	319.99364		
Product 4	Expected MS	-H'	-2H+Na'	-2H/2'
5T				
4T				
3T	963.09892	962.09162	984.07412	480.54216
2T	659.05288	658.04558		
T	NA	NA		
Product 5	Expected MS	-H'	-2H+Na'	-2H/2'
5T				
4T				
3T	848.07202	847.06472	869.04722	423.02871
2T	544.02598	543.01868		
T	NA	NA		

2-2. Liquid Ga is claimed "as catalytic centers and as cofactors, forming a ligand- and cofactor-free artificial nuclease platform". Clarify the term "cofactor". In the context it is used here, "catalytic center" should be synonymous.

Response: We thank the reviewer for this point. We agree that in the context of our system, the terms "catalytic center" and "cofactor" could be seen as synonymous, and we appreciate the opportunity to clarify the unique role of the liquid metal.

In conventional enzymatic and artificial metallonuclease systems, a *cofactor* is an auxiliary species required to assist a separate site. In our system, no such external cofactors are involved. To avoid confusion and to accurately reflect our system, we now describe the Ga droplets as an integrated platform that serves both structural and reactive roles.

The revised manuscript now reads (Main manuscript, page 1):

"Compared with previously reported artificial metallonuclease, the liquid Ga uniquely integrates its oxide layer for substrate adsorption and its Ga^0 core with electrons as cleavage active center, forming a ligand- and cofactor-free artificial nuclease platform."

2-3. Figure 1 c is completely hypothetical. Do the authors really know that the Ga droplets stay like this when/if they enter the cells? Also, how would they be taken up, some droplets are > 500 nm. Would phagocytosis be a potential mechanism?

Response: Thank you for raising these important questions. We would like to clarify that no cell-based experiments were performed in this study. All experiments were conducted in acellular systems, as explicitly stated in the Experimental Section (Method 3,4,5)

Accordingly, Figure 1c is intended solely as a conceptual schematic illustrating of a potential future application of Ga droplets within synthetic artificial cell models, such as lipid vesicles or protocells. This distinction is supported by the labels in the figure, which specify an "Artificial phospholipid membrane" and "Biomimicry". In such controlled synthetic systems, Ga droplets would typically be encapsulated during the membrane assembly process rather than relying on biological uptake mechanisms like phagocytosis.

While we demonstrate that Ga droplets can be synthesized with smaller diameters (50~250 nm) through optimized sonication, we agree that for any future live-cell applications, size and internalization pathways would be critical factors to investigate. However, systematic studies of cellular internalization, intracellular stability, and *in vitro* or *in vivo* behavior were beyond the scope of this fundamental mechanistic study. The scope of the present work is to identify and mechanistically elucidate the ·OH-mediated DNA cleavage activity of Ga droplets under aqueous conditions.

To prevent any further misunderstanding, we have revised the caption for Figure 1c (Main manuscript page 3) to explicitly state: "Schematic illustration of the proposed potential application of Ga droplets as artificial nucleases within a synthetic artificial cell environment".

2-4. Experiments for DNA cleavage have apparently been carried out at room temperature. Since the melting point of Ga is about 29 °C only, it is very important to specify what "room temperature" is. Depending on lab air conditioning and season r.t. might vary between 20 and 30 °C.

Response: Thank you for this important comment. All DNA cleavage experiments were conducted at a controlled laboratory temperature of approximately 22 °C. Although the bulk melting point of Ga is 29.8 °C, Ga droplets can remain in the liquid state well below this temperature due to a pronounced supercooling effect [10]. This behavior is described in detail in the Supporting Information (Supplementary Method 2) Specifically, bulk Ga can remain liquid below its melting point once melted, which we confirmed by differential scanning calorimetry (DSC) analysis (Supplementary Fig. 4), consistent with previous reports[11].

We have also added a corresponding statement to the Ga droplet preparation procedure (Method 1, Main manuscript page 17) to explicitly specify the experimental temperature and the role of supercooling:

"In addition, Ga exhibits a pronounced supercooling effect, allowing it to remain in the liquid state for extended periods below its melting point. Additional details are provided in Supplementary Method 2."

2-5. For a mechanism where hydroxyl radicals are involved, it is difficult to infer DNA cleavage solely from a DNA beacon cleavage process based on fluorescence (de)quenching; radicals may also attack the dyes.

Response: We thank the reviewer for this insightful concern regarding the potential for radical interference with fluorophore stability. We would like to clarify that in our reporting system, DNA cleavage is inferred from a quantifiable recovery of fluorescence intensity, rather than a quenching effect.

As illustrated in Fig. 1b and 1c, the commercial DNA beacon system maintains the fluorophore in a quenched state due to its close proximity to the quencher (within ~5 nucleotides, ~3 nm). Upon scission of the phosphodiester backbone by the Ga-droplet system, the physical separation of the fluorophore and quencher results in a measurable fluorescence recovery.

To rigorously exclude the possibility that ·OH directly affects the fluorophore, we performed control experiments incubating Ga droplets (which generate ·OH radicals) with free Texas Red dye in the absence of DNA (Supplementary Fig. 2). No significant change in fluorescence intensity was observed under these conditions, indicating that ·OH radicals do not induce spurious enhancement or oxidative bleaching of the dye itself.

In addition, the influence of external stimuli used in Fig. 6 on dye stability was further examined by additional control experiments and added into Supplementary Information. Together, these results confirm that the observed fluorescence increase originates from DNA cleavage rather than radical-induced modification of the fluorophore.

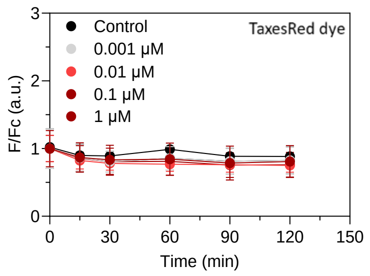

Supplementary Fig. 2. Time-dependent fluorescence of TexasRed dye incubated with Ga droplets to exclude the influence of Ga droplets on the TexasRed dye.

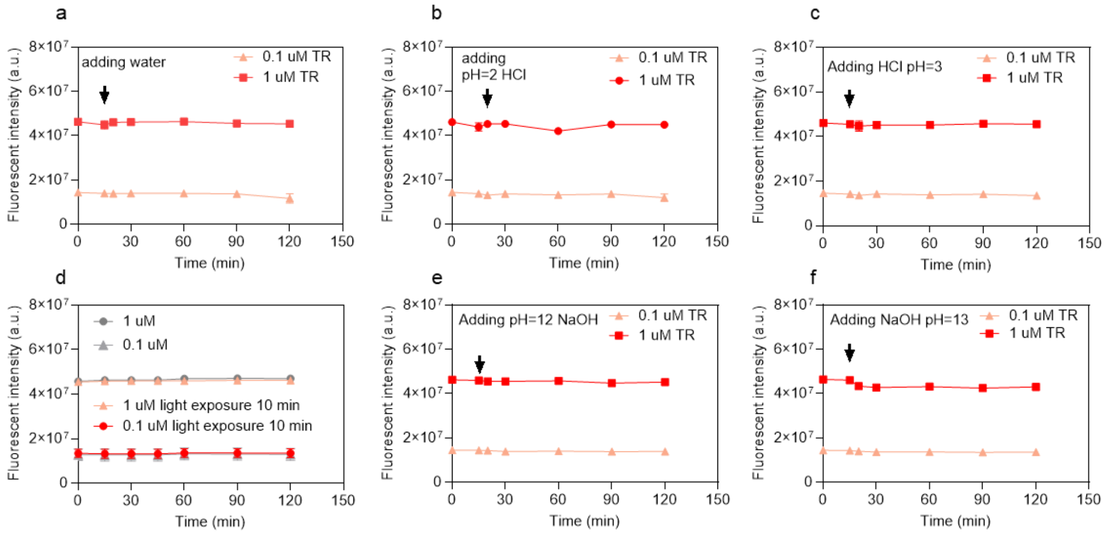

Supplementary Fig. 38. Influence of external stimuli on the Texas Red dye: (a) water, (b) pH 2 HCl, (c) pH 3 HCl, (d) light exposure for 10 min, (e) pH 12 NaOH, and (f) pH 13 NaOH; 10 µL was added to the 100 µL solution at the time indicated by the arrow.

2-6. The claim that Ga (0) is responsible for cleavage is difficult to conclude. In order to form reactive oxygen species, a change in oxidation state should be observed.

Response: Thank you for this important comment. To address this point, we performed SEM imaging and XPS analysis on Ga droplets incubated with DNA under cleavage conditions. These analyses reveal clear changes in the Ga oxidation state, evidenced by an increased Ga^{3+} contribution relative to metallic Ga^0, indicating oxidation of Ga during the reaction. The corresponding structural and chemical evolution of Ga droplets, together with their correlation to ·OH production mechanism and DNA cleavage activity, are discussed in detail in the response to Question 1-2.

The information relevant to this question in the Supplementary Information provided below for your convenience:

Supplementary Discussion 3:

"Upon the transfer from EtOH to DI water, the formation of ·OH is accompanied by the oxidation of metallic (Ga^0) into Ga. After 24 h in DI water (Supplementary Fig. 19), SEM images reveal a gradual morphological transformation of Ga droplets into rod-like oxide structures. Correspondingly, the Ga 3d X-ray photoelectron spectroscopy (XPS) spectrum exhibits a dominant peak at ~20 eV, characteristic of Ga^{3+} species**.** Together, these results indicate the formation of GaOOH [1-3]......

The above equation indicates that electron transfer from Ga droplets to dissolved oxygen in the presence of water is essential for ·OH generation and the subsequent DNA cleavage process."

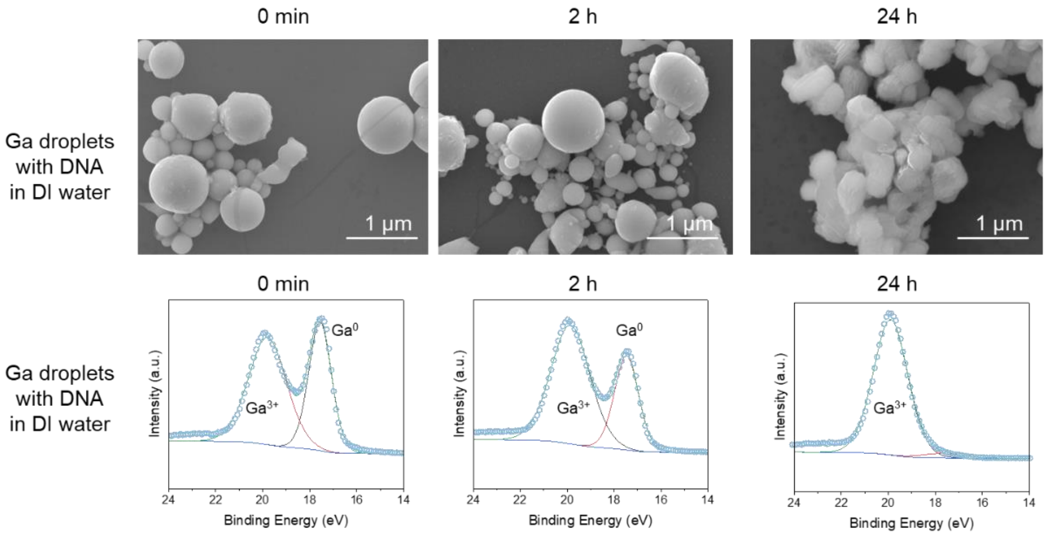

Supplementary Fig. 4. SEM images and Ga 3d XPS spectra of Ga droplets incubated with the TR-ssDNA reporter for 0 min, 2 h, and 24 h; SEM images show a gradual transformation of spherical droplets into rod-like structures; XPS results show a progressive oxidation of metallic Ga^0, with Ga^0 no longer detectable after 24 h. Based on this characteristic morphology and prior reports, the oxidized phase is assigned to GaOOH[1-3].

2-7. Figure 4: Why would phosphate (from the diester bond) bind to elemental Ga? Phosphate typically binds Ga (III). DFT is not conclusive, see above.

Response: Thank you for this important comment. We agree that phosphate groups preferentially bind Ga (III) species. In the present system, Ga (III) exists either as free Ga^{3+} species in solution or as surface Ga (III) sites within Ga oxides (Ga_2O_3 and GaOOH) formed on Ga droplets.

To further elucidate the adsorption of ssDNA by Ga droplets, classical molecular dynamics (MD) simulations were used to investigate the adsorption behaviour of ssDNA on Ga oxide surfaces (Ga_2O_3 and GaOOH) (Supplementary Discussion 5 and Supplementary Method 6). In parallel, density functional theory (DFT) calculations were performed to evaluate the interactions of ssDNA with an isolated Ga^0 atom and a Ga^{3+} ion (Supplementary Discussion 6 and Supplementary Method 7).

The discussion of the computational calculations has been revised in the manuscript to clarify the respective roles of Ga^0, Ga oxides, and Ga^{3+} species, as follows:

Main manuscript (page 9):

Computational Modeling of DNA Interfacial Adsorption

To further elucidate the role of the Ga interface, we employed multiscale computational approaches to examine the individual contributions of the species present in the system: metallic Ga^0, Ga oxide layer, and free Ga^{3+}. Classical molecular dynamics (MD) simulations were employed to investigate the adsorption behavior of ssDNA on Ga oxide surfaces (Supplementary Table 13; Supplementary Discussion 5; Supplementary Method 6). We evaluated two representative orientations: ssDNA aligned either parallel or perpendicular to the Ga oxide surface (Fig. 4f). The simulations indicate that, given the significant size disparity between the Ga droplets (average diameter ≈ 330 nm) and the ssDNA (~3 nm in length), the DNA molecules preferentially adopt a flat adsorption configuration. This lying-down

orientation on the relatively planar droplet surface facilitates simultaneous interfacial interactions across multiple molecular moieties of the DNA strand.

Density functional theory (DFT) was employed to characterize the adsorption affinity of ssDNA toward both Ga^0 atoms and Ga^{3+} ions (Supplementary Discussion 6). The simulations show that Ga^0 can absorb nucleobases, the sugar ring, and the phosphodiester bond (Supplementary Fig. 27a), mirroring the multi-site, whole-molecule adsorption observed in our MD simulations. Notably, the phosphate groups exhibit a higher adsorption affinity for Ga^0 than the nucleobase nitrogen sites, likely due to enhanced electrostatic and van der Waals interactions. In the presence of ·OH, the cleavage of this phosphate diester bond is energetically favorable (Supplementary Fig. 27b, c; Supplementary Discussion 6; Supplementary Method 7). In contrast, free Ga^{3+} -phosphate interactions lead to the formation of thermodynamically stable Ga-O bonds. These stale bonds effectively 'lock' the structure and do not promote cleavage, even in the presence of ·OH, thereby explaining why Ga^{3+} does not contribute to the system's nuclease-like activity (Supplementary Fig. 28). This finding is fundamentally consistent with our experimental observation in (Fig. 3b; Supplementary Fig. 9a).

Main manuscript (page 10):

Synergistic Mechanism of ·OH-Assisted Hydrolysis

….The details of a possible mechanism for Ga droplets' preference to T-T and A-A are provided in Supplementary Discussion 7, Supplementary Fig. 29 and Supplementary Method 8.

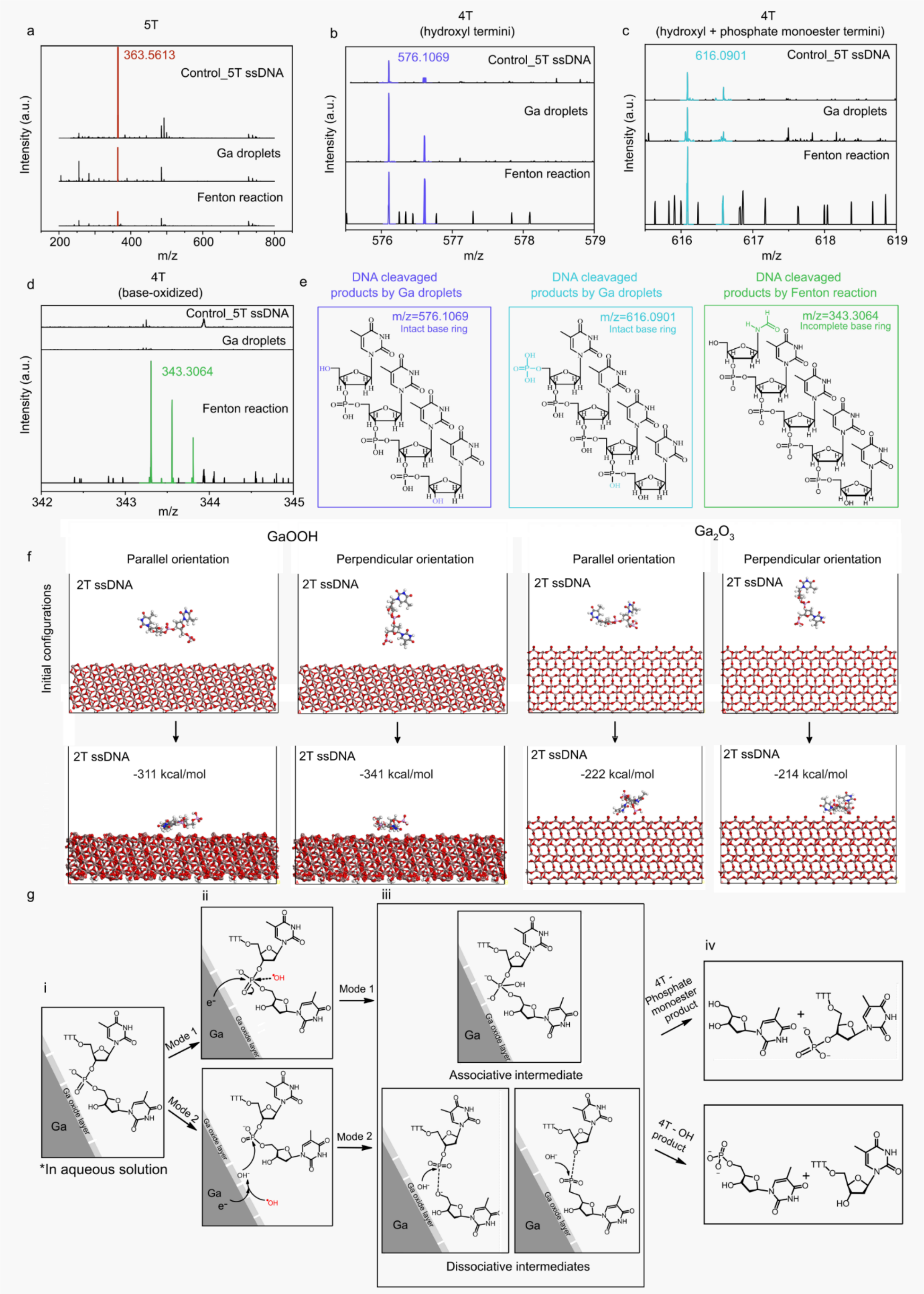

Fig.4 | Mechanism of Ga droplets' nuclease-mimicking effect: ROS-assisted hydrolytic cleavage activity. a-d. MS analysis of 5T ssDNA fragments after Ga droplets or Fenton reaction treatment: (a) full spectrum showing fragmentation pattern with 5T ssDNA peaks; (b) zoom-in of 4T fragments with two hydroxyl termini; (c) zoom-in of 4T fragment with hydroxyl and phosphate monoester termini; (d)

zoom-in of 4T fragment with oxidized base. In control samples, some hydrolytic-like peaks were observed due to in-source decay and high-voltage ionization[8,9]. The analysis of the 3T-to-1T fragments is provided in Supplementary Discussion 4. e. 4T fragment structures corresponding to m/z in b-d. f. MD simulations of 2T-ssDNA interacting with the Ga oxide layer, Ga_2O_3 and GaOOH, using two initial configurations: ssDNA perpendicular or parallel to the surface. The water molecules were made invisible for clarity of visualization **g.** Proposed mechanism of Ga-induced DNA cleavage.

Supplementary Method:

Supplementary Method 6. Molecular dynamics (MD) simulations details

Classical MD simulations were performed using the Large-scale Atomic/Molecular Massively Parallel Simulator (LAMMPS) to investigate the interactions between the single-stranded DNA (ssDNA) and the oxide layer surfaces.[12] Force field parameters for the Ga atoms were initially adapted from the Universal Force Field (UFF) parameters for aluminium (Al), given their similar chemical properties as group 13 elements.[13] These parameters were subsequently refined through iterative optimization until the simulated system density was within 10% of the experimental values.

To model the Ga_2O_3 substrate, the (100) surface was cleaved from the most stable β-Ga_2O_3 bulk structure.[14] The optimised surface dimensions were 54.75 × 58.06 × 24.77 $Å^{3,}$ and the model contained 2880 Ga and 4320 O atoms. The GaOOH substrate was constructed using the bulk crystal structure obtained from the Materials Project database and cleaving the (110) surface with dimensions of 45.71 × 62.22 × 14.91 $Å^3$ and containing 1200 Ga, 1200 H, and 2400 O atoms. This surface was chosen as it corresponds to the dominant diffraction peak, as consistently reported in prior studies[1,15]. The optimized force field parameters for both oxides are presented in Supplementary Table 13. After iterative optimization, the systems reached equilibrium densities of 6.05 g/cm^3 for Ga_2O_3 and 4.68 g/cm^3 for GaOOH after 5 ns *NPT* simulations (1 fs timestep), in good agreement with experimental values of 5.89 g/cm^3 and 5.20 g/cm^3, respectively.

The ssDNA-water layer was constructed by randomly distributing water molecules to achieve a density of approximately 1.0 g cm^{-3} to account for solvent effects. This ssDNA-water layer was then added to the simulation boxes of the Ga_2O_3 and GaOOH substrates. Force field parameters for the ssDNA and the water molecules were taken from the AMBER force field.[16] Molecular dynamics simulations were performed for 5 ns using the *NVT* ensemble at 305 K for 8 systems: Ga_2O_3-ssDNA-two thymine (2T), Ga_2O_3-ssDNA-two guanine (2G), GaOOH-ssDNA-2T, and GaOOH-ssDNA-2G, with each system simulated with the ssDNA initially oriented parallel and perpendicular to the surfaces. Non-bonded bonded intramolecular interactions were handled using AMBER scaling: 1-2 and 1-3 interactions were excluded, while 1-4 electrostatic and van der Waals interactions were scaled by factors of 1/1.2 and 1/2, respectively.[17] The temperature was maintained using the Nosé-Hoover thermostat[18,19]. Non-bonded interactions were treated with cutoff distances of 8.5 Å for van der Waals forces and 15 Å for electrostatic interactions. Geometric mixing rules were used for the pairwise van der Waals interactions. All simulations employed a 1 fs timestep, with atomic coordinates recorded every 10 ps for the trajectory analysis. Binding energies (E_{bin}) were calculated using the following equation:

$$E_{\mathrm{bin}} = E_{\mathrm{total}} - E_{\mathrm{substrate}} - E_{\mathrm{solvated\_DNA}}$$

where E_{total} is the total energy of the combined system after 5 ns *NVT* equilibration, $E_{\mathrm{substrate}}$ is the equilibrated energy of the isolated substrate (Ga_2O_3 or GaOOH), and $E_{\mathrm{solvated\_DNA}}$ is the energy of the solvated ssDNA layer. All binding energies were calculated in the presence of explicit water molecules to account for solvation effects.

Supplementary Method 7. Density functional theory (DFT) computational methods

......Calculations for the Ga ion (Ga^{3+}) -ssDNA system followed the same reaction sequence and energy-referencing scheme, providing qualitative insight into the relative energetics of Ga^{0}- and Ga^{3+}-involved DNA cleavage pathways.

Supplementary Discussion:

Supplementary Discussion 5. MD simulation for ssDNA and Ga oxide interaction

To investigate the binding interactions between the ssDNA and Ga_2O_3/GaOOH substrates in different initial orientations, we performed MD simulations with explicit solvation (Fig. 4f and Supplementary Fig.29 d). After equilibration (5 ns *NVT* at 305 K), we analysed the interfacial interactions from the final frame of each trajectory and calculated the corresponding binding energies (Fig. 4f and Supplementary Fig.29d). The water molecules were made invisible for clarity of visualization.

For ssDNA-2T on Ga_2O_3 (Fig.4f), both initial orientations (parallel and perpendicular) converged to parallel configurations, with the phosphate backbone preferentially aligned towards surface O atoms. The binding energies were -222 kcal mol^{-1} and -214 kcal mol^{-1} for the two structures, respectively. In contrast, ssDNA-2G exhibited a stronger affinity to Ga_2O_3, with the initially parallel orientation (Supplementary Fig.29 d) maintaining a V-shaped conformation by changing its torsion angles (binding energy: -290 kcal mol^{-1}), while the initially perpendicular orientation adopted a tilted configuration on the surface (binding energy: -139 kcal mol^{-1}). The binding energy of this structure was weaker due to fewer functional groups interacting with the surface. The enhanced binding of ssDNA-2G compared to ssDNA-2T can be attributed to the greater number of aromatic rings in the guanine bases, which provide stronger van der Waals interactions with the Ga_2O_3 substrate.

For ssDNA interacting with the GaOOH substrate, the binding was notably stronger than with Ga_2O_3. For ssDNA-2T (Fig.4f), both initial orientations converged to parallel configurations with binding energies of −311 kcal mol^{-1} and -341 kcal mol^{-1}. In comparison, ssDNA-2G displayed a stronger affinity for the GaOOH surface than ssDNA-2T, adopting a parallel orientation regardless of its initial configuration (Supplementary Fig.29d). Both structures had binding energies of -462 kcal mol^{-1} (initially parallel) and -369 kcal mol^{-1} (initially perpendicular). The consistently stronger binding energies for a parallel to the surface indicate greater thermodynamic stability as a result of increased contact area and enhanced interactions due to the type of functional group.

Overall, the MD simulations demonstrate that: (i) ssDNA binds more strongly to GaOOH than Ga_2O_3, and (ii) ssDNA-2G exhibits enhanced substrate affinity compared to ssDNA-2T due to the additional aromatic rings that enhance van der Waals interactions.

Supplementary Discussion 7. Possible mechanism of Ga droplets' preference to T-T and A-A

The adsorption behavior of ssDNA on Ga droplets was further examined using competitive inhibition by thymidine and phosphate ions. Previous studies have established that inhibition by free nucleosides (e.g., thymidine) indicates adsorption through sugar or nucleobase moieties[20], whereas inhibition by phosphate ions implies interaction with the phosphodiester backbone[21]. The results show that Ga droplets' DNA cleavage activity was selectively suppressed by phosphate ions but not by thymidine (Supplementary Fig.29 a, b). Combined with MD simulation results showing that ssDNA adopts a largely flat adsorption configuration on Ga oxide layer and DFT results indicating preferential binding of the phosphodiester group to Ga^{0}, these findings indicate that the ssDNA molecule adsorbs as a whole onto the Ga surface, while interactions involving the phosphodiester backbone play the dominant role in the subsequent cleavage.

To clarify the relationship between adsorption and the observed cleavage preference toward 5T vs 5G sequences, quantitative adsorption experiments were performed (Supplementary Method 8,9). The

results show that GaOOH exhibits stronger ssDNA adsorption than Ga_2O_3, and that both oxide surfaces display higher affinity for 5G ssDNA compared to 5T ssDNA (Supplementary Fig.29c). Consistently, classical molecular dynamics (MD) simulations reveal that (i) ssDNA binds more strongly to GaOOH than to Ga_2O_3, and (ii) 2G ssDNA exhibits higher binding affinity than 2T ssDNA, attributable to additional aromatic rings that enhance van der Waals interactions (Fig.4f; Supplementary Fig.29d,e). In the DFT calculations, 5T and 5G exhibit comparable adsorption strengths to the Ga^0 atom, with a slight preference for 5T (Supplementary Fig. 27 b,c).

To exclude the possibility that reduced cleavage of 5G arises from overly strong adsorption leading to slow desorption and blockage of reactive sites on Ga droplets, we conducted cleavage experiments using excess Ga droplets to provide abundant reactive interfaces, followed by PBS addition to promote ssDNA desorption. Under these conditions, Ga droplets still exhibited a clear cleavage preference for T-rich sequences over G-rich sequences (Supplementary Fig.29f). Moreover, when normalized to the activity of the commercial nuclease S1, Ga droplets retained a stronger relative cleavage efficiency toward 5T ssDNA over 5G ssDNA (Supplementary Fig.29g). These results indicate that the observed cleavage preference originates from adsorption and binding configuration difference, rather than from desorption-limited kinetics.

Based on these results, we propose the following mechanism: Both experimental results and MD simulations show that, on Ga oxide surfaces (Ga_2O_3 and GaOOH), 2G ssDNA exhibits stronger adsorption than 2T ssDNA, whereas Ga droplets display lower cleavage efficiency toward 2G.

This suggests that DNA adsorption strength does not correlate linearly with cleavage efficiency. One possible reason is that strong adsorption of 2G induces pronounced conformational distortions, as evidenced by torsional angle changes and distorted structures (Supplementary Fig. 29d). Such structural deformations are likely to impede cleavage by altering the geometry of the phosphodiester bond, introducing steric barriers to ·OH access, and restricting the conformational flexibility required for efficient electron transfer. In contrast, 2T ssDNA maintains a more native and less distorted conformation, preserving the structural accessibility of the phosphodiester linkage (Fig. 4f) and thereby enabling more efficient cleavage. Another possible reason is that differences in interfacial electron redistribution. Following initial adsorption, Ga^0-2T and Ga^0-2G complexes exhibit different electronic redistribution patterns (Supplementary Fig.27d). In both cases, electrons are transferred from Ga to non-bridging phosphate oxygens; however, Ga^0-2T displays a more moderate and delocalized redistribution, resulting in a less stabilized phosphate environment and increased susceptibility to cleavage.

Supplementary Table:

Supplementary Table 13. MD calculation results

Supplementary Table 13-1. Lenard-Jones parameters for Ga_2O_3

Element	Epsilon (kca mol^{-1})	Sigma (Å)	Charges (e)
Ga	0.505000	4.008153	1.20
O	0.170000	3.000012	-0.80

Supplementary Table 13-2. Bond parameters for Ga_2O_3

Bond	K (kcal mol^{-1} $Å^{-2}$)	r_0 (Å)
Ga-O	800.0000	1.9485

Supplementary Table 13-3. Angle parameters for Ga_2O_3

Angle	K (kcal mol^{-1} rad^{-2})	q_0 (degrees)

O-Ga-O	119.50287	106.960
Ga-O-Ga	119.50287	121.946

Supplementary Table 13-4. Lenard-Jones parameters for GaOOH

Element	Epsilon (kca mol^{-1})	Sigma (Å)	Charges (e)
Ga	0.505000	4.008153	0.450000
H	0.000000	0.000000	0.090000
OH	0.210400	3.066000	-0.240000
Oz	0.170000	3.000012	-0.300000

Supplementary Table 13-5. Bond parameters for GaOOH

Bond	K (kcal mol^{-1} $Å^{-2}$)	r_0 (Å)
Ga-OH	597.500	2.05140
Ga-Oz	597.500	1.94647
H-OH	742.800	0.97000

Supplementary Table 13-6. Angle parameters for GaOOH

Angle	K (kcal mol^{-1} rad^{-2})	q_0 (degrees)
OH-Ga-OH	119.50287	76.926
Ga-OH-H	119.50287	111.988
Ga-OH-Ga	119.50287	103.074

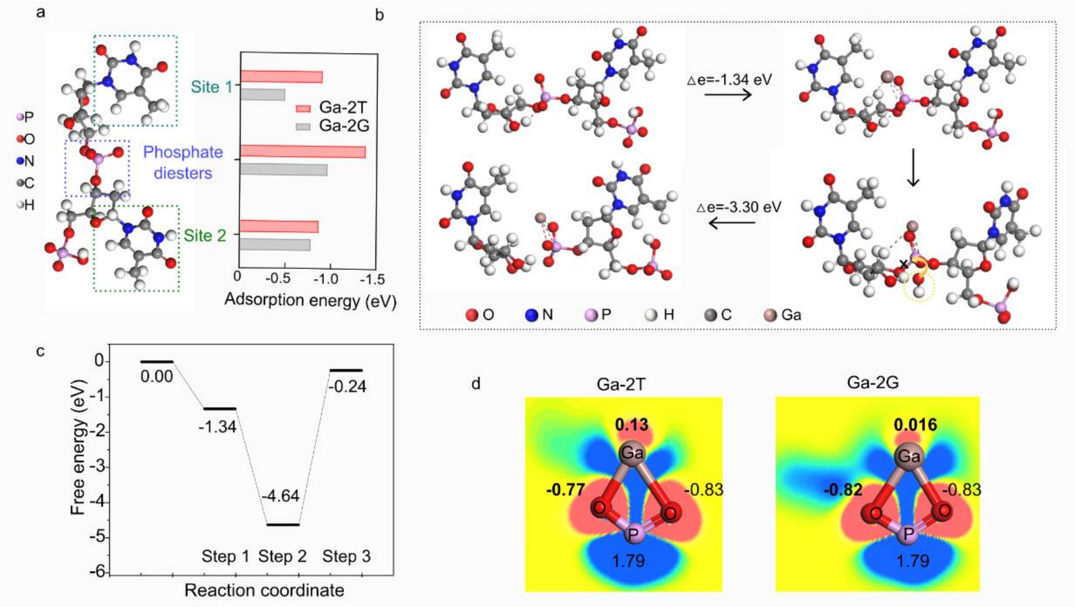

Supplementary Fig. 5. **(a)** Comparison of adsorption energies between Ga and 2T or 2G ssDNA at three representative adsorption sites: phosphate diester bond, base site 1 (near 3'-OH), base site 2 (near 5'-phosphate). **(b)** DFT-calculated reaction pathway, including Ga adsorption, ·OH cleavage, and Ga

desorption from 2T ssDNA. (**c**) Calculated reaction energy diagram of 2T ssDNA showing the energy profile for Ga^0-induced cleavage of the phosphate diester bond. (**d**) Charge density difference plot showing the Ga coordinated with the oxygen atoms around the phosphate diester bond of 2T *vs* 2G**.**

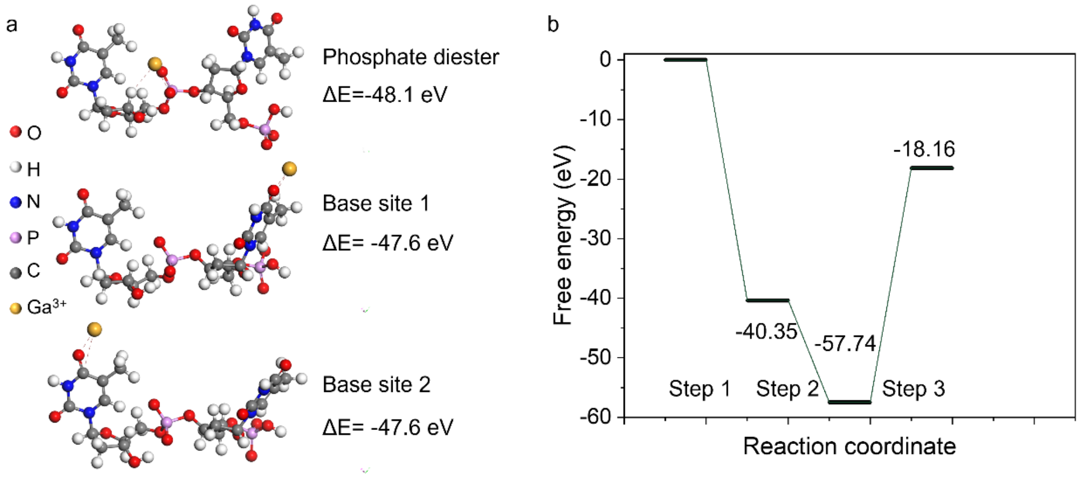

Supplementary Fig. 6. (**a**) Calculated adsorption energies of Ga^{3+} on 2T and 2G ssDNA at three representative binding sites: the phosphate diester group, base site 1 (proximal to the 3′-OH), and base site 2 (proximal to the 5′-phosphate). Ga^{3+} exhibits strong electrostatic coordination to the phosphate moiety relative to nucleobase sites for both sequences. (**b**) Computed reaction energy profile for the Ga^{3+}-2T ssDNA complex in the presence of •OH (Supplementary Method 7). The Ga^{3+}-phosphate interaction excessively stabilizes the post-reaction intermediate, resulting in a prohibitively high desorption barrier for product release ($\Delta E \approx 39.58$ eV for step 3). This large energetic penalty renders Ga^{3+}-assisted phosphodiester cleavage kinetically inaccessible, indicating that Ga^{3+} does not function as an effective center for DNA backbone scission.

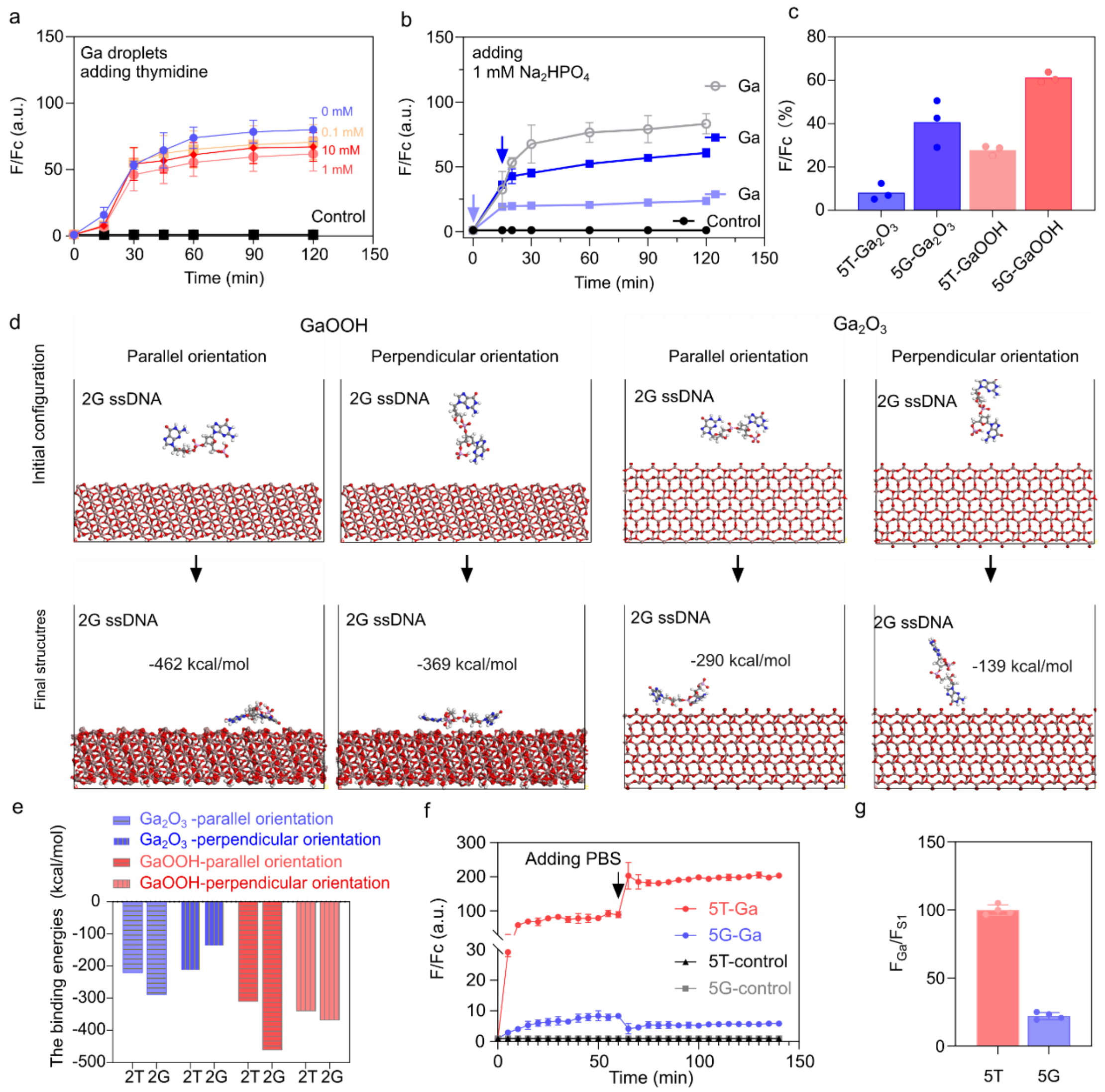

Supplementary Fig. 29. The ssDNA adsorption on Ga droplets and base-sequence preference. a-b, DNA cleavage activity of Ga droplets in the presence of added **(a)** thymidine and **(b)** Na_2HPO_4. **(c)**, Adsorption percentage of 5T ssDNA TR-reporter on Ga oxide surfaces (Ga_2O_3 and GaOOH) (Supplementary 8). **(d)** Classical molecular dynamics (MD) simulations of 2G ssDNA interacting with Ga oxide layers (Ga_2O_3 and GaOOH) using two initial configurations: ssDNA oriented perpendicular or parallel to the surface. **(e)** Comparison of binding energies in MD simulations of 2T ssDNA and 2G ssDNA interacting with Ga_2O_3 and GaOOH surfaces. **(f)** DNA cleavage activity of excess Ga droplets (0.5 mg mL^{-1}, fivefold higher than that used in other experiments) toward 5T and 5G ssDNA TR-reporters. **(g)** End-point fluorescence intensity (at 120 min) of 5T and 5G ssDNA TR-reporters cleaved by Ga droplets, normalized to the activity of S1 nuclease.

2-8. The mechanistic discussion about GaOOH is based on a Langmuir and a Chemical Science paper about eutectic Ga indium (EGaIn) liquid metal nanoparticles. It is not so clear that this can legitimately be compared with the present system.

Response: Thank you for raising this important point. We agree that pure Ga and eutectic Ga–In (EGaIn) are distinct systems and should not be treated as identical. However, the formation of GaOOH is a fundamental chemical property of Ga in aqueous environments, which is largely unaffected by alloying with In.

Thermodynamically, oxidation of Ga is significantly more favourable over In, as evidenced by the more negative Gibbs free energy of formation for Ga oxides[22]. As a result, Ga-rich oxide and hydroxide phases preferentially form at the surface. Consistent with this behavior, oxidation of EGaIn in water has been shown to induce phase separation into GaOOH and metallic In, with In excluded from the Ga-rich oxide/hydroxide phase[1]. This observation demonstrates that GaOOH formation is governed by Ga chemistry rather than by the specific alloy composition.

To ensure our mechanistic discussion is grounded in the present system, we have prioritized data from our new control experiments performed directly on pure Ga droplets rather than over-relying on alloy-based literature.

The discussion and results are provided in Supplementary Information and are shown below for convenience:

Supplementary Discussion 3:

"Upon the transfer from ethanol to DI water, the formation of ·OH is accompanied by the oxidation of Ga^0 into Ga. After 24 h in DI water (Supplementary Fig. 19), SEM images reveal a gradual morphological transformation of Ga droplets into rod-like oxide structures. Correspondingly, the Ga 3d XPS spectrum exhibits a dominant peak at ~20 eV, characteristic of Ga^{3+} species. Together, these results indicate the formation of GaOOH [1-3]……"

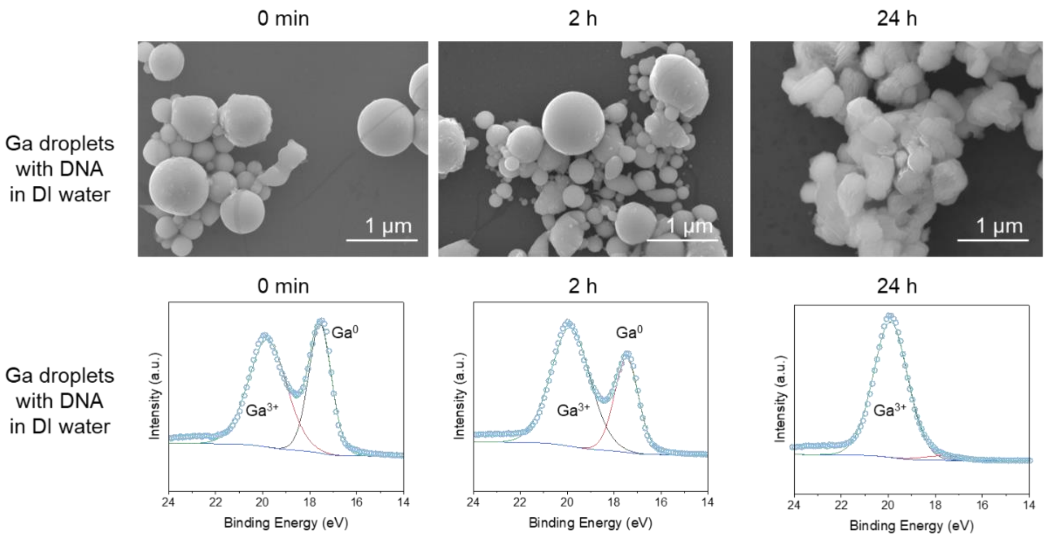

Supplementary Fig. 7. SEM images and Ga 3d XPS spectra of Ga droplets incubated with the TR-ssDNA reporter for 0 min, 2 h, and 24 h; SEM images show a gradual transformation of spherical droplets into rod-like structures; XPS results show a progressive oxidation of metallic Ga^0, with Ga^0 no longer detectable after 24 h. Based on this characteristic morphology and prior reports, the oxidized phase is assigned to GaOOH[1-3].

2-9. Also, high pH values were tested. Shouldn't Ga $(OH)_3$ form at high pH values? Also, extreme pH conditions lead to DNA degradation anyways (dsDNA is typically stable only between pH 5-9).

Response: Thank you for these important comments. Regarding GaOOH formation, Ga $(OH)_3$ is thermodynamically unstable in aqueous environments and readily undergoes dehydration to form hydrated GaOOH[2,3,23]. To clarify this process, we have revised the reaction scheme and cited the relevant literature documenting this transition in the Supplementary Discussion 3:

Upon exposure to aqueous solution, Ga_2O_3 gradually transforms into Ga $(OH)_3$ then GaOOH *via* Reaction 1[2,3,23].

$$Ga_2O_3 + 3H_2O \rightarrow 2\text{Ga(OH)}_3$$

$$\text{Ga(OH)}_3 \rightarrow \text{GaOOH} + H_2\text{O}$$

With respect to DNA stability, we would like to clarify that all pUC19 dsDNA cleavage experiments were performed exclusively at neutral pH (pH 7.0), where the double-stranded structure is fully stable. Extreme pH conditions were only utilized during studies of the single-stranded (ssDNA) TR-reporter to explore the limits of the Ga-droplet reactivity.

To address the reviewer's concern regarding non-specific pH-induced DNA degradation or dye effects, we performed quantitative control experiments (now added as Supplementary Fig. 36). In these controls, the TR-ssDNA reporter was exposed to HCl or NaOH without Ga droplets. The resulting fluorescence increased only modestly (from ~1500 a.u. to <3000 a.u., a<2-fold change). In stark contrast, the Ga-droplet mediated reaction pronounced a signal exceeding 120,000 a.u. (>80-fold increase; Fig. 6h, i). This significant disparity (nearly two orders of magnitude) confirms indicate that the observed signal is a direct consequence of Ga droplet–mediated cleavage rather than pH-induced artifacts.

The relevant data mentioned above are provided below for your reference:

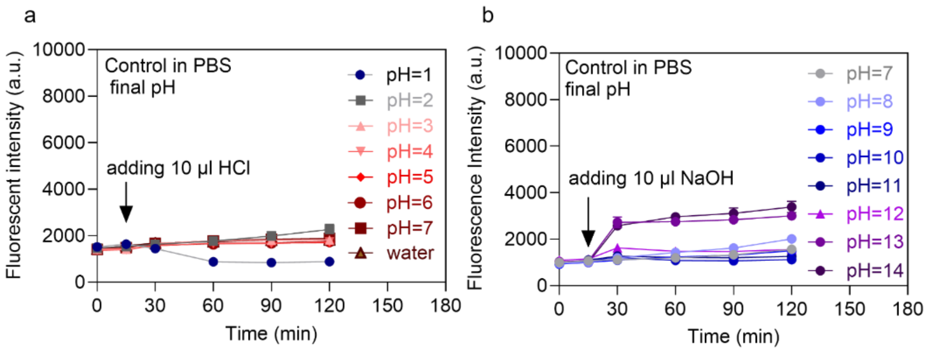

Supplementary Fig. 8. Control experiments showing fluorescence changes of the TR-reporter in PBS upon HCl or NaOH addition. In the control sample containing only TR-ssDNA reporter with HCl or NaOH, the fluorescence increased modestly from ~1500 a.u. to < 3000 a.u. (< 2-fold). In contrast, samples with Ga droplets exhibited a pronounced fluorescence increase exceeding 120,000 a.u. (>80-fold in Fig.6). These results indicate that Ga droplets act as efficient artificial nuclease-mimic, enabling DNA cleavage even under extreme pH conditions.

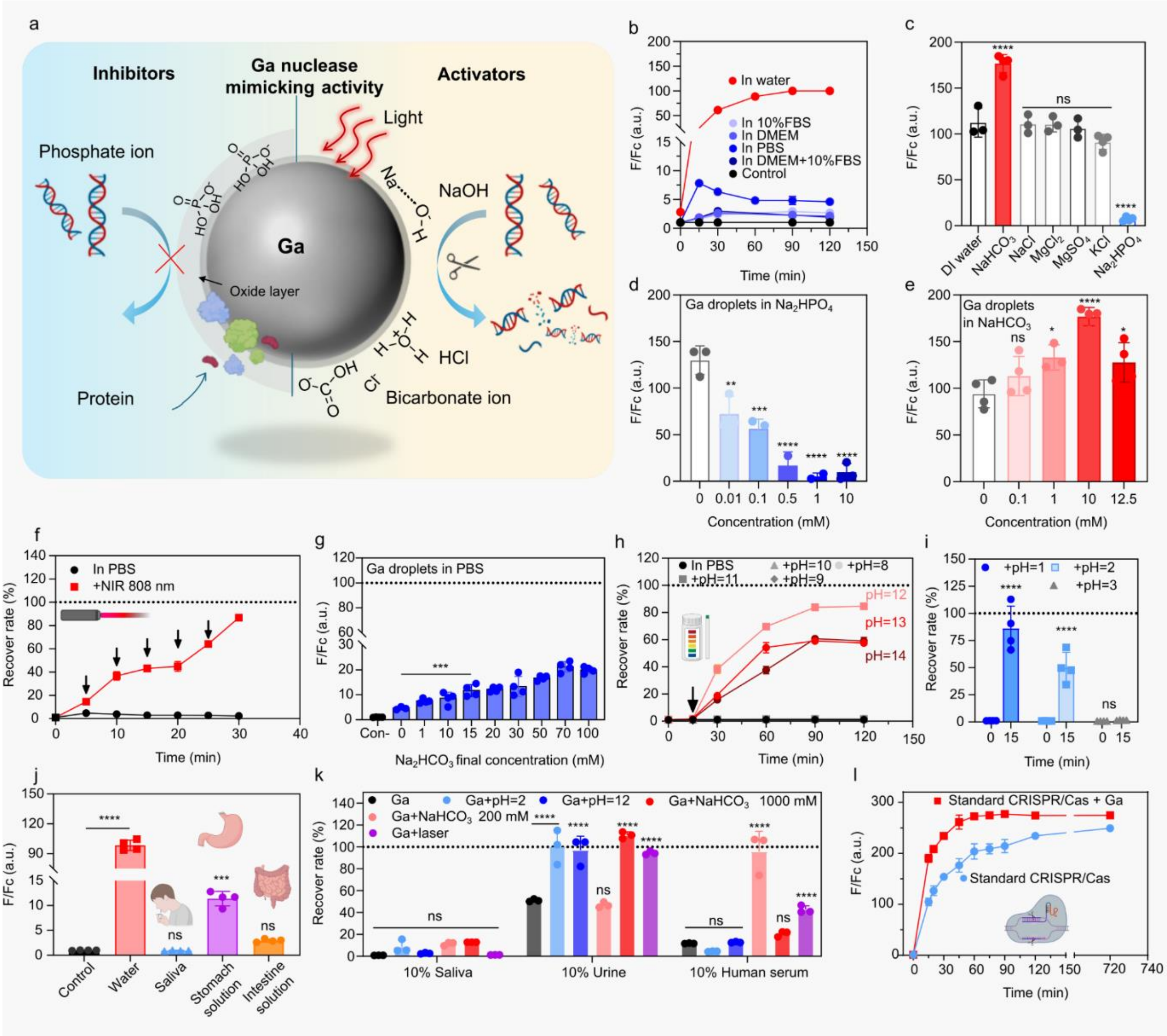

Fig. 6 | Modulation of Ga droplets' nuclease-mimicking activity in typical biological environments. **a.** Schematic illustrating the inhibition and recovery of Ga droplets mediated DNA cleavage under biologically relevant conditions. **b.** Cleavage of TR-ssDNA reporters by Ga droplets in various biological media, including DI water, DMEM, DMEM with 10% FBS, pure FBS, and PBS. **c.** Effects of intracellularly abundant ions on Ga nuclease-mimicking activity at physiological concentrations. **d.** Inhibition of Ga-mediated DNA cleavage by Na_2HPO_4 solution. **e.** Concentration-dependent enhancement of Ga nuclease-mimicking activity by $NaHCO_3$. **f-i.** Recovery of Ga nuclease-mimicking activity in PBS by **(f)** 808 nm NIR laser irradiation, **(g)** adding $NaHCO_3$, **(h)** adding NaOH solution (final pH=11), **(i)** adding HCl solution (final pH=3) **(g)**. **j-i.** TR-ssDNA reporters cleaved by Ga droplets in different biologically relevant environments: **(j)** in 100% saliva, simulated gastric and intestinal fluids, (**k**) in 10% saliva, urine, human serum, with different additives, (**l**) in the CRISPR (clustered regularly interspaced short palindromic repeats)/Cas9 (CRISPR-associated protein 9). ($p < 0.05$ is considered as statistically significant. *$p < 0.05$, **$p < 0.01$, ***$p < 0.001$, and ****$p < 0.0001$. a.u = arbitrary units). Some graphical elements were created*Created in BioRender. Liu, L. (2026) https://BioRender.com/407xx9q.*

2-10. The formation of alloy particles is not described in detail.

Response: Thank you for this comment. To address this point, we have expanded the description of the alloy particle formation process in Supplementary Method 9.

“The preparation of Ga-based alloys followed previously established protocols.[24-26] Owing to the unique ability of liquid Ga to dissolve a wide range of metals at relatively mild temperatures (< 400 °C), this approach provides a straightforward route for alloy formation[26].”

In addition, SEM and EDS analyses of the resulting alloy particles have been performed and included in the Supporting Information to confirm alloy composition and morphology.

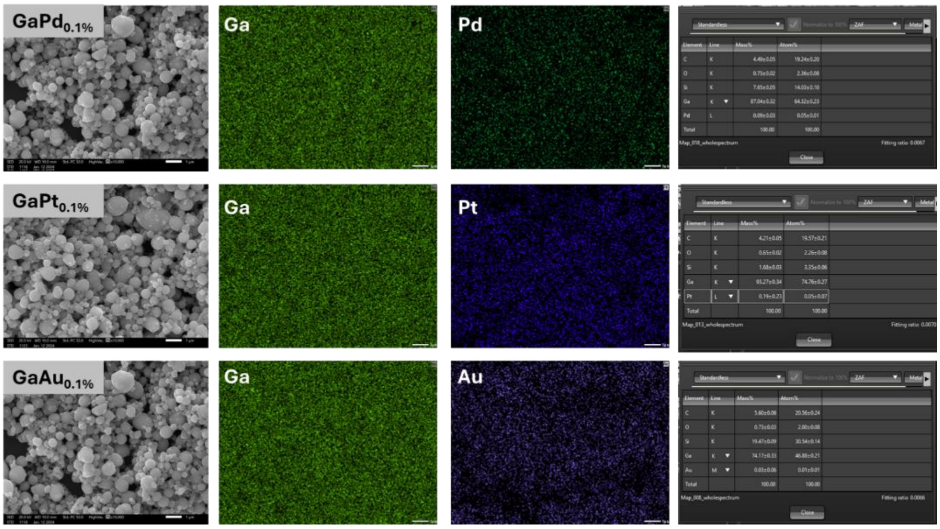

Supplementary Fig. 31. SEM images, EDS elemental maps, and elemental composition analysis of Ga-based alloys.

2-11. “After different periods of time, Na2HPO4 was added to a final concentration of 20 mM to stop the reaction and detach the cleaved plasmid from the droplets’ surface, which improved visualization of bands in the gel.” What is the rationale for this step? And could the increased pH (around 9) instead explain the observed effect?

Response: Thank you for the insightful question. We examined whether an increase in pH alone could promote DNA detachment by performing control experiments using NaOH at pH 9. This treatment resulted in only a minor detachment effect and was significantly less effective than Na_2HPO_4 (Response Figure 1). In addition, the addition of PBS (containing phosphate ions, pH=7) can also promote the attachment of ssDNA on Ga droplets’ surface (Supplementary Discussion 7). The results indicate that while elevated pH may contribute modestly to DNA detachment, the dominant factor is the presence of phosphate ions.

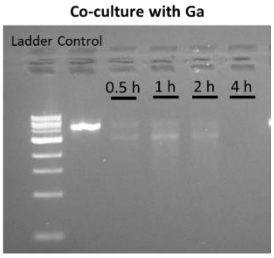

Response Figure 1. The gel electrophoresis of adding pH=9 NaOH into Ga-pUC19 solution.

The following has been revised in Agarose gel electrophoresis (Method 4, Main manuscript page 18) to address your question:

After different periods of time, Na_2HPO_4 was added to a final concentration of 20 mM to stop the reaction and detach the cleaved plasmid from the droplets' surface, which improved visualization of bands in the gel. This behavior is attributed to the strong adsorption affinity of phosphate ions toward Ga oxide/hydroxide surfaces, which may competitively displace DNA adsorption. The details are provided in Supplementary Discussion 7.

In Supplementary Discussion 7, the related discussions are shown below:

The adsorption behavior of ssDNA on Ga droplets was further examined using competitive inhibition by thymidine and phosphate ions. Previous studies have established that inhibition by free nucleosides (e.g., thymidine) indicates adsorption through sugar or nucleobase moieties[20], whereas inhibition by phosphate ions implies interaction with the phosphodiester backbone[21]. The results show that Ga droplets' DNA cleavage activity was selectively suppressed by phosphate ions but not by thymidine (Supplementary Fig.29 a, b). Combined with MD simulation results showing that ssDNA adopts a largely flat adsorption configuration on Ga oxide layer and DFT results indicating preferential binding of the phosphodiester group to Ga^0, these findings indicate that the ssDNA molecule adsorbs as a whole onto the Ga surface, while interactions involving the phosphodiester backbone play the dominant role in the subsequent cleavage.

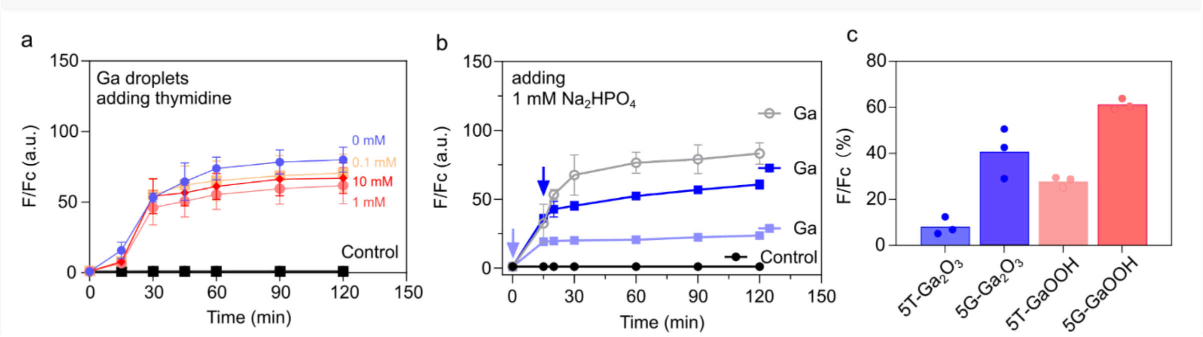

Supplementary Fig. 29. The ssDNA adsorption on Ga droplets and base-sequence preference. a-b, DNA cleavage activity of Ga droplets in the presence of added **(a)** thymidine and **(b)** Na_2HPO_4.

2-12. EPR: "Ga droplets (40 µL, 1 mg/mL in EtOH) underwent solvent exchange into water, followed by 5 s of sonication for dispersion. Then, 4 µL of 1 M DMPO (for ·OH) or TEMPO (for O_2^-) in aqueous solution was added and sonicated" Sonication itself may induce radical formation. Was this aspect considered and double-checked with the literature? Also, it is not clear what "solvent exchange" specifically means here.

Response: We thank the reviewer for raising this important point. We agree that sonication can, in some systems, induce radical formation and that this possibility must be carefully excluded to ensure the validity of our EPR data.

To address this concern, we performed additional control EPR experiments, including measurements of (i) Ga droplets with sonication, (ii) Ga droplets without sonication, and (iii) water with and without sonication under otherwise identical conditions. These controls show that ·OH signals are observed only in the presence of Ga droplets and are not generated by the sonication process itself. Together, these additional controls and clarifications confirm that the observed ·OH generation originates from Ga droplets rather than from sonication-induced artifacts.

More details to back up this answer can be found in the revise Main manuscript and Supplementary Information and are provided below for your convenience:

Main manuscript (Page 6):

Mechanism of the Nuclease-Mimicking Activity of Ga droplets

Identification of Ga^0 as the Active Species

The system was primarily composed of metallic Ga (Ga^0) and DNA. Although Ga^{3+} ions (Fig. 3a; Method 6) and Ga oxides can coexist under these conditions[2], rigorous control experiments showed that neither species contributed significantly to the observed activity. Specifically, chelating Ga^{3+} with ethylenediaminetetraacetic acid (EDTA) did not significantly alter DNA cleavage efficiency (Fig. 3b), and Ga^{3+} alone showed no cleavage activity (Supplementary Fig. 9a). Similarly, Ga oxides were also inactive when tested independently for DNA cleavage (Supplementary Fig. 9b, c, 10; Supplementary Method 3). These results confirm that Ga^0 is the central species driving DNA cleavage, prompting further investigation into the interfacial reactions between Ga^0 and the aqueous environment.

EPR Characterization of Radical Intermediates

To elucidate how Ga^0 mediates DNA cleavage, we investigated whether Ga^0 -associated reactive oxygen species (ROS) generated in aqueous solution[27,28] contribute to this process. Electron paramagnetic resonance (EPR) spectroscopy and signal simulations (Method 7 and Supplementary Method 4) were performed to detect these intermediates. To clarify the role of sonication, we compared the cleavage intensity and EPR signals of Ga droplets transferred to deionized (DI) water with and without sonication (Fig. 3c-e). The results show that Ga droplets can cleave DNA and generate ROS without sonication. However, brief sonication enhanced this activity by improving droplet dispersion, disrupting the surface oxide layer, and increasing interfacial exposure, as evidenced by the intensified EPR signals and increased cleavage efficiency (Supplementary Fig. 11; Fig.3c).

The EPR spectra (Fig. 3d, e) revealed a mixture of 5,5-Dimethyl-1-pyrroline N-oxide (DMPO) adducts: DMPO-OH, DMPO-OOH, and DMPO-$CH(CH_3)OH$ adducts, indicating the generation of hydroxyl radical (·OH) [29], superoxide ($O_2\cdot^-$)[30,31], and 1-hydroxyethyl radicals ($CH(CH_3)OH\cdot$) [32], respectively. The $CHCH_3OH\cdot$ signal likely stems from trace EtOH [33] in the Ga stock solution. Due to the short half-life of DMPO-OOH ($t_{1/2}$ = 60 s)[34], this signal disappeared after a 90-min dead time, leaving a clear and prominent ·OH signal (Fig. 3f). No ·OH signal was detected in the Ga-free controls (Supplementary Fig. 12 a, b), and the 2,2,6,6-tetramethylpiperidine (TEMP) probing ruled out singlet oxygen (Supplementary Fig. 13). These results collectively demonstrate that Ga^0-water system intrinsically produces ·OH and $O_2\cdot^-$.

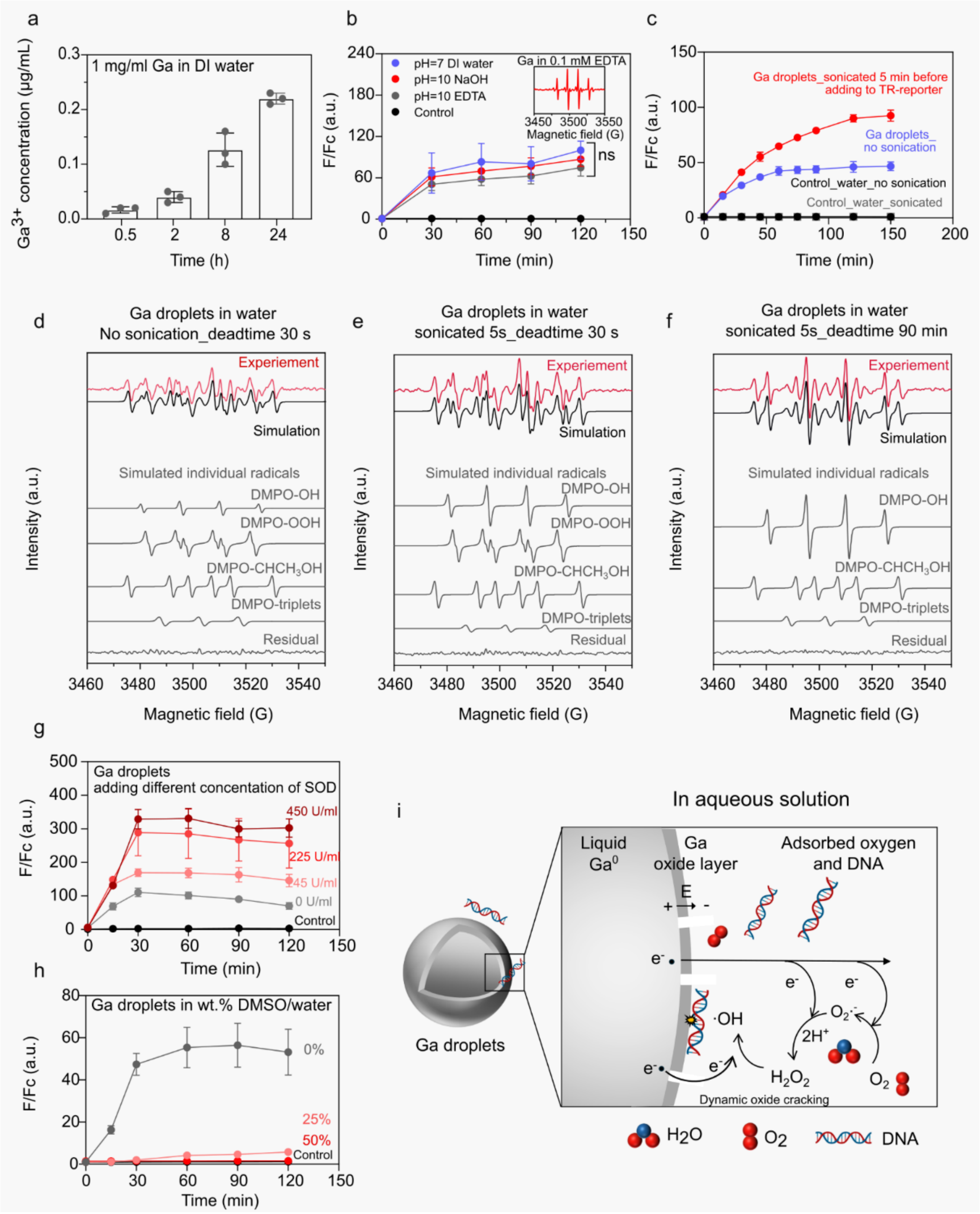

Figure 3. | ROS generation of Ga droplets in aqueous solution. a. Inductively coupled plasma–mass spectrometry (ICP-MS) analysis showing time-dependent release of Ga^{3+} ions from Ga droplets in deionized water. b. Fluorescence intensity of TR-ssDNA reporters after treatment with Ga droplets in the presence of EDTA under chelating-active conditions (pH = 10). Inset figure: EPR spectra of Ga droplets in 0.1 mM EDTA. (a.u = arbitrary units). c. Comparison of the nuclease-mimicking activity of Ga droplets with and without sonication before incubation with the ssDNA TR-reporter. d-f. EPR spectra of Ga droplets in water without sonication (d), and after brief sonication (5 s), measured following different delay times (30 s (e) and 90 min (f); dead time defined as the interval between sample preparation, sealing, equipment setting and EPR data acquisition). Corresponding simulated spectra are shown for comparison (see Supplementary Method 4).

Supplementary figures:

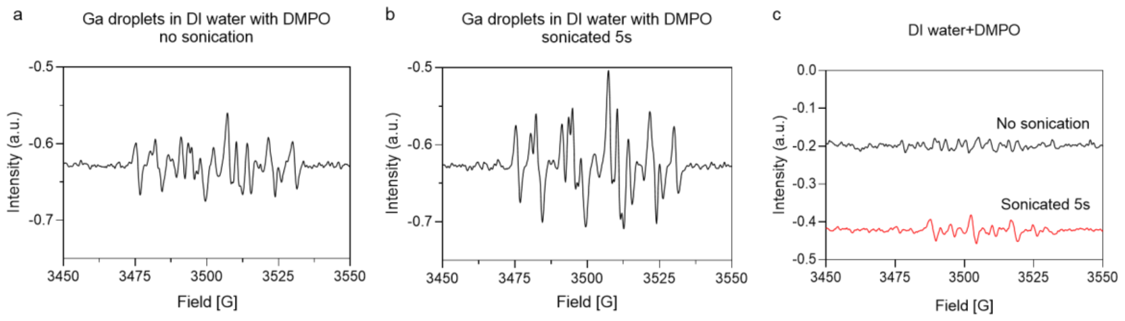

Supplementary Fig. 11. EPR spectra of Ga droplets in DI water in the presence of DMPO: **(a)** no sonication, **(b)** sonicated for 5 s. **(c)** Control spectra of DI water + DMPO without sonication and after 5 s sonication. Dead time = 30s. The triplet signal observed in DI water after 5 s sonication indicates that it originates from sonication-induced partial degradation of DMPO.

Supplementary Methods:

EPR spectroscopy (Method 7)

For the detection of ·OH and $O_2{\cdot}^-$, Ga droplets (40 µL, 1 mg mL^{-1} in EtOH) were subjected to solvent exchange into DI water. Briefly, EtOH was removed by centrifugation, followed by the addition of DI water and gentle pipetting to redisperse the Ga droplets prior to the experiment. DMPO was purified by treatment with activated charcoal, filtered, and stored as frozen aliquots prior to use. Then DMPO (4 µL, 100 mM) was then added. Samples were measured without sonication or after sonication for 5 s or 30 s. Negative control samples contained only DI water and DMPO, with and without identical sonication. The positive control was the Fenton reaction; experimental details are described in the section on Fenton reaction–mediated DNA cleavage. EPR spectra were recorded after defined dead times (0-90 min) following sample preparation, as specified in each figure, to capture the temporal evolution of $O_2{\cdot}^-$ to ·OH.

For the detection of singlet oxygen, Ga droplets (40 µL, 1 mg mL^{-1} in EtOH) were solvent-exchanged into DI water. TEMP was freshly distilled at 152°C and was then added. Samples were measured directly without sonication. Negative control samples containing only DI water with TEMP were prepared. Positive control samples consisted of TEMP mixed with 50 µM methylene blue and irradiated with red light (>630 nm) to induce photosensitized singlet oxygen ($^1O_2$) generation.

All samples were immediately transferred into glass capillaries after different treatment and sealed with wax. Spectra were acquired at room temperature using a Bruker EMX X-band spectrometer (microwave power 20 mW; scan width 200 G).

Supplementary Method 4. Electron paramagnetic resonance (EPR) spectral analysis and simulation

Continuous-wave X-band EPR spectra were analyzed using the SpinFit module in Xenon (Bruker BioSpin), which employed perturbation theory, with the electron Zeeman interaction treated as the dominant term and isotropic hyperfine coupling included as a first-order perturbation. Experimental spectra were fitted using multicomponent simulations to account for overlapping signals arising from different 5,5-dimethyl-1-pyrroline N-oxide (DMPO) spin-trapped radical adducts. Hyperfine coupling constants, including the g value of each radical adduct, were initially loaded from the SpinFit library. A single isotropic g value was assumed for each radical species. Least-squares fitting was used to optimize

hyperfine coupling constants, line positions, line widths, and relative component intensities simultaneously, enabling estimation of the relative contributions of individual radical species. The quality of the fits was assessed by inspection of the residual spectra, defined as the difference between the experimental and simulated spectra. Following spectral simulation, the hyperfine coupling constants of each DMPO radical adduct were compared with values reported in the NIEHS spin trap database: https://www.niehs.nih.gov/ research/resources/databases/spintrap.

The hyperfine coupling constants used in the simulations were as follows. 1. Ga in water without sonication: DMPO-hydroxyl radical (DMPO-OH) (aN = 15.05 G, aH = 14.45 G), DMPO-hydroperoxyl radical (DMPO-OOH) (a_N = 14.15 G, a_H^β = 11.27 G, a_H^γ = 1.21 G), DMPO-1-hydroxyethyl (DMPO-$CH(OH)CH_3$)· (a_N = 15.87 G, a_H = 22.97 G), and a triplet attributed to partially degraded DMPO: a_N = 14.91 G; 2. Ga in water sonicated 5s_deadtime 30s: DMPO-OH (a_N = 14.99 G, a_H = 14.55 G), DMPO-OOH (a_N = 14.11 G, a_H^β = 11.31 G, a_H^γ = 1.22 G), DMPO-$CH(OH)CH_3$ (a_N = 15.87 G, a_H = 22.96 G), and a triplet attributed to partially degraded DMPO: a_N = 15.02 G; 3. Ga in water sonicated 5s_deadtime 90 min: DMPO-OH (a_N = 14.94 G, a_H = 14.57 G), DMPO-$CH(OH)CH_3$ (a_N = 15.85 G, a_H = 22.88 G), and a triplet attributed to partially degraded DMPO: a_N = 14.66 G.

For the DMPO-OOH adduct, because DMPO spin trapping cannot distinguish between superoxide radical ($O_2{\cdot}^-$) and its protonated form, the hydroperoxyl radical (·OOH), radical species assignment is guided by the solution pH[35]. Under neutral conditions (DI water), superoxide ($O_2{\cdot}^-$) predominates ($O_2{\cdot}^- + H^+ \rightleftharpoons {\cdot}OOH$, $pK_a \approx 4.9$), and the DMPO-OOH signal is therefore attributed to $O_2{\cdot}^-$ [30,35].

We have also clarified the meaning of "solvent exchange" in the Method 7 (Main manuscript Page20):

"For the detection of ·OH and ·OOH, Ga droplets (40 µL, 1 mg mL^{-1} in ethanol) were subjected to solvent exchange into deionized (DI) water. Briefly, ethanol was removed by centrifugation, followed by the addition of DI water and gentle pipetting to redisperse the Ga droplets prior to the experiment. "

Minor issues:

2-13. For agarose gel electrophoresis, Ga droplets were "co-cultured" with DNA – "co-cultured" is inappropriate terminology. It is rather used when two cell lines are incubated at the same time at the same place. Better use "incubated together".

Response: We thank the reviewer for the suggestion; we have changed the "co-cultured" into "incubated together" throughout the paper.

2-14. Figure 2. It should be explained somewhere what "MinION" is.

Response: Thank you for the careful check. We have revised Fig. 2h to replace *MinION* with *PromethION 2 Solo* which was ultimately used for data acquisition and the captions.

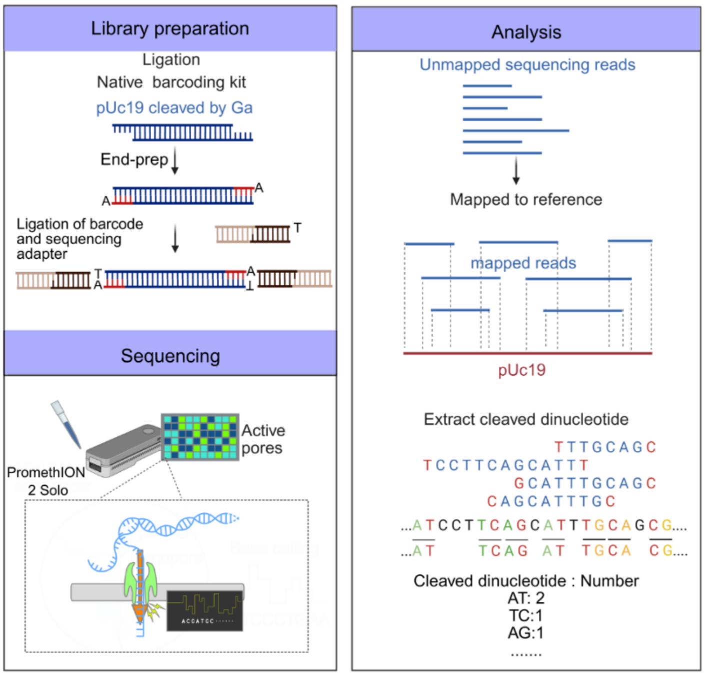

Fig. 2h. Schematic of Oxford Nanopore Technologies (ONT) sequencing used for single-molecule cleavage site identification, adapted from "Principle of Nanopore DNA Sequencing" by DataBase Center for Life Science (DBCLS), *CC-BY 4.0* and some elements are created in *BioRender. Liu, L. (2026) https://BioRender.com/38ph5fk. (Method 5)*

The following has been added to Method 5:

"Promethion flowcell (FLO-PRO114M) and sequenced on a PromethION 2 Solo device, a nanopore sequencer capable of generating long-read data in a single run, using Minknow version 24.06.15 with default settings."

The following text has been added to Supplementary Information 2.1 Library preparation:

"The prepared library was initially sequenced using a MinION device (Oxford Nanopore Technologies), a portable single–flow-cell nanopore sequencer. However, the sequencing yield was insufficient for reliable downstream analysis. To overcome this limitation, sequencing was performed on a PromethION 2 Solo system using an R10.4.1 flow cell and MinKNOW (v24.06.15), which provides substantially higher throughput and improved data quality."

2-15. Also, other ROS might play a role for DNA cleavage. Only hydroxyl radicals, superoxide radical anions and CO_2 radicals were evaluated.

Response: Thank you for this insightful comment. We agree that additional reactive oxygen species (ROS) may potentially contribute to DNA cleavage. To further clarify this point, we performed additional EPR measurements and examined the possible involvement of superoxide and singlet oxygen. The results still show that that ·OH radicals are essential for the Ga droplets-mediated DNA cleavage, while the contribution of $CH_3CH_2O\cdot$ is negligible and $O_2^{\cdot -}$ is indirect.

We note that a weak signal previously assigned to CO_2-derived radicals overlaps strongly with signals from ethanol-derived radicals, making unambiguous assignment challenging based on experimental spectra alone. With the aid of updated spectral simulation using the SpinFit module in Xepr (Bruker

BioSpin), this minor feature is now more reliably attributed to CH_3CH_2OH-related radical species rather than CO_2-derived radicals (please refer to the answer to question Q2-12 for more details).

More details to back up this answer can be found in the revise Main manuscript and Supplementary Information and are provided below for your convenience:

Main manuscript (page 6)

Validation of ·OH as the Primary Cleavage Agent

To identify which of these radicals involved in this cleavage process, we performed a series of scavenger experiments. The addition of EtOH to probe the involvement of $CHCH_3OH$· had no effect on cleavage (Supplementary Fig. 14), and the $O_2^{·-}$ scavenger superoxide dismutase (SOD) actually increased the signal (Fig. 3g) likely by accelerating the conversion of $O_2^{·-}$ to hydrogen peroxide (H_2O_2)[36] and subsequently into ·OH. Conversely, the ·OH scavenger dimethyl sulfoxide (DMSO), which reacted with ·OH substantially faster than with $O_2^{·-}$ [37,38] markedly suppressed the DNA cleavage (Fig. 3h). Moreover, the direct involvement of ·OH was further confirmed by the observed decrease of ·OH signal as the concentration of DNA increased (Supplementary Fig. 15a). Collectively, these results confirm that ·OH radicals are the primary species responsible for the Ga droplets-mediated DNA cleavage, while $CHCH_3OH$· is negligible and $O_2^{·-}$ is indirect. Finally, the suppression of cleavage under hypoxia (nitrogen-purged) or water-depleted (glycerol environment) conditions confirms that the presence of both oxygen and water is essential for the interfacial generation of the ·OH radicals, which is required for this nuclease-mimicking activity (Supplementary Fig. 15b-d).

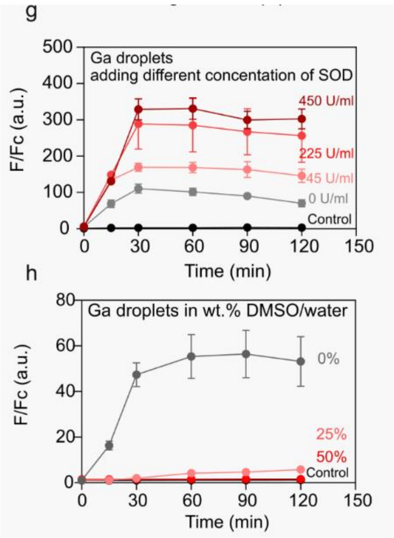

Figure 3.g. Effect of SOD, a superoxide scavenger, on the nuclease-mimicking activity of Ga droplets, evaluated using the TR-reporter. **h**. Effect of ·OH quenchers, DMSO, on the nuclease-mimicking activity of Ga droplets, using the TR-reporter.

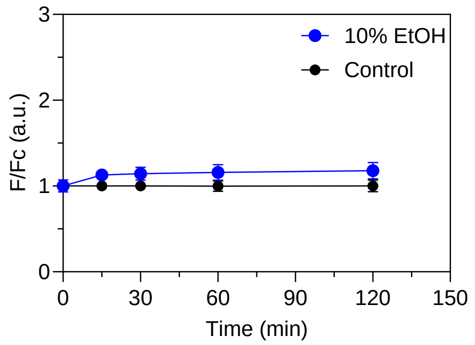

Supplementary Fig. 14. Incubation of TR-ssDNA reporter with 10% EtOH after 5s sonication showing no cleavage effect, indicating the contribution of $CH_3CH_2O\cdot$ is negligible.

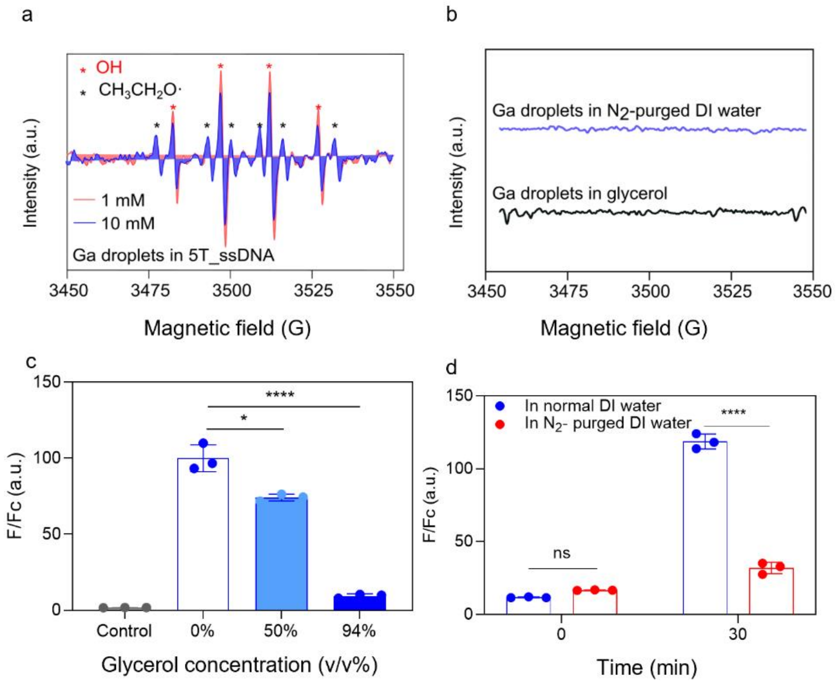

Supplementary Fig. 15. (a) EPR spectra of Ga droplets in 1 mM or 10 mM 5T_ssDNA; deadtime 90 min. (b) EPR spectra of Ga droplets in oxygen-depleted water (nitrogen-purge) and water-depleted (glycerol) condition, showing suppressed radical formation. TR-ssDNA reports cleavage activity of Ga droplets in (c) water-depleted (glycerol) condition and (d) oxygen-depleted water (nitrogen-purge) water.*p < 0.05, **p < 0.01, ***p < 0.001, and ****p < 0.0001.

2-16. It is good to list contaminants from MS analysis, however, the presence of numerous fatty acids from ZIP-tip desalting seems unusual, and why are they present in such abundance?

Response: Thank you for this question. In C18-based ZipTip desalting, trace hydrophobic background species originating from sample handling materials, solvents, or the ZipTip resin itself can be retained and subsequently detected by MS, appearing as fatty acid–like signals. Similar background signals associated with C18 cleanup have been reported previously[39]. To clarify the origin of these peaks, we performed negative control experiments. The same fatty acid–like signals were observed in control samples containing only 5T ssDNA in DI water and in DI water alone, both processed using the identical, standard ZipTip desalting protocol.[40]), indicating that these signals originate from the desalting workflow.

To clarify this point, the following has been added to the Main manuscript and Supplementary Discussion:

Main manuscript (page 19):

We added the following to the Methods, MS measurement (Method 8): "Control samples containing only 5T ssDNA in DI water (denoted as Negative control 5T ssDNA) and DI water alone (denoted as DI water) were processed through the identical ZipTip desalting procedure, which includes going through Wash solution #1 (0.1 M triethylammonium acetate (TEAA), pH 7.0), Wash solution #2 (Milli-Q water), and elution with 50% acetonitrile/Milli-Q water. "

Supplementary Table 5:

The theoretical results are from simulation of Chemdraw Professional (Version 23.1.1.3). Some peaks observed in the MS spectra are not attributable to DNA fragments and cannot be assigned using standard MS databases, which are most likely derived from fatty acid–like residues introduced during the ZipTip desalting process. Most fatty acid may come from ziptip desalt column; they are commonly used in silica modification. Other background chemicals may come from instrument cleaning (e.g. surfactants) or other experimental sources."

The Supplementary figures:

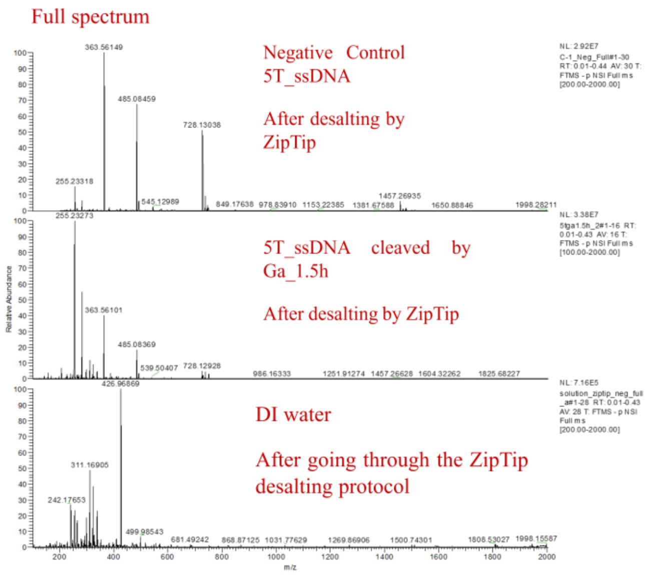

Supplementary Fig. 22. Full MS spectrum of negative control 5T ssDNA, 5T ssDNA cleaved by Ga_1.5h and DI water.

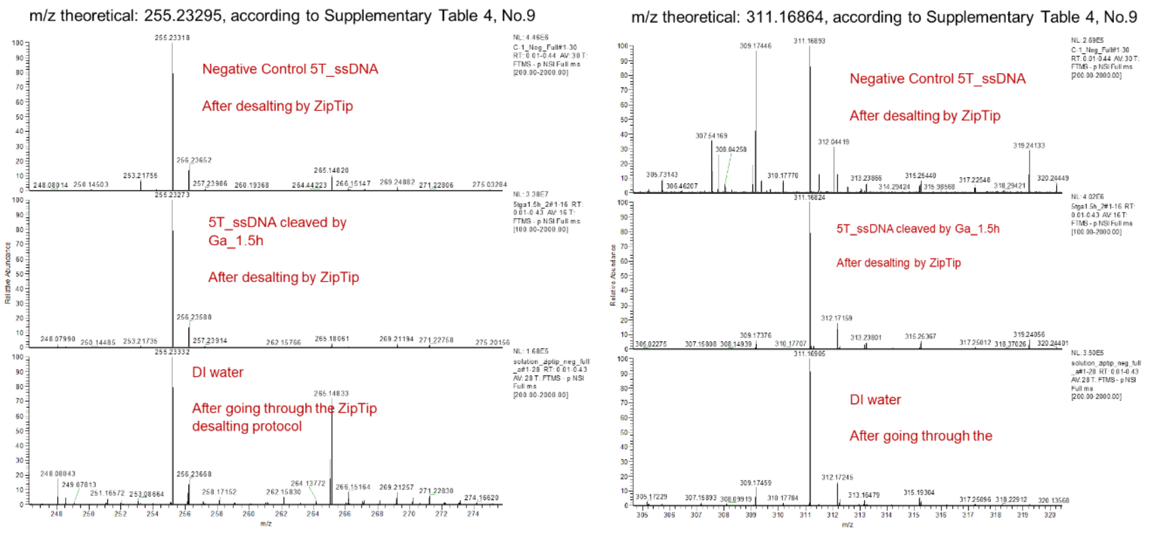

Supplementary Fig. 23. Enlarged MS spectrum of negative control 5T ssDNA, 5T ssDNA cleaved by Ga_1.5h and DI water, all go through the identical Ziptip protocol. The same fatty acid-like peaks were also observed in negative controls (Negative control 5T_ssDNA and only DI water processed through the identical and standard ZipTip protocol), indicating that these signals originate from the desalting workflow.

2-17. Clarify whether ICP-MS or -OES was used. The reader finds contradictory information about the ICP technique.

Response: Thank you for the careful check. We have revised the typo “ICP-OES, PerkinElmer, USA” into the correct one “ICP-MS, PerkinElmer, USA”in the Method ICP-MS (Method 7).

Reviewer #3 (Remarks to the Author):

The manuscript "Ga in Liquid State Shows Nuclease-Mimicking Activity" presents a study on the use of liquid Ga for nuclease-like activity with preferred cleavage sites. It is an interesting and unusual work, combining aspects of heterogeneous catalysis and biochemistry. For me, the main serious weakness of this manuscript lies in the proposed role of Ga and its involvement in the cleavage process, which requires further research. Below are several points that could help improve the paper:

3-1. The study relies mainly on fluorescence data for cleavage, without providing kinetic constants, turnover numbers, or activation energies. The paper definitely requires more quantitative kinetic data, if possible, under varying pH, O2 levels, and temperature.

Response: We thank the reviewer for emphasizing the need for a quantitative kinetic framework. We have performed a detailed kinetic analysis of Ga droplet–mediated ssDNA cleavage under varying temperatures and environmental conditions.

The extracted kinetic constants (V_{max}, K_m), turnover number (TON), and activation energy (E_a) have now been determined and are presented in Supplementary Information and copied below for your convenience:

Supplementary Discussion:

"Supplementary Discussion 1. Kinetic analysis of Ga droplets as nuclease mimic

To quantitatively evaluate the nuclease-mimic behaviour of Ga droplets, we performed fluorescence-based steady-state kinetic analysis of ssDNA cleavage (Supplementary Fig. 3). The reaction rates exhibited clear substrate-saturation behaviour and could be described using an apparent Michaelis–Menten framework, yielding maximum reaction velocity (V_{max}) and michaelis constant (K_m) values (Supplementary Table 2; Supplementary Method 2).

Although Ga droplets do not possess discreet, well-defined active sites analogous to protein enzymes, the extracted parameters provide a rigorous quantitative description of the maximal cleavage velocity and the effective substrate affinity of the reactive interface. The observed saturation behaviour suggests that the rate-limiting step involves the interfacial association of DNA with the Ga-oxide surface prior to radical-mediated scission. By applying this framework, we demonstrate that the Ga-droplet platform effectively recapitulates the kinetic signature of natural nucleases, providing a high-efficiency pathway for DNA degradation.

Arrhenius analysis of the temperature-dependent kinetic parameters yielded an apparent activation energy of 16.87 $kJ \cdot mol^{-1}$, which is substantially lower than the activation energy typically reported for uncatalyzed phosphodiester hydrolysis of DNA (~90–120 $kJ \cdot mol^{-1}$)[41]. This value is comparable to activation energies reported for radical-mediated Fenton-type reactions (typically ~20–40 $kJ \cdot mol^{-1}$)[42] and falls within the range of apparent activation energies observed for enzymatic nucleases and artificial nuclease systems (~10–60 $kJ \cdot mol^{-1}$)[43,44]. This significant reduction in energetic barrier supports the conclusion that Ga droplets function as effective nuclease mimics, facilitating DNA backbone cleavage through an accelerated pathway. The lowered apparent energy activation is consistent with a mechanism in which surface-mediated processes, such as substrate adsorption, electronic interactions with the Ga surface, and ·OH generation, collectively reduce the energetic cost of phosphodiester bond scission.

It is important to note that the activation energy derived here represents an apparent activation energy, as the experimentally determined kinetic parameters are influenced by Ga droplet concentration, particle size distribution, and surface oxidation state. Accordingly, the reported value should be interpreted as a system-specific reference rather than an intrinsic elementary reaction barrier.

The cleavage efficiency of Ga droplets was further evaluated by estimating an apparent turnover number (TON) following conventions commonly used in organometallic and heterogeneous reagent-mediated scission. Because only the surface of Ga droplets is considered reactive and the number of accessible active sites cannot be rigorously quantified, the resulting TON is necessarily an apparent turnover number and is provided for reference. The relatively low apparent TON observed under the present conditions is attributed to progressive surface oxidation of Ga droplets in aqueous environments (Supplementary Discussion 3), which limits sustained activity over multiple cycles (Supplementary Fig.21). Previous studies have demonstrated that Ga oxide can be removed or reduced through chemical or electrochemical treatments followed by re-sonication, enabling regeneration of reactive Ga droplets[2,3]. While such regeneration strategies could, in principle, extend the functional lifetime of the platform, they were not explored in this work to maintain focus on elucidating the intrinsic DNA cleavage behaviour.

Taken together, Ga droplets function as nuclease mimics by lowering the apparent energetic barrier for DNA cleavage. Nevertheless, their mode of action is fundamentally different from that of canonical enzymes, as it is dictated by surface chemistry and materials properties rather than by the precise spatial arrangement of a discrete active site. By bridging the gap between heterogeneous inorganic chemistry and biochemical function, this platform provides a unique, ligand-free alternative for achieving accelerated DNA scission."

Supplementary Figures:

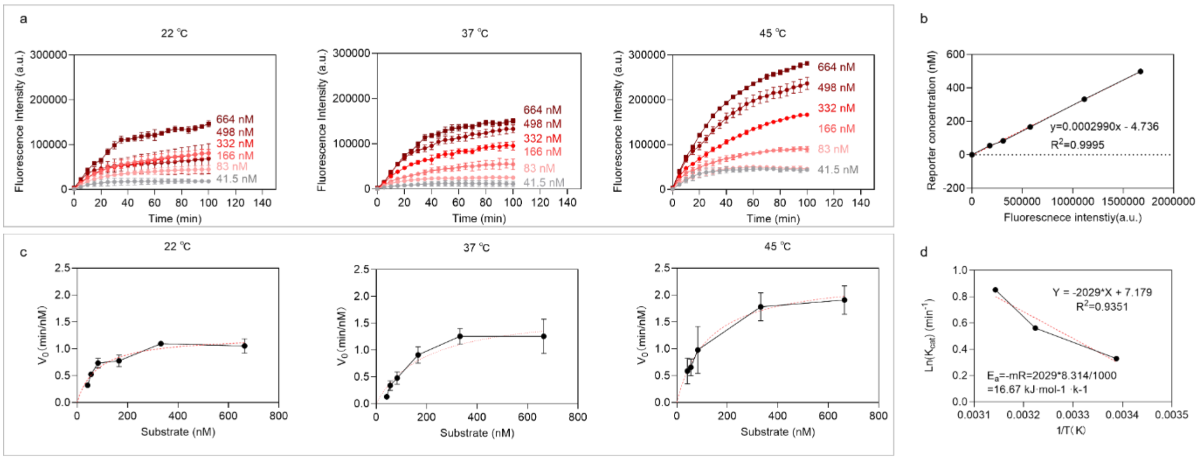

Supplementary Fig.3 (a) Kinetic performance of Ga droplets at 22 °C, 37 °C, and 45 °C, shown as time-dependent fluorescence increases of TR-ssDNA reporters at increasing substrate concentrations.(b) Relationship between fluorescence intensity and TR-ssDNA reporter concentration, with excess S1 nuclease used to define the maximal fluorescence corresponding to complete substrate cleavage.(c) Initial reaction rates (v_0) extracted from the early linear regions of the progress curves and fitted using the Michaelis–Menten model to obtain apparent kinetic parameters $V_{\max}$and K_m. (d) Arrhenius plots derived from temperature-dependent kinetic parameters, used to calculate the apparent activation energy of Ga droplet–mediated ssDNA cleavage.

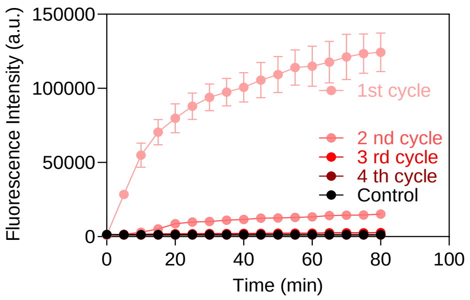

Supplementary Fig. 21. The cleavage effect of Ga droplets on TR-ssDNA reporters after reusing.

Supplementary Tables:

Supplementary Table 2. Kinetic parameters for Ga droplets acting as artificial nuclease mimics.

Supplementary Table 2-1. **Michaelis–Menten parameters (V_{max} and K_m)**

Michaelis-Menten	22 °C	37 °C	45 °C
Best-fit values			
Vmax	1.325	1.752	2.343
Km	159.1	195.9	123.4
95% CI (profile likelihood)			
Vmax	1.159 to 1.543	1.419 to 2.225	1.935 to 2.884
Km	108.4 to 238.3	117.7 to 335.2	72.25 to 214.1
Goodness of Fit			
Degrees of Freedom	26	20	16
R squared	0.8751	0.8341	0.8206
Sum of Squares	0.3635	0.7449	1.157
Sy.x	0.1182	0.193	0.2689

Supplementary Table 2-1. Parameters used for apparent activation energy determination.

T(°C)	Vmax (nM/min)	Km (nM)	T(K)	Kcat (min^{-1})	1/T	Ln（Kcat）
22	1.325	158.9	295.15	1.325	0.003388	0.281412459
37	1.752	145.7	310.15	1.752	0.003224	0.560757993
45	2.343	616.8	318.15	2.343	0.003143	0.85143216

The influence of pH was further evaluated using HCl and NaOH (Response Figure 2); however, the observed trends cannot yet be fully rationalized. Ongoing studies on Ga droplet interactions with sugars

suggest that the identity of the acid or base can substantially modify the surface chemistry of Ga droplets, resulting in markedly different reactivity profiles. Consequently, the effect of pH cannot be readily decoupled from specific acid–surface interactions. A more systematic investigation and mechanistic elucidation of these effects are therefore required but are beyond the scope of the present study.

The influence of dissolved oxygen has also been examined and is presented in Supplementary Fig. 15d. Its role in hydroxyl radical (·OH) generation and the associated DNA cleavage process is discussed in detail in the context of the proposed mechanism. Please refer to the answer for Q 3-4for more details.

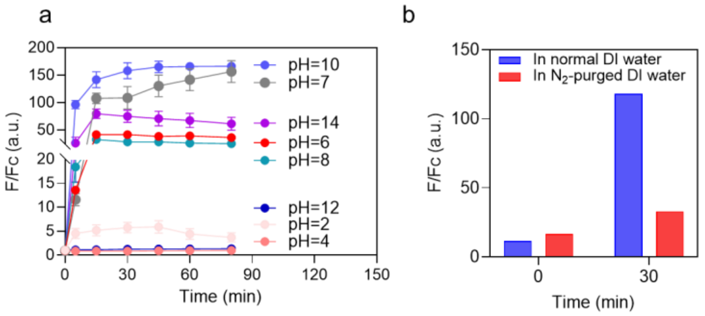

Response Figure 2. DNA cleavage performance of Ga droplets under a. different pH conditions and b. oxygen levels (ambient O_2 vs reduced O_2).

Supplementary Methods:

The methods for conducting the above experiments have been added to Supplementary Method 1:

Kinetic analysis of ssDNA cleavage by Ga droplets (Supplementary Method 1)

Fluorescence intensity $F(t)$(a.u.) from TR-ssDNA reporters was used to quantify the extent of ssDNA cleavage. To convert fluorescence signals into product formation, excess S1 nuclease was added at each temperature to achieve complete reporter cleavage, and the corresponding fluorescence intensity was taken as the maximum signal (F_{max}) for that temperature. For each substrate (TR-ssDNA reporter) concentration, the maximum fluorescence intensity was obtained and used as a reference for data normalization (Supplementary Fig.3).

Determination of initial reaction rates (v_0)

Initial reaction rates were determined from the early linear region of the fluorescence–time progress curves. A time window was selected in which the fluorescence increase was linear, and substrate depletion was negligible. The initial rate $v_0$was approximated as:

$$v_0 \approx \frac{F_{\text{corr}}(t_2) - F_{\text{corr}}(t_1)}{t_2 - t_1}$$

where $F_{\text{corr}}(t)$is the background-corrected fluorescence intensity at time t.

Determination of apparent $V_{\text{max}}$and K_m

Steady-state kinetic parameters were determined independently at each temperature by fitting the initial rate data to the Michaelis–Menten model:

$$v_0([S], T) = \frac{V_{\text{max}}(T)\,[S]}{K_m(T) + [S]}$$

where [S] is the TR-ssDNA reporter concentration (nM), v_0 is the initial rate (a.u.·min^{-1} or nM·min^{-1}), $V_{\max}(T)$ is the apparent maximum reaction rate at temperature T, and $K_m(T)$ is the apparent Michaelis constant. Nonlinear regression of v_0 versus $[S]$ was used to extract $V_{\max}$ and K_m. By definition, K_m corresponds to the substrate concentration at which $v_0 = V_{\max}/2$.

Apparent turnover rate constant (k_{cat})

An apparent turnover rate constant was defined as:

$$k_{\text{cat}} = \frac{V_{\max}}{[E]_{\text{eff}}}$$

where $[E]_{\text{eff}}$ represents the effective concentration of active Ga droplets. Because the effective concentration was identical for all temperatures, $[E]_{\text{eff}}$ was treated as a constant. Taking the natural logarithm:

$$\ln k_{\text{cat}} = \ln V_{\max} - \ln[E]_{\text{eff}}$$

The constant term $\ln[E]_{\text{eff}}$ affects only the intercept and does not influence the temperature-dependent slope used to extract the activation energy. Therefore, for Arrhenius analysis, the effective concentration was set to unity, and $k(T)$ was taken as $V_{\max}(T)$.

Activation energy determination by Arrhenius analysis

The apparent activation energy was determined using the Arrhenius equation:

$$k(T) = A\, e^{-E_a/(RT)}$$

Taking the natural logarithm gives:

$$\ln k = \ln A - \frac{E_a}{R}\frac{1}{T}$$

where T is the absolute temperature (K), calculated as $T(\text{K}) = T(^\circ\text{C}) + 273.15$, and R=8.314 J mol^{-1} K^{-1}. Arrhenius plots were constructed by plotting $\ln k$ (with $k = V_{\max}$) against $1/T$. Linear regression ($y = b + mx$) was performed, and the activation energy was obtained from the slope:

$$E_a = -mR$$

Activation energies were reported in kJ·mol^{-1}.

Apparent turnover number (TON)

In chemical catalysis, the turnover number (TON) is defined as the total number of substrate molecules converted per catalyst unit before deactivation:

$$\text{TON} = \frac{n_{\text{product}}}{n_{\text{catalyst}}}$$

In this study, the amount of cleaved ssDNA reporter was 41.7 ± 4.2nM, and the Ga concentration was 0.1 mg·mL^{-1}, corresponding to 1,434,246.9 nM. Because only the surface of Ga droplets is considered

active for cleaving DNA, the TON reported here is an apparent turnover number and is provided for reference only. The apparent TON was calculated to be $2.9 \pm 0.29 \times 10^{-5}$.

3-2. It is unclear whether Ga acts catalytically or stoichiometrically (i.e., is consumed during oxidation). To clarify this, recycling experiments should be performed to confirm whether Ga deactivates or retains its activity.

Response: We thank the reviewer for the opportunity to clarify whether Ga acts catalytically or stoichiometrically in this system. We performed recycling experiments using Ga droplets with an ssDNA reporter and quantitatively evaluated their reusability in terms of the turnover number (TON), following the conventional definition used in chemical catalysis. The results show that the Ga droplets could be reused for up to two cycles, yielding an apparent TON of $2.9 \pm 0.29 \times 10^{-5}$(see details in response to the last question **Q3-1**).

The limited reusability arises from progressive surface oxidation of Ga droplets in aqueous environments (see response to next question **Q3-3**). As the oxide layer grows beyond a critical thickness, it increasingly impedes electron transfer from the Ga^0 core, thereby suppressing •OH generation (mechanism discussed in **Q3-4**) and consequently inhibiting DNA cleavage.

We concluded that Ga acts stoichiometrically and revise the catalytic related description in the Main manuscript:

Main manuscript (Page 1)

"Compared with previously reported artificial metallonuclease, the liquid gallium uniquely integrates its oxide layer for substrate adsorption and its Ga^0 core with electrons as a cleavage active center, forming a ligand- and cofactor-free artificial nuclease platform."

Main manuscript (Page 16)

"…liquid Ga droplets uniquely utilize their native oxide layer for substrate adsorption and the electron-rich Ga^0 core as the active center, forming a scaffold-free, metal-only nuclease-mimic platform."

3-3. The surface of liquid Ga particles is typically covered by a thin oxide film in aqueous environments. Although the authors state that Ga0 is the active species, the reaction conditions strongly suggest that the reactions occur at the oxide–liquid interface. A clearer surface analysis (e.g., XPS, FTIR) would strengthen this claim. Atomic-resolution TEM of droplet surface evolution would also be very helpful.

Response: Thank you for the valuable suggestion about the Ga droplets surface evolution. To address this, we performed detailed SEM imaging and XPS analysis on Ga droplets incubated with DNA in deionized (DI) water.

Our findings indicate that: in aqueous environments, Ga droplets undergo a time-dependent transformation. Prolonged exposure (24h) leads to the transformation of spherical Ga droplets into rod-shaped GaOOH structures (see Supplementary Fig. 19). This oxidation is closely related to the ·OH formation mechanism therefore, it has been integrated the corresponding mechanistic discussion.

More details to back up this answer can be found in the Supplementary Information, and are provided below for your convenience:

Supplementary Figure:

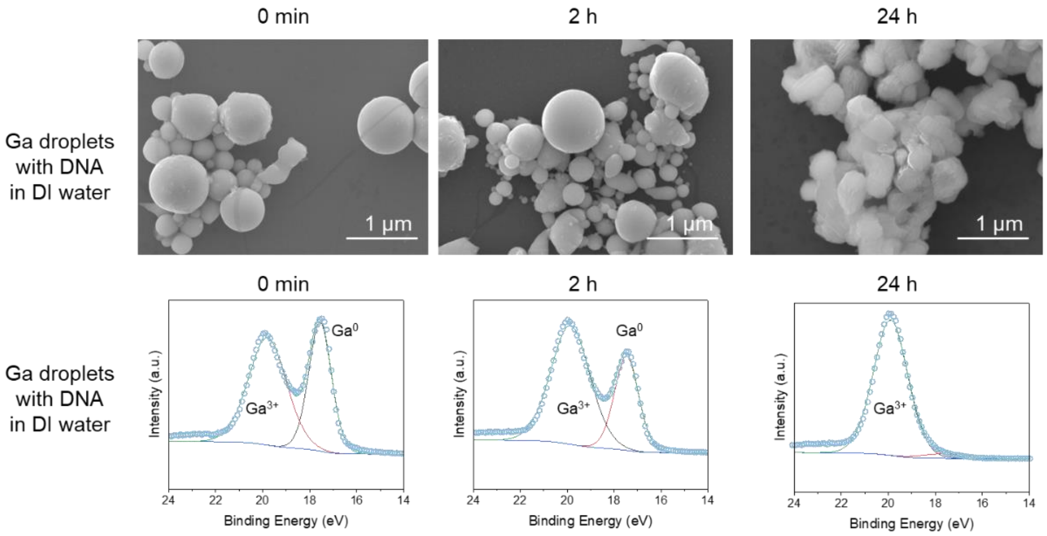

Supplementary Fig. 9. SEM images and Ga 3d XPS spectra of Ga droplets incubated with the TR-ssDNA reporter for 0 min, 2 h, and 24 h; SEM images show a gradual transformation of spherical droplets into rod-like structures; XPS results show a progressive oxidation of metallic Ga^0, with Ga^0 no longer detectable after 24 h. Based on this characteristic morphology and prior reports, the oxidized phase is assigned to GaOOH[1-3].

Supplementary Discussion:

Supplementary Discussion 3. Oxidation State Evolution of Ga Droplets

Upon the transfer from ethanol to DI water, the formation of ·OH is accompanied by the oxidation of Ga^0 into Ga. After 24 h in DI water (Supplementary Fig. 19), SEM images reveal a gradual morphological transformation of Ga droplets into rod-like oxide structures. Correspondingly, the Ga 3d XPS spectrum exhibits a dominant peak at ~20 eV, characteristic of Ga^{3+} species. Together, these results indicate the formation of GaOOH [1-3]. Consistently, after 24 h, radical signals were no longer detectable by EPR, and DNA cleavage activity was fully suppressed (Supplementary Fig. 20).

Based on these observations, together with the ·OH signals detected in Fig. 3 and prior literature, the reaction pathway of Ga droplets in water is summarized below.

Upon contact with water, two reactions occur: First, Ga droplets were prepared in EtOH, with Ga_2O_3 present on their surface. Upon exposure to aqueous solution, Ga_2O_3 gradually transforms into Ga $(OH)_3$ then GaOOH *via* Reaction 1[2,3,23].

Reaction 1:

$$Ga_2O_3 + 3H_2O \rightarrow 2Ga(OH)_3$$

$$Ga(OH)_3 \rightarrow GaOOH + H_2O$$

Based on ·OH detection in the EPR and previous research on GaOOH, we first propose the following reaction pathway (reaction 2). The corresponding reduction potentials and potential ROS chain reactions are provided in Supplementary Table 4.

Reaction 2:

$$2Ga + 2H_2O + O_2 \rightarrow 2GaOOH + H_2$$

$$2Ga \rightarrow 2Ga^{3+} + 6e^- \qquad (1)$$
$$O_2 + 2e^- + 2H_2O \rightarrow 4 \cdot OH \qquad (2)$$
$$2Ga^{3+} + 4 \cdot OH + 4e^- \rightarrow 2GaOOH + H_2 \qquad (3)$$

The above equation indicates that electron transfer from Ga droplets to dissolved oxygen in the presence of water is essential for hydroxyl radical generation.

3-4. The proposed OH-assisted cleavage is interesting, but the origin of OH radicals is not clear. Usually, such radicals are generated in photocatalysis; here, it appears they might arise from the oxidation of Ga by water, but the mechanism is not well explained. Radicals could also form sonochemically during redispersion of Ga, but they are unlikely to persist long enough to react. This pathway and the mechanism of OH radical generation should be studied in greater detail.

Response: We thank the reviewer for the opportunity to clarify the origin of the ·OH radicals. To summarize, the generation of ·OH is an intrinsic redox process driven by the spontaneous oxidation of the metallic gallium (Ga^0) core in the presence of dissolved oxygen and water. Critically, while brief sonication of Ga droplets with DMPO (5 s) in the EPR test is used to ensure uniform droplet dispersion, our control experiments (Fig.3d; Supplementary Fig.11) confirm that sonication is not the source of radical generation. Instead, the primary role of sonication is to mechanically disrupt the passivating oxide layer, thereby increasing the exposed metallic surface area and enhancing the intrinsic interfacial redox activity of the Ga droplets.

More details to back up this answer can be found in the revise manuscript and Supplementary discussion, and are provided below for your convenience:

Main manuscript (Page 6):

Mechanism of the Nuclease-Mimicking Activity of Ga droplets

Identification of Ga^0 as the Active Species

The system was primarily composed of metallic Ga (Ga^0) and DNA. Although Ga^{3+} ions (Fig. 3a; Method 6) and Ga oxides can coexist under these conditions[2], rigorous control experiments showed that neither species contributed significantly to the observed activity. Specifically, chelating Ga^{3+} with ethylenediaminetetraacetic acid (EDTA) did not significantly alter DNA cleavage efficiency (Fig. 3b), and Ga^{3+} alone showed no cleavage activity (Supplementary Fig. 9a). Similarly, Ga oxides were also inactive when tested independently for DNA cleavage (Supplementary Fig. 9b, c, 10; Supplementary Method 3). These results confirm that Ga^0 is the central species driving DNA cleavage, prompting further investigation into the interfacial reactions between Ga^0 and the aqueous environment.

EPR Characterization of Radical Intermediates

To elucidate how Ga^0 mediates DNA cleavage, we investigated whether Ga^0 -associated reactive oxygen species (ROS) generated in aqueous solution[27,28] contribute to this process. Electron paramagnetic resonance (EPR) spectroscopy and signal simulations (Method 7 and Supplementary Method 4) were performed to detect these intermediates. To clarify the role of sonication, we compared the cleavage intensity and EPR signals of Ga droplets transferred to deionized (DI) water with and without sonication (Fig. 3c-e). The results show that Ga droplets can cleave DNA and generate ROS without sonication. However, brief sonication enhanced this activity by improving droplet dispersion, disrupting the surface oxide layer, and increasing interfacial exposure, as evidenced by the intensified EPR signals and increased cleavage efficiency (Supplementary Fig. 11; Fig.3c).

The EPR spectra (Fig. 3d, e) revealed a mixture of 5,5-Dimethyl-1-pyrroline N-oxide (DMPO) adducts: DMPO-OH, DMPO-OOH, and DMPO-CH(CH_3)OH adducts, indicating the generation of hydroxyl radical (·OH) [29], superoxide ($O_2{\cdot}^-$)[30,31], and 1-hydroxyethyl radicals (CH(CH_3)OH·) [32], respectively. The $CHCH_3OH$· signal likely stems from trace EtOH [33] in the Ga stock solution. Due to the short half-life of DMPO-OOH ($t_{1/2}$ = 60 s)[34], this signal disappeared after a 90-min dead time, leaving a clear and prominent ·OH signal (Fig. 3f). No ·OH signal was detected in the Ga-free controls (Supplementary Fig. 12 a, b), and the 2,2,6,6-tetramethylpiperidine (TEMP) probing ruled out singlet oxygen (Supplementary Fig. 13). These results collectively demonstrate that Ga^0-water system intrinsically produces ·OH and $O_2{\cdot}^-$.

Validation of ·OH as the Primary Cleavage Agent

To identify which of these radicals involved in this cleavage process, we performed a series of scavenger experiments. The addition of EtOH to probe the involvement of $CHCH_3OH$· had no effect on cleavage (Supplementary Fig. 14), and the $O_2{\cdot}^-$ scavenger superoxide dismutase (SOD) actually increased the signal (Fig. 3g) likely by accelerating the conversion of $O_2{\cdot}^-$ to hydrogen peroxide (H_2O_2)[36] and subsequently into ·OH. Conversely, the ·OH scavenger dimethyl sulfoxide (DMSO), which reacted with ·OH substantially faster than with $O_2{\cdot}^-$ [37,38] markedly suppressed the DNA cleavage (Fig. 3h). Moreover, the direct involvement of ·OH was further confirmed by the observed decrease of ·OH signal as the concentration of DNA increased (Supplementary Fig. 15a). Collectively, these results confirm that ·OH radicals are the primary species responsible for the Ga droplets-mediated DNA cleavage, while $CHCH_3OH$· is negligible and $O_2{\cdot}^-$ is indirect. Finally, the suppression of cleavage under hypoxia (nitrogen-purged) or water-depleted (glycerol environment) conditions confirms that the presence of both oxygen and water is essential for the interfacial generation of the ·OH radicals, which is required for this nuclease-mimicking activity (Supplementary Fig. 15b-d).

Proposed mechanisms for ·OH formation

Based on our experimental results and established redox kinetics, we propose that the ·OH formation (Fig. 3i) is driven by the potent electron-donating capacity of liquid Ga[45,46]. Upon contact with the aqueous interface, electrons from metallic Ga^0 core facilitate the stepwise reduction of dissolved oxygen, initiating a radical cascade forming $O_2{\cdot}^-$, H_2O_2 and ultimately ·OH as shown in the EPR result (Fig.3d- f; Supplementary Table 4). Concurrently, the Ga droplets undergo gradual oxidation to GaOOH (Supplementary Discussion 3).

We identify two primary pathways that facilitate the flux of electrons from the liquid Ga^0 core to the aqueous interface. The first one can be described as quantum tunneling across the native oxide. In this case, initially, the oxide layer formed on Ga droplets in EtOH is sufficiently thin to allow quantum tunneling[47,48]. Within this oxide layer, the near surface electron states below the Fermi level establish an internal electric field that facilitates electron transport[49,50]. Upon transfer to water, the Ga^0 core donates electrons through this thin shell to dissolved oxygen in the water, initiating ROS generation. However, as the oxide layer thickens beyond a few nanometers, the electric field weakens, rendering the process self-limiting and eventually blocking further electron transport[50,51]. To validate this tunneling-dependent activity, we compared Ga droplets with varying degrees of oxide thicknesses. We first examined samples stored in EtOH for one day *vs* one month. X-ray photoelectron spectroscopy (XPS) analysis revealed that while Ga droplets aged for one month retain a dominant metallic Ga^0 core, they exhibited a significantly higher Ga^{3+} contribution compared to the one-day sample (Supplementary Fig. 16). This chemical shift was also concurrent with SEM observations of less rounded morphologies, indicating thickened oxide layers. Functionally, the DNA cleavage activity of the one-month-old droplets remained detectable but was reduced compared to freshly prepared droplets (Supplementary Fig. 17). This demonstrates that while a thickened oxide layer substantially suppresses electron flux, it does not achieve total passivation. To further isolate the effect of oxide thickness, we prepared Ga droplets under nitrogen to minimize the oxidation (denoted as Ga droplets with thinner oxide layers),

or under air bubbling to promote it (denoted as Ga droplets with thicker oxide layers). Upon transferring to aqueous DNA solutions, the droplets with thinner oxide layers exhibited significantly higher radical generation and cleavage efficiency than those with thicker ones (Supplementary Fig. 18,19). Collectively, these results provide evidence that the initial nuclease activity is likely mediated by electron tunneling across the oxide interface, a process that is progressively hindered as the dielectric barrier thickens.

The second alternative or concurrent pathway for the flux of electrons can be due to the mechanical surface renewal *via* oxide cracking. This involves the mechanical breakage of the oxide shell, which enables direct contact between the metallic Ga^0 core and the aqueous environment. In water, the oxidation of Ga induces significant volumetric changes[52,53] (Supplementary Discussion 3; Supplementary Fig.20, 21) that generate interfacial strain[54], leading to the cracking of the surface oxide layer[55]. This mechanical rupture continuously exposes fresh, unpassivated Ga^0 surfaces to the surrounding solution. By maintaining a direct Ga^0-water interface, this process facilitates unhindered electron transfer and ·OH production. While tunneling is critical during the initial contact phase, this surface renewal mechanism ensures that reactive metallic sites remain accessible even as oxidation proceeds.

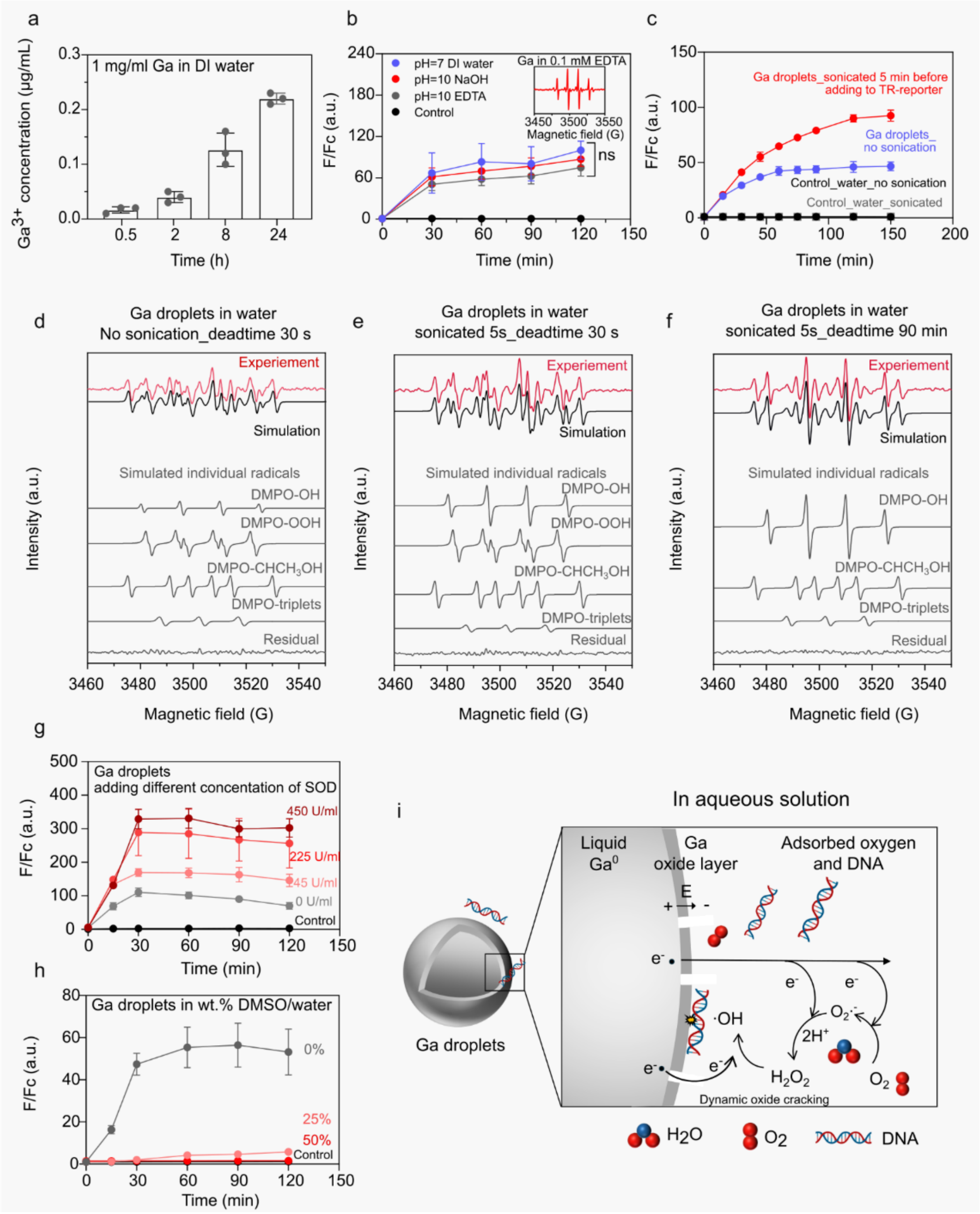

Figure 3. | ROS generation of Ga droplets in aqueous solution. a. Inductively coupled plasma–mass spectrometry (ICP-MS) analysis showing time-dependent release of Ga^{3+} ions from Ga droplets in deionized water. b. Fluorescence intensity of TR-ssDNA reporters after treatment with Ga droplets in the presence of EDTA under chelating-active conditions (pH = 10). Inset figure: EPR spectra of Ga droplets in 0.1 mM EDTA. (a.u = arbitrary units). c. Comparison of the nuclease-mimicking activity of Ga droplets with and without sonication before incubation with the ssDNA TR-reporter. d-f. EPR spectra of Ga droplets in water without sonication (d), and after brief sonication (5 s), measured following different delay times (30 s (e) and 90 min (f); dead time defined as the interval between sample preparation, sealing, equipment setting and EPR data acquisition). Corresponding simulated spectra are shown for comparison (see Supplementary Method 4). g. Effect of SOD, a superoxide scavenger, on the nuclease-mimicking activity of Ga droplets, evaluated using the TR-reporter. h. Effect of ·OH quenchers, DMSO, on the nuclease-mimicking activity of Ga droplets, using the TR-reporter. i. Schematic illustration of ·OH generation by Ga droplets in aqueous solution.

Main manuscript (Page 19) :

EPR spectroscopy (Method 7)

For the detection of ·OH and $O_2·^-$, Ga droplets (40 µL, 1 mg mL⁻¹ in EtOH) were subjected to solvent exchange into DI water. Briefly, EtOH was removed by centrifugation, followed by the addition of DI water and gentle pipetting to redisperse the Ga droplets prior to the experiment. DMPO was purified by treatment with activated charcoal, filtered, and stored as frozen aliquots prior to use. Then DMPO (4 µL, 100 mM) was then added. Samples were measured without sonication or after sonication for 5 s or 30 s. Negative control samples contained only DI water and DMPO, with and without identical sonication. The positive control was the Fenton reaction; experimental details are described in the section on Fenton reaction–mediated DNA cleavage. EPR spectra were recorded after defined dead times (0-90 min) following sample preparation, as specified in each figure, to capture the temporal evolution of $O_2·^-$ to ·OH.

For the detection of singlet oxygen, Ga droplets (40 µL, 1 mg mL⁻¹ in EtOH) were solvent-exchanged into DI water. TEMP was freshly distilled at 152°C and was then added. Samples were measured directly without sonication. Negative control samples containing only DI water with TEMP were prepared. Positive control samples consisted of TEMP mixed with 50 µM methylene blue and irradiated with red light (>630 nm) to induce photosensitized singlet oxygen ($^1O_2$) generation.

All samples were immediately transferred into glass capillaries after different treatment and sealed with wax. Spectra were acquired at room temperature using a Bruker EMX X-band spectrometer (microwave power 20 mW; scan width 200 G).

Supplementary Figures:

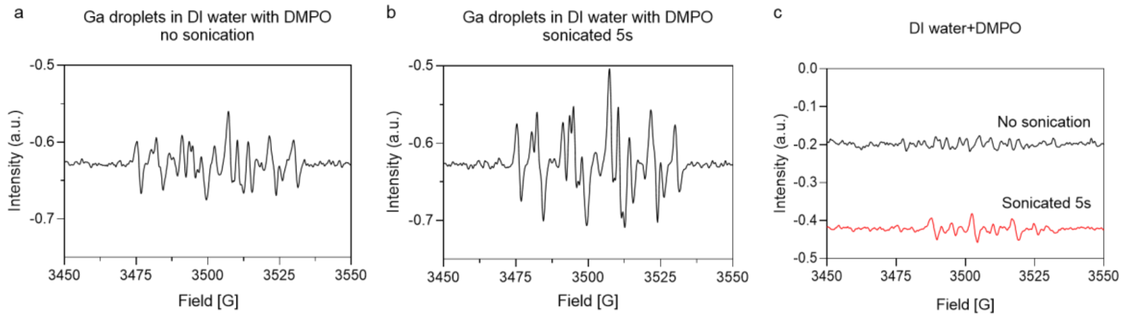

Supplementary Fig. 11. EPR spectra of Ga droplets in DI water in the presence of DMPO: **(a)** no sonication, **(b)** sonicated for 5 s. **(c)** Control spectra of DI water + DMPO without sonication and after

5 s sonication. Dead time = 30s. The triplet signal observed in DI water after 5 s sonication indicates that it originates from sonication-induced partial degradation of DMPO.

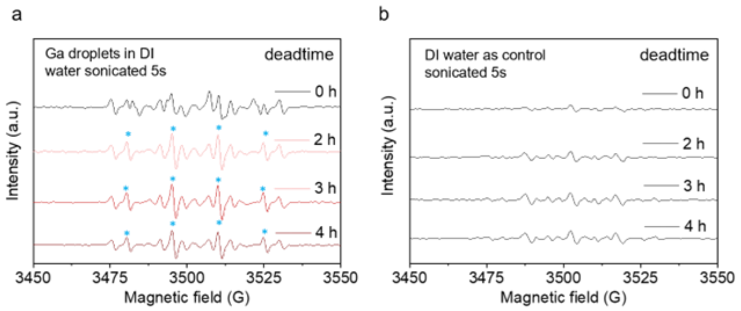

Supplementary Fig. 12. Electron paramagnetic resonance (EPR) spectra of **(a)** Ga droplets in DI water, **(b)** DI water as control; dead time defined as the interval between sample preparation, sealing, equipment setting and EPR data acquisition: spectra were recorded at different time points following sample preparation.

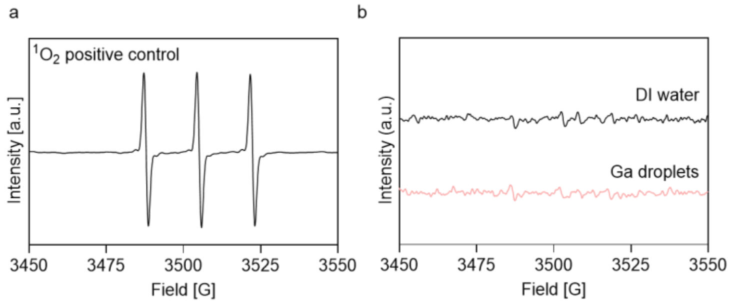

Supplementary Fig. 13. EPR spectra of **(a)** methylene blue (MB) with TEMP under red-light irradiation as a positive control for singlet oxygen generation, and (b) DI water and Ga droplets in DI water with TEMP. No detectable signal was observed in the Ga droplet samples, indicating that Ga droplets do not generate singlet oxygen.

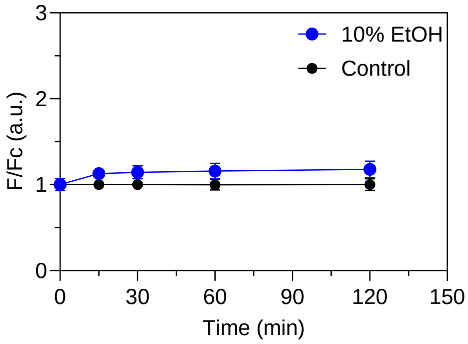

Supplementary Fig. 14. Incubation of TR-ssDNA reporter with 10% EtOH after 5s sonication showing no cleavage effect, indicating the contribution of $CH_3CH_2O\cdot$ is negligible.

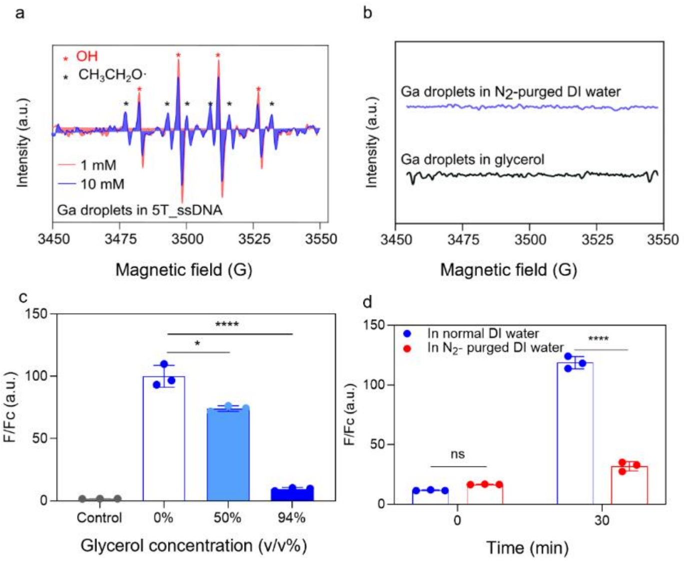

Supplementary Fig. 15. (a) EPR spectra of Ga droplets in 1 mM or 10 mM 5T_ssDNA; deadtime 90 min. (b) EPR spectra of Ga droplets in oxygen-depleted water (nitrogen-purge) and water-depleted (glycerol) condition, showing suppressed radical formation. TR-ssDNA reports cleavage activity of Ga droplets in (c) water-depleted (glycerol) condition and (d) oxygen-depleted water (nitrogen-purge) water. *$p < 0.05$, **$p < 0.01$, ***$p < 0.001$, and ****$p < 0.0001$.

Supplementary Methods:

Supplementary Method 4. Electron paramagnetic resonance (EPR) spectral analysis and simulation

Continuous-wave X-band EPR spectra were analyzed using the SpinFit module in Xenon (Bruker BioSpin), which employed perturbation theory, with the electron Zeeman interaction treated as the dominant term and isotropic hyperfine coupling included as a first-order perturbation. Experimental spectra were fitted using multicomponent simulations to account for overlapping signals arising from different 5,5-dimethyl-1-pyrroline N-oxide (DMPO) spin-trapped radical adducts. Hyperfine coupling constants, including the g value of each radical adduct, were initially loaded from the SpinFit library. A single isotropic g value was assumed for each radical species. Least-squares fitting was used to optimize hyperfine coupling constants, line positions, line widths, and relative component intensities simultaneously, enabling estimation of the relative contributions of individual radical species. The quality of the fits was assessed by inspection of the residual spectra, defined as the difference between the experimental and simulated spectra. Following spectral simulation, the hyperfine coupling constants of each DMPO radical adduct were compared with values reported in the NIEHS spin trap database: https://www.niehs.nih.gov/ research/resources/databases/spintrap.

The hyperfine coupling constants used in the simulations were as follows. 1. Ga in water without sonication: DMPO-hydroxyl radical (DMPO-OH) (aN = 15.05 G, aH = 14.45 G), DMPO-hydroperoxyl radical (DMPO-OOH) (a_N = 14.15 G, a_H^{β} = 11.27 G, a_H^{γ} = 1.21 G), DMPO-1-hydroxyethyl (DMPO-$CH(OH)CH_3$)· (a_N = 15.87 G, a_H = 22.97 G), and a triplet attributed to partially degraded DMPO: a_N = 14.91 G; 2. Ga in water sonicated 5s_deadtime 30s: DMPO-OH (a_N = 14.99 G, a_H = 14.55 G), DMPO-OOH (a_N = 14.11 G, a_H^{β} = 11.31 G, a_H^{γ} = 1.22 G), DMPO-$CH(OH)CH_3$ (a_N = 15.87 G, a_H = 22.96 G), and a triplet attributed to partially degraded DMPO: a_N = 15.02 G; 3. Ga in water sonicated 5s_deadtime 90 min: DMPO-OH (a_N = 14.94 G, a_H = 14.57 G), DMPO-$CH(OH)CH_3$ (a_N = 15.85 G, a_H = 22.88 G), and a triplet attributed to partially degraded DMPO: a_N = 14.66 G.

For the DMPO-OOH adduct, because DMPO spin trapping cannot distinguish between superoxide radical ($O_2^{\cdot-}$) and its protonated form, the hydroperoxyl radical (·OOH), radical species assignment is

guided by the solution pH[35]. Under neutral conditions (DI water), superoxide ($O_2^{\cdot-}$) predominates ($O_2^{\cdot-} + H^+ \rightleftharpoons \cdot OOH$, $pK_a \approx 4.9$), and the DMPO-OOH signal is therefore attributed to $O_2^{\cdot-}$ [30,35].

3-5. If OH radicals produced from water are involved in cleavage, hydroxyl groups should appear in the degraded DNA. I recommend performing experiments with labeled H2O17 and detecting in corporation by NMR to confirm the proposed mechanism.

Responses: We thank the reviewer for this valuable suggestion. The use of $H_2^{17}O$ NMR to track oxygen incorporation is indeed a powerful method for studying traditional nucleophilic phosphodiester hydrolysis. However, based on our mechanistic evidence, we believe this specific experiment would not provide a definitive validation for the following reasons:

We have proven that the ·OH radicals are generated primarily through the reduction of dissolved oxygen O_2 rather than the dissociation of water molecules (supported by the formation of $O_2^{\cdot-}$ in Fig.3d). Therefore, as shown in the redox chain in Supplementary Table 4, the oxygen atoms in the resulting ·OH radicals originate from the O_2 dissolved in water reduced by the electron from Ga^0.

Supplementary Table 4. Potential reactions and ROS chain reactions triggered by the introduction of Ga droplets into DI water.

Half reaction	Redox potential* (E_0) [V]
$Ga - 3e^- \rightarrow Ga^{3+}$	0.55
$O_2 + e^- \rightarrow O_2^- \cdot$	-0.16
$O_2^- \cdot + 2H^- + e^- \rightarrow H_2O_2$	0.89
$H_2O_2 + H^+ + e^- \rightarrow H_2O + \cdot OH$	0.38

*pH=7.0 [56]

And then the proposed mechanism of Ga droplets nuclease-mimic activity based on new added MD calculation's adsorption (the oxygen from gas O_2 contributes to ·OH-involved hydrolysis) (Fig.4g):

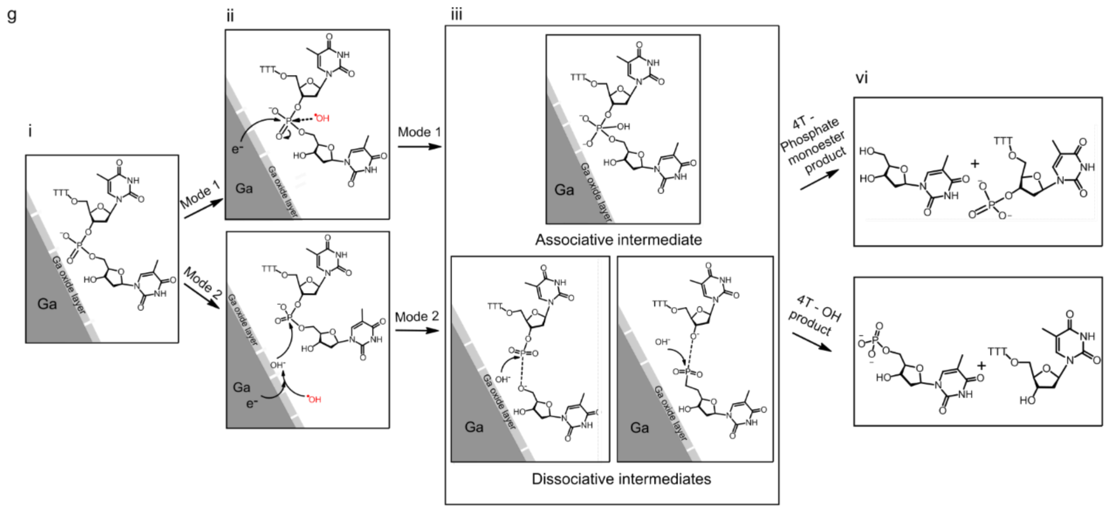

Fig. 4g. Proposed mechanism of Ga-induced DNA cleavage.

While traditional hydrolysis of phosphodiester obeys another path using water or hydroxide ions[57] (Response figure.3):

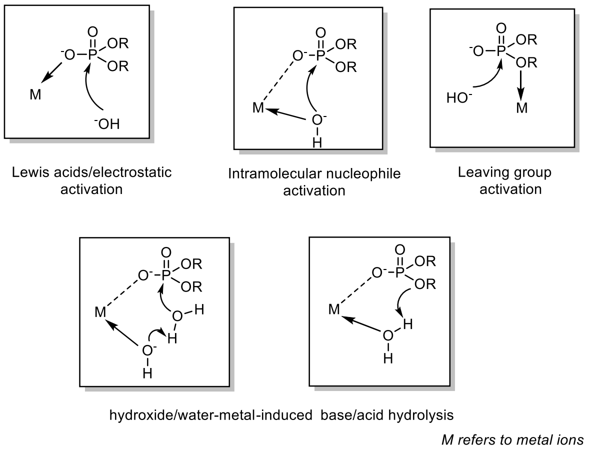

Response figure.3. Schematic illustrating the traditional hydrolytic pathway of phosphodiester bond cleavage mediated by water or hydroxide ions.

Consequently, even in high-purity $H_2{}^{17}O$, the degraded DNA would predominantly incorporate ^{16}O from the dissolved gas, leading to a false-negative result for incorporation.

Furthermore, even if we use the $^{17}O_2$, two significant technical hurdles remain to obtain a strong ^{17}O signal:

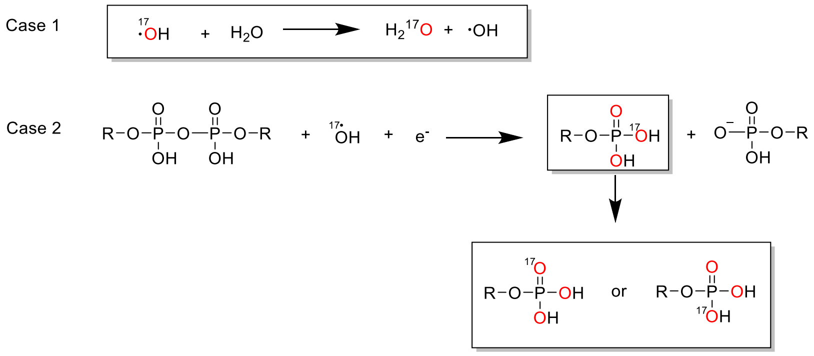

Response figure.4. Schematic illustration of the possible oxygen (^{17}O) incorporation pathways in NMR measurements.

First, ·OH can undergo H-transfer with water[58], which lowers the concentration of ^{17}OH radicals (Response figure.4, Case 1). In addition, the hybridized-orbital structure of phosphate can dilute the ^{17}O abundance in the NMR signal (Response figure.4, Case 2). It is also worth noting that case 1 may also lead to signal response when using the $H_2{}^{17}O$ to support a falsification (ideally, if $H_2{}^{17}O$ shows no ^{17}O signal, this falsification would further support our radical-involved hypothesis, however it cannot be achieved according to the two cases mentioned).

To validate the proposed mechanism, we performed additional Electron paramagnetic resonance (EPR) and mass spectrometry (MS) experiments to provide more evidence. Please refer to the responses to Q3-4 (EPR results for the mechanism of •OH generation and its key role in DNA cleavage) and Q3-6 (MS results for the evidence that Ga droplets cleave phosphate diester without the formation of oxidative fragments, and the proposed reaction pathway) for further details.

3-6. OH radicals alone (without Ga) might also cause DNA cleavage. Model tests using OH radicals generated by H2O2 or other methods could help clarify the specific role of Ga versus pure OH radicals.

Response: Thank you for the valuable suggestion. To distinguish the role of the Ga interface from that of ·OH generated by H_2O_2**,** we performed a comparative fragmentation analysis using a classical Fenton reaction (Fe–EDTA/H_2O_2) as a positive control for pure ·OH-mediated oxidative cleavage.

As detailed in our revised manuscript and Supplementary Discussion 3, high-resolution MS analysis reveals a fundamental difference in the chemical fingerprint of the two systems. In the Fenton system (Fig.4d-e; Supplementary Fig.26), we observed a wide array of oxidative base-modified fragments (e.g., base propenals and sugar-ring oxidation products), which are the hallmark of non-specific radical attack on DNA[5]. In contrast, these oxidative products were entirely absent in the Ga treated group. Instead, the Ga droplets exclusively produced fragments derived from phosphodiester bond scission, yielding hydroxyl and phosphate termini (Fig.4b-e). This indicates that while ·OH radicals are involved, the Ga-interface provides a protective or directed environment that favors a hydrolytic-like cleavage pathway over the typical oxidative degradation characteristic of Fenton chemistry.

Overall, the combined results support the proposed mechanism as shown in Fig.4g, which will present in the next question.

The detailed information to back up the above discussion can be found in the revised manuscript and supplementary information, and provided as below for your convenience:

Discussion (Main manuscript, page 8)

Mass Spectrometry Fingerprinting of DNA Fragmentation

"To elucidate the DNA cleavage mechanism of Ga droplets, we compared their fragmentation patterns with those generated by a classical ·OH-based Fenton reaction using high-resolution mass spectrometry (MS) (Fig. 4a-e; Method 8; Supplementary Method 5). Relative to the control, both Ga-treated and Fenton-treated samples showed a marked decrease in the intact 5T ssDNA ion (*m/z* 363.56, z = -4), accompanied by the appearance of 4T fragments at *m/z* 576.10 and 616.09 (z = -2) (Supplementary Figs. 22, 23; Supplementary Tables 5-6). These ions correspond to two specific cleavage products: a 4T fragment bearing two hydroxyl termini and a second fragment containing one hydroxyl and one phosphate monoester terminus (Supplementary Fig. 24; Supplementary Tables 7-10; Supplementary Discussion 4).

To assess the involvement of oxidative pathways, expected fragments arising from typical metallonuclease mechanisms, including hydrogen abstraction and nucleobase oxidation, were predicted and used for targeted *m/z* screening (Supplementary Fig. 25; Supplementary Tables 11, 12). Oxidative base-modified fragments were detected in the Fenton reaction, whereas no such species were observed in Ga-treated samples (Supplementary Fig. 26). This agrees with established Fenton chemistry, in which ·OH radicals preferentially attack nucleobases, causing secondary cleavage of the phosphodiester backbone[5,7]. In contrast, Ga droplets exclusively generated phosphodiester bond-derived cleavage products, with no detectable nucleobase or sugar-ring oxidation, showing similar hydrolytic nuclease activity. These results further provide evidence that Ga droplets cleave DNA *via* an OH-assisted hydrolytic mechanism targeting the phosphodiester bond, rather than through oxidative degradation characteristics of classical Fenton systems."

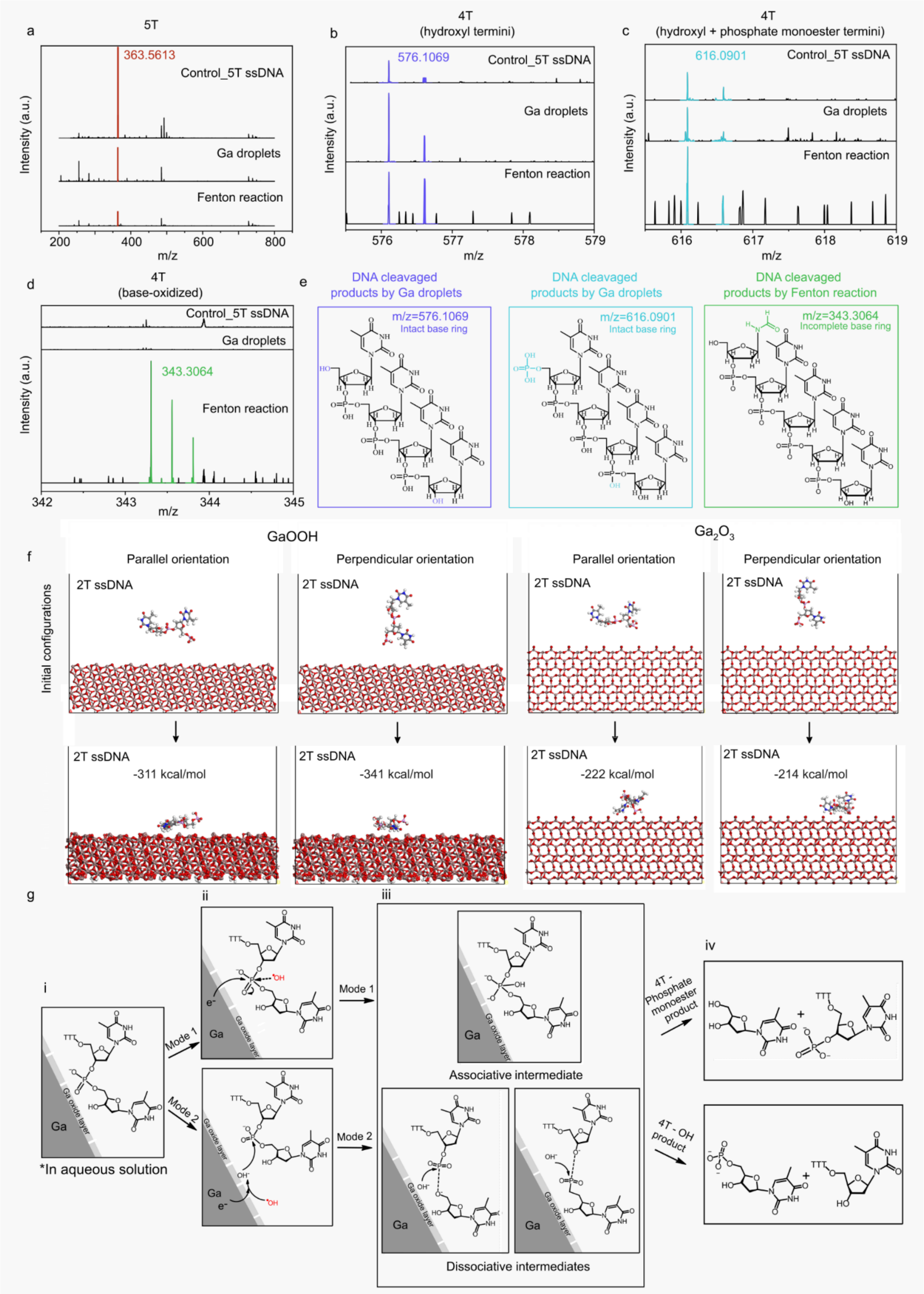

Fig.4 | Mechanism of Ga droplets' nuclease-mimicking effect: ROS-assisted hydrolytic cleavage activity. a-d. MS analysis of 5T ssDNA fragments after Ga droplets or Fenton reaction treatment: (a) full spectrum showing fragmentation pattern with 5T ssDNA peaks; (b) zoom-in of 4T fragments with two hydroxyl termini; (c) zoom-in of 4T fragment with hydroxyl and phosphate monoester termini; (d)

zoom-in of 4T fragment with oxidized base. In control samples, some hydrolytic-like peaks were observed due to in-source decay and high-voltage ionization[8,9]. The analysis of the 3T-to-1T fragments is provided in Supplementary Discussion 3. e. 4T fragment structures corresponding to m/z in b-d. f. MD simulations of 2T-ssDNA interacting with the Ga oxide layer, Ga_2O_3 and GaOOH, using two initial configurations: ssDNA perpendicular or parallel to the surface. The water molecules were made invisible for clarity of visualization g**.** Proposed mechanism of Ga-induced DNA cleavage.

Main manuscript (Page 19)

MS measurement (Method 8)

In addition, a Fenton reaction was performed as a positive control for oxidative DNA cleavage, as described in Supplementary Method 5.

Supplementary Method:

Fenton reaction–mediated DNA cleavage (Supplementary Method 5)

ssDNA (2 mg mL^{-1}) was incubated with Fe^{2+} (from $NH_4Fe(SO_4)_2 \cdot 12H_2O$, 20 µM), ascorbic acid (0.1 mM), and hydrogen peroxide (H_2O_2 ,10 mM) to induce oxidative cleavage. Reactions (total volume, 20 µL) were carried out for 2 h or 16 h and quenched by adding 2 µL of 1 M ethylenediaminetetraacetic acid (EDTA), followed by brief mixing. Samples were allowed to stand for 10–20 min to ensure complete metal chelation, placed on ice to suppress residual reactions, desalted using ZipTip, and analyzed using an LTQ Orbitrap XL mass spectrometer equipped with static nanospray (Thermo Scientific, USA).

Supplementary Figure:

Product 1 ↓:

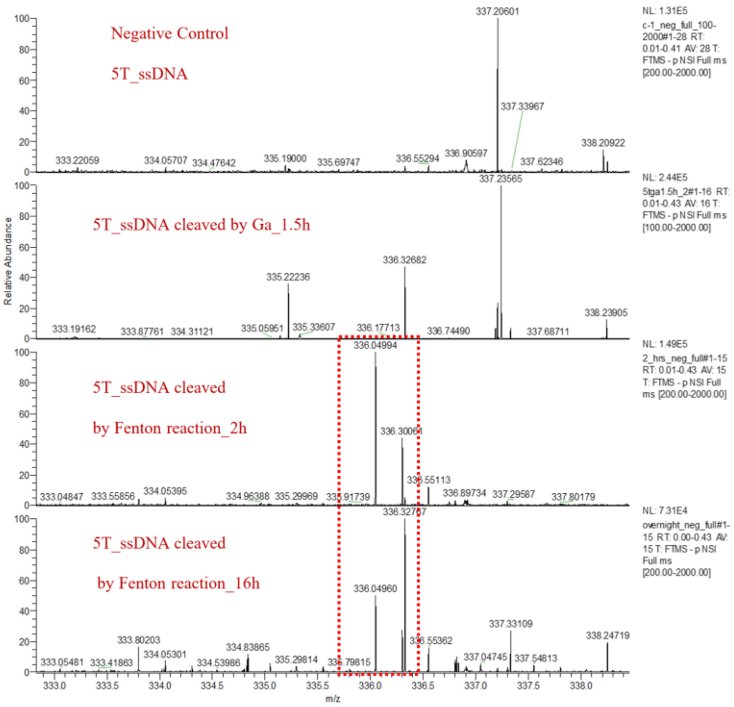

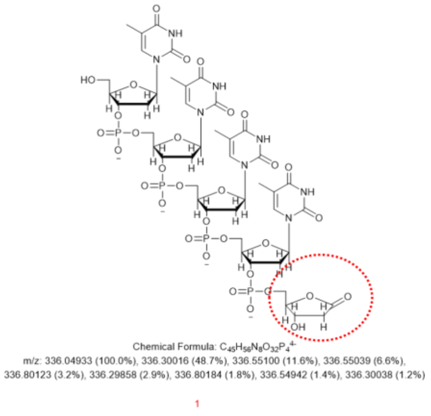

Product 2 ↓:

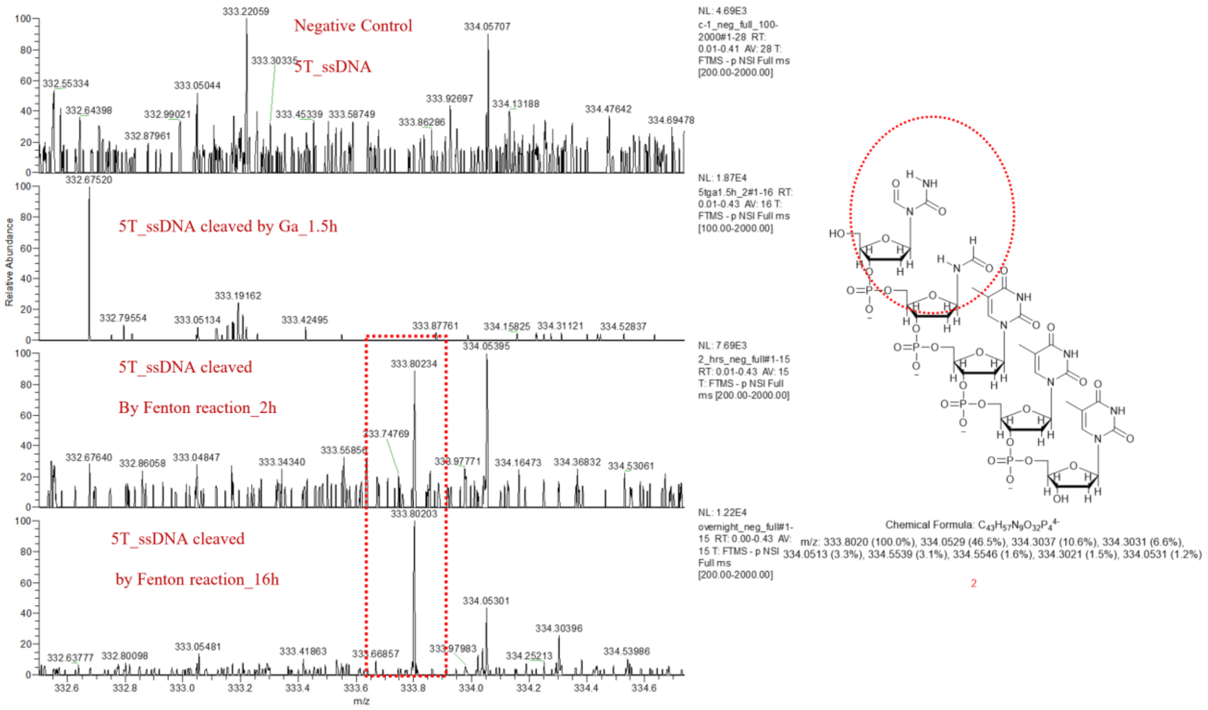

Product 3 ↓:

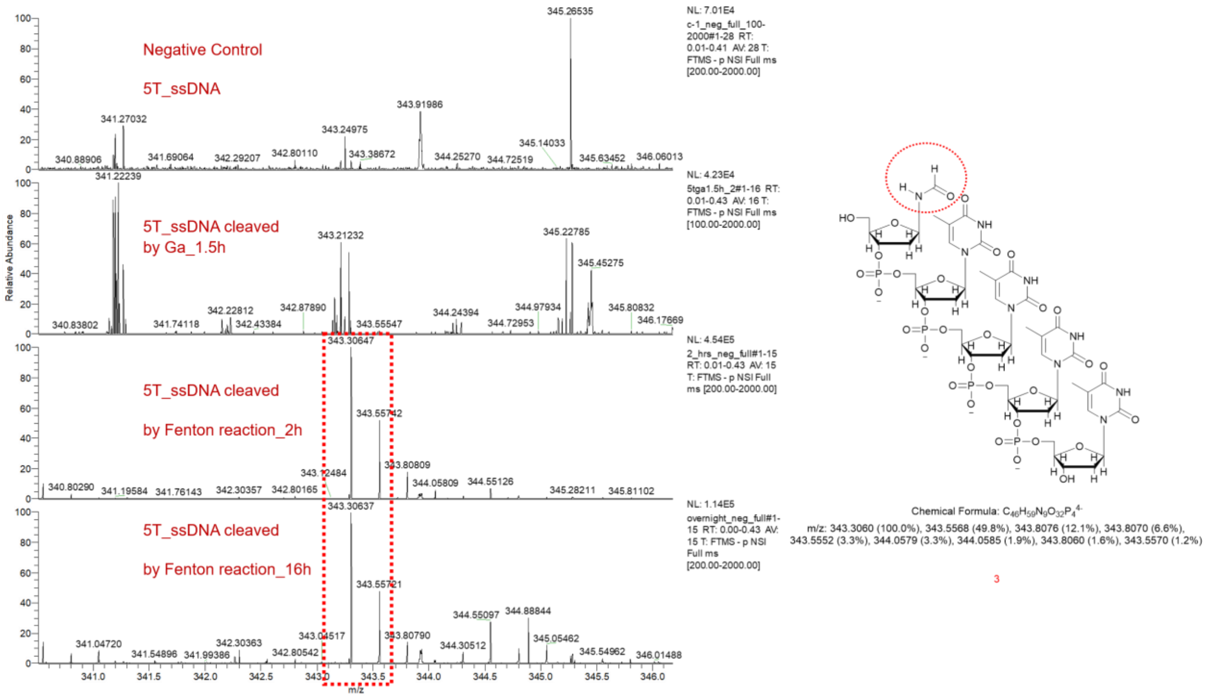

Product 4 ↓:

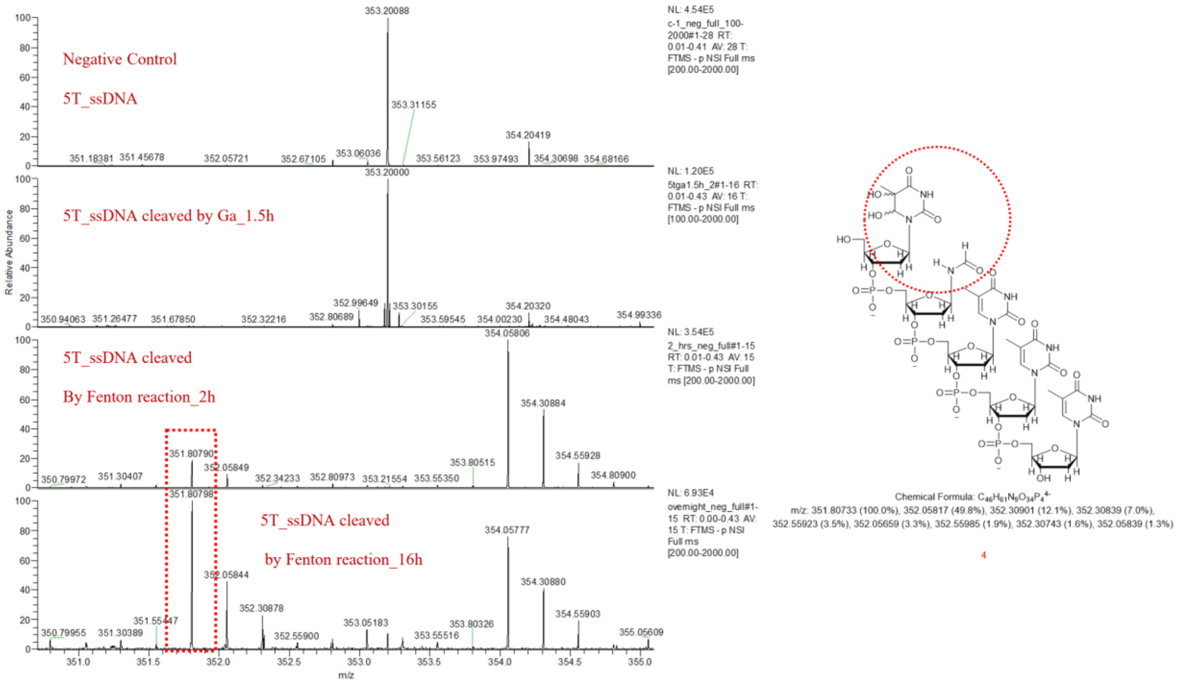

Product 5 ↓:

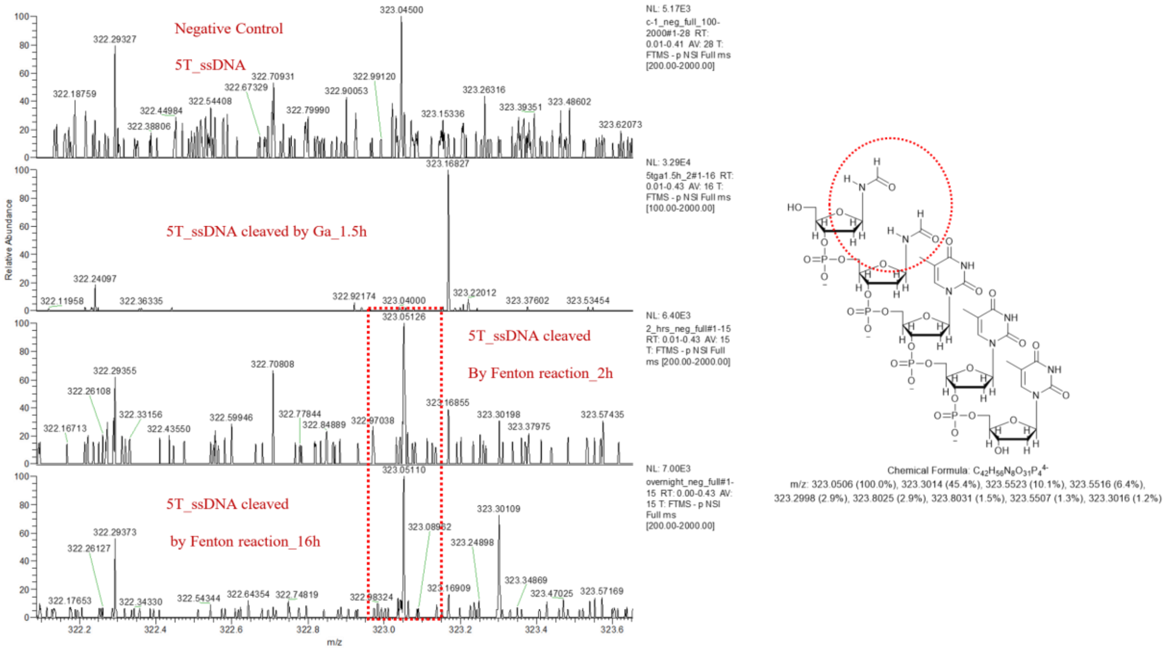

Supplementary Fig. 26. Oxidative fragments identified only in Fenton cleavage with MS and supposed structure are listed (the position of fragmented nucleobases may vary within the 5T sequences).

Supplementary Table:

Supplementary Table 12. Calculated m/z values of theoretical fragments: product 1 to 5 in Supplementary Fig.26.

These tables summarize the screened data from this procedure. Additional data are available upon request; representative main peaks and structures are shown in Supplementary Fig.26.

Supplementary Table 12-1. (only hydroxyl-group residues at the DNA fragment termini, from product

1 to 5)

Product 1	Expected MS	-H'	-2H+Na'	-2H/2'	-3H+Na'	-3H/3	-4H+Na'	-4H'
5T	1348.2264	1347.2191	1369.2016	673.1059	684.09715	448.4015	455.729	336.0493
4T	1044.18036	1043.17306	1065.15556	521.08288	532.07413	347.05282		
3T	740.13432	739.12702	761.10952	369.05986				
2T	436.08828	435.08098						
T	NA							
Product 2	Expected MS	-H'	-2H+Na'	-2H/2'	-3H+Na'	-3H/3	-4H+Na'	-4H'
5T	1339.2373	1338.23	1360.2125	668.61135	679.6026	445.40513	452.73263	333.80202
4T	1035.19126	1034.18396	1056.16646	516.58833	527.57958	344.05645		
3T	731.14522	730.13792	752.12042	364.56531				
2T	427.09918	426.09188						
T	NA							
Product 3	Expected MS	-H'	-2H+Na'	-2H/2'	-3H+Na'	-3H/3	-4H+Na'	-4H'
5T	1377.253	1376.2457	1398.2282	687.6192	698.61045	458.07703	465.40453	343.30595
4T	1073.20696	1072.19966	1094.18216	535.59618	546.58743	356.72835		
3T	769.16092	768.15362	790.13612	383.57316				
2T	465.11488	464.10758						
T								
Product 4	Expected MS	-H'	-2H+Na'	-2H/2'	-3H+Na'	-3H/3	-4H+Na'	-4H'
5T	1411.2584	1410.2511	1432.2336	704.6219	715.61315	469.41217	476.73967	351.8073
4T	1107.21236	1106.20506	1128.18756	552.59888	563.59013	368.06349		
3T	803.16632	802.15902	824.14152	400.57586				
2T	499.12028	498.11298						
T	NA							
Product 5	Expected MS	-H'	-2H+Na'	-2H/2'	-3H+Na'	-3H/3	-4H+Na'	-4H'
5T	1296.2315	1295.2242	1317.2067	647.10845	658.0997	431.06987	438.39737	323.050575
4T	992.18546	991.17816	1013.16066	495.08543	506.07668	329.72119		
3T	688.13942	687.13212	709.11462	343.06241				
2T	384.09338	383.08608						
T	NA							

Supplementary Table 12-2. (only one hydroxyl-group residue at the DNA fragment termini, from product 1 to 6)

Product 1	Expected MS	-H'	-2H+Na'	-2H/2'	-3H+Na'	-3H/3
5T						
4T	1124.14666	1123.13936	1145.12186	561.06603	572.05728	373.7082533
3T	820.10062	819.09332	841.07582	409.04301		
2T	516.05458	515.04728				
T	212.00854	211.00124				
Product 2	Expected MS	-H'	-2H+Na'	-2H/2'	-3H+Na'	-3H/3
5T						
4T	1115.15756	1114.15026	1136.13276	556.57148	567.56273	370.7118867
3T	811.11152	810.10422	832.08672	404.54846		
2T	507.06548	506.05818				
T	NA	NA				
Product 3	Expected MS	-H'	-2H+Na'	-2H/2'	-3H+Na'	-3H/3
5T						

4T	1153.17326	1152.16596	1174.14846	575.57933	586.57058	383.3837867
3T	849.12722	848.11992	870.10242	423.55631		
2T	545.08118	544.07388				
T	241.03514	240.02784				
Product 4	Expected MS	-H'	-2H+Na'	-2H/2'	-3H+Na'	-3H/3
5T						
4T	1187.17866	1186.17136	1208.15386	592.58203	603.57328	394.71892
3T	883.13262	882.12532	904.10782	440.55901		
2T	579.08658	578.07928				
T	NA	NA				
Product 5	Expected MS	-H'	-2H+Na'	-2H/2'	-3H+Na'	-3H/3
5T						
4T	1072.15176	1071.14446	1093.12696	535.06858	546.05983	356.37662
3T	768.10572	767.09842	789.08092	383.04556		
2T	464.05968	463.05238				
T	NA	NA				

Supplementary Table 12-3. (two hydroxyl-group residues at the DNA fragment termini, from product 1 to 6)

Product 1	Expected MS	-H'	-2H+Na'	-2H/2'
5T				
4T				
3T	900.06692	899.05962	921.04212	449.02616
2T	596.02088	595.01358		
T	291.97484	290.96704		
Product 2	Expected MS	-H'	-2H+Na'	-2H/2'
5T				
4T				
3T	891.07782	890.07052	912.05302	444.53161
2T	587.03178	586.02448		
T	NA	NA		
Product 3	Expected MS	-H'	-2H+Na'	-2H/2'
5T				
4T				
3T	929.09352	928.08622	950.06872	463.53946
2T	625.04748	624.04018		
T	321.00144	319.99364		
Product 4	Expected MS	-H'	-2H+Na'	-2H/2'
5T				
4T				
3T	963.09892	962.09162	984.07412	480.54216
2T	659.05288	658.04558		
T	NA	NA		
Product 5	Expected MS	-H'	-2H+Na'	-2H/2'
5T				
4T				
3T	848.07202	847.06472	869.04722	423.02871
2T	544.02598	543.01868		
T	NA	NA		

3-7. The DFT simulations use isolated Ga atoms, ignoring solvation effects and dynamic oxide surfaces. The authors should discuss how these might affect the calculated results.

Response: We thank the reviewer for this constructive critique regarding the simulation parameters. We agree that isolated Ga atoms in a vacuum do not fully capture the complexity of a dynamic, hydrated oxide interface. To address this limitation, we have expanded our computational framework to include Ga^0, Ga^{3+} species in DFT calculations, as well as oxide phases (Ga_2O_3 and GaOOH) with water molecules in classical molecular dynamics (MD) simulations, thereby providing a more realistic description of the Ga–DNA interaction environment in aqueous systems:

1. Solvation effects and the role of Ga^{3+}:

Ga droplets in aqueous solution can release Ga^{3+} ions, as confirmed by ICP–MS measurements (Fig. 3a). To evaluate whether solvated Ga^{3+} species could contribute to DNA cleavage, we performed DFT calculations on Ga^{3+}-ssDNA systems following the same reaction sequence used for Ga^0. The results indicate that the desorption energy of the Ga^{3+}-ssDNA complex is prohibitively high ($\Delta E \approx 39.58$ eV). This large energy renders Ga^{3+}-assisted phosphodiester cleavage kinetically inaccessible, indicating that Ga^{3+} does not act as an effective reactive centre for DNA backbone scission.

2. Solvation-induced oxide layer evolution:

In aqueous environments, the oxide layer of Ga droplets gradually transforms from Ga_2O_3 to GaOOH, (Supplementary Discussion 3). To compare the influence of Ga_2O_3 and GaOOH, we conducted MD simulations of ssDNA interacting with both Ga_2O_3 and GaOOH surfaces in the presence of water molecules. These simulations show that the DNA phosphate backbone remains accessible to surface even under fully solvated conditions, supporting the feasibility of surface-mediated reactions at hydrated oxide interfaces.

Together, owing to current computational constraints, a unified simulation explicitly incorporating metallic Ga, Ga_2O_3, and GaOOH together with ssDNA is beyond practical feasibility. We therefore employ a complementary strategy in which DFT calculations elucidate intrinsic energetic barriers for key bond-cleavage steps involving Ga^0, whereas MD simulations capture adsorption geometries, hydration effects, and surface variations associated with different oxide phases at the oxide–water interface. By combining these complementary approaches, we bridge the gap between idealized electronic-level descriptions and the stochastic, dynamic nature of the Ga oxide/ssDNA interface in aqueous environments.

The detailed information to back up the above discussion can be found in the revised manuscript and supplementary information, and provided as below for your convenience:

Main manuscript (page 9):

Computational Modeling of DNA Interfacial Adsorption

To further elucidate the role of the Ga interface, we employed multiscale computational approaches to examine the individual contributions of the species present in the system: metallic Ga^0, Ga oxide layer, and free Ga^{3+}. Classical molecular dynamics (MD) simulations were employed to investigate the adsorption behavior of ssDNA on Ga oxide surfaces (Supplementary Table 13; Supplementary Discussion 5; Supplementary Method 6). We evaluated two representative orientations: ssDNA aligned either parallel or perpendicular to the Ga oxide surface (Fig. 4f). The simulations indicate that, given the significant size disparity between the Ga droplets (average diameter ≈ 330 nm) and the ssDNA (~3 nm in length), the DNA molecules preferentially adopt a flat adsorption configuration. This lying-down

orientation on the relatively planar droplet surface facilitates simultaneous interfacial interactions across multiple molecular moieties of the DNA strand.

Density functional theory (DFT) was employed to characterize the adsorption affinity of ssDNA toward both Ga^0 atoms and Ga^{3+} ions (Supplementary Discussion 6). The simulations show that Ga^0 can absorb nucleobases, the sugar ring, and the phosphodiester bond (Supplementary Fig. 27a), mirroring the multi-site, whole-molecule adsorption observed in our MD simulations. Notably, the phosphate groups exhibit a higher adsorption affinity for Ga^0 than the nucleobase nitrogen sites, likely due to enhanced electrostatic and van der Waals interactions. In the presence of ·OH, the cleavage of this phosphate diester bond is energetically favorable (Supplementary Fig. 27b, c; Supplementary Discussion 6; Supplementary Method 7). In contrast, free Ga^{3+} -phosphate interactions lead to the formation of thermodynamically stable Ga-O bonds. These stale bonds effectively 'lock' the structure and do not promote cleavage, even in the presence of ·OH, thereby explaining why Ga^{3+} does not contribute to the system's nuclease-like activity (Supplementary Fig. 28). This finding is fundamentally consistent with our experimental observation in (Fig. 3b; Supplementary Fig. 9a).

Together, these computational and experimental results indicate a dual-pathway mechanism for DNA cleavage: (1) Oxide-mediated adsorption and tunneling: Ga oxides (Ga_2O_3 and GaOOH) act as a scaffold that adsorbs ssDNA, effectively accumulate them at the interfaces. Simultaneously, quantum tunnelling across this native oxide layer can facilitate the necessary electron transfer for ·OH generation, leading to localized DNA cleavage. (2) Direct metallic contact: At sites of oxide rupture, Ga^0 is directly accessible to oxygen, water and ssDNA, enabling localized electron transfer and efficient ·OH formation to mediate the subsequent cleavage of the phosphodiester bond.

Synergistic Mechanism of ·OH-Assisted Hydrolysis

Integrating the results, we propose that Ga-induced hydrolysis of DNA occurs *via* a synergistic multi-step mechanism (Fig. 4g): (1) Interfacial Sequestration (Fig. 4g, i) in which ssDNA is adsorbed onto the Ga droplets surface while Ga droplets generate ·OH radicals through the previously discussed electron-transfer pathways. (2) Radical-Mediated Activation (Fig. 4g, ii-iii) in which the ·OH participates in the reaction *via* two proposed initiation modes as follows: (a) Phosphodiester groups adsorbed on the Ga droplets surface, carrying an increased negative charge that enhances their attraction to ·OH and facilitates direct radical attacks on the phosphorus center (Fig. 4g, ii). (b) Alternatively, ·OH near the Ga droplets surface receives electrons from the metallic core to OH^-, which activate the P=O bond and induce nucleophilic attack by hydroxide ions (Fig. 4g, ii). DFT results suggest that Ga preferentially associates with two oxygen atoms that are not directly connected to the sugar rings, indicating the hydrolysis reacts *via* forming associative nucleophilic intermediates (Fig. 4g. iii), possibly through an associative nucleophilic substitution followed by dissociative elimination, or a concerted associative–dissociative pathway[59]. The synergistic effect of Lewis's acid activation by Ga and nucleophile attack by Ga and nucleophile activation by ·OH or OH^-, underpins the ·OH assisted hydrolytic cleavage mechanism[59,60]. The details of a possible mechanism for Ga droplets' preference to T-T and A-A are provided in Supplementary Discussion 7, Supplementary Fig. 29 and Supplementary Method 8. (3) Hydrolytic Product Formation (Fig. 4g. iv): The reaction concludes with the release of distinct 4T fragments. Mode 1 primarily yields a 4T-phosphate monoester product, preserving the phosphate group on one terminus and mode 2 results in a 4T-OH product, where both cleavage fragments possess hydroxyl termini.

Together, Ga droplets implement DNA cleavage through a combination of adsorption preference and ·OH-assisted phosphodiester hydrolysis. This mechanism differs fundamentally from conventional ROS-induced cleavage, where ·OH typically abstracts hydrogen from the sugar moiety or oxidizes nucleobases[5,7] , resulting in random fragmentation[61]. Instead, Ga droplets' surface-localized activity results in a T-T and A-A cleavage preference while generating fragments with intact phosphate or ribose ring termini[61].

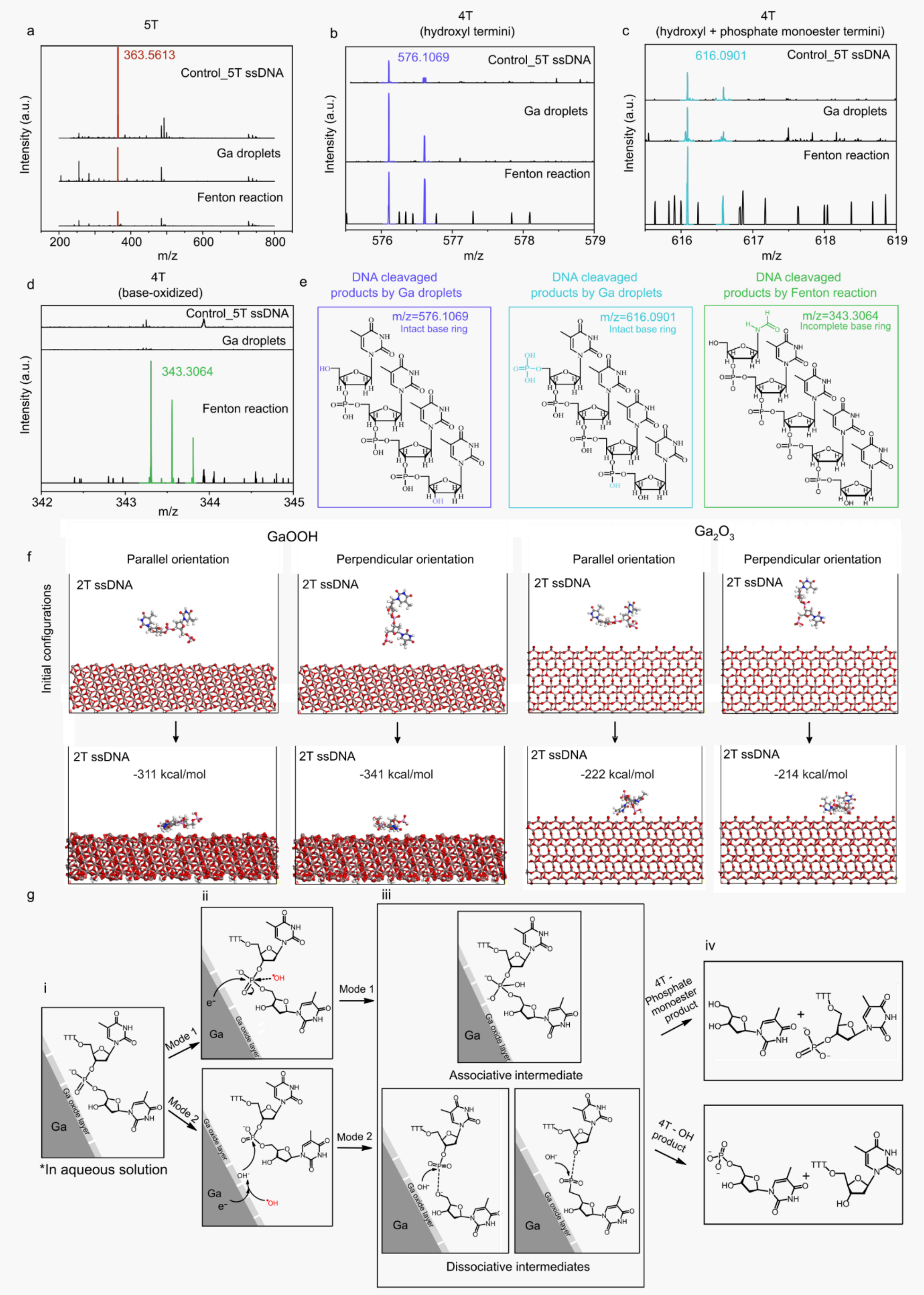

Fig.4 | Mechanism of Ga droplets' nuclease-mimicking effect: ROS-assisted hydrolytic cleavage activity. a-d. MS analysis of 5T ssDNA fragments after Ga droplets or Fenton reaction treatment: (a)

full spectrum showing fragmentation pattern with 5T ssDNA peaks; (b) zoom-in of 4T fragments with two hydroxyl termini; (c) zoom-in of 4T fragment with hydroxyl and phosphate monoester termini; (d) zoom-in of 4T fragment with oxidized base. In control samples, some hydrolytic-like peaks were observed due to in-source decay and high-voltage ionization[8,9]. The analysis of the 3T-to-1T fragments is provided in Supplementary Discussion 4. e. 4T fragment structures corresponding to m/z in b-d. f. MD simulations of 2T-ssDNA interacting with the Ga oxide layer, Ga_2O_3 and GaOOH, using two initial configurations: ssDNA perpendicular or parallel to the surface. The water molecules were made invisible for clarity of visualization **g.** Proposed mechanism of Ga-induced DNA cleavage.

Supplementary Figures:

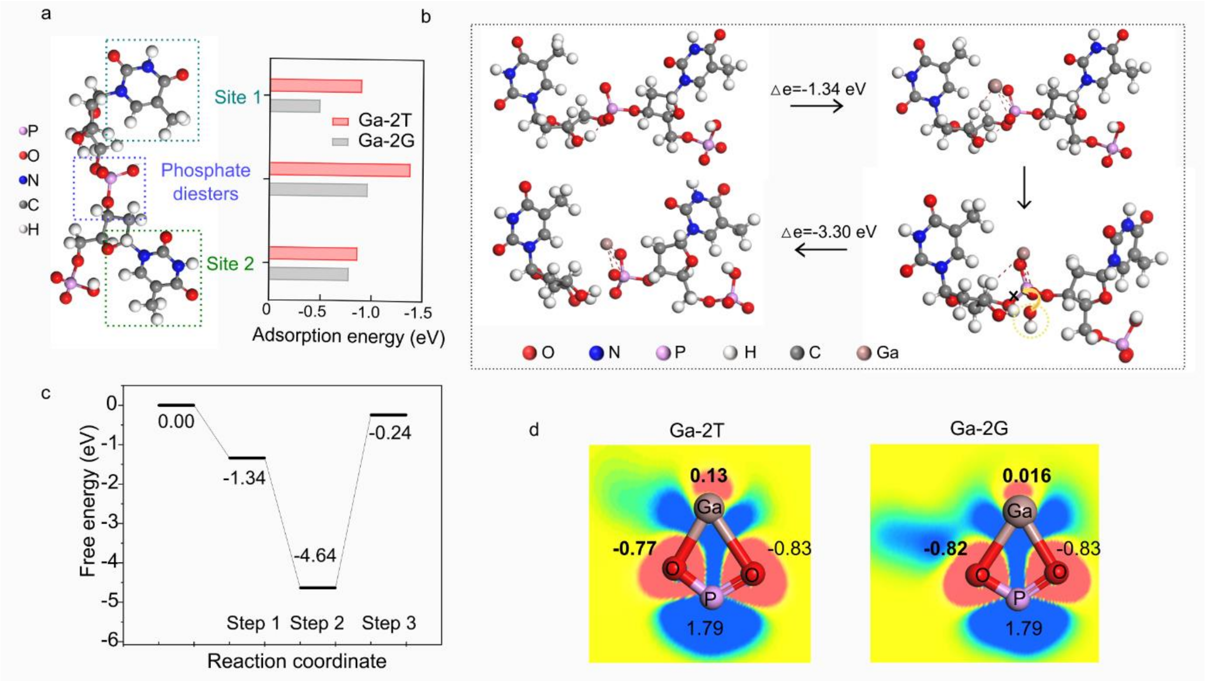

Supplementary Fig. 10. (**a**) Comparison of adsorption energies between Ga and 2T or 2G ssDNA at three representative adsorption sites: phosphate diester bond, base site 1 (near 3'-OH), base site 2 (near 5'-phosphate). (**b**) DFT-calculated reaction pathway, including Ga adsorption, ·OH cleavage, and Ga desorption from 2T ssDNA. (**c**) Calculated reaction energy diagram of 2T ssDNA showing the energy profile for Ga^0-induced cleavage of the phosphate diester bond. (**d**) Charge density difference plot showing the Ga coordinated with the oxygen atoms around the phosphate diester bond of 2T *vs* 2G**.**

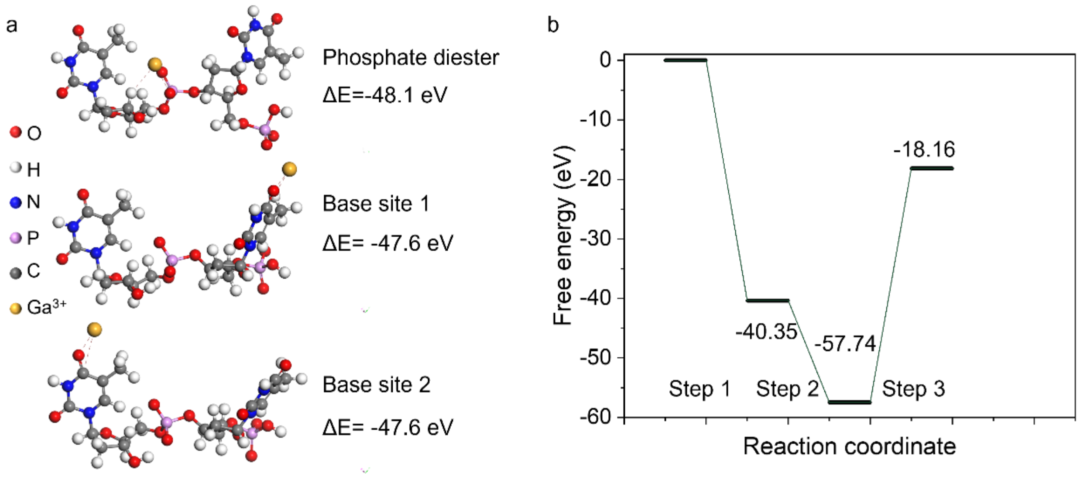

Supplementary Fig. 11. (**a**) Calculated adsorption energies of Ga^{3+} on 2T and 2G ssDNA at three representative binding sites: the phosphate diester group, base site 1 (proximal to the 3′-OH), and base site 2 (proximal to the 5′-phosphate). Ga^{3+} exhibits strong electrostatic coordination to the phosphate moiety relative to nucleobase sites for both sequences. (**b**) Computed reaction energy profile for the Ga^{3+}–2T ssDNA complex in the presence of •OH (Supplementary Method 7). The Ga^{3+}-phosphate interaction excessively stabilizes the post-reaction intermediate, resulting in a prohibitively high desorption barrier for product release ($\Delta E \approx 39.58$ eV for step 3). This large energetic penalty renders Ga^{3+}-assisted phosphodiester cleavage kinetically inaccessible, indicating that Ga^{3+} does not function as an effective center for DNA backbone scission.

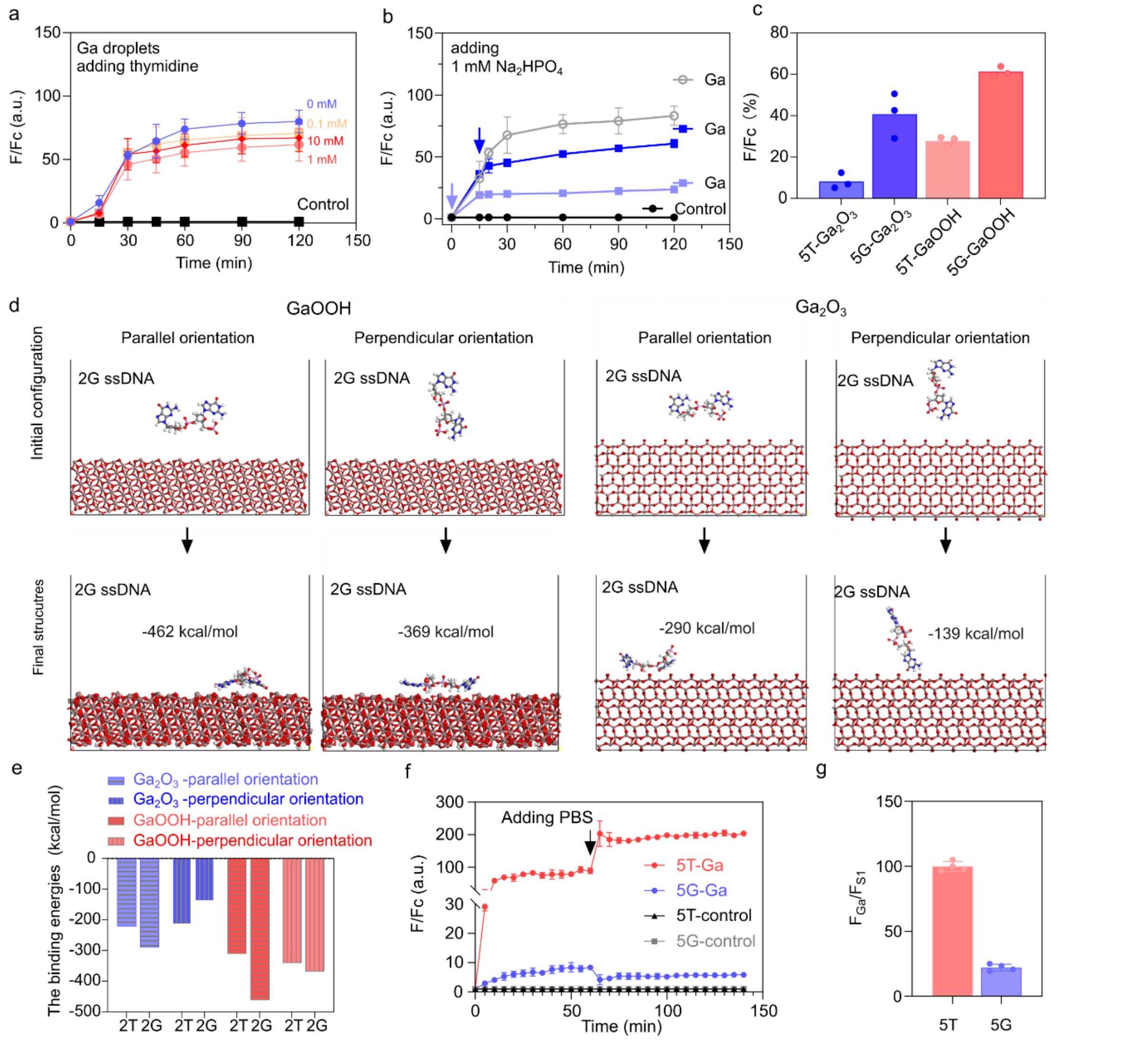

Supplementary Fig. 29. The ssDNA adsorption on Ga droplets and base-sequence preference. a-b, DNA cleavage activity of Ga droplets in the presence of added **(a)** thymidine and **(b)** Na_2HPO_4. **(c)**, Adsorption percentage of 5T ssDNA TR-reporter on Ga oxide surfaces (Ga_2O_3 and GaOOH) (Supplementary Method 3,8). **(d)** Classical molecular dynamics (MD) simulations of 2G ssDNA interacting with Ga oxide layers (Ga_2O_3 and GaOOH) using two initial configurations: ssDNA oriented perpendicular or parallel to the surface (Supplementary Method 6). **(e)** Comparison of binding energies in MD simulations of 2T ssDNA and 2G ssDNA interacting with Ga_2O_3 and GaOOH surfaces. **(f)** DNA cleavage activity of excess Ga droplets (0.5 mg mL^{-1}, fivefold higher than that used in other experiments) toward 5T and 5G ssDNA TR-reporters. Adding PBS pH=7 to promote the de-attach of adsorbed ssDNA

reporter from the surface of Ga droplets (**g**) End-point fluorescence intensity (at 120 min) of 5T and 5G ssDNA TR-reporters cleaved by Ga droplets, normalized to the activity of S1 nuclease.

Supplementary Methods:

Supplementary Method 6. Molecular dynamics (MD) simulations details

Classical MD simulations were performed using the Large-scale Atomic/Molecular Massively Parallel Simulator (LAMMPS) to investigate the interactions between the single-stranded DNA (ssDNA) and the oxide layer surfaces.[12] Force field parameters for the Ga atoms were initially adapted from the Universal Force Field (UFF) parameters for aluminium (Al), given their similar chemical properties as group 13 elements.[13] These parameters were subsequently refined through iterative optimization until the simulated system density was within 10% of the experimental values.

To model the Ga_2O_3 substrate, the (100) surface was cleaved from the most stable β-Ga_2O_3 bulk structure.[14] The optimised surface dimensions were 54.75 × 58.06 × 24.77 $Å^{3,}$ and the model contained 2880 Ga and 4320 O atoms. The GaOOH substrate was constructed using the bulk crystal structure obtained from the Materials Project database and cleaving the (110) surface with dimensions of 45.71 × 62.22 × 14.91 $Å^3$ and containing 1200 Ga, 1200 H, and 2400 O atoms. This surface was chosen as it corresponds to the dominant diffraction peak, as consistently reported in prior studies[1,15]. The optimized force field parameters for both oxides are presented in Supplementary Table 13. After iterative optimization, the systems reached equilibrium densities of 6.05 g/cm^3 for Ga_2O_3 and 4.68 g/cm^3 for GaOOH after 5 ns *NPT* simulations (1 fs timestep), in good agreement with experimental values of 5.89 g/cm^3 and 5.20 g/cm^3, respectively.

The ssDNA-water layer was constructed by randomly distributing water molecules to achieve a density of approximately 1.0 g cm^{-3} to account for solvent effects. This ssDNA-water layer was then added to the simulation boxes of the Ga_2O_3 and GaOOH substrates. Force field parameters for the ssDNA and the water molecules were taken from the AMBER force field.[16] Molecular dynamics simulations were performed for 5 ns using the *NVT* ensemble at 305 K for 8 systems: Ga_2O_3-ssDNA-two thymine (2T), Ga_2O_3-ssDNA-two guanine (2G), GaOOH-ssDNA-2T, and GaOOH-ssDNA-2G, with each system simulated with the ssDNA initially oriented parallel and perpendicular to the surfaces. Non-bonded bonded intramolecular interactions were handled using AMBER scaling: 1-2 and 1-3 interactions were excluded, while 1-4 electrostatic and van der Waals interactions were scaled by factors of 1/1.2 and 1/2, respectively.[17] The temperature was maintained using the Nosé-Hoover thermostat[18,19]. Non-bonded interactions were treated with cutoff distances of 8.5 Å for van der Waals forces and 15 Å for electrostatic interactions. Geometric mixing rules were used for the pairwise van der Waals interactions. All simulations employed a 1 fs timestep, with atomic coordinates recorded every 10 ps for the trajectory analysis. Binding energies (E_{bin}) were calculated using the following equation:

$$E_{bin} = E_{total} - E_{substrate} - E_{solvated\_DNA}$$

where E_{total} is the total energy of the combined system after 5 ns *NVT* equilibration, $E_{substrate}$ is the equilibrated energy of the isolated substrate (Ga_2O_3 or GaOOH), and $E_{solvated\_DNA}$ is the energy of the solvated ssDNA layer. All binding energies were calculated in the presence of explicit water molecules to account for solvation effects.

Supplementary Discussion 5. MD simulation for ssDNA and Ga oxide interaction

To investigate the binding interactions between the ssDNA and Ga_2O_3/GaOOH substrates in different initial orientations, we performed MD simulations with explicit solvation (Fig. 4f and Supplementary Fig.29 d). After equilibration (5 ns *NVT* at 305 K), we analysed the interfacial interactions from the

final frame of each trajectory and calculated the corresponding binding energies (Fig. 4f and Supplementary Fig.29d). The water molecules were made invisible for clarity of visualization.

For ssDNA-2T on Ga_2O_3 (Fig.4f), both initial orientations (parallel and perpendicular) converged to parallel configurations, with the phosphate backbone preferentially aligned towards surface O atoms. The binding energies were -222 kcal mol^{-1} and -214 kcal mol^{-1} for the two structures, respectively. In contrast, ssDNA-2G exhibited a stronger affinity to Ga_2O_3, with the initially parallel orientation (Supplementary Fig.29 d) maintaining a V-shaped conformation by changing its torsion angles (binding energy: -290 kcal mol^{-1}), while the initially perpendicular orientation adopted a tilted configuration on the surface (binding energy: -139 kcal mol^{-1}). The binding energy of this structure was weaker due to fewer functional groups interacting with the surface. The enhanced binding of ssDNA-2G compared to ssDNA-2T can be attributed to the greater number of aromatic rings in the guanine bases, which provide stronger van der Waals interactions with the Ga_2O_3 substrate.

For ssDNA interacting with the GaOOH substrate, the binding was notably stronger than with Ga_2O_3. For ssDNA-2T (Fig.4f), both initial orientations converged to parallel configurations with binding energies of −311 kcal mol^{-1} and -341 kcal mol^{-1}. In comparison, ssDNA-2G displayed a stronger affinity for the GaOOH surface than ssDNA-2T, adopting a parallel orientation regardless of its initial configuration (Supplementary Fig.29d). Both structures had binding energies of -462 kcal mol^{-1} (initially parallel) and -369 kcal mol^{-1} (initially perpendicular). The consistently stronger binding energies for a parallel to the surface indicate greater thermodynamic stability as a result of increased contact area and enhanced interactions due to the type of functional group.

Overall, the MD simulations demonstrate that: (i) ssDNA binds more strongly to GaOOH than Ga_2O_3, and (ii) ssDNA-2G exhibits enhanced substrate affinity compared to ssDNA-2T due to the additional aromatic rings that enhance van der Waals interactions.

Supplementary Discussion:

Supplementary Discussion 7. Possible mechanism of Ga droplets' preference to T-T and A-A

The adsorption behavior of ssDNA on Ga droplets was further examined using competitive inhibition by thymidine and phosphate ions. Previous studies have established that inhibition by free nucleosides (e.g., thymidine) indicates adsorption through sugar or nucleobase moieties[20], whereas inhibition by phosphate ions implies interaction with the phosphodiester backbone[21]. The results show that Ga droplets' DNA cleavage activity was selectively suppressed by phosphate ions but not by thymidine (Supplementary Fig.29 a, b). Combined with MD simulation results showing that ssDNA adopts a largely flat adsorption configuration on Ga oxide layer and DFT results indicating preferential binding of the phosphodiester group to Ga^0, these findings indicate that the ssDNA molecule adsorbs as a whole onto the Ga surface, while interactions involving the phosphodiester backbone play the dominant role in the subsequent cleavage.

To clarify the relationship between adsorption and the observed cleavage preference toward 5T vs 5G sequences, quantitative adsorption experiments were performed (Supplementary Method 8,9). The results show that GaOOH exhibits stronger ssDNA adsorption than Ga_2O_3, and that both oxide surfaces display higher affinity for 5G ssDNA compared to 5T ssDNA (Supplementary Fig.29c). Consistently, classical molecular dynamics (MD) simulations reveal that (i) ssDNA binds more strongly to GaOOH than to Ga_2O_3, and (ii) 2G ssDNA exhibits higher binding affinity than 2T ssDNA, attributable to additional aromatic rings that enhance van der Waals interactions (Fig.4f; Supplementary Fig.29d,e). In the DFT calculations, 5T and 5G exhibit comparable adsorption strengths to the Ga^0 atom, with a slight preference for 5T (Supplementary Fig. 27 b,c).

To exclude the possibility that reduced cleavage of 5G arises from overly strong adsorption leading to slow desorption and blockage of reactive sites on Ga droplets, we conducted cleavage experiments

using excess Ga droplets to provide abundant reactive interfaces, followed by PBS addition to promote ssDNA desorption. Under these conditions, Ga droplets still exhibited a clear cleavage preference for T-rich sequences over G-rich sequences (Supplementary Fig.29f). Moreover, when normalized to the activity of the commercial nuclease S1, Ga droplets retained a stronger relative cleavage efficiency toward 5T ssDNA over 5G ssDNA (Supplementary Fig.29g). These results indicate that the observed cleavage preference originates from adsorption and binding configuration difference, rather than from desorption-limited kinetics.

Based on these results, we propose the following mechanism: Both experimental results and MD simulations show that, on Ga oxide surfaces (Ga_2O_3 and GaOOH), 2G ssDNA exhibits stronger adsorption than 2T ssDNA, whereas Ga droplets display lower cleavage efficiency toward 2G.

This suggests that DNA adsorption strength does not correlate linearly with cleavage efficiency. One possible reason is that strong adsorption of 2G induces pronounced conformational distortions, as evidenced by torsional angle changes and distorted structures (Supplementary Fig. 29d). Such structural deformations are likely to impede cleavage by altering the geometry of the phosphodiester bond, introducing steric barriers to ·OH access, and restricting the conformational flexibility required for efficient electron transfer. In contrast, 2T ssDNA maintains a more native and less distorted conformation, preserving the structural accessibility of the phosphodiester linkage (Fig. 4f) and thereby enabling more efficient cleavage. Another possible reason is that differences in interfacial electron redistribution. Following initial adsorption, Ga^0-2T and Ga^0-2G complexes exhibit different electronic redistribution patterns (Supplementary Fig.27d). In both cases, electrons are transferred from Ga to non-bridging phosphate oxygens; however, Ga^0-2T displays a more moderate and delocalized redistribution, resulting in a less stabilized phosphate environment and increased susceptibility to cleavage.

Supplementary Tables:

Supplementary Table 13. MD calculation results

Supplementary Table 13-1. Lenard-Jones parameters for Ga_2O_3

Element	Epsilon (kca mol^{-1})	Sigma (Å)	Charges (e)
Ga	0.505000	4.008153	1.20
O	0.170000	3.000012	-0.80

Supplementary Table 13-2. Bond parameters for Ga_2O_3

Bond	K (kcal mol^{-1} $Å^{-2}$)	r_0 (Å)
Ga-O	800.0000	1.9485

Supplementary Table 13-3. Angle parameters for Ga_2O_3

Angle	K (kcal mol^{-1} rad^{-2})	q_0 (degrees)
O-Ga-O	119.50287	106.960
Ga-O-Ga	119.50287	121.946

Supplementary Table 13-4. Lenard-Jones parameters for GaOOH

Element	Epsilon (kca mol^{-1})	Sigma (Å)	Charges (e)
Ga	0.505000	4.008153	0.450000

H	0.000000	0.000000	0.090000
OH	0.210400	3.066000	-0.240000
Oz	0.170000	3.000012	-0.300000

Supplementary Table 13-5. Bond parameters for GaOOH

Bond	*K* (kcal mol^{-1} $Å^{-2}$)	r_0 (Å)
Ga-OH	597.500	2.05140
Ga-Oz	597.500	1.94647
H-OH	742.800	0.97000

Supplementary Table 13-6. Angle parameters for GaOOH

Angle	*K* (kcal mol^{-1} rad^{-2})	q_0 (degrees)
OH-Ga-OH	119.50287	76.926
Ga-OH-H	119.50287	111.988
Ga-OH-Ga	119.50287	103.074

3-8. The paper refers to "nuclease-mimicking activity," but, from a non-specialist perspective, it would be valuable to compare the results with actual enzymes using catalytic numbers or other relevant metrics.

Response: Thank you for this important question. To facilitate comparison with enzymatic systems, we quantified the activity of Ga droplets using kinetic parameters commonly employed in chemical catalysis, including the Michaelis constant (Km) values, turnover number (TON), and activation energy. The activation energy is comparable to activation energies reported for radical-mediated Fenton-type reactions (typically ~20–40 kJ·mol^{-1})[42] and falls within the range of apparent activation energies observed for enzymatic nucleases and artificial nuclease systems (~10–60 kJ·mol^{-1})[43,44] while the TON is considered low. More details can be found in our response to your question **Q3-1.**

To further place the performance of Ga droplets in context for non-specialist readers, we directly compared their DNA cleavage behaviour with three widely used commercial nucleases-S1 nuclease, Benzonase, and DNase I-under comparable experimental conditions. This comparative analysis (Supplementary Method 12, Supplementary Fig. 39, and Supplementary Table 16) demonstrates that, despite their non-enzymatic nature, Ga droplets exhibit robust nuclease-mimicking activity and broad operational versatility across different conditions.

Key findings include: 1) High performance: Ga droplets (~250 nm) match the efficiency of S1 nuclease and outperform Benzonase and DNase I under standard conditions. 2) Extreme robustness: Unlike traditional enzymes, they require no cofactors and remain active across high temperatures (up to 70 °C) and tolerate EDTA. 3) External control: Their activity can be uniquely restarted or boosted in inhibitory environments (like PBS) using 808 nm laser irradiation. 4) economic advantage: They provide comparable results at roughly 1% of the cost of commercial protein-based nucleases.

The following discussion and summary table have been added to the Main manuscript and Supplementary information:

Main manuscript (page 14):

Furthermore, systematic comparisons in both DI water and PBS against commercial nucleases (S1 nuclease, Benzonase, and DNase I; Supplementary Fig. 39; Supplementary Table 16); Supplementary Method 12) established Ga droplets as a distinct and efficient nuclease-mimic platform. In DI water, Ga droplets (0.1 mg mL^{-1}, ~330 nm) achieved approximately one-third of the cleavage efficiency of S1 nuclease, while size reduction to ~250 nm enabled cleavage efficiencies comparable to S1 and exceeding those of Benzonase and DNase I. In PBS, phosphate ions suppressed the activity of both S1 nuclease and Ga droplets, whereas Benzonase retained partial activity; critically, unlike protein enzymes, Ga droplets can be reactivated and even outperform commercial nucleases upon external stimulation, exemplified here by 808 nm laser irradiation. In dsDNA reporter assays, S1 nuclease showed no activity and DNase I failed to cleave short dsDNA fragments (<5-6 bp), consistent with its intrinsic substrate and length constraints[4], whereas Ga droplets maintained endonuclease-like activity across ssDNA and dsDNA substrates. Beyond their tunable activity (Fig. 5a), wide temperature ranges (Fig.5f; Supplementary Fig.3), high ionic strength (Supplementary Fig. 33), and EDTA-containing environments (Fig. 3b), Ga droplets achieve comparable cleavage efficiencies at ~1% of the cost. Importantly, in contrast to many radical-generating artificial nucleases that cause extensive base oxidation and sugar-ring degradation[5,6], Ga droplets predominantly yield hydrolysis-like cleavage products, preserving nucleobase and sugar-ring integrity while selectively cleaving the phosphodiester backbone.

Together, the sensitivity of Ga droplets to inhibitors such as phosphate, together with their reactivation by physical and chemical triggers, including pH adjustment, bicarbonate addition, and light irradiation *via* modulation of surface oxide integrity, highlights a unique, switchable, and programmable nuclease-mimic behavior.

Supplementary Figures:

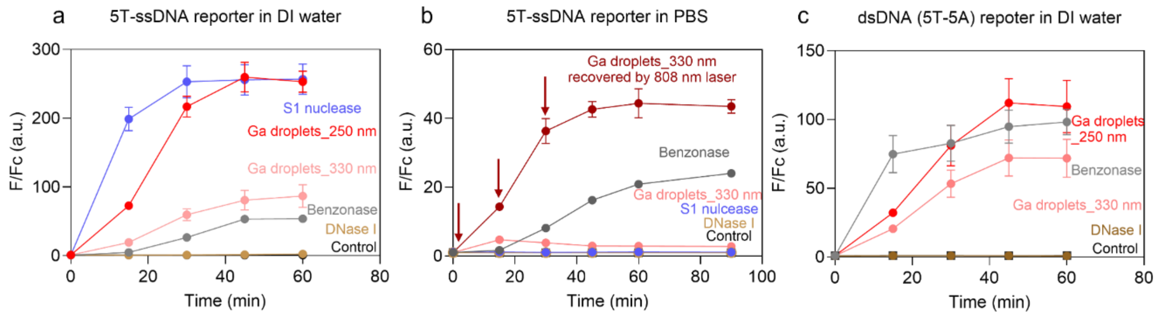

Supplementary Fig. 39. Comparison of the DNA cleavage activity of Ga droplets and commercial nucleases (S1 nuclease, Benzonase, DNase I). Ga droplets with mean diameters of ~330 nm and ~250 nm were synthesized by probe sonication for 30 min and 120 min, respectively.

Supplementary Methods:

Supplementary Method 12. Commercial nuclease activity

S1 nuclease, Benzonase, and DNase I digestions were performed according to the manufacturers' instructions. S1 nuclease reactions were conducted in acetate-based buffer containing zinc ions (Zn^{2+}), while Benzonase and DNase I reactions were in Tris-based buffers supplemented with magnesium ions (Mg^{2+}) and calcium ion (Ca^{2+}) for DNase I). All reactions were incubated at 37 °C for the indicated time. Reporter DNA concentrations were identical to those used in the Ga-droplet experiments (100 µL, 166 nM per well; 0.024 µg ssDNA per well). Nucleases were added at 0.033 U per well (1 U defined as activity per 1 µg DNA).

Supplementary Tables:

Supplementary Table 16. Comparison between Ga droplets as nuclease mimics and commercial nucleases. *The information was compiled from the official product manuals of Sigma-Aldrich, New England Biolabs, and Thermo Fisher Scientific.

	S1 Nuclease	Benzonase	DNase I	Ga droplets (~330 nm)
Description & Source	endonuclease from *Aspergillus* (fungal)	Broad-spectrum endonuclease from *Serratia marcescens* (bacterial)	Non-specific deoxyribonuclease from bovine pancreas	Metallic -Oxide micro/nanodroplets
Substrate Specificity	ssDNA > >dsDNA; Endonuclease	dsDNA, ssDNA; Endonuclease	dsDNA (100-500 x over ssDNA); length > 6bp; Endonuclease	dsDNA, ssDNA, Endonuclease-like/nuclease-mimic cleavage
Temperature	Optimal at ~37 °C.	Optimal temp 37 °C (active 0–42 °C).	Optimal at ~37 °C.	Broad temperature tolerance (0–70 °C); cleavage activity
Ion tolerance	Salt Inhibition>100 mM monovalent salts (Na+, K+) Phosphate Inhibition>1 mM	Salt Inhibition>300 mM monovalent salts (Na+, K+) Phosphate Inhibition>20 mM	Salt Inhibition>20 mM. Phosphate Inhibition>1 mM	Salt Inhibition>300 mM monovalent salts (Na+, K+)； Phosphate Inhibition>1 mM； can be recovered
EDTA tolerance	No	No	No	Yes
Price	Sigma_N5661-50KU 401 AUD	Sigma_E8263-5KU 358 AUD	Biolabs_M0303S_1000U 152 AUD	~2.4 AUD / g
Cost required to achieve the effect in Supplementary Fig.39	0.033U/166 nM 0.145 AUD/µM ssDNA	0.033U/166 nM 0.0133 AUD/µM ssDNA	0.033U/166 nM 0.0277 AUD/µM ssDNA	0.033U/166 nM 0.00145 AUD/µM ssDNA

References

1 Lin, Y., Liu, Y., Genzer, J. & Dickey, M. D. Shape-transformable liquid metal nanoparticles in aqueous solution. *Chem. Sci.* **8**, 3832-3837 (2017). https://doi.org/10.1039/C7SC00057J

2 Creighton, M. A. *et al.* Oxidation of Gallium-based Liquid Metal Alloys by Water. *Langmuir* **36**, 12933-12941 (2020). https://doi.org/10.1021/acs.langmuir.0c02086

3 Gan, T., Handschuh-Wang, S., Shang, W. & Zhou, X. GaOOH Crystallite Growth on Liquid Metal Microdroplets in Water: Influence of the Local Environment. *Langmuir* **38**, 14475-14484 (2022). https://doi.org/10.1021/acs.langmuir.2c02539

4 Weston, S. A., Lahm, A. & Suck, D. X-ray structure of the DNase I-d(GGTATACC)2 complex at 2·3Å resolution. *J. Mol. Biol.* **226**, 1237-1256 (1992). https://doi.org/https://doi.org/10.1016/0022-2836(92)91064-V

5 Chattopadhyaya, R. Oxidative damage to DNA constituents by iron-mediated Fenton reactions--the thymidine family. *J Biomol Struct Dyn* **32**, 155-169 (2014). https://doi.org/10.1080/07391102.2012.745167

6 Jiang, Q., Xiao, N., Shi, P., Zhu, Y. & Guo, Z. Design of artificial metallonucleases with oxidative mechanism. *Coord. Chem. Rev.* **251**, 1951-1972 (2007). https://doi.org/10.1016/j.ccr.2007.02.013

7 Burrows, C. J. & Muller, J. G. Oxidative Nucleobase Modifications Leading to Strand Scission. *Chem. Rev.* **98**, 1109-1152 (1998). https://doi.org/10.1021/cr960421s

8 Ni, J., Pomerantz, C., Rozenski, J., Zhang, Y. & McCloskey, J. A. Interpretation of oligonucleotide mass spectra for determination of sequence using electrospray ionization and tandem mass spectrometry. *Anal. Chem.* **68**, 1989-1999 (1996). https://doi.org/10.1021/ac960270t

9 Potier, N., Van Dorsselaer, A., Cordier, Y., Roch, O. & Bischoff, R. Negative electrospray ionization mass spectrometry of synthetic and chemically modified oligonucleotides. *Nucleic Acids Res.* **22**, 3895-3903 (1994). https://doi.org/10.1093/nar/22.19.3895

10 Malakooti, M. H. *et al.* Liquid Metal Supercooling for Low-Temperature Thermoelectric Wearables. *Adv. Funct. Mater.* **29**, 1906098 (2019). https://doi.org/https://doi.org/10.1002/adfm.201906098

11 Zhang, C. *et al.* Study on the nucleating agents for gallium to reduce its supercooling. *Int. J. Heat Mass Transfer* **148**, 119055 (2020). https://doi.org/https://doi.org/10.1016/j.ijheatmasstransfer.2019.119055

12 Plimpton, S. J., Kohlmeyer, A., Thompson, A. P., Moore, S. G. & Berger, R. *LAMMPS: Large-scale Atomic/Molecular Massively Parallel Simulator*. (Zenodo, 2023).

13 Rappe, A., Colwell, K. & Casewit, C. Application of a universal force field to metal complexes. *Inorg. Chem.* **32**, 3438-3450 (1993).

14 Tang, J. *et al.* Unique surface patterns emerging during solidification of liquid metal alloys. *Nat. Nanotechnol.* **16**, 431-439 (2021). https://doi.org/10.1038/s41565-020-00835-7

15 Hsieh, Y.-S. *et al.* Investigation of metal-insulator-semiconductor diode with alpha-Ga2O3 insulating layer by liquid phase deposition. *Thin Solid Films* **685**, 414-419 (2019). https://doi.org/https://doi.org/10.1016/j.tsf.2019.06.044

16 Wang, J., Wolf, R. M., Caldwell, J. W., Kollman, P. A. & Case, D. A. Development and testing of a general amber force field. *J. Comput. Chem.* **25**, 1157-1174 (2004). https://doi.org/https://doi.org/10.1002/jcc.20035

17 Yang, L. *et al.* New-generation amber united-atom force field. *J. Phys. Chem. B* **110**, 13166-13176 (2006).

18 Evans, D. J. & Holian, B. L. The Nose–Hoover thermostat. *J. Chem. Phys.* **83**, 4069-4074 (1985). https://doi.org/10.1063/1.449071

19 Martyna, G. J., Klein, M. L. & Tuckerman, M. Nosé–Hoover chains: The canonical ensemble via continuous dynamics. *J. Chem. Phys.* **97**, 2635-2643 (1992).

https://doi.org/10.1063/1.463940
20 Lu, C., Liu, Y., Ying, Y. & Liu, J. Comparison of MoS2, WS2, and Graphene Oxide for DNA Adsorption and Sensing. *Langmuir* **33**, 630-637 (2017). https://doi.org/10.1021/acs.langmuir.6b04502
21 Liu, G. *et al.* Revealing the infiltration process and retention mechanisms of surface applied free DNA tracer through soil under flood irrigation. *Sci. Total Environ.* **905**, 167378 (2023). https://doi.org/https://doi.org/10.1016/j.scitotenv.2023.167378
22 Zavabeti, A. *et al.* A liquid metal reaction environment for the room-temperature synthesis of atomically thin metal oxides. *Science* **358**, 332-335 (2017). https://doi.org/doi:10.1126/science.aao4249
23 Rashid-Nadimi, S. *et al.* The Role of Electrochemical Oxidation on the Interfacial Tension of Eutectic Gallium Indium. *ACS Electrochem.* **1**, 103-114 (2025). https://doi.org/10.1021/acselectrochem.4c00088
24 Rahim, M. A. *et al.* Low-temperature liquid platinum catalyst. *Nat. Chem.* **14**, 935-941 (2022). https://doi.org/10.1038/s41557-022-00965-6
25 Tang, J. *et al.* Liquid-Metal-Enabled Mechanical-Energy-Induced CO_2 Conversion. *Adv. Mater.* **34**, 2105789 (2022). https://doi.org/https://doi.org/10.1002/adma.202105789
26 Idrus-Saidi, S. A. *et al.* Liquid metal synthesis solvents for metallic crystals. *Science* **378**, 1118-1124 (2022). https://doi.org/10.1126/science.abm2731
27 Fu, M. *et al.* Gallium-based liquid metal micro/nanoparticles for photothermal cancer therapy. *J. Mater. Sci. Technol.* **142**, 22-33 (2023). https://doi.org/https://doi.org/10.1016/j.jmst.2022.08.049
28 Liu, T. *et al.* Photothermal photodynamic therapy and enhanced radiotherapy of targeting copolymer-coated liquid metal nanoparticles on liver cancer. *Colloids Surf. B Biointerfaces* **207**, 112023 (2021). https://doi.org/10.1016/j.colsurfb.2021.112023
29 Pei, S., You, S., Ma, J., Chen, X. & Ren, N. Electron Spin Resonance Evidence for Electro-generated Hydroxyl Radicals. *Environ. Sci. Technol.* **54**, 13333-13343 (2020). https://doi.org/10.1021/acs.est.0c05287
30 Clément, J.-L. *et al.* Assignment of the EPR Spectrum of 5,5-Dimethyl-1-pyrroline N-Oxide (DMPO) Superoxide Spin Adduct. *J. Org. Chem.* **70**, 1198-1203 (2005). https://doi.org/10.1021/jo048518z
31 Villamena, F. A., Merle, J. K., Hadad, C. M. & Zweier, J. L. Superoxide Radical Anion Adduct of 5,5-Dimethyl-1-pyrroline N-Oxide (DMPO). 1. The Thermodynamics of Formation and Its Acidity. *J. Phys. Chem. A* **109**, 6083-6088 (2005). https://doi.org/10.1021/jp052431f
32 Diaz-Uribe, C. E. *et al.* Visible light superoxide radical anion generation by tetra(4-carboxyphenyl)porphyrin/TiO2: EPR characterization. *J. Photochem. Photobiol. A* **215**, 172-178 (2010). https://doi.org/https://doi.org/10.1016/j.jphotochem.2010.08.013
33 Burchill, C. E. & Ginns, I. S. The radiation-induced oxidation of ethanol and methanol by hydrogen peroxide in aqueous solution. *Can. J. Chem.* **48**, 2628-2632 (1970). https://doi.org/10.1139/v70-441
34 Villamena, F. A., Merle, J. K., Hadad, C. M. & Zweier, J. L. Superoxide Radical Anion Adduct of 5,5-Dimethyl-1-pyrroline N-Oxide (DMPO). 2. The Thermodynamics of Decay and EPR Spectral Properties. *J. Phys. Chem. A* **109**, 6089-6098 (2005). https://doi.org/10.1021/jp0524330
35 Huang, X. *et al.* Photolysis of Dissolved Organic Matter over Hematite Nanoplatelets. *Environ Sci Technol* **58**, 2798-2807 (2024). https://doi.org/10.1021/acs.est.3c08752
36 Sakamoto, T. & Imai, H. Hydrogen peroxide produced by superoxide dismutase SOD-2 activates sperm in Caenorhabditis elegans. *J. Biol. Chem.* **292**, 14804-14813 (2017). https://doi.org/10.1074/jbc.M117.788901
37 Hyland, K. & Auclair, C. The formation of superoxide radical anions by a reaction between O2, OH− and dimethyl sulfoxide. *Biochem. Biophys. Res. Commun.* **102**, 531-537 (1981). https://doi.org/https://doi.org/10.1016/0006-291X(81)91552-7
38 Bansal, S. & Wang, B. A critical factor in reactive oxygen species (ROS) studies: the

need to understand the chemistry of the solvent used: the case of DMSO. *Chem. Sci.* **15**, 17843-17851 (2024). https://doi.org/10.1039/D4SC05038J
39 Keller, B. O., Sui, J., Young, A. B. & Whittal, R. M. Interferences and contaminants encountered in modern mass spectrometry. *Anal. Chim. Acta* **627**, 71-81 (2008). https://doi.org/10.1016/j.aca.2008.04.043
40 Corporation, M. Sample Preparation of Oligonucleotides Prior to MALDI-TOF MS Using ZipTipC18 and ZipTipμ-C18 Pipette Tips. (Millipore Corporation, Billerica, MA, USA).
41 Luong, T. K. N., Shestakova, P. & Parac-Vogt, T. N. Kinetic studies of phosphoester hydrolysis promoted by a dimeric tetrazirconium(iv) Wells–Dawson polyoxometalate. *Dalton Trans.* **45**, 12174-12180 (2016). https://doi.org/10.1039/C6DT02211A
42 Kumar, A., Pottiboyina, V. & Sevilla, M. D. Hydroxyl Radical (OH•) Reaction with Guanine in an Aqueous Environment: A DFT Study. *J. Phys. Chem. B* **115**, 15129-15137 (2011). https://doi.org/10.1021/jp208841q
43 Klvaňa, M., Bren, U. & Florián, J. Uniform Free-Energy Profiles of the P-O Bond Formation and Cleavage Reactions Catalyzed by DNA Polymerases β and λ. *J Phys Chem B* **120**, 13017-13030 (2016). https://doi.org/10.1021/acs.jpcb.6b08581
44 Van, R. *et al.* Exploring CRISPR-Cas9 HNH-Domain-Catalyzed DNA Cleavage Using Accelerated Quantum Mechanical Molecular Mechanical Free Energy Simulation. *Biochem.* **64**, 289-299 (2025). https://doi.org/10.1021/acs.biochem.4c00651
45 Xie, W. *et al.* Gallium-Based Liquid Metal Particles for Therapeutics. *Trends Biotechnol.* **39**, 624-640 (2021). https://doi.org/10.1016/j.tibtech.2020.10.005
46 Tang, S.-Y., Tabor, C., Kalantar-Zadeh, K. & Dickey, M. D. Gallium Liquid Metal: The Devil's Elixir. *Annu. Rev. Mater. Res.* **51**, 381-408 (2021). https://doi.org/10.1146/annurev-matsci-080819-125403
47 Goff, A. *et al.* An exploration into two-dimensional metal oxides, and other 2D materials, synthesised via liquid metal printing and transfer techniques. *Dalton Trans.* **50**, 7513-7526 (2021). https://doi.org/10.1039/D0DT04364H
48 Lu, H. *et al.* Nanoengineering Liquid Metal Core–Shell Nanostructures. *Adv. Funct. Mater.* **34**, 2311300 (2024). https://doi.org/https://doi.org/10.1002/adfm.202311300
49 Hsieh, T.-E., Frisch, J., Wilks, R. G. & Bär, M. Unravelling the Surface Oxidation-Induced Evolution of the Electronic Structure of Gallium. *ACS Appl. Mater. Interfaces* **15**, 47725-47732 (2023). https://doi.org/10.1021/acsami.3c09324
50 Ding, Y., Zeng, M. & Fu, L. Surface Chemistry of Gallium-Based Liquid Metals. *Matter* **3**, 1477-1506 (2020). https://doi.org/10.1016/j.matt.2020.08.012
51 Gabovich, A. M., Kuznetsov, V. I. & Voitenko, A. I. Tunneling as a marker of quantum mechanics. *Low Temp. Phys.* **50**, 925-947 (2024). https://doi.org/10.1063/10.0030409
52 Lin, Y., Liu, Y., Genzer, J. & Dickey, M. D. Shape-transformable liquid metal nanoparticles in aqueous solution. *Chem. Sci.* **8**, 3832-3837 (2017). https://doi.org/10.1039/c7sc00057j
53 He, J., Shi, F., Wu, J. & Ye, J. Shape Transformation Mechanism of Gallium–Indium Alloyed Liquid Metal Nanoparticles. *Adv. Mater. Interf.* **8**, 2001874 (2021). https://doi.org/https://doi.org/10.1002/admi.202001874
54 Ye, S. *et al.* Oxidation-Induced Oxide Shell Rupture and Phase Separation in Eutectic Gallium–Indium Nanoparticles. *ACS Nano* **18**, 25107-25117 (2024). https://doi.org/10.1021/acsnano.4c06764
55 Chiu, S.-H. *et al.* Electrochromic PEDOT:PSS with Embedded Liquid Gallium Nanoparticles. *ACS Appl. Mater. Interfaces* **17**, 43968-43978 (2025). https://doi.org/10.1021/acsami.5c13143
56 Li, J. *et al.* Vanadium Dioxide Nanocoating Induces Tumor Cell Death through Mitochondrial Electron Transport Chain Interruption. *Glob. Challenges* **3**, 1800058 (2019). https://doi.org/https://doi.org/10.1002/gch2.201800058
57 Mancin, F., Scrimin, P. & Tecilla, P. Progress in artificial metallonucleases. *Chem. Commun.* **48**, 5545-5559 (2012). https://doi.org/10.1039/c2cc30952a

58 Codorniu-Hernández, E. & Kusalik, P. G. Mobility Mechanism of Hydroxyl Radicals in Aqueous Solution via Hydrogen Transfer. *J. Am. Chem. Soc.* **134**, 532-538 (2012). https://doi.org/10.1021/ja208874t
59 Sträter, N., Lipscomb, W. N., Klabunde, T. & Krebs, B. Two-Metal Ion Catalysis in Enzymatic Acyl- and Phosphoryl-Transfer Reactions. *Angew. Chem. Int. Ed.* **35**, 2024-2055 (1996). https://doi.org/https://doi.org/10.1002/anie.199620241
60 Weston, J. Mode of Action of Bi- and Trinuclear Zinc Hydrolases and Their Synthetic Analogues. *Chem. Rev.* **105**, 2151-2174 (2005). https://doi.org/10.1021/cr020057z
61 Mancin, F., Scrimin, P., Tecilla, P. & Tonellato, U. Artificial metallonucleases. *Chem. Commun.*, 2540-2548 (2005). https://doi.org/10.1039/b418164f
